# A genomic history of the North Pontic Region from the Neolithic to the Bronze Age

**DOI:** 10.1038/s41586-024-08372-2

**Published:** 2025-02-05

**Authors:** Alexey G. Nikitin, Iosif Lazaridis, Nick Patterson, Svitlana Ivanova, Mykhailo Videiko, Valentin Dergachev, Nadiia Kotova, Malcolm Lillie, Inna Potekhina, Marta Krenz-Niedbała, Sylwia Łukasik, Serhij Makhortykh, Virginie Renson, Henry Shephard, Gennadie Sirbu, Sofiia Svyryd, Taras Tkachuk, Piotr Włodarczak, Kim Callan, Elizabeth Curtis, Eadaoin Harney, Lora Iliev, Aisling Kearns, Ann Marie Lawson, Megan Michel, Matthew Mah, Adam Micco, Jonas Oppenheimer, Lijun Qiu, J. Noah Workman, Fatma Zalzala, Swapan Mallick, Nadin Rohland, David Reich

**Affiliations:** 1 Department of Biology, Grand Valley State University, Allendale, MI, USA; 2 Department of Human Evolutionary Biology, Harvard University, Cambridge, MA, USA; 3 Department of Genetics, Harvard Medical School, Boston, MA, USA; 4 Broad Institute of MIT and Harvard, Cambridge, MA, USA; 5 Institute of Archaeology, National Academy of Sciences of Ukraine, Kyiv, Ukraine; 6 Scientific Research Laboratory of Archaeology, Borys Grinchenko Kyiv University, Kyiv, Ukraine; 7 Center of Archaeology, Institute of Cultural Heritage, Academy of Science of Moldova, Chișinău, Moldova; 8 Faculty of Biology, Adam Mickiewicz University in Poznań, Poznań, Poland; 9 University of Missouri Research Reactor, Columbia, MO, USA; 10 Archaeological Institute of America, Boston, MA, USA; 11 Thracology Scientific Research Laboratory of the State University of Moldova, Department of Academic Management, Academy of Science of Moldova, Chișinău, Moldova; 12 Museum of History of Ancient Halych, “An Ancient Halych” National Reserve, Halych, Ukraine; 13 Institute of Archaeology and Ethnology, Polish Academy of Sciences, Kracow, Poland; 14 Howard Hughes Medical Institute, Harvard Medical School, Boston, MA, USA

## Abstract

The North Pontic Region was the meeting point of the farmers of Old Europe and the foragers and pastoralists of the Eurasian steppe^1,2^, and the source of migrations deep into Europe^3–5^. We report genome-wide data from 81 prehistoric North Pontic individuals to understand the genetic makeup of its people. North Pontic foragers had ancestry not only from Balkan and Eastern hunter-gatherers^6^ but also European farmers and, occasionally, Caucasus hunter-gatherers. During the Eneolithic, a wave of migrants from the Caucasus-Lower Volga (CLV) area^7^ bypassed local foragers to mix in equal parts with Trypillian farmers, forming the people of the Usatove culture around 4500 BCE. A temporally overlapping wave of CLV migrants blended with foragers instead of farmers to form Serednii Stih people^7^. The third wave was the Yamna: descendants of the Serednii Stih who formed by mixture around 4000 BCE and expanded in the Early Bronze Age (3300 BCE). The temporal gap between Serednii Stih and the Yamna is bridged by a genetically Yamna individual from Mykhailivka, Ukraine (3635–3383 BCE), a site of archaeological continuity across the Eneolithic-Bronze Age transition, and a likely epicenter of Yamna formation. Each of these three waves of migration propagated distinctive ancestries while also incorporating outsiders, a flexible strategy that may explain the peoples’ of the North Pontic outsized success in spreading their genes and culture across Eurasia^3–5,8–10^.

The area north of the Black Sea, the North Pontic Region (NPR, Fig. 1, Supplementary File SI1.1), has been proposed as the homeland for communities that developed core Indo-European language terminology^11^, which began spreading across Eurasia facilitated by the turn-of-the-third-millennium BCE expansion of the Yamna archaeological complex (hereafter Yamna)^10^. During preceding times, a diverse array of archaeological groups inhabited the NPR.

Genome-wide ancient DNA studies have revealed that the genetic ancestry of post-glacial hunter-gatherer groups in the NPR was derived from a mixture of ancestries related to “Western Hunter-Gatherers” (WHGs) and the Danubian Iron Gates “Balkan Hunter Gatherers” (BHGs)^6^, to “Eastern Hunter-Gatherers”^3^ (EHGs) in the east. In Ukraine, the Mesolithic-Neolithic transition (after 5800 BCE) was marked by WHG admixture with the EHG ancestry of previously established local populations^6^.

During the Neolithic, the western NPR was home to Balkan and central European farming cultures, such as Criș, Starčevo, and LBK, carrying Early European Farmer (EEF) ancestry, stemming from Anatolian Neolithic Farmers (ANF) with different proportions of WHG admixture^12^. The Neolithic hunter gatherer populations of the Dnipro Valley (Ukraine_N, hereafter UNHG) continued to retain the EHG/WHG-based genetic ancestry^6^.

In the early Eneolithic (ca. 4800 BCE), farming groups of the Cucuteni-Trypillia archaeological complex (hereafter Trypillia) spread eastwards across the Carpathians to the Dnipro Valley^13,14^. The ancestry of Trypillia was primarily EEF-derived with an admixture from WHG^6,15–18^.

During their eastward expansion, Trypillia encountered mobile communities of the Serednii Stih archaeological complex (hereafter Stih)^13^, which likely formed in the Azov-Dnipro-Donets area in the first half of the 5^th^ millennium BCE^19–21^. The presence of early Stih in the Azov steppe around 4700–4500 BCE is supported by strontium isotope analysis of an early Stih individual from the Mariupol necropolis (Supplementary File SI1). However, knowledge about the genetic ancestry of steppe populations like Stih (referred to as “steppe ancestry”^3–6,10,13^) has been limited until now due to small sample sizes which revealed highly variable ancestry^6,13,18^.

In the 4^th^ millennium BCE, a distinctive archaeological complex known as Usatove was established in the northwestern NPR. Sampled Usatove individuals harbored EEF and steppe ancestries, as well as a Caucasus Eneolithic/Maykop-related genetic component^5^, but the proximate sources of the composing ancestries remain unclear. In the second half of the 4^th^ millennium BCE, the NPR was occupied by diverse groups, characterized by distinct burial rites and pottery types/techniques, and increased mobility, possibly including wheeled wagon transportation^2^. This diversity was eclipsed in the last third of the 4^th^ millennium by the expansion of the Yamna, persisting into the first half of the following millennium.

Genetic ancestry data on the Epipaleolithic to Early Bronze Age populations of the NPR come from limited sites, hampering the understanding of population dynamics, particularly in the time preceding the genetic turnover precipitated by Yamna-related people^3,4,10,22^. This report analyzes prehistoric NPR individuals from a much wider selection of archaeological sites than has previously been available, including substantially larger sample sizes from Trypillia, Usatove, and Stih. Co-analyzing with the data reported in the linked paper^7^, we examine the contribution of these groups to the genetic ancestry of Yamna with a particular focus on integrating the results of the present study with the archaeological evidence to produce a holistic picture of genetic and archaeological transformations preceding and following the formation of the Yamna.

## Results

We generated whole genome ancient DNA data for 81 ancient individuals from the NPR from the Neolithic to the Bronze Age (for 76, data are reported for the first time) (Online Table 1). To generate these data, we sampled 206 skeletal elements and built 462 next-generation sequencing libraries; after screening we took 245 forward into analysis (Online Table 2). We enriched our analyses by generating 51 radioEt dates (Online Table 3) and used comparative data to analyze isotopic ratios (Supplementary File SI1.4; Online Table 4). We co-analysed these data with that from an accompanying study of steppe populations including 291 newly reported individuals and 63 individuals with improved data^7^.

**Fig. 2:**
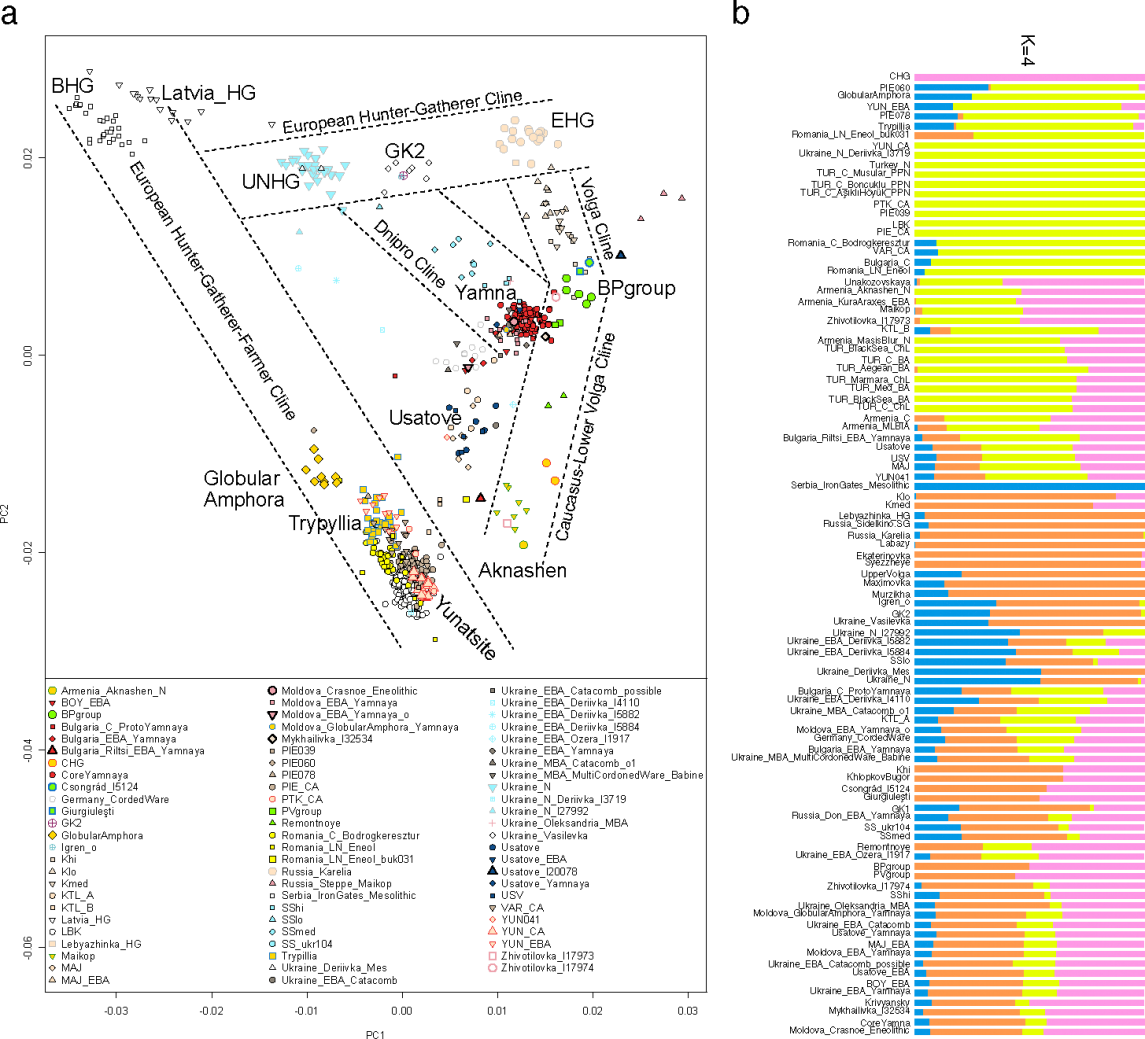
Genetic variation in the North Pontic Region. (a) PCA of the NPR samples in relation to the three steppe clines (Volga, Dnipro, and Caucasus-Lower Volga) and respective samples from^7^. Raw coordinates of the plotted points can be found in Online Table 5. (b) Unsupervised ADMIXTURE summary graph of populations from this report and^7^ (Supplementary File SI3). Components broadly correspond to CHG (pink), Anatolian-European Neolithic (yellow), BHG (blue), and EHG (orange).

We carried out Principal component analysis (PCA)^23^, forming the axes using a set of populations^7^ (Fig. 2a; Methods) designed to capture Siberian-European hunter-gatherer (top) to West Asian (bottom) differentiation and Eastern-Western European (horizontally top) and Inland/Highland Mediterranean (horizontally bottom) differentiation. This analysis reveals five major clines. Four—the Caucasus-Lower Volga (CLV) Cline, the Volga Cline, the Dnipro Cline, and the European Hunter-Gatherer (EuHG) Cline—are described formally in the accompanying study^7^. The fifth, the European Farmer and Hunter Gatherer cline (EFHG), is formed by European farmers (central European LBK and populations related to Gumelnița/Karanovo from the Yunatsite site in Bulgaria (Yunatsite Chalcolithic, YUN_CA), on one side, and BHG (Serbia_IronGates_Mesolithic), on the other (Fig. 2a).^24^

UNHG individuals are located on the “eastern” end of the EuHG cline towards BHG, and the “northern” edge of the Dnipro cline. This suggests that UNHG contributed to later Eneolithic and Bronze Age (BA) people on the Dnipro cline, with Core Yamna^7^ at the “southern” end.

The Eneolithic (apart from the Stih) and BA individuals in Fig. 2a are mostly located towards the “farmer” end of the EFHG cline. Four NPR individuals form a cline between the Core Yamna and steppe Maykop, and while seemingly proximate in PCA to the “BPgroup” population consisting of Eneolithic individuals from Lower Volga Berezhnovka and Caucasus Progress 2, in fact are revealed by qpAdm to be ancestrally different, tracing about half of their ancestry to Siberian/Central Asian Neolithic sources^7^. Two of these (Usatove_I20078 and Zhivotilovka_I17974) are late Eneolithic (3300–3000 BCE) individuals from Moldova. The other two, Csongrád_I5124 from Hungary^7^ and I20072 (Giurgiuleşti) from Moldova, (4300–4000 BCE) are archaeologically associated with the people that left “Ochre Graves” across the NPR and adjacent Balkan-Carpathian area^25,26^.

### Sources of Neolithic NPR ancestry

We computed *f*_*3*_ statistics with UNHG as a target and a wide variety of possible sources (Extended Data Table 1, Supplementary File SI2, Table SI2. 3). The results suggest the UNHG population is, to a first approximation, composed of sources related to EHG and BHG.

However, it is evident from the PCA in Fig. 2a that the UNHG end of the EuHG cline is shifted towards populations with EEF ancestry. In unsupervised ADMIXTURE analysis (Fig. 2b; Supplementary Information File SI3, Fig. SI3. 1), the UNHG are assigned small components of Anatolian Farmer/CHG ancestry, not present in Mesolithic Ukraine (Deriivka), EHG (Karelia) or BHG (Iron Gates) groups. When samples from individuals labeled Ukraine_N (UNHG) are modeled with other EuHG populations from^7^, only a single 2-source model (p=0.576) with 72.5±2.9% GK2 from the Golyubaya Krinitsa site on the Lower Don^7^ and 27.5±2.9% BHG ancestry, remains viable (here and in what follows, we indicate statistical uncertainty through standard errors; a 95% confidence interval corresponds to 1.96 standard errors in either direction of the point estimate). Fitting to a broader cline between EHG and BHG as a mixture of these two sources with either Lebyazhinka or Karelia as the EHG source, fails (p<1e-9) and qpAdm output suggests that these models underestimate shared genetic drift with Turkey_N (Z<−3.5).

Three-source models (Supplementary Information File SI2 (Table SI2. 42)) all include EHG-BHG sources along with ~7–9% of EEF ancestry, the latter accounting for the underestimated drift with Turkey_N in a model without such ancestry.

To test whether EEF ancestry is a general feature of UNHG populations, we fit a model that included central European LBK representing EEF ancestry to 35 individuals with the Ukraine_N label (Extended Data Table 2; Supplementary File SI2, Appendix V). The results show that this pattern is not driven by a few outliers.

The UNHG are inferred to have significant BHG and EHG ancestry, and harbor in *increase* of BHG ancestry relative to Mesolithic individuals from Vasylivka III^6^ and Vasylivka I^27^ (Fig. 2). A migration of people from the Iron Gates area to the Dnipro Valley in the 7^th^ millennium BCE^28^ is thus genetically consistent with being responsible for this shift. As BHG individuals from the Iron Gates has been shown to carry sporadic EEF ancestry^6^, the existence of some Iron Gates-like migrants carrying such ancestry could account for both BHG and EEF admixture compared to Mesolithic Ukraine.

Hunter-gatherers of WHG-EHG mixed background in the Baltic^3,29,30^ do not carry the EEF ancestry we detect in the UNHG (Supplementary File SI2, Appendix V). The Pitted Ware/Battle Axe Culture populations from Ajvide in Sweden^31,32^ and Västerbjers^33^, in which EEF ancestry was incorporated into groups of predominantly hunter-gatherer background, are correctly inferred by our model to have ~1/5 EEF-related ancestry. Our finding of EEF-related ancestry in UNHGs provides a separate and much earlier instance of the incorporation of farmer ancestry into the hunter-gatherer communities at the periphery of the Neolithic expansion in Europe.

UNHG individuals I31730 (Mariupol Necropolis, this report) and I1738 (Vovnigi 2^6^) that failed the LBK-EHG-BHG model can be modeled with CHG instead of LBK as a source (Extended Data Table 2), consistent with CHG-related ancestry occasionally extending past the middle Don ^7,34^ to the Dnipro Valley during the second half of the 6^th^ millennium BCE.^7^

### CLV admixture and long-range mobility

The ancestry of Serednii Stih individuals is examined in detail in ref.^7^ Stih could be modeled with one source being the Core Yamna as the endpoint of the Dnipro cline (a proxy for earlier populations in the Eneolithic from which the Yamna descend^7^), and Dnipro-Don HGs (UNHG or GK2). Because Core Yamna themselves are consistent with being a ~4/5 mixture of CLV cline and Dnipro-Don HG populations^7^, the Stih ancestry formation can be seen as the result of the fusion of CLV cline migrants with Dnipro-Don HGs.

The ancestry of a Stih outlier from Igren-8 (I27930; Igren_o; 4400–4000 BCE)^7^ appears to be similar to the Neolithic GK2 individual (5610–5390 BCE) from the Middle Don^34^ and to Mesolithic hunter-gatherers from Vasylivka 1 and Vasylivka 3^6,27^ (Fig. 2a) and could be modeled as having ~2/3 EHG and ~1/3 BHG ancestry^7^. Individual I27930 thus represents a Neolithic ancestry carry-over in a burial context of Stih^35^, plausibly appearing in the Dnipro Valley as a result of a long-range migration from the Middle Don or continuing the Mesolithic ancestry of the nearby Vasylivka.

Individual I20072 (4330–4058 calBCE) from Giurgiulești on the Lower Danube is cladal with the Lower Volga-North Caucasus Eneolithic groups (Supplementary File SI2, Table SI2. 1). Along with the contemporaneous Csongrád individual from Hungary, they represent an example of long-distance migration, across an even larger range than individual I27930 from Igren (Igren_o), spanning from the Volga to the heart of Central Europe.

### Trypillia and Usatove

**Fig. 3:**
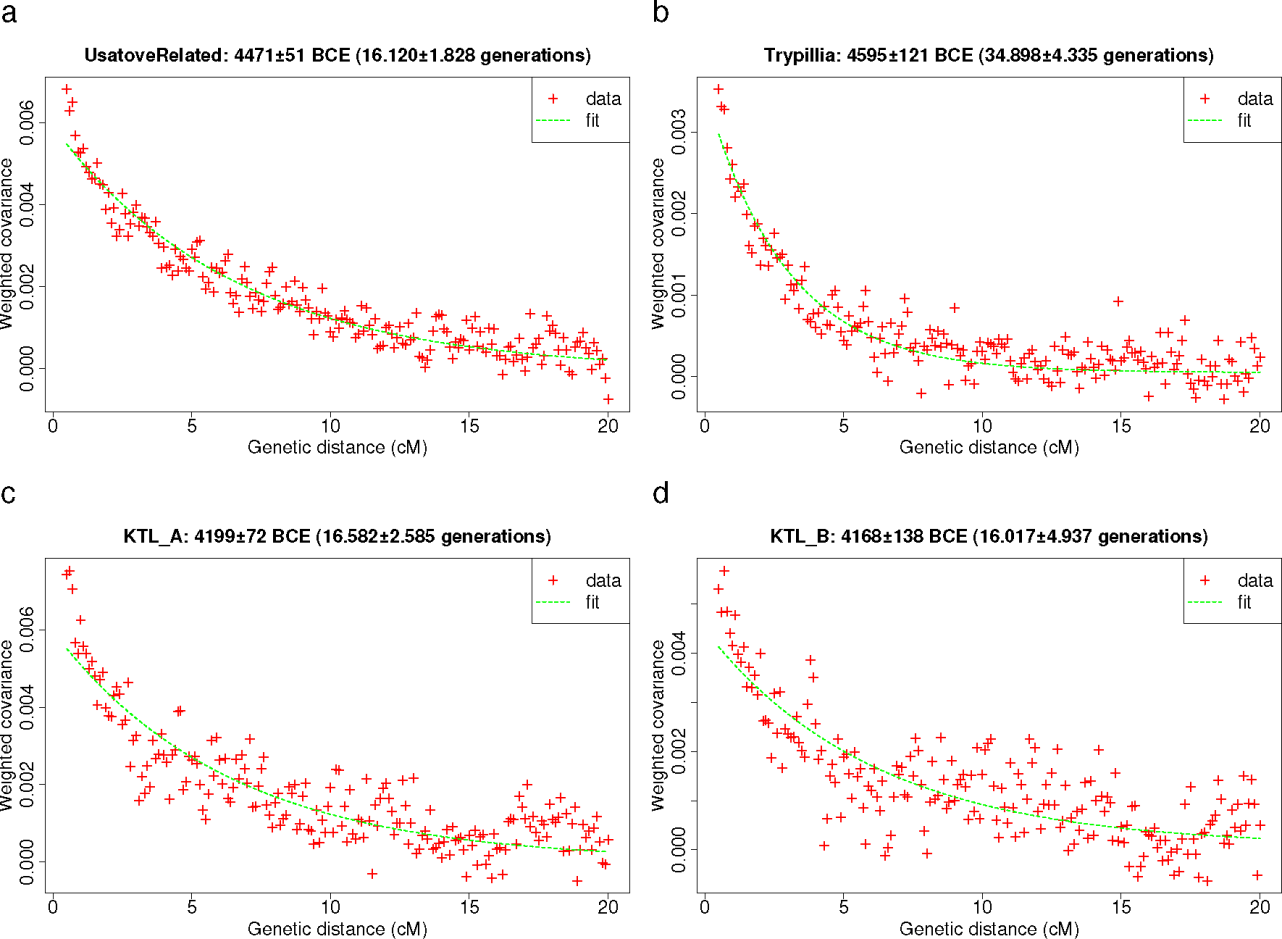
DATES estimates of admixture timing of CLV and European farmer ancestry admixture. (a) Usatove-related individuals from this study and^5^. (b) Trypillians from this study and^17^. Kartal cluster A (c) and B (d) from^5^. We show ±1 standard error, both for the raw admixture date, and the translation to years BCE (assuming fixed generation length of 28 years and not including uncertainty about the age of the admixed individuals; Methods).

Admixture *f*_3_-statistics involving Trypillian individuals from this report and^6,15–17^ show that they are admixed (Extended Data Table 1), with^6^ more hunter-gatherer ancestry than EEF groups such as Yunatsite or LBK, but without a more refined understanding of ancestry sources^36^. A qpAdm model with BPgroup, YUN_CA, and BHG is feasible for 23 of the 24 Trypillians, all including some CLV (Extended Data Table 3 and Supplementary File SI2, p. 107). For these 23 Trypillia individuals their genetic ancestry is, on average, 81% Balkan Eneolithic (such as in YUN_CA), 14% BHG, and 5% CLV-derived BPgroup (Table 1 and Extended Data Table 4). According to DATES^37^, the formative admixture of Trypillia took place 4595±121 BCE (95% C.I. 4832–4358 BCE) (Table 1, Extended Data Table 4, Fig. 3).

Usatove individuals from our study and^5^ are genetically varied and occupy the space in the PCA between the Trypillians and the area where the CLV, Volga, and Dnipro clines intersect. Formal modeling with qpAdm reveals that the Usatove population can only be modeled (p=0.128) as a mixture of ~45% PVgroup (intermediate group on the CLV cline) and ~55% Trypillians (Table 1). A generalized 3-way model in Supplementary File SI2 confirmed that the CLV ancestry in Usatove was not from the lower Volga-centered BPgroup, but had a significant proportion of southern Caucasus Neolithic (Aknashen)-related ancestry.^5^ In contrast to Usatove, the CLV admixture in the Cernavodă I population from Kartal (KTL_A^5^) in the Danube delta is best estimated as BPgroup-derived, with relatively less or no Aknashen-related ancestry (Table 1). We estimate using DATES^37^ that the formative admixture of Usatove took place 4471±51 BCE (95% C.I. 4571–4371 BCE) (Table 1, Fig. 3).

### Yamna ancestry and Caucasus admixture

Following ref.^7^, we define a group we call “Core Yamna,” represented by a genetically homogeneous set of 104 high data quality individuals archaeologically assigned to the Yamna and Afanasievo cultures. In ref.^7^ it is shown that these individuals were from mixed origins around 4000 BCE and formed an ancestral population that expanded from a small founding size around 3750–3350 BCE. Core Yamna is also the largest ancestral source in all individuals carrying Yamna ancestry, who differ only in having additional admixture from local populations the Core Yamna must have encountered during their expansion^7^. In ref.^7^ multiple lines of evidence indicate that the Core Yamna and likely the Yamna itself formed in the Dnipro-Don area of the northeastern NPR region, while not being able to narrow their geographic origin further based on genetic evidence alone.

**Extended Data Figure 1.**
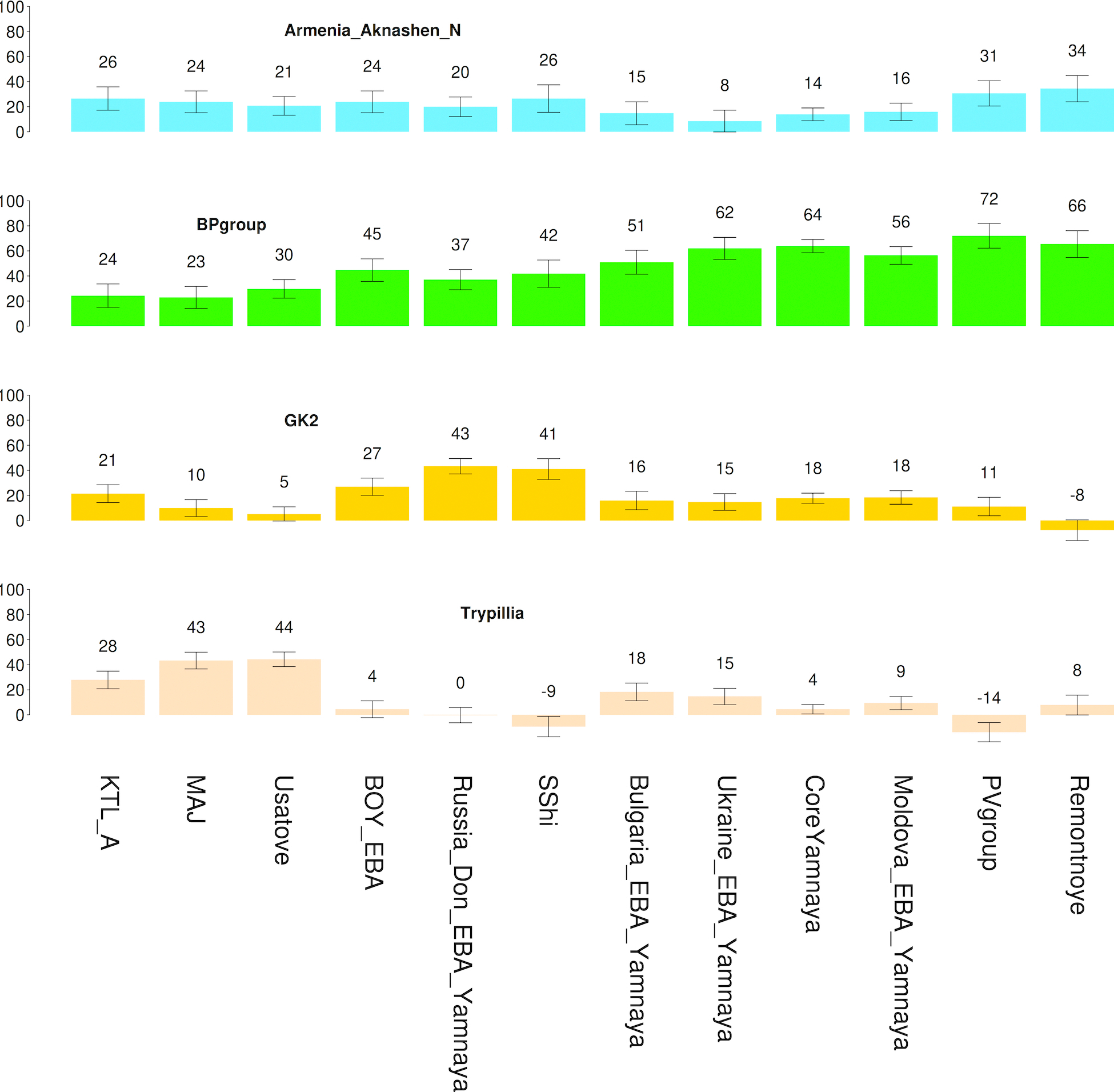
Admixture proportions of 4-source model with Trypillians as the 4th source. Plotted populations fit the model (p>0.05) and we only show populations where the RMSE of standard errors (S.E.) is less than 10% of the point estimate (shown above each bar). For full list of tested populations and alternative choices of modeling see Supplementary File SI2, Appendix I. Sample sizes are in Online Table 4 of ref.^7^.

Ref. ^7^ further showed that the Core Yamna can be modeled as a mixture of CLV and NPR hunter-gatherer groups. When EEF ancestry is forced as an additional source into the Core Yamna beyond CLV and NPR hunter-gatherer sources (Extended Data Fig. 1; Supplementary File SI2, Appendix III) its proportion is not significantly greater than zero (3.2±3.1%) while that of the Caucasus Neolithic is (15.6±4.3%), suggesting Anatolian-related ancestry^10^ in the Core Yamna mediated mainly from Caucasus Neolithic populations (like Aknashen in Armenia^10^) and not from European farmers of Anatolian origin^38^. Further support for this hypothesis comes from the fact that qpAdm models of exclusively CLV+NPR hunter-gatherer ancestry conform with independently derived unsupervised ADMIXTURE estimates of ancestry (Fig. 2b; Supplementary File SI3, p. 141). While EEF ancestry in the Core Yamna is conjectural, it was clearly present in the western Yamna from Bulgaria, Hungary, Moldova, Romania, and Serbia^7^. Yamna admixture became a general ancestry feature in southeast Europe postdating this culture’s expansion, except for the southernmost corner of the Balkan Peninsula in the Aegean^10,39–41^.

Seeking to narrow down the location from which the Yamna originated, we focused on the chronologically earliest Core Yamna individual, Mykhailivka_I32534 (3635–3383 calBCE), from the second (proto-Yamna) layer of the Mykhailivka site in the lower Dnipro Valley in Ukraine, pre-dating the onset of Yamna expansion and forming a clade with it (p=0.684). Mykhailivka_I32534 continues to fit as a clade with Core Yamna when CLV groups are placed on the Right set of qpAdm analysis (Supplementary File SI2, Table SI2. 2). Moreover, when either UNHG or EEF are added as a second source, both are not significant (|Z|<1) and nominally negative, providing no evidence for ancestry other than Core Yamna. Mykhailivka_I32534 thus bridges the temporal gap between the Late Serednii Stih populations the main Yamna expansion that are sampled from south Siberia to eastern Europe and in which any associations with the locale of Yamna formation have been wiped out by thousands of kilometers of distance.

Of the three other early (ca. 3350–3100 BCE) individuals with predominantly Core Yamna ancestry, all from Moldova, individual I20196 from Crasnoe (Moldova_Crasnoe_Eneolithic) was cladal with Core Yamna (p=0.683). Of the other two, I17743 from Mereni (part of Moldova_EBA_Yamnaya) harbored 6.9% EEF admixture (p=0.593) and Zhivotilovka_I17974 from Bursuceni had 18.2% Steppe Maykop admixture (p=0.324; Supplementary File SI2, Table SI2. 9, and p. 106, respectively).

Besides Mykhailivka_I32534, four Yamna individuals from Ukraine, I12168, I20975, I3141_enhanced, and I2105^6^ are cladal with the Core Yamna group in showing no evidence of EEF admixture. Three Yamna Ukraine individuals from the northwest NPR harbor significant such admixture from proximate sources like Bulgaria Eneolithic or Trypillia (Supplementary File SI2, Table SI2. 4 and Table SI2. 13). Thus, the northwest NPR is consistent with being the place where the Yamna first received substantial EEF admixture during their western expansion.

The substantial proportion of EEF ancestry in two Yamna outlier individuals from Moldova is best fitted by Core Yamna + Trypillia or Globular Amphora models (Supplementary File SI2, Table SI2. 10). One of the Yamna individuals from Bulgaria harbored 22.3% YUN_CA-related admixture, while another individual from the same site was cladal with the Core Yamna (Supplementary File SI2, Table SI2. 6 and Table SI2. 7). Thus, the Yamna expansion, beginning in Ukraine and reaching the South Balkans, included both individuals who maintained the Core Yamna genetic profile, as well as those admixing with local farmers and initiating the transmission of Yamna ancestry and, probably, Indo-European languages beyond the steppe.

Two of the Steppe Maykop-shifted individuals in PCA (Fig. 2a), Zhivotilovka_I17974 and Usatove_I20078 from Moldova were formed of the same Yamna+Steppe Maykop-associated admixture process, with I17974 carrying about ~1/3 of the Steppe Maykop-associated ancestry found in I20078 (18.2±6.0% vs. 60.6±6.2%) (Table 1, Supplementary File SI2, Table SI2. 15 and Table SI2. 16). Zhivotilovka_I17973, co-buried with I17974, cannot be well-modeled with any of the sources available to us, but is nearest to the “southern” end of the CLV cline (Maykop of the North Caucasus (p=0.0025) or the Aknashen Neolithic of the South Caucasus (p=0.0047), which is corroborated by the position of I17973 in the PCA (Fig. 2a). In the northeastern NPR, an early Yamna individual Ukraine_EBA_Ozera_I1917^6^ is best modeled as an even mix of Core Yamna and Maykop, providing, like individual I17973, a clear link to the Caucasus. More evidence for this link comes from the Early Bronze Age population from Mayaky^5^, which is discontinuous with the Usatove from the same region but represented a unique combination of 1/5 Maykop ancestry with the remainder best represented by the Yamna of the Lower Don, a population which was itself a mix of Core Yamna and NPR hunter-gatherers^7^.

### Yamna ancestry in the Bronze Age

We find that individuals of the Catacomb archaeological complex, which chronologically partially overlaps and succeeds Yamna in the NPR, continued to harbor Yamna genetic ancestry. The population, labeled “Ukraine_EBA_Catacomb”, including individuals I12840 and I16668 from our dataset, is cladal with the Core Yamna (p=0.075, Supplementary File SI2, Table SI2. 1). Yamna ancestry persisted in the NPR into the second half of the 3^rd^ millennium BCE.

The Catacomb group was succeeded in the NPR by the Babyne (Multi-Cordoned Ware) complex (Supplementary File SI1.4). Feasible models for Babyne ancestry involve Core Yamna, a European farmer source, and considerable hunter-gatherer ancestry (Table 1, Supplementary File SI2, Table SI2. 14). Similarly admixed populations have been described from the Bronze Age of what is today Romania at the sites of Arman (Cârlomănești) and Târgșoru Vechi in Muntenia^10^, indicating that populations of high hunter-gatherer ancestry contributed to some post-Yamna people in the NPR and Southern Carpathians.

## Discussion

This study presents a comprehensive reconstruction of the population dynamics in the North Pontic steppe and forest steppe, leading up to and following the emergence of the Yamna.

We demonstrate that the Neolithic populations of the Dnipro Valley were admixed, roughly with BHG and EHG sources, along with approximately ~7–9% EEF ancestry in UNHG population except for some outliers such as individual I27992 buried in a boat-shaped grave from Yasynyvatka (27±6.0% EEF, this report) and an unadmixed EEF individual I3719 from Deriivka I^6^ (103.5±1.6% EEF). CHG ancestry was also sporadically present at ~7–10%, notably in the Neolithic necropolis at Mariupol. The proximal sources of EEF ancestry in UNHG remain unclear but may have been mediated by BHG migrants in the Dnipro Valley or individuals of EEF genetic background such as individual I3719^6^ that were included in UNHG communities.

The Eneolithic Trypillia population was mainly formed from the sources along the EFHG cline that received limited (~5%) admixture from people with BPgroup CLV ancestry. Usatove was formed from PVgroup CLV people evenly intermixing with Trypillian ancestry.

The evidence from Usatove and Trypillia clarifies the process of the CLV admixture in the NPR in the Eneolithic. Some carriers of Volga-CLV ancestry, as in Giurgiuleşti and Csongrád, advanced across the NPR steppe to the Balkans and Carpathian region largely without admixing with the people they encountered along the way. In contrast, the eastward-bound Trypillian farmers incorporated the Volga-CLV incomers’ ancestry. An intriguing possibility raised by our findings is that Usatove was formed around an outpost in the Danube-Dniester delta area where migrants of Trypillia and early CLV-PVgroup and their economic interests converged. A similar scenario is feasible for the Cernavodă I population of Kartal_A, but with BPgroup-derived carriers of CLV ancestry such as in Giurgiuleşti and Csongrád individuals. Alternatively, Usatove and Kartal A could have formed as a “commonwealth” of co-existing and interdependent cultures in which Trypillia and populations from the Caucasus-Volga both participated. A third scenario places egalitarian Trypillians under the dominance of hierarchically organized patriarchal societies carrying CLV ancestry, extending into the northwestern NPR.

In contrast to Usatove, Serednii Stih carriers of CLV and UNHG-related ancestries in the NPR^7^ lacked appreciable EEF ancestry. The results in ref.^7^ and herein establish the Core Yamna as a late Serednii Stih-derived population that had more CLV ancestry than sampled Serednii Stih individuals but was made of the same CLV and UNHG/GK2 derived components. CLV ancestry comprised ~5% in Trypillia and ~50% of Usatove ancestry, while in Yamna it was ~77%^7^. In Usatove, ~14% of CLV ancestry was southern Caucasus Aknashen-related (Supplementary File SI 2, p.104), while in the Core Yamna the Aknashen-related ancestry was ~21%, thus suggesting that the westward CLV migration may not originate at one single point^7^.

The existence of unadmixed Core Yamna in a wide area from the Altai to Bulgaria is most parsimoniously explained as a consequence of rapid Yamna expansion. The question of whether the remarkable homogeneity of the Core Yamna cluster was a consequence of relative isolation during their formative period or a purposeful avoidance of heterogamy, remains to be answered. In contrast with the formative period, the Yamna taking part in the western expansion carried HG-enriched ancestries related to that seen in Don Yamna, and ancestries from Maykop and Steppe Maykop, while absorbing local EEF ancestry. Was this shift in interpopulation interaction strategy potentially a result of a shifting balance of power which enabled or encouraged broader mating opportunities? The integrative nature of these communities, coupled with their remarkable mobility, plausibly contributed to the Yamna’s success in disseminating their Indo-European language and culture across geographic and population boundaries.

The chronologically earliest (3635–3383 calBCE) individual with the Core Yamna ancestry comes from the Mykhailivka settlement displaying a succession of uninterrupted cultural layers from the late Eneolithic to the EBA^42,43^. In the context of the archaeological evidence, the presented results increase the plausibility of arguments that the lower Dnipro, specifically the area around the Mykhailivka site at a crossroads of ancient steppe “highway” network across the Pontic-Caspian steppe (Supplementary File SI1.2), is a place where Yamna first emerged. The Catacomb and Babyne groups that succeeded Yamna in the NPR continued to harbor Yamna genetic ancestry and displayed a resurgence of hunter-gatherer ancestry towards the Middle Bronze Age. The geographic dispersal of individuals with Babyne genetic ancestry may reflect the high mobility of this group, like that of the Yamna but smaller in scale.

### Waves of CLV expansion

Our analysis suggests a history of three CLV-related partially overlapping waves of migrations into the NPR in the Eneolithic (Table 1). Potentially the earliest BPgroup/PVgroup-related Lower-Volga-end-of-CLV wave started before around 4500 BCE. It was associated with Giurgiuleşti-Csongrád “Ochre Graves” (Fig. SI1.1.1), and left admixture in Trypillia, Usatove, and Kartal_A^5^. A second and more protracted wave carried an intermediate (West Manych-Remontnoye type) part of the CLV cline, and became associated, in its initial pulse, with the formation of Serednii Stih around 4500 BCE, and contributing to the formation of Kartal_B^5^. Otherwise, however, this second wave remained largely contained in the Lower Dnipro Valley region, notably during the steppe “hiatus” in the late 5^th^-early 4^th^ millennium BCE, characterized by a sharp climatic shift towards aridity and cooler temperatures and relative lack of archaeological material^2,44,45^.

The Core Yamna genetic mixture is estimated to have taken place at 4038±48 BCE (95% C.I.: 3944–4132 BCE)^7^, at the height of the steppe hiatus. It is unclear whether this date corresponds to an admixture of populations that happened rapidly, or if it corresponds to a process that unfolded over generations, in which case the date we estimate is an average. Thus, the steppe hiatus may be a reason for the emergence of the Core Yamna ancestry from a nascent Stih-derived proto-Yamna population that was isolated due to the climatic upheaval. In this scenario, the individual from Mykhailivka represents such a proto-Yamna population near the geographical origin of the Core Yamna and sampled from the time where its genetic distinctiveness had already developed.

The third wave of CLV ancestry expansion is that of the Yamna proper, beginning ca. 3300 BCE and lasting into the middle of the following millennium. All three expansion waves spread ancestry from different points on the geographically and genetically diverse CLV cline.

It is remarkable that the three genetic waves of CLV ancestry expansion align, spatially and temporally, with the three waves of Kurgan People proposed by Marija Gimbutas in the 1950s to explain the spread of Indo-European influences and the fall of “Old Europe” (summarized in^1,46^). While Gimbutas envisioned the spread of Kurgan ancestry as a result of a conquest and emphasized *cultural* transformation, our results present evidence of massive *genetic* transformations effected by the spread of CLV ancestry during Waves 1 and 2, and especially, with the spread of the Yamna during Wave 3. Such genetic changes must have involved complex cultural dynamics, in which both conflict and peaceful synthesis may have played a role. Future studies that explore the cultural impact these three expansion waves brought must be informed by the new understanding of the immense genetic impacts that accompanied them.

## Materials and Methods

### Wet laboratory work

In clean rooms where the goal was to protect bones and teeth from contamination by the individuals handling them, we processed human skeletal remains into powder^47^, extracted DNA using a method designed to retain short molecules^47–49^ in some cases using automated liquid handlers^50^, and converted the extracts into double-stranded^51^ and single-stranded^52^ libraries, which were molecularly barcoded with appended dual barcodes (for double-stranded libraries) and dual indices (for both double-stranded and single-stranded libraries) to allow them to be pooled together and then bioinformatically deconvoluted at the analysis stage. We enriched the libraries for sequences overlapping more than 1.2 million SNPs as well as the mitochondrial genome^53^, and then sequenced on NextSeq500, HiSeqX, or NovaSeq instruments, targeting on the order of a hundred thousand sequences for unenriched libraries and on the order of 30 million molecules for enriched ones. Online Table 2 provides information on each library we analyzed.

### Bioinformatic analysis

Following sequencing, we used identifying sequences (barcodes and indices) to demultiplex reads into the appropriate library, before trimming these and sequence adapters. We then used paired-end sequences requiring an overlap of at least 15 base pairs (allowing for 1 mismatch), using a modified version of SeqPrep 1.1 (https://github.com/jstjohn/SeqPrep); at overlapping bases, we selected the highest quality nucleotide to represent the sequence at that position. We aligned sequences to both the human reference genome sequence (hg19) (https://www.internationalgenome.org/category/grch37/) and to the inferred ancestral Reconstructed Sapiens Reference Sequence (RSRS) mitochondrial sequence^54^, using BWA’s samse command^55^. We removed duplicated molecules based on having the same start/stop positions and orientation in their alignment and the same barcodes. The computational pipelines we used are publicly available on GitHub at https://github.com/dReichLab/ADNA-Tools and https://github.com/dReichLab/adna-workflow. We called variants using a ‘pseudohaploid genotyping’ approach, where a single base is randomly selected from a pool of possible bases at each SNP, filtering by a minimum mapping quality of least 10, and base quality of at least 20, trimming each sequence by two base pairs to remove damage artifacts. To assess ancient DNA authenticity, we used both *contamMix-1.0.1051*^56^ to search for heterogeneity in mitochondrial DNA sequences which are expected to be non-variable in uncontaminated individuals, and ANGSD^57^ to search for heterogeneity in X chromosome sequences which should be non-variable in contaminated male individuals^57^. We also evaluated authenticity by searching for an increase in cytosine-to-thymine errors in the final nucleotide (in untrimmed reads) which is expected for genuine ancient DNA^58^ and by computing the ratio of Y chromosome to the sum of X and Y chromosome sequences which is expected to be very low for females and to have a very much higher value for males. We determined a consensus sequence for mitochondrial DNA using *bcftools* (https://github.com/samtools/bcftools) and *SAMtools*^59^ requiring a minimum of 2-fold coverage to call the nucleotide and a majority rule to determine its value. We used *HaploGrep2* to determine the mitochondrial haplogroups based on this consensus sequence, leveraging the phylotree database (mtDNA tree build 17)^60^.

### Population genetic analysis

We performed principal components in smartpca^23^ using lsqproject: YES and newshrink: YES parameters and the populations OberkasselCluster (set of trans-Alpine WHG individuals identified in^27^), Russia_Firsovo_N, Iran_HajjiFiruz_C^9^, Iran_C_SehGabi^61^, Iran_C_TepeHissar^62^, Israel_C^63^, and Germany_EN_LBK^3,12,29,64^ to form the axes (Fig. 2).

We used qpWave and qpAdm^3,65^ to test whether *n*+1 “left” populations (one Test and *n* sources) are consistent with descending from *n* ancestral sources with respect to a set of Right populations as in^7^ (OldAfrica^66–68^, Russia_AfontovaGora3^69^, CHG^70^, Iran_GanjDareh_N^61^, Italy_Villabruna^69^, Russia_Sidelkino.SG^8^, Turkey_N^29^).

We performed a subset of unsupervised ADMIXTURE analysis^71^ using a new data processing pipeline focusing on “summary individuals” that prevents the formation of population-specific ancestry components. This provides a complementary approach to qpAdm allowing us to obtain insights into the ancestry of diverse population from the NPR and neighboring regions (Fig. 2b).

We dated the admixture time of Usatove-related populations (individuals from Mayaky presented in this report and from Mayaky (MAJ) and Usatove-Velykyj Kuyalnik (USV) from^5^) and Trypillians, using DATES^37^ to infer the number of generations prior to the ^14^C date of the studied individuals, and converted to a calendar date assuming 28 years per generation^72^. Uncertainty ranges reflect the standard error computed by DATES and not the uncertainty of the average ^14^C date of admixed individuals.

## Extended Data

**Extended Data Figure 2.**
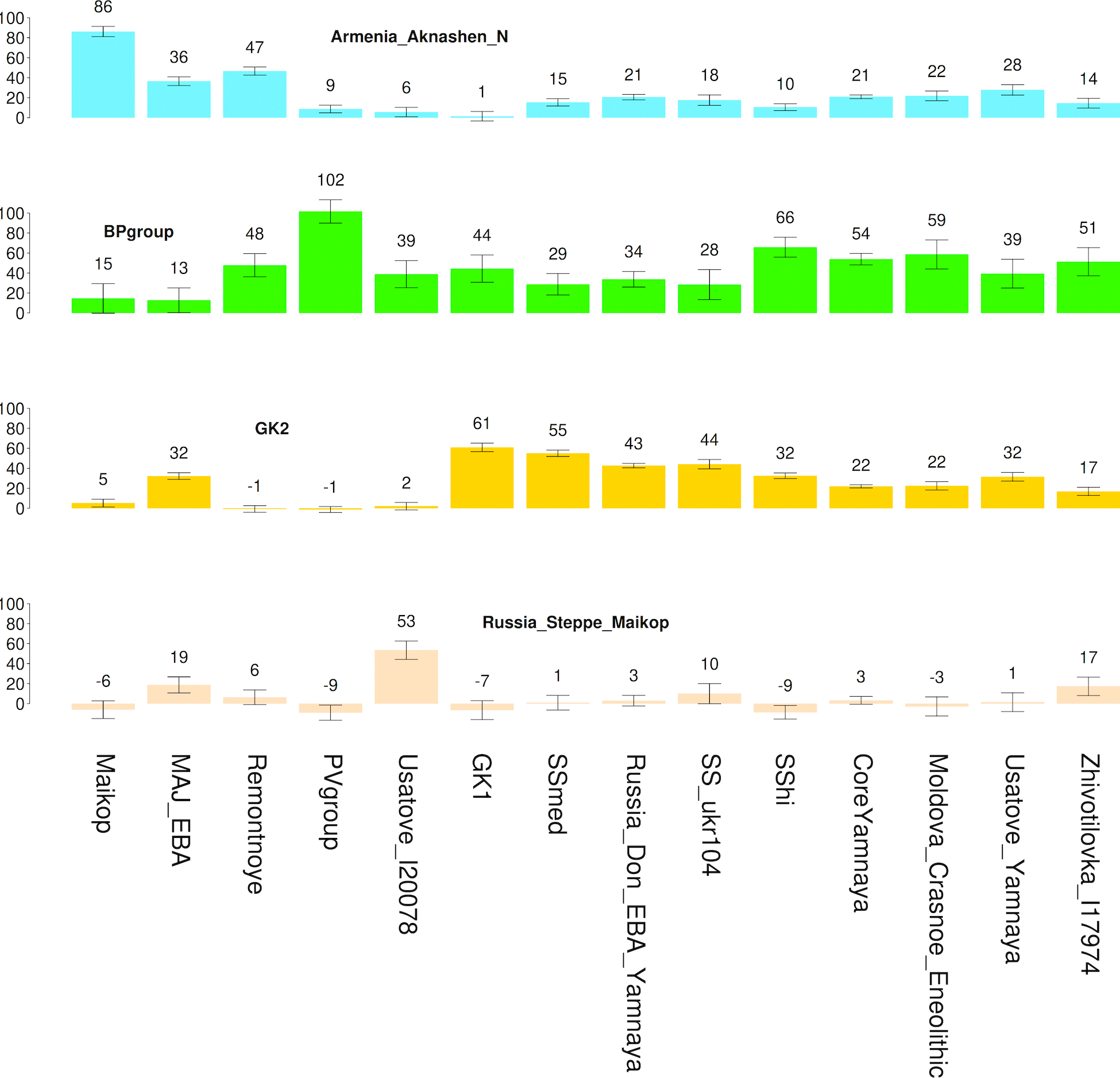
Admixture proportions of 4-source model with Steppe Maykop as the 4th source. Plotted populations fit the model (p>0.05) and we only show populations where the RMSE of standard errors (S.E.) is less than 10% of the point estimate (shown above each bar). For full list of tested populations including sample sizes and alternative choices of modeling see Supplementary File SI2, Appendix II. Sample sizes are in Online Table 4 of ref.^7^.

**Extended Data Table 1.** Statistics of the form *f*_3_(Source_1_, Source_2_; Test). The statistic with the lowest Z-score of all the considered pairs is shown. P-values from *qpAdm* are based on a Hotelling T2 test.

Test	Source1	Source2	*f*_3_(Source_1_, Source_2_; Test)	Z-score
BOY_EBA	TTK	Trypillia	−0.016097	−7.0
Bulgaria_EBA_Yamna	Russia_Karelia	YUN_CA	−0.011836	−9.2
CoreYamna	Maykop	Russia_Karelia	−0.006310	−13.6
GlobularAmphora	Serbia_IronGates_Mesolithic	YUN_CA	−0.005914	−8.2
KTL_A	Russia_Karelia	YUN_CA	−0.014186	−17.5
KTL_B	Russia_Karelia	YUN_CA	−0.009922	−9.1
MAJ	Russia_Karelia	YUN_CA	−0.009438	−12.7
MAJ_EBA	GlobularAmphora	TTK	0.004403	1.6
Moldova_EBA_Yamna	Maykop	Russia_Karelia	−0.007198	−10.0
PIE_CA	Serbia_IronGates_Mesolithic	YUN_CA	−0.002351	−6.9
PTK_CA	TTK	YUN_CA	0.001444	0.3
Romania_LN_Eneol	Armenia_Aknashen_N	Serbia_IronGates_Mesolithic	0.002525	0.5
SShi	Armenia_Aknashen_N	Russia_Karelia	−0.010140	−6.3
SSmed	BPgroup	Serbia_IronGates_Mesolithic	−0.012501	−10.6
Trypillia	Serbia_IronGates_Mesolithic	YUN_CA	−0.008350	−23.8
Ukraine_Deriivka_Mes	Russia_Karelia	Serbia_IronGates_Mesolithic	−0.003244	−1.3
Ukraine_EBA_Catacomb	Armenia_Aknashen_N	Russia_Karelia	−0.022783	−1.7
Ukraine_EBA_Yamna	Maykop	Russia_Karelia	−0.009610	−8.1
Ukraine_MBA_MultiCordonedWare_Babyne	GK2	YUN_CA	−0.017018	−2.5
Ukraine_N	Russia_Karelia	Serbia_IronGates_Mesolithic	−0.007871	−17.2
Ukraine_Vasilevka	Serbia_IronGates_Mesolithic	TTK	−0.005716	−3.0
Usatove	Russia_Karelia	YUN_CA	−0.008941	−10.1
USV	Russia_Karelia	YUN_CA	−0.011918	−12.0
VAR_CA	Serbia_IronGates_Mesolithic	YUN_CA	−0.003861	−9.3
YUN_EBA	Serbia_IronGates_Mesolithic	YUN_CA	−0.001677	−2.6

**Extended Data Table 2.** Ancestry of UNHG individuals. EHG=Lebyazhinka_HG; BHG=Serbia_IronGates_Mesolithic; CHG=Caucasus_Hunter_Gatherer. We include close relatives and outliers. P-values from *qpAdm* are based on a Hotelling T2 tests

Modeling Ukraine Neolithic individuals with LBK as a source									
		Proportions			Std. errors				
Individual	P-value	LBK	EHG	BHG	LBK	EHG	BHG	Z-score of LBK	Population Label
I5878_enhanced	1.22E-01	9.7%	58.3%	32.0%	2.4%	3.7%	4.1%	**4.0**	Ukraine_N_father.or.son.I5883
I5886_enhanced	5.56E-04	7.4%	58.1%	34.4%	1.9%	3.3%	3.5%	**3.9**	Ukraine_N
I5886_published	2.23E-02	9.6%	57.6%	32.7%	3.0%	4.6%	5.0%	**3.2**	Ukraine_N
I5892	3.59E-01	3.2%	57.2%	39.6%	2.7%	4.1%	4.6%	1.2	Ukraine_N
I5870	6.63E-01	7.8%	56.2%	36.0%	2.3%	3.7%	3.9%	**3.4**	Ukraine_N
I3716_published	2.61E-01	8.7%	56.1%	35.2%	2.7%	4.8%	5.0%	**3.2**	Ukraine_N
I31730	3.72E-03	6.5%	54.9%	38.5%	2.3%	3.9%	4.1%	**2.8**	Ukraine_N
I1736	8.22E-01	6.5%	54.8%	38.7%	1.9%	3.2%	3.4%	**3.4**	Ukraine_N
I27992	3.95E-01	27.0%	54.5%	18.5%	6.0%	9.5%	10.6%	**4.5**	Ukraine_N_I27992
I3720	1.00E-01	5.8%	53.8%	40.4%	3.6%	5.3%	5.7%	1.6	Ukraine_N
I5872_published	6.42E-01	10.1%	53.2%	36.7%	3.0%	4.3%	4.8%	**3.4**	Ukraine_N
I3717	6.08E-01	9.4%	53.1%	37.5%	2.0%	3.3%	3.5%	**4.7**	Ukraine_N
I6133_published	2.55E-01	1.9%	52.5%	45.7%	3.8%	6.0%	6.7%	0.5	Ukraine_N
I5957_published	8.41E-01	3.7%	52.5%	43.8%	3.0%	5.0%	5.5%	1.2	Ukraine_N
I5869	5.99E-01	10.4%	51.9%	37.7%	2.7%	4.5%	5.0%	**3.9**	Ukraine_N_1d.rel.I5870
I3713_published	9.28E-02	5.8%	51.4%	42.8%	3.4%	5.5%	6.0%	1.7	Ukraine_N
I1732	3.43E-01	3.5%	51.4%	45.1%	1.8%	3.1%	3.3%	1.9	Ukraine_N
I1378_enhanced	5.91E-02	4.0%	51.4%	44.6%	2.2%	3.8%	4.0%	1.8	Ukraine_N_son.I1732
I3715	3.50E-01	5.1%	51.1%	43.8%	1.8%	3.5%	3.7%	**2.8**	Ukraine_N
I5888_enhanced	2.30E-02	6.3%	50.9%	42.9%	1.8%	3.0%	3.3%	**3.5**	Ukraine_N_father.or.son.I5875
I27982	1.80E-03	11.9%	50.9%	37.2%	4.8%	7.5%	8.0%	**2.5**	Ukraine_N
I27994	2.39E-01	7.6%	50.8%	41.6%	2.0%	3.1%	3.3%	**3.8**	Ukraine_N
I5883	6.50E-01	7.2%	50.4%	42.4%	2.5%	3.9%	4.3%	**2.9**	Ukraine_N
I4112_enhanced	3.14E-02	6.4%	50.2%	43.4%	2.1%	3.7%	3.7%	**3.0**	Ukraine_N_dup.I4112
I5889_published	3.31E-01	10.2%	50.0%	39.8%	3.6%	5.4%	5.6%	**2.8**	Ukraine_N
I3721	5.35E-01	15.2%	49.6%	35.3%	3.1%	5.0%	5.2%	**4.9**	Ukraine_N
I5893_enhanced	5.52E-01	4.0%	48.9%	47.1%	2.3%	3.5%	3.8%	1.7	Ukraine_N_1d.rel.I5881
I3714	4.43E-01	8.1%	48.8%	43.1%	2.6%	4.0%	4.5%	**3.1**	Ukraine_N
I5879	9.33E-01	5.0%	48.7%	46.3%	2.5%	4.2%	4.4%	2.0	Ukraine_N_father.or.son.I3718
I5891_enhanced	2.98E-01	2.6%	48.3%	49.0%	2.9%	4.4%	5.0%	0.9	Ukraine_N_1d.rel.I4114
I3712_published	9.16E-01	14.3%	47.7%	38.1%	3.4%	5.2%	5.7%	**4.2**	Ukraine_N
I5875	2.34E-01	7.0%	46.8%	46.2%	1.9%	3.3%	3.5%	**3.7**	Ukraine_N
I1734	8.96E-01	7.2%	46.8%	46.0%	1.9%	3.0%	3.2%	**3.8**	Ukraine_N
I4114	7.20E-01	7.3%	46.0%	46.7%	1.9%	2.9%	3.1%	**3.8**	Ukraine_N
I5873_published	7.85E-01	12.3%	45.9%	41.8%	4.9%	7.9%	8.2%	**2.5**	Ukraine_N
I5881_published	8.66E-01	5.6%	45.8%	48.6%	3.0%	5.1%	5.4%	1.9	Ukraine_N
I4112_published	3.76E-01	7.6%	45.6%	46.8%	3.5%	5.6%	5.7%	**2.2**	Ukraine_N
I4111	2.08E-02	8.6%	45.1%	46.3%	1.8%	3.0%	3.3%	**4.8**	Ukraine_N
I1738	2.69E-03	5.8%	44.1%	50.2%	1.8%	3.2%	3.4%	**3.2**	Ukraine_N
I5890	2.39E-01	7.9%	43.7%	48.4%	2.0%	3.4%	3.8%	**4.0**	Ukraine_N
I5881_enhanced	4.98E-02	8.2%	43.2%	48.6%	1.8%	3.1%	3.3%	**4.6**	Ukraine_N
I3718	6.34E-01	8.0%	42.9%	49.1%	1.9%	3.1%	3.4%	**4.2**	Ukraine_N
I27990	2.40E-01	10.3%	39.5%	50.2%	2.7%	4.7%	4.9%	**3.8**	Ukraine_N
I5868_published	7.67E-01	12.5%	38.1%	49.4%	4.8%	7.8%	8.5%	**2.6**	Ukraine_N
I3719_enhanced	9.27E-01	103.5%	4.2%	−7.6%	1.6%	2.2%	2.4%	**64.7**	Ukraine_N_Deriivka_13719
**Modeling Ukraine Neolithic individuals with CHG as a source**									

Individual	P-value with LBK	P-value with CHG	CHG	EHG	BHG	CHG	EHG	BHG
I5886_enhanced	5.60E-04	2.20E-03	8.50%	50.80%	40.70%	2.50%	3.70%	2.90%
I5886_published	2.20E-02	4.70E-02	13.10%	47.60%	39.40%	3.90%	6.00%	4.70%
I31730	3.70E-03	9.30E-02	7.40%	48.30%	44.40%	2.80%	4.50%	3.70%
I5888_enhanced	2.30E-02	1.10E-02	6.50%	45.60%	47.90%	2.20%	3.70%	3.10%
I27982	1.80E-03	1.00E-03	16.70%	38.20%	45.10%	6.50%	9.90%	7.40%
I4112_enhanced	3.10E-02	2.00E-02	7.10%	44.40%	48.50%	2.80%	4.30%	3.30%
I4111	2.10E-02	1.80E-03	7.90%	39.80%	52.40%	2.30%	3.60%	3.10%
I1738	2.70E-03	2.30E-01	10.20%	37.10%	52.70%	2.20%	3.40%	3.10%
I5881_enhanced	5.00E-02	3.90E-04	6.90%	39.60%	53.50%	2.30%	3.50%	3.20%

**Extended Data Table 3.** By-individual modeling of Trypillians. P-values from *qpAdm* are based on Ha otelling T2 test.

		Proportions			Std. errors			
Trypillian individual	P-value	BPgroup	Iron Gates	YUN_CA	BPgroup	Iron Gates	YUN_CA	Z-scoire of BPgroup
I2111_enhanced	0.6637	−5.1%	20.4%	84.7%	3.7%	3.4%	3.3%	−1.4
VERT117_wNonUDG.SG	0.0863	−3.9%	14.9%	89.0%	2.6%	2.5%	2.3%	−1.5
I7586	0.3971	−1.4%	14.3%	87.1%	2.3%	2.2%	2.1%	−0.6
VERT029_wNonUDG.SG	0.3637	0.6%	13.5%	86.0%	2.3%	2.2%	2.0%	0.3
VERT035_wNonUDG.SG	0.0279	0.9%	17.8%	81.4%	2.4%	2.1%	2.1%	0.4
VERT028_wNonUDG.SG	0.1660	1.0%	15.8%	83.1%	2.5%	2.2%	2.1%	0.4
VERT100B_wNonUDG.SG	0.2974	1.7%	15.2%	83.0%	2.3%	2.1%	2.1%	0.7
I1929	0.5967	1.8%	14.7%	83.5%	6.6%	5.7%	5.2%	0.3
I13064	0.1473	3.0%	14.9%	82.1%	2.2%	2.1%	1.9%	1.4
VERT030_wNonUDG.SG	0.1079	3.2%	12.7%	84.1%	2.4%	2.2%	2.0%	1.3
VERT115_wNonUDG.SG	0.3177	3.4%	14.2%	82.3%	3.0%	2.7%	2.6%	1.1
VERT106C_wNonUDG.SG	0.9459	3.5%	15.5%	81.1%	3.1%	2.7%	2.7%	1.1
VERT015_wNonUDG.SG	0.0019	3.8%	13.5%	82.7%	2.3%	2.1%	2.0%	1.7
VERT033_wNonUDG.SG	0.0606	3.9%	12.2%	83.9%	2.6%	2.3%	2.2%	1.5
VERT107_wNonUDG.SG	0.0914	3.9%	17.4%	78.7%	2.3%	2.2%	2.0%	1.7
I7584	0.3849	5.1%	12.6%	82.2%	5.0%	4.4%	4.1%	1.0
I2110	0.4913	5.3%	13.5%	81.1%	2.4%	2.3%	2.2%	**2.2**
VERT105B_wNonUDG.SG	0.0105	5.4%	12.3%	82.3%	2.5%	2.3%	2.1%	**2.2**
VERT111_wNonUDG.SG	0.0004	5.5%	10.2%	84.3%	2.7%	2.5%	2.3%	**2.0**
I1926_enhanced	0.3223	5.9%	16.0%	78.1%	2.3%	2.3%	2.1%	**2.6**
VERT104B_wNonUDG.SG	0.2516	5.9%	12.0%	82.2%	2.4%	2.0%	2.0%	**2.5**
I3151_enhanced	0.4581	6.1%	14.8%	79.1%	3.9%	3.6%	3.3%	1.6
VERT118_wNonUDG.SG	0.3989	7.1%	12.2%	80.7%	2.6%	2.3%	2.2%	**2.7**
I7920	0.1891	7.5%	13.5%	79.0%	2.4%	2.0%	2.1%	**3.1**
VERT103B_wNonUDG.SG	0.0252	8.2%	10.6%	81.2%	2.6%	2.2%	2.2%	**3.2**
I7923	0.7187	9.2%	15.3%	75.5%	5.6%	5.1%	4.2%	1.6
VERT031_wNonUDG.SG	0.5192	13.5%	11.5%	75.0%	2.5%	2.2%	2.2%	**5.4**
I20069	0.0926	25.8%	9.9%	64.3%	2.4%	2.2%	2.1%	**10.8**

**Extended Data Table 4.** Individuals in the North Pontic Region 4500-2500 BCE are well described as a result of three expansion waves: two waves of Caucasus-Lower Volga (CLV) cline expansion and a wave of Yamna expansion, largely succeeding each other in time (expanded version of Table 1).

Genetic ID, Arch. ID, Date	Pop. Source(s)	P-value	Comment

**Wave 1: Early pioneers from the genetically northern end of the Caucasus-Lower Volga (CLV) cline & their descendants**			
I20072: Giurgiuleşti Burial 6 (3), 4330–4058 caIBCE	BPgroup^a^	0.896	Eneolithlc Individual from Moldova who was a descendant of Lower-Volga North Caucasus Eneollthic people (the low-EHG end (BPgroup endpoint, Fig. 2a) of the Volga Cline at a junction with the Caucasus-Lower Volga (CLV) cline), an example of long-range migration across the NPR
I5124: Csongrád Burial 1,4331–4073 caIBCE	87% BPgroup and 13% LebyazhinkaJHG	0.116	Eneolithic Individual from Hungary with ancestry from the BPgroup end of the Volga cline, similar to a subset of Khvalynsk individuals, an example of long-range migration across the NPR
Tryplllia genetic ancestry forming 4832–4358 BCE	Mean: 5% BPgroup, 14% BHG, 81% YUN_CA^b^	7e-6	Heterogeneous Eneolithic Trypillia population from Ukraine and Moldova formed on the European farmer-hunter-gatherer cline and included CLV with admixture from Usatove-related groups in the second half of the 4^th^ millennium BCE. The given model fits 23 of 28 Trypillian individuals but not the Trypillians as a whole.
Usatove (Mayaky), genetic ancestry forming 4571–4371 BCE	45% PVgroup^c^ and 55% Trypillians	0.128	Eneolithic Usatove from Mayaky in Ukraine were an even mix of an intermediate PVgroup population on the CLV cline or, alternatively a mix of BPgroup and Caucasus Neolithic (Aknashen), and Trypillians
Usatove (Mayaky), MAJ	44% PVgroup and 56% Trypillians	0.231	Another group of Usatove individuals from Mayaky^5^
Usatove (Usatove-Velykyj Kuyalnik), USV	48% PVgroup and 52% Tryplllians	0.083	Usatove individuals from Usatove-Velykyj Kuyalnik in Ukraine^5^
Cernavodă I, KTL_A, genetic ancestry forming 4340–4058 BCE	54% BPgroup and 46% Trypillians	0.618	Eneolithic Cernavodă I population from Kartal in Ukraine (cluster A^5^) an even mix of BPgroup and European farmers. This mix is similar to Usatove and related populations, but without the Caucasus Neolithic ancestry evident in Usatove; the mixture that formed KTL A also occurred significantly later on average.
**Wave 2: Migration from the genetically intermediate part of the CLV cline and establishment of Core Yamna ancestry**			
Serednii Stih, genetic ancestry forming ca. 4400 BCE^35^ (SShi, SSmed, SSIo subsets)	CLV ancestry: 13–17% Aknashen Neolithic and 8–56% BPgroup; Dnipro-Don ancestry: 31–56% GK2 ancestry 26% Remontnoye^d^ and 74% SShi subset of Serednil Stih	0.102–0.851	Eneolithic Stih Individuals from Ukraine were genetically heterogeneous but formed a cline between CLV people (themselves a mix of Caucasus Neolithic (Aknashen-related) and North Caucasus-Lower Volga Eneolithic (BPgroup-related) people) with Dnipro-Don people (Ukraine Neolithic hunter-gatherer-related)^7^
Core Yamna, genetic ancestry forming 4132–3944 BCE	CLV ancestry: 21% Aknashen Neolithic and 57% BPgroup; Dnipro-Don ancestry: 23% GK2 ancestry	0.675 0.934	Early Bronze Age (EBA) Core Yamna cluster includes individuals across 5000 km from central Siberia to southeastern-central Europe and was formed on the basis of admixture of CLV people with Dnipro-Don people. Their emergence likely occurred in the North Pontic Region as descendants of a late Stih population who are unique in possessing this combination of ancestries^7^
Cernavodă I, KTL_B, genetic ancestry forming 4438–3898 BCE	27% Remontnoye and 73% European farmers (YUN_CA+Globular Amphora)	0.294	Eneolithic Cernavodă I population from Kartal cluster B in Ukraine cluster B^5^ had much less CLV ancestry than the cluster A individuals. This ancestry was also not from the Lower Volga (BPgroup) end of the CLV cline, but rather from a population like Maykop or Remontnoye
I1428: Riltsi Kurgan 264, Burial 5, 3360–2890 caIBCE	50% Remontnoye and 50% YUN_CA	0.558	Eneolithic individual from Bulgaria who was a mixture of CLV people (PVgroup or Remontnoye) and European farmers such as YUN_CA
I17973: Bursuceni Kurgan 1, Burial 21, Skeleton 1,3354–3103 caIBCE	Maykop (?)	0.0025	Late Eneolithic Individual from the same burial as 117974 is related to populations from the Caucasus (Fig. 2) but with some unspecified ancestry

**Wave 3: Yamna expansion**			
**Core Yamna**			
I32534: Mykhailivka 1, Square VI, 3635–3383 caIBCE	Core Yamna	0.684	Eneolithic individual from Ukraine is the earliest ^14^C-dated individual with Core Yamna ancestry in the NPR
I20196: Crasnoe Kurgan 9, Burial 9, Skeleton 2, 3352–3101 caIBCE	Core Yamna	0.683	Eneolithic Individual from Moldova was genetically a Yamna descendant
I12229: Mayaky, Kurgan 1, Burial 9, 3088–2911 caIBCE	Core Yamna	0.178	EBA Individual from the Usatove site at Mayaky is discontinuous with the earlier Usatove people from Mayaky and was genetically a Yamna descendant
I20079: Taraclla II, Kurgan 10, Burial 2, 2571–2355 caIBCE	Core Yamna	0.864	Early-Middle Bronze Age (EMBA) Individual from Zhyvotylivka-Volchans'k/lll-C (ZV/lll-C) type burial from Moldova was genetically a Yamna descendant
Catacomb Archaeological Complex I12840: Dubynove, Kurgan 1, Burial 10, 2453–2148 caIBCE I16668: Revova, Kurgan 3, Burial 10, 2800–2000 BCE	Core Yamna	0.075	EMBA Catacomb individuals from Ukraine (MJ-09 from Mamaj Gora^37^,112840 and 116668, this study) were Yamna descendants
**Core Yamna + European Farmer-Hunter-Gatherer descendants**			
I1456: Durankulak, Kurgan F, burial 15 (main burial), 3500–3000 BCE	45% Core Yamna and 55% Globular Amphora	0.099	Eneolithic Individual from Bulgaria was a Yamna+Globular Amphora descendant representing a similar mix (but in different proportions) to the Corded Ware
Bulgaria Yamna, 3300–2500 BCE	Core Yamna and 0–22% YUN_CA	-	
Bulgaria Yamna, Boyanovo subset, 3300–2500 BCE^5^ Moldova Yamna, 3300–2500 BCE	94% Core Yamna and 6% YUN_CA Core Yamna and 0–16% YUN_CA	0.211	EBA Yamna individuals from Bulgaria, Moldova, and Ukraine (^7^ and herein) included unadmixed Core Yamna as well as others with European farmer ancestry. This admixture started no later than the date of individual 117743 (Mereni II) from Moldova (3358–3100 BCE) which already had 6.9% such ancestry.
Ukraine Yamna, 3300–2500 BCE	Core Yamna and 0–8% YUN_CA		
I17747: Tiraspol Kurgan 3, Burial 15, 2865–2576 caIBCE	61% Core Yamna and 39% Trypillia	0.523	Late EBA Yamna individual from Moldova had more farmer ancestry than other Yamna from the region
I20076: Ocnlta Kurgan 1, Burial 3, 2906–2702 caIBCE	88% Core Yamna and 12% Globular Amphora	0.180	Individual from an EBA Yamna burial in Moldova with Globular Amphora-style pot is analyzed separately but is of mostly Yamna descent
I4110,15882,15884: Deriivka I cemetery, 3500–2700 BCE^6^	36–46% Core Yamna, 23–44% Balkan Hunter Gatherer (BHG), 15–32% Trypillia	0.179–0.889	Three Eneolithic-EBA Individuals from Ukraine had some Yamna ancestry but substantial (BHG) ancestry represented by Serbia Iron Gates hunter-gatherers
I13071: Bll’shivtsi Individual 1,2201–2032 caIBCE	72% Core Yamna and 28% YUN_CA	0.458	Middle Bronze Age (MBA) individual from a catacomb burial in western Ukraine with 2/3–1/3 Core Yamna- European Farmer ancestry, the source of the farmer ancestry being unclear.
I12234: Liubasha Kurgan Burial 3, 1499–1127 caIBCE I7925: Liubasha Kurgan, Burial 9, 2119–1624 caIBCE 112235: Liubasha Kurgan, Burial 11, 1686–1311 caIBCE 116674: Liubasha kurgan Burial 15, 2434–1943 caIBCE 112231: Sychavka Kurgan, Burial 18, 2118–1565 caIBCE	77% Core Yamna and 15% Globular Amphora and 8% UNHG	0.148	These five MBA individuals of Multi-Cordoned Ware/Babyne archaeological circle from Ukraine were mostly of Yamna descent but mixed with a population of even more hunter-gatherer ancestry than the Globular Amphora
**Core Yamna + Dnipro-Don Hunter Gatherer descendants**			
Don Yamna, 3200–2600 BCE	40% Core Yamna and 60% SSmed	0.237	Yamna from the lower Don were formed on the basis of the same elements as the Core Yamna and Serednii Stih but with more UNHG Ukraine Neolithic ancestry^7^
**Core Yamna + Steppe Mavkop descendants**			
I20078: Taraclia II Kurgan 2, Burial 14, 3340–3034 caIBCE	39% Core Yamna, 61% Steppe Maykop	0.432	Late Eneolithic Individual from a ZV/III-C type burial from Moldova was mix of Yamna with Steppe Maykop
I17974: Bursuceni Kurgan 1 Burial 21, Sk. 2, 3334–3030 caIBCE	82% Core Yamna, 18% Steppe Maykop	0.324	Late Eneolithic Individual from a ZV/III-C type burial from Moldova, another mix of Yamna & Steppe Maykop
**Yamna + Mavkop descendants**			
I1917: Ozera Kurgan 18 Burial 14, 3096–2913 caIBCE	50% Core Yamna and 50% Maykop	0.345	This individual from Ukraine^6^ displaying mixed Maykop-Yamna burial traditions had half Maykop ancestry
Mayaky Yamna, 2900–2500 BCE	81% Don Yamna and 19% Maykop	0.424	Three EBA Yamna individuals from Kurgan 1 and a ground burial at the Usatove site of Mayaky^5^ were a mixture of Don Yamna (itself a mixture of Core Yamna and Dnipro-Don hunter-gatherers) and Maykop

Notes: For admixture dates we give one standard error, and a 95% confidence interval. For direct dates on bones analyzed for DNA, we indicate the 95% calibrated confidence with suffix “calBCE”; all other dates are archaeologically estimated ranges.

a BPgroup is a homogeneous group from the Lower Volga-North Caucasus Eneolithic (CLV) at the bend between CLV and Volga (EHG-rich) clines (Fig. 2a) from Berezhnovka and Progress 2 that carries CHG, EHG, and Siberian/Central Asian Neolithic-related ancestries^7^.

b Balkan farmers of Gumelnița/Karanovo from Yunatsite in Bulgaria.

c PVgroup BP-related group from the CLV cline with more Aknashen (south Caucasus) ancestry than BPgroup, from Berezhnovka & Vonjucka^7^.

d Remontnoye represents a population composed of a southern ancestry represented by either the Aknashen Neolithic of Armenia or the Bronze Age Maykop, and a northern ancestry from the low-EHG end of the Volga Cline such as the BPgroup^7^.

## Supplementary Material

Supplement Information

Online Tables

## Figures and Tables

**Fig. 1:**
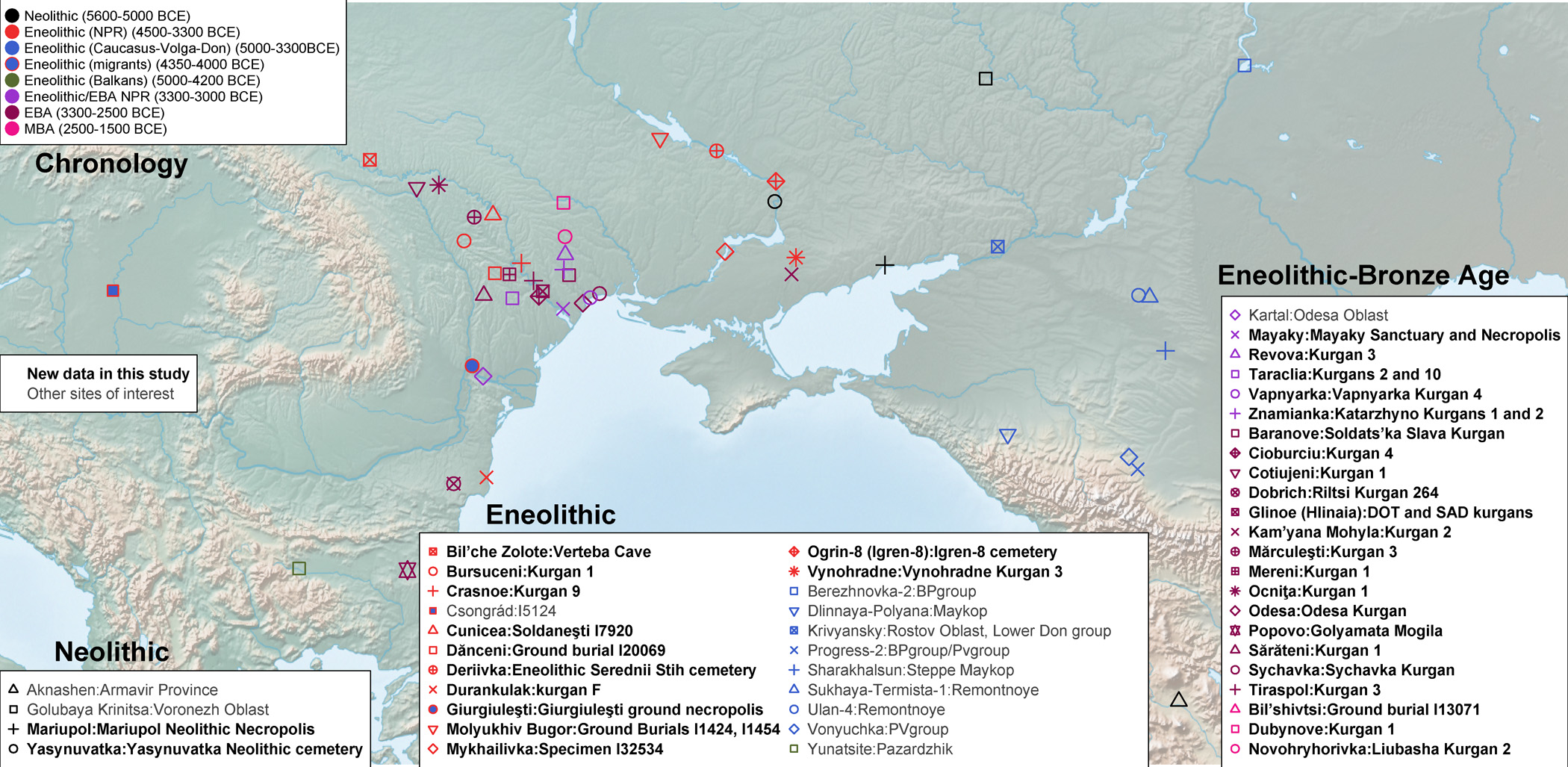
Map of sampling locations including newly generated data and key context populations. The map was drawn using public domain Natural Earth data with the rnaturalearth package in R^24^.

**Table 1.** Genetic profiles of individuals in the North Pontic Region 4500–2500 BCE are well described as a result of three expansion waves: two waves of Caucasus-Lower Volga (CLV) cline expansion and a wave of Yamna expansion, largely succeeding each other in time (Extended Data Table 4 is a more detailed version).

Individual or group identifier and date	Model fit to data	

**Wave 1: Early pioneers from the genetically northern end of the Caucasus-Lower Volga (CLV) cline and descendants**		
I20072: Giurgiuleşti Burial 6, 4330–4058 calBCE	BPgroup^a^	
I5124: Csongrád Burial 1, 4331–4073 calBCE	87% BPgroup / 13% Lebyazhinka_HG	
Trypillia formation by mixture 4832–4358 BCE	5% BPgroup / 14% BHG / 81% YUN_CA^b^	
Usatove, formation by mixture 4571–4371 BCE	44–48% PVgroup^c^ / 52–55% Trypillians	
Cernavodă I, KTL_A, formation by mixture 4340–4058 BCE	54% BPgroup / 46% Trypillians	

**Wave 2: Migration from a genetically intermediate part of the CLV cline and establishment of Core Yamna ancestry**		
Serednii Stih, formation by mixture ~4400 BCE^35^	13–17% Aknashen N. / 8–56% BPgroup / 31–56% Dnipro-Don	
Core Yamna, formation by mixture 4132–3944 BCE	26% Remontnoye^d^ / 74% SShi of Serednii Stih	
Cernavodă I, KTL_B, formation by mixture 4438–3898 BCE	27% Remontnoye / 73% European farmers	
I1428: Riltsi Kurgan 264, Burial 5, 3360–2890 calBCE	50% Remontnoye / 50% YUN_CA	
I17973: Bursuceni K. 1, B. 21, Sk. 1, 3354–3103 calBCE	Consistent with being Maykop direct descendant	

**Wave 3: Yamna expansion**		

**Core Yamna genetic ancestry in the Eneolithic and Bronze Age NPR**		
I32534: Mykhailivka 1, Square VI, 3635–3383 calBCE	Core Yamna	
I20196: Crasnoe K. 9, B. 9, Sk. 2, 3352–3101 calBCE	Core Yamna	
I12229: Mayaky, Kurgan 1, Burial 9, 3088–2911 calBCE	Core Yamna	
I20079: Taraclia II, Kurgan 10, Burial 2, 2571–2355 calBCE	Core Yamna	
I12840: Dubynove, Kurgan 1, Burial 10, 2453–2148 calBCE	Core Yamna	
I16668: Revova, Kurgan 3, Burial 10, 2800–2000 BCE	Core Yamna	

**Mixtures of Core Yamna and European farmers**		
I1456: Durankulak, K. F, B. 15, 3500–3000 BCE	45% Core Yamna / 55% Globular Amphora	
Bulgaria Yamna, 3300–2500 BCE	78–100% Core Yamna / 0–22% YUN_CA	
Moldova Yamna, 3300–2500 BCE	84–100% Core Yamna / 0–16% YUN_CA	
Ukraine Yamna, 3300–2500 BCE	92–100% Core Yamna / 0–8% YUN_CA	
I17747: Tiraspol Kurgan 3, Burial 15, 2865–2576 calBCE	61% Core Yamna / 39% Trypillia	
I20076: Ocniţa Kurgan 1, Burial 3, 2906–2702 calBCE	88% Core Yamna / 12% Globular Amphora	
I4110, I5882, I5884: Deriivka I cemetery, 3500–2700 BCE^6^	36–46% Core Yamna / 23–44% BHG / 15–32% Trypillia	
I13071: Bil’shivtsi Individual 1, 2201–2032 calBCE	72% Core Yamna / 28% YUN_CA	
I12234: Liubasha and Sychavka Kurgans, 2434–1127 BCE	77% Core Yamna /15% Globular Amphora / 8% UNHG	

**Mixture of Core Yamna and Dnipro-Don Hunter Gatherers descendants**		
Don Yamna, 3200–2600 BCE	40% Core Yamna / 60% SSmed	

**Mixtures of Core Yamna and Steppe Maykop descendants**		
I20078: Taraclia II K. 2, B. 14, 3340–3034 calBCE	39% Core Yamna / 61% Steppe Maykop	
I17974: Bursuceni K. 1 B. 21, Sk. 2, 3334–3030 calBCE	82% Core Yamna / 18% Steppe Maykop	

**Yamna + Maykop descendants**		
I1917: Ozera Kurgan 18 Burial 14, 3096–2913 calBCE	50% Core Yamna / 50% Maykop	
Mayaky Yamna, 2900–2500 BCE	81% Don Yamna / 19% Maykop	

Notes: For admixture dates we give 95% CI.

a BPgroup are homogeneous people from Berezhnovka and Progress 2, at the bend between Caucasus Lower Volga (CLV) and Volga clines (Fig. 2a) ^7^.

b Balkan Hunter Gatherers (BHG) and Balkan farmers of Gumelnița/Karanovo from Yunatsite in Bulgaria (YUN_CA).

c PVgroup is a BP-related population from the CLV cline from Berezhnovka and Vonjucka, with more Aknashen (South Caucasus) ancestry than BPgroup.^7^

d Remontnoye is a mix of southern ancestry represented by either the Aknashen Neolithic of Armenia or Bronze Age Maykop, and a northern ancestry from the low-EHG end of the Volga Cline such as BPgroup^7^.

## Data Availability

Genotype data for individuals included in this study can be obtained from the Harvard Dataverse repository through the following link (https://doi.org/10.7910/DVN/CJTV3Q). The DNA sequences reported in this paper have been deposited in the European Nucleotide Archive under accession number PRJEB81468. Other newly reported data such as radiocarbon dates and archaeological context information are included in the manuscript and supplementary files.
