# Supplementary material for: A genomic history of the North Pontic Region from the Neolithic to the Bronze Age
Source: Nature. Author manuscript; Available in PMC 2025 Mar 15. (PMC11909631; doi:10.1038/s41586-024-08372-2)
Supplement: Supplement Information [file NIHMS2057029-supplement-Supplement_Information.pdf]

Supplementary File SI1

Archaeological background with archaeogenetic notes and highlights, burial descriptions, and isotope analysis of selected Specimens

Contents

1. Archaeological background (with archaeogenetic notes) 9
  1.1. Ponto-Caspian Steppe from the Early Holocene to the Neolithic period (9700-5000 BCE) 9
  1.2. The Eneolithic period (5000-3300 BCE) 10
    1.2.1. Northwest Pontic coast in the 4th millennium BCE 17
    1.2.2. Diet isotopes, Reservoir Effect, and the Usatove chronology 18
    1.2.3. Contacts between the NPR, the Caucasus, and the Near East 19
  1.3. The Early Bronze Age (3300/3000-2400 BCE) 22
    1.3.1. Yamna Archaeological Complex 22
    1.3.2. Catacomb Archaeological Complex 24
  1.4. The Middle Bronze Age (2400-2000 BCE) 25
  1.5. Flat-top kurgan sanctuaries in the NPR and the origin of Yamna 26

2. Burial descriptions 28
  Bulgaria 28
    Durankulak Kurgan F 28
    Golyamata Mogila (Popovo) 28
    Riltsi Kurgan 264 29
  Moldova 29
    Bursuceni Kurgan 1 29
    Cioburciu Kurgan 4 31
    Cotiujeni Kurgan 1 31
    Crasnoe Kurgan 9 32
    Cunicea 33
    Dănceni II 33
    Giurgiuleşti 34
    Glinoe (Hlinaia) 34
    Mărculeşti Kurgan 3 36
    Mereni II Kurgan 1 36
    Ocniţa Kurgan 1 37
    Sărăteni Kurgan 1 38
    Taraclia II Kurgans 2 and 10 38
    Tiraspol Kurgan 3 39
  Ukraine 40
    Bil’shivtsi 40
    Deriivka II 41
    Dubynove Kurgan 1 42
    Kam’yana Mohyla Kurgan 2 43
    Katarzhyno Kurgans 1 and 2 45
    Kolomiytsiv Yar Tract 48
    Mariupol Necropolis 48
    Mayaky 52
    Molyukhiv Bugor 57
    Mykhailivka 58

Liubasha Kurgan (Kurgan 2) ... 59
Odesa Kurgan ... 61
Ogrin-8 (Igren-8) ... 62
Revova Kurgan 3 ... 62
Shakhtar (Shevchenko) kurgan group ... 66
Soldats'ka Slava Kurgan ... 67
Sychavka Kurgan ... 67
Vapnyarka Kurgan 4 ... 68
Verteba Cave ... 71
Vynohradne Kurgan 3 ... 72
Yasynuvatka ... 73

3. Archaeogenetic notes and highlights ... 75
3.1. Genetic ancestry of Zhyovotylivka-Volchans'k/III-C burial type (ZV/III-C) individuals ... 75
3.2. Corded Ware connections ... 75
3.3. Uniparental lineage connections in the Ponto-Caspian region ... 76
3.3.1 MtDNA lineages ... 76
3.3.2. Y lineages ... 78

4. Strontium isotope analysis of specimens from the Neolithic Mariupol Necropolis and settlement ... 80

References ... 84

Table SI1. 1. Data summary report for specimens used in the analysis ... 3
Table SI1. 2. Strontium isotope ratios for samples from the Mariupol Necropolis and the Kalmius Settlement ... 83

Figure SI1. 1. Ochre Graves in the Ponto-Caspian region. ... 12
Figure SI1. 2. A cloak pin with beads and cylinder seals suspended from it. Uruk, 4th millennium BCE. Neues Museum, Berlin. Photo: M. Y. Videiko, 2023. ... 21
Figure SI1. 3. Hammer-top pin of the Yamna archeological complex. Stari Bilyari Kurgan 1 Burial 4. Photo: S. Ivanova, undated. ... 21
Figure SI1. 4. Flat-top kurgan sanctuaries and ancient river crossings in southern Ukraine ... 27
Figure SI1. 5. DOT Kurgan 1, Burial 9. Photo by V. Sinica, 2013. ... 34
Figure SI1. 6. SAD Kurgan 1, Burial 25. Photo by V. Sinica, 2013 ... 35
Figure SI1. 7. SAD Kurgan 1, 1Burial 26. Photo by V. Sinica, 2013 ... 36
Figure SI1. 8. Bi'lshivtsi (Kuty) burial. Image by T. Tkachuk ... 41
Figure SI1. 9. Deriivka 2 Burial 5. Photo by A. G. Nikitin, 2014. ... 42
Figure SI1. 10. Kam'yana Mohyla. Google Maps/Google Earth, https://www.google.com/maps, 2022. ... 44
Figure SI1. 11. Kam'yana Mohyla Kurgan 2 Burial 2. Photo: S. Makhortykh, 2017. ... 45
Figure SI1. 12. Neolithic Mariupol Necropolis. Drawing by M. Makarenko, 1933 [1] ... 49
Figure SI1. 13. Mariupol Necropolis, Burial 50. Photo from[1]. ... 50
Figure SI1. 14. Left: Mariupol Necropolis Burial 21. Right: a flint arrow or spearhead from the cremation on top of the burial. Images from[1]. ... 51
Figure SI1. 15. Mariupol Necropolis Burial 21. Photo by M. Makarenko, 1930. ... 52
Figure SI1. 16. Revova Kurgan 3 showing a reconstruction of a dolmen over the main Burial 19. Drawing by S. Ivanova and A. G. Nikitin. ... 63
Figure SI1. 17. Revova Kurgan 3, Burial 19. Photo by S. Ivanova, 2003. ... 65
Figure SI1. 18. Vapnyarka Kurgan 4 Burial 3. Photo by S. Ivanova, 2008. ... 69
Figure SI1. 19. Vapnyarka Kurgan 4 Burial 16. Photo by S. Ivanova, 2008. ... 70
Figure SI1. 20. Vynogradne Kurgan 3 Burial 15. Photo by I. D. Potekhina, 2012. ... 73
Figure SI1. 21. Yasynuvatka Burial 24, occipital view of the cranium. Photo: A. G. Nikitin, 2014. ... 74
Figure SI1. 22. Simplified geological map of Ukraine. ... 81
Figure SI1. 23. Comparison of the $^{87}Sr/^{86}Sr$ ratio of the three samples from Mariupol with ratios for baselines and human remains (including enamel, dentin, and bone samples) from other sites in Ukraine and Olenii (Russia). ... 82

Table SI1. 1. Data summary report for specimens used in the analysis.

№	Genetic ID	Specimen code	Site, Burial	^{14}C uncalBP (Lab Code)	BCE or Calibrated BCE, 2σ (95.4%)[a]	Genetic sex	MtDNA haplogroup	Y chromosome haplogroup
	**Mariupol type cemeteries, Ukraine (Neolithic)**							
1	I31730	MM.50a	Mariupol Necropolis Burial 50 (adult)[1]	6395±35 (PSUAMS-10373)	5474-5236 calBCE	M	U5a1+@16192	I2a1b1a2a2a
2	I31731	MM.50b	Mariupol Necropolis Burial 50 (immature)	3720±30 (BETA-681064)[b]	2204-1986 calBCE[c]	F(?)	U5b2 (<2X)[d]	
3	I27982	Yas23a/Ya42	Yasynuvatka, Burial 23a, Pit Б-1 [2,3]	n/a	5560-4750 BCE	M	U4a1	Q1b
4	I27983	Yas24/Ya40	Yasynuvatka, Burial 24, Pit Б-1 [2,3]	n/a	5560-4750 BCE	M	H	R1b1a1b1a1a2c1a3a2
5	I27986	Ya8	Yasynuvatka, Burial 35, Pit Б-1 [2–5]	6320±30 BP, BETA-441244/445800)[5]; 5810±60 (Ki-3162)[4]	5363-5216 calBCE; 4797-4502 calBCE	M	U5a2	P1
6	I27990	Yas44	Yasynuvatka, Burial 44, Pit Б-1[2,3]	n/a	5560-4750 BCE	M	U5a1	Q1b
7	I27992[e]	Yas50	Yasynuvatka, Burial 50, Pit Б-1[2,3]	n/a	5560-4750 BCE	F	K1a3	
8	I27994	Yas55	Yasynuvatka, Burial 55, Pit A2[2,3]	6650±30 (BETA-445802)[5]	5630-5484 calBCE	M	T2a1b	I2a1b2 (I-S2555)
9	I27995	Yas58	Yasynuvatka, Burial 58, Pit A2 [2,3]	n/a	5650-5450 BCE	M	U4b1b1	n/a
	**Cucuteni-Trypillia, Moldova (Eneolithic)**							
10	I7920	12M1	Cunicea-Prişanscaia Gora (Soldanești), Section 4, Layer 3, burial 4	4505±25 (PSUAMS-4872)	3350-3099 calBCE	M	K1b2	I2a1b1a2a2a2 (I-S12195)
11	I20069	DII.301	Dănceni II, Ground Burial 2 (301)	4435±20 (PSUAMS-8832)	3323-2935 calBCE	M	H	I2a1b1a2a2a (I-L699)
	**Cucuteni-Trypillia, Ukraine (Eneolithic)**							
12	I1926 [h]	1V1a-H1	Verteba Cave, Site 7[6], individual 3.17.1[7–9]	4906±23 (weighted mean[10] of 4890±30 (Beta-432808[8]) and 4925±33 (OxA-26207[7])	3756-3638 calBCE	M	H5a	G2a2b2a (G-P303)
13	I2109	3V3a-H3	Verteba Cave, Site 7[6], Individual V1-2SE (3.18.1)	4863±33 (OxA-26202)[7]	3710-3531 calBCE	M	J1c	G2a (G-L1259)
14	I2110 [h]	4V4a-H4	Verteba Cave, Site 7[6], Individual 3.14.1[7–9]	4976±33 (OxA-26203)[7]	3911-3650 calBCE	M	T2b	G2a2b2a (G-P303)
15	I2111 [h]	5V5a-H5	Verteba Cave, Site 7[6], Individual 3.16.1[7–9]	4888±32 (OxA-26204)[7]	3765-3543 calBCE	M	H+16311	G2a (G-P15)

16	I3151 [h]	6V6a-H6	Verteba Cave, Site 7, individual 3.15.1[7–9]	4807±33 (OxA-26201)[7]	3644-3527 calBCE	M	U8b1b	E1b1b1a1b1 (E-L618)
17	I1929	9 V9a-A22.2	Verteba Cave Site 6, Individual 2	4910±400 (Ki-14308)[6]	3900-3500 calBCE	F	H	
18	I7921	A22.P3	Verteba Cave, Site 6[6], Individual 3[6,7]	n/a	3900-3500 BCE	F	H2a	
19	I7923	RL2.05 (VRT-LR1)	Verteba Cave, Site 2[6], Individual 1	4955±30 (PSUAMS-8692)	3789-3649 calBCE	M	T2b+16362	J2a1
20	I7584	VCS1.CL.05	Verteba Cave Site 1[6], Individual 1	4875±25 (PSUAMS-10769)	3708-3543 calBCE	F	H5b	
21	I7586	VCS1.TBL.05	Verteba Cave, Site 5[6], Individual 2	4920±25 (PSUAMS-9527)	3766-3642 calBCE	F	J1c2	
22	I13068	VRT1.1.1	Verteba Cave, Site 7[6], cranium 1 (west)	n/a	3950-3500 BCE	M	T2	n/a
23	I13064	VRT-VSE.22N, VRT-VNWL3	Verteba Cave, Site 7[6], Individual 2	4820±30 (PSUAMS-7807)	3648-3528 calBCE	M	T2	I2a1b1a2b (I-Z161)
	**Usatove, Ukraine (Eneolithic-Early Bronze Age)**							
24	I12615	Ma6	Mayaky Sanctuary, Burial 1	6100±30 (BETA-432410)[5]	5208-4907 calBCE	F	U4b1b2	
25	I12228	Ma13	Mayaky Sanctuary, Burial 2	5580±30 (BETA-432406)[5]	4486-4350 calBCE	F	U4[f]	
26	I12229	Mau1	Mayaky Kurgan 1, Burial 9[11]	4375±25 (PSUAMS-7865)	3088-2911 calBCE	F	U5a1a1	
27	I12227	Ma2	Mayaky Kurgan 6, Burial 1	5060±30 (BETA 441234)[5]	3955-3783 calBCE	F	n/a	
28	I12704	Mau6	Mayaky Kurgan 7, Burial 2[11]	5144±20 (weighted mean[10] of 5530±32 (OxA-22959)[12], 5390±30 (BETA-441235)[5], and 5545±40 (PSUAMS-7793, this report)	3632-3368 calBCE[g]	M	T2h2	E1b1b1a1
29	I12710	Mau12	Mayaky Kurgan 8, Burial 4[11]	5295±30 (PSUAMS-7862)	3894-3379 calBCE[g]	F	W1	
30	I12707	Mau9	Mayaky Kurgan 8, Burial 6, Skeleton 2[11]	5330±40 (PSUAMS-7794)	3946-3385 calBCE[g]	F	U5a1d1	
31	I12701	Ma11	Mayaky Kurgan 9, Burial 1	4300±30 (BETA 432412)[5]	3011-2881 calBCE	F	X2	
32	I12706	Mau8	Mayaky Kurgan 9, Burial 2[11]	5444±19 (weighted mean[10] of 5471±24, 0xA-22960[12], and 5400±30, PSUAMS-7846)	3948-3653 calBCE[g]	F	X2d	
33	n/a	Ma12	Mayaky Kurgan 10, Burial 2, Skeleton 1[11]	5530±30 (BETA-432413)[5]	4045-3658 calBCE[g]	F	U4[f]	n/a
34	I1423 (I12705)	Mau7/MAJ1	Mayaky Kurgan 10, Burial 2, Skeleton 2[11]	5390±30 (PSUAMS-7845)	3950-3633 calBCE[g]	M	H+195	R1a
35	I7929	REV3.19	Revova Kurgan 3, Burial 19[13]	4905±20 (PSUAMS-4763)	3711-3639 calBCE	M	U4d3	J1 (J-FT265222)
	**Serednii Stih, Ukraine (Eneolithic)**							

36	I27282	Der15	Deriivka II, Skeleton 8[14]	5345±30 (PSUAMS-10370)	4322-4042 calBCE	M	U4b1a	I2a1b1a2
37	I28319	Der16	Deriivka II, Skeleton 5[14]	5344±29 (weighted mean [10] of 5380±90 (OxA-5032)[15] and 5340±30 (BETA-445808)[5]	4321-4052 calBCE	F	T2a1b	
38	I27283	Der41	Deriivka II, Skeleton 11[14]	5500±30 (PSUAMS-10371)	4444-4262 calBCE	M	U4c	I2a1b1a2a2a
39	I2108	Igren-8 9	Ogrin-8, Burial 20	5415±25 (PSUAMS-8220)	4340-4178 calBCE	F	U2e	
40	I1924	Igren 8-78	Ogrin-8, Burial 24	5105±25 (PSUAMS-8219)	3971-3802 calBCE	F	U5b2	
41	I27930	Igren 8-74	Ogrin-8, Burial 3a	n/a	4400-4000 BCE	M	U4	R1b1b (R-V88)
42	I7585 [h]	KST07	Kolomiytsiv Yar Tract, Burial 1[16]	5170±30 (BETA-523816)	4049-3820 calBCE	F	U4b1b2	
43	I31732	MM.21	Mariupol Necropolis, Burial 21[1]	5790±30 (PSUAMS-10374)	4715-4548 calBCE	F	n/a	
44	I1424	MOB1	Molyukhiv Bugor, Burial 6[15,17,18]	4943±28 (weighted mean[10] of 5020±50 (OxA-17502), and 4909±33 (OxA-17503)[15])	3777-3648 calBCE	M	U5a1	I2a1b1a2a2a (I-L699)
45	I1454	MOB3	Molyukhiv Bugor, Burial 4, Skeleton 2[17,18]	3680+70 (Ki-14236[18])	2286-1886 calBCE	F	U/K[17]	
46	I1430	VIN1	Vynogradne Kurgan 3, Burial 15 [17,19–21]	5230±60 (Ki-14726)[19]	4241-3951 calBCE	M	H13	I2a1b1a2a2a (I-L699)
	**Steppe Eneolithic, Ukraine**							
47	I12167 (I31733)	K1.10	Katarzhyno Kurgan 1, Burial 10[13]	5039±27 (weighted mean of 4970±110 (Ki-11376), 4950±70 (Ki-9523)[13], and 5060±30 (PSUAMS-10375)	3951-3715 calBCE	F	U5a2a2 (<2X)[d]	
48	I12618	V4.4	Vapnyarka Kurgan 4, Burial 4[22]	4480±30 (BETA-647661); 4100±80 (Ki-15013)[22]	3341-3031 calBCE; 2878-2476 calBCE	F(?)	U8b1a2a (<2X)[d]	
	**Steppe Eneolithic, Moldova**							
49	I20196	Kr/K9/B9/S2	Crasnoe Kurgan 9, Burial 9, Skeleton 2[23]	4510±25 (PSUAMS-8835)	3352-3101 calBCE	F	J1b1a1	
50	I17977	Kr/K9/B9/S2(d)	Crasnoe Kurgan 9, Burial 9, Skeleton 3 (child)[23]	n/a	3352-3101 calBCE (based on I20196 – 1st degree kin)	F	J1b1a (<2X)[d]	
	**Steppe Eneolithic/III-C type burials, Moldova**							
51	I17743	MeII1.15	Mereni II Kurgan 1, Burial 15	4515±30 (PSUAMS-7822)	3358-3100 calBCE	F	H+16311	
52	I17973	Br/K1/B21/S1	Bursuceni Kurgan 1, Burial 21, Skeleton 1[24]	4516±13 (weighted mean[10] of 4530±20 (PSUAMS-8723), 4520±25 (PSUAMS-8310) and 4500±20 (PSUAMS-8722)	3354-3103 calBCE	M	U1a1a	J2b2b2~ (J-Z42942)
53	I17974	Br/K1/B21/S2	Bursuceni Kurgan 1 Burial 21, Skeleton 2[24]	4470±20 (PSUAMS-8724)	3334-3030 calBCE	F	U5a1g1	

54	I17975 (I20195)	Br/K1/B21/S3	Bursuceni Kurgan 1 Burial 21, Skeleton 3[24]	n/a	3334-3030 calBCE (based on I17974 twin)	F	U5a (<2X)[d]	
55	I20078	TII.K2.14	Taraclia II Kurgan 2, Burial 14	4480±25 (PSUAMS-8833)	3340-3035 calBCE	M	H2a1	Q1b2b1b2b~ (Q-BZ1466)
56	I20079	TII.K10.2	Taraclia II Kurgan 10, Burial 2	3965±20 (PSUAMS-8834)	2566-2347 calBCE	M	K1b2b	R1b1a1b (R-M269)
	**Suvorove, Moldova (Eneolithic)**							
57	I20072	GK2.6	Giurgiulești, Burial 6 (3)[25]	5370±26 (MAMS-23175)[25]	4330-4058 calBCE	M	H13	Q1a2
58	I20073	GK2.11	Giurgiulești, Burial 11 (4)[25]	5571±32 (MAMS-28088)[25]	4348-4248 calBCE	M	K1b2b (<2X)	CF
	**Proto-Yamna, Bulgaria (Eneolithic)**							
59	I1456	DUR1	Durankulak Kurgan F, burial 15 (main burial)[26]	n/a	3500-3000 BCE	M	U5a2a	R1a1a1 (R-M417)
	**Yamna, Ukraine (Eneolithic-Early Bronze Age)**							
60	I32534	M1P1.6	Mykhailivka 1, square VI	4755±25 (PSUAMS-10750)	3635-3383 calBCE	F	H6a1	
61	I12170	K2.1	Katarzhyno Kurgan 2, Burial 1[13]	4490±30 (BETA-647662); 4270±90 (Ki-17331)[13]	3348-3038 calBCE; 3314-2537 calBCE	F	U4c1	
62	I20975	KM17P2	Kamyana Mohyla Kurgan 2, Burial 2[27]	4141±21 (BE-8040.1.1)[27]	2872-2626 calBCE	M	N1b1a2	R1b1a1b1b (R-M12149)
63	I12168	KTR-1.13	Katarzhyno Kurgan 1, Burial 13[13]	4359±16 (weighted mean [10] of 4375±20 (PSUAMS-4762) and 4335±25 (PSUAMS-9815)	3018-2911 calBCE	M	U4a	R1b1a1b1b3 (R-Z2108)
64	I11999	KTR-1.1	Katarzhyno Kurgan 1, Burial 1[13]	4045±20 (PSUAMS-4903)	2626-2476 calBCE	M	U4a2	R1b1a1b1b3 (R-Z2108)
65	I12233	L2.16	Liubasha Kurgan, Burial 16[13]	4330±35 (PSUAMS-7790)	3074-2888 calBCE	M	U4a1	R1b1a1 (R-P297)
66	I7927	R3.7	Revova Kurgan 3, Burial 7[13]	3910±60 (Ki-11058)	2568-2206 calBCE	n/a	U5a (>2X)[d]	n/a
67	I7928	R3.16	Revova Kurgan 3, Burial 16[13]	4135±60 (Ki-11059)[13]	2885-2501 calBCE	M	U[28]/U+152, 16311 (>2X)[d]	n/a
68	I12005	SL-10	Soldats'ka Slava Kurgan, Burial 10[13]	4350±50 (Ki-17903)[29]	3265-2883 calBCE	M	T2c1a2	n/a
69	I12843	S15	Sychavka Kurgan, Burial 15[30]	4097±24 (weighted mean [10] of 3960±80 (Ki-16610)[30] and 4110±25 (PSUAMS-9556)	2857-2502 calBCE	M	U5a1a1	R1b1a1b1b (R-M12149)
70	I12619	V4.16	Vapnyarka Kurgan 4, Burial 16[22]	4050±60 (Ki-15014)[22]	2868-2462 calBCE	M	W3a1a	R1b1a1b1b3 (R-Z2108)
71	I3141 [h]	Yamna 5	Shevchenko (Shakhtar) Kurgan 28, Burial 12[8]	n/a	3300-2500 BCE	F	H	
	**Yamna, Bulgaria (Early Bronze Age)**							

72	I1448	POP1	Popovo, Golyamata Mogila, Burial 29[31]	n/a	3300-2500 BCE	F	H6a1b	
73	I1427	POP4	Popovo, Golyamata Mogila, Burial 37[31]	n/a	3300-2500 BCE	M	U5a1a1	R1b1a1b1b3 (R-Z2108)
74	I1428	RIL3	Riltsi Kurgan 264, Burial 5[32]	n/a	3360-2890 BCE	F	K	
	**Yamna, Moldova (Eneolithic, Early Bronze Age)**							
75	I20067	CiK4.3	Cioburciu Kurgan 4, Burial 3	n/a	3300-2500 BCE	F	I4	
76	I20068	CoK1.6	Cotiujeni Kurgan 1 Burial 6	4175±20 (PSUAMS-10740)	2881-2671 calBCE	M	H13a2b2a	R1b1a1b1b (R-M12149)
77	I12507	Glinoe DOT K1B9	Glinoe (Hlinaia) DOT Kurgan 1, Burial 9	4275±20 (PSUAMS-10777)	2914-2882 calBCE	F	U5a1a1	
78	I12512	Glinoe SAD K1B25	Glinoe (Hlinaia) SAD Kurgan 1, Burial 25	4165±25 (PSUAMS-10778)	2881-2633 calBCE	M	H2a1	R1
79	I12637	Glinoe SAD K1B26	Glinoe (Hlinaia) SAD Kurgan 1, Burial 26	n/a	3300-2500 BCE	M	U2e3	R1b1a1b1b (R-M12149)
80	I17742	MK3.15	Mărculeşti Kurgan 3, burial 15	n/a	3300-2500 BCE	F	n/a	
81	I20076	OcK1.3	Ocniţa Kurgan 1, Burial 3	4230±25 (PSUAMS-11947)	2906-2702 calBCE	F	U5a2a1	
82	I17744	SK1.1	Sărăteni Kurgan 1, Burial 1	4115±20 (PSUAMS-8442)	2860-2578 calBCE	M	H6a1a	R1b1a1b1b3 (R-Z2108)
83	I17745	SK1.9	Sărăteni Kurgan 1, Burial 9	4105±25 (PSUAMS-8309)	2862-2573 calBCE	M	H13a2b2a	R1b1a1b (R-M269)
84	I17747	TiK3.15	Tiraspol Kurgan 3, Burial 15	4115±25 (PSUAMS-7864)	2865-2576 calBCE	M	U4d3	R1b1a1b1b (R-M12149)
	**Catacomb, Ukraine (Early Bronze Age)**							
85	I13071	BLV1	Bil'shivtsi, Individual 1[33]	3720±25 (PSUAMS-7848)	2201-2032 calBCE	F	T2c1d1a	
86	I12621	DBN1.9	Dubynove Kurgan 1, Burial 9[13]	3970±35 (PSUAMS-7791)	2575-2348 calBCE	M	U5b	n/a
87	I12840	DBN1.10	Dubynove Kurgan 1, Burial 10[13]	3830±30 (PSUAMS-7805)	2453-2148 calBCE	F	H2b	
88	n/a	D1.11	Dubynove Kurgan 1, Burial 11[13,28]	3720±70 (Ki-11202)	2343-1905 calBCE	n/a	U5a1g1	
89	I7926/I13062	D1.12/DBN-1.12	Dubynove Kurgan 1, Burial 12[13,28]	3900±80 (Ki-11203)	2580-2139 calBCE	M(?)	H2a	
90	I11850	ODK1	Odesa Kurgan, Burial 10	4150±25 (PSUAMS-11224)	2874-2630 calBCE	F	H+16311	
91	I16668	REV3.10	Revova Kurgan 3, Burial 10[13]	n/a	2800-2000 BCE	M	U3a (<2X) [d]	CF
92	I16669	REV3.13	Revova Kurgan 3, Burial 13[13]	3940±60 (Ki-11172)	2580-2207 calBCE	M(?)	H32 (<2X) [d]	
93	I13072	SL-1	Soldats'ka Slava Kurgan, Burial 1[13]	n/a	2800-2000 BCE	M	T1a1 (<2X) [d]	n/a

94	I12617	V4.3	Vapnyarka, Kurgan 4 Burial 3[22]	4089±24 (weighted mean[10] of 3960±70 (Ki-15230)[22], and 4105±25 BP (PSUAMS-10808)	2851-2500 calBCE	F	R1a1a1	
	**Multi-Cordoned Ware/ Babyne, Ukraine (Bronze Age)**							
95	I12234	L3	Liubasha Kurgan, Burial 3[13]	3080±70 (Ki-11176)[13]	1499-1127 calBCE	M	R1a (<2X) [d]	R1a1a (R-M198)
96	I7925	L9	Liubasha Kurgan, Burial 9[13]	3520±80 (Ki-11173)[13]	2119-1624 calBCE	M	I1a1	R1a1a1b
97	I12235	L11	Liubasha Kurgan, Burial 11[13]	3230±70 (Ki-17332)[13]	1686-1311 calBCE	M	H13a1a2	R1a1a
98	I7925(d)	N2.12	Liubasha Kurgan, Burial 12[13]	n/a	2120-1620 BCE (based on I7925 twin)	M	I1a1	R1a1a1b
99	I16674	L2.15	Liubasha Kurgan, Burial 15[13]	3740±70 (Ki-11201)[13]	2434-1943 calBCE	M	H15a1a1	R1b1a1b1b (R-M12149)
100	I16670	REV-3.14	Revova Kurgan 3 Burial 14	3590 ± 70 (Ki-11175)	2139-1748 calBCE	n/a	n/a	
101	I12231	S18	Sychavka Kurgan, Burial 18	3490±90 (Ki-16611)	2118-1565 calBCE	M	U4a1b	R1b1a1a2a2

[a] Radiocarbon dates were calibrated using the IntCal20 atmospheric curve[34], unless otherwise indicated.
[b] Radiocarbon date from enamel carbonate.
[c] Calibration: BetaCal4.20; HPD method: INTCAL20.
[d] Mitochondrial coverage obtained during whole-genome amplification is below 2X and is considered unreliable according to the standards used by the Reich's lab.
[e] The specimen has a high contamination rate mtcontam=[0.717,0.942]. It is not cladal with Ukrainian Neolithic hunter-gatherers, which is a further consequence of contamination.
[f] Mitochondrial DNA haplogroup assignment by low-resolution PCR-SNP analysis of the diagnostic coding and hypervariable I (HVR-1) regions of mtDNA followed by Sanger sequencing at GVSU, following a protocol detailed in[28,35].
[g] Calibrated using reservoir offset of 440±45 ^{14}C years.
[h] This study reports enhanced coverage.

1. Archaeological background (with archaeogenetic notes)

Written by Alexey G. Nikitin, with contributions from Svitlana Ivanova, Mykhailo Videiko, Iosif Lazaridis, and Nick Patterson

In this section, we will use a tiered structure for the designation and taxonomic grouping of archaeological type units, namely archaeological circle/horizon/complex – archaeological group – regional subgroup – local archaeological type. An archaeological circle (horizon, or complex) is composed of archaeological groups, connected by isomorphism of the structure, typological series, and similarity of features to such an extent that it allows us to assume kinship among these groups through common origin or the presence of common elements, which are distinctly different from similar taxonomic units across the geographic and chronological scale; or a group of related archaeological groups characterized by the same typological types. An archaeological group is defined by a recurrent assemblage of similar artifacts and features found within a defined geographic area over a particular time period, and which can be further subdivided into regional subgroups. A local archaeological type is defined by a regionally restricted set of artifacts and features that are variably represented within an archaeological group/subgroup.

1.1. Ponto-Caspian Steppe from the Early Holocene to the Neolithic period (9700-5000 BCE)

The Eurasian steppe is an extensive temperate grassland, stretching across 8000 km from Hungary to Manchuria, covering nearly one-fifth of the Earth's circumference. The Ural Mountains divide this vast expanse into two distinct ecosystems: the Pontic-Caspian steppe to the west, and the East Eurasian steppe to the east. The Pontic-Caspian steppe encompasses coastal Bulgaria and Romania, southern Moldova, Ukraine, the Kuma-Manych depression in the Northern Caucasus foreland, and the lower Volga region. In the North Pontic region (NPR), a transitional forest-steppe vegetation zone exists between the southern steppe and the forest north of the Black Sea. Climatic features, vegetation types, and faunal compositions vary in the NPR based on proximity to major waterways such as the Dnipro River, which bisects the region from northwest to southeast, and the southern border formed by the Black Sea.

Historically, the Eurasian steppe has played a crucial role as a major thoroughfare for travel and trade. Its expansive flat terrain provides an optimal route connecting Asia, the North Caucasus, and Europe.

During the last Ice Age, the southern part of the NPR was a peri-glacial tundra steppe. It likely served as a glacial refugial zone for human populations of central and eastern Europe during the Last Glacial Maximum (LGM)[36]. These migrations brought the carriers of Western and Eastern Hunter Gatherer (WHG and EHG) ancestry closer together. These groups remained genetically separated until the inflow of WHG ancestry in the gene pool of hunter-gatherers of the Dnipro Valley in the early Neolithic[8]. During the LGM, humans settled along the river valleys such as the Danube, Dnipro, and Dniester rivers, which formed a shared alluvial plain since the late Pleistocene[37], and their river mouths extended ca. 100 km into the now submerged coastline of the brackish Neoeuxinian Lake that existed in the place of the Black Sea at the time.

Following the LGM, the melting of glaciers resulted in the rise of the Caspian Sea levels, causing the Khvalynian transgression. The Caspian Sea subsequently spilled into the

Neoeuxinian Lake via the Manych depression, inundating the coastal shelf, raising the water levels of the lake by 20 meters during 10000-9000 BCE[38] and drowning the coastal glacial refugia such as the Black/Azov Sea Lowland[39]. The prevalence of grasses and broad-leaved trees in the area during the Early Holocene indicates the warming of the climate compared to the LGM, when the existing forest was dominated by pine suggesting of cool and dry conditions at that time[40].

In the Early Holocene, human habitation in the NPR continued to concentrate along the river valleys. The Neoeuxinian Lake remained disconnected from the World Ocean until ca. 7600 BCE. The two-way connection between the Black Sea and the Sea of Marmara became re-established by the onset of the Holocene Climatic Optimum ca. 7000 BCE[40,41]. The presence of a deciduous forest in the northeast NPR During the 6th – late 5th millennium (ca. 6000-4200) BCE[40] corroborates the establishment of an overall warming trend and humid conditions[40] during the late part of the Stone Age.

The mid-7th – 6th millennium BCE that outlines the Neolithic period of the NPR chronology that is primarily defined by a coexistence, with various levels of interaction, between the Neolithic agricultural communities of the Balkans and fisher-hunter-foragers living along the tributaries to the Black Sea. The agricultural revolution in the NPR begins in its western part with the establishment of Karanovo-Starčevo-Körös-Criş farming communities in the late 7th - early 6th millennium BCE, thus coinciding with the earliest stages of the Neolithization of Europe. The LBK archaeological complex, which formed in central Europe in the early 6th millennium, reached the Prut-Dniester interfluve in the northwestern forest steppe area of the NPR shortly thereafter. Over 300 LBK sites are now known in the area east of the Carpathians. The site of Kamyane-Zavallia in south-central Ukraine (48.579, 30.059) is the easternmost LBK site in the NPR region excavated to date[42]. A detailed review of the presence of LBK in west-northwest NPR can be found in[43].

The central and eastern NPR in the Neolithic were settled by populations that followed Mesolithic subsistence practices and who had limited interactions with the farming world of the Balkans. The archaeological space of these groups is generally defined by extended supine cemeteries of Mariupol type (M-t cemeteries) named after the eponymic Neolithic Necropolis at Mariupol[1]. Ukrainian archaeology scholars have different views on archaeological group divisions within the populations that left M-t cemeteries. Genetically, the M-t Neolithic groups studied to date display homogeneity irrespective of cultural subdivisions[5,8]. The Neolithic of the North Pontic is further detailed in[44].

We note that two published Mesolithic samples from the Deriivka 1 archaeological site, I5876 (7040-6703 calBCE) and I5885 (6392-5927 calBCE) cluster with the Neolithic Ukraine samples on the PCA in this report (Fig 2a). Their radiocarbon dates also postdate the other four Mesolithic Ukraine samples published to date. If we consider the Ukrainian hunter-fisher-forager ancestry transformation to be associated with the processes that led to the Neolithic transition, these genetic and radiocarbon dating results, taken together, suggest assigning the two abovementioned Deriivka samples to the Neolithic, thus extending the Mesolithic-Neolithic boundary in the Dnipro Valley to the first half of the 7th millennium BCE.

1.2. The Eneolithic period (5000-3300 BCE)

The examination of archaeological materials of the Eneolithic North Pontic region suggests that the formation of the steppe archaeological groups in the NPR in the 5th - 4th millennium BCE

was affected by the dynamics of the economic and cultural development of neighboring agricultural societies, represented by the Cucuteni-Trypillia archaeological complex in the North Pontic forest-steppe area, the Darkveti-Meshoko archaeological complex, and, later, the Maykop-Novosvobodnaya complex of the North Caucasus. Archaeological sources show that the general stability of the development of these agricultural societies, primarily the Cucuteni-Trypillia, also ensured the stability of the development of their steppe neighbors.

Interactions between the steppe populations and farmers of the Gumelniţa-Karanovo, Cucuteni-Trypillia, and Darkveti-Meshoko become evident since the beginning of the 5th millennium BCE. These integrations lasted through the end of the early Eneolithic, ca. 4200 BCE. It has been suggested that the switch from the collective extended supine burials of the Neolithic to a new steppe burial tradition of the individual contracted on-the-back burials (Chapli-type burials, Kryvyi Rih, Suvorove, etc.) is considered to be a reaction of a stratum of steppe populations to first direct contacts with agricultural societies, reflecting significant changes in the economic, social, and spiritual spheres of life of the steppe inhabitants[45].

The contracted on the back position is found in a series of the so-called Ochre Graves, dating to the second half of the 5th millennium BCE and stretching from the North Caucasus to the west Pontic. We can cautiously speculate that Ochre Graves represent Wave 1 of the CLV expansion. According to[46], these include necropoli at Decea Mureşului and Fălciu in Romania, Giurgiuleşti in Moldova, Suvorove II, Kryvyj Rih, Chapli, Perto-Svystunove, Zaporizhzhia, Novodanylivka (one grave containing 15 long flint blades and a stellae with petroglyphs), Oleksandrivs'k, four Ochre Grave burials among Deriivka II and Lower Mykhailivka burials in a necropolis adjacent to a settlement in Ukraine, Archara, Dzhangar, Livencovka (Rostov-on Don), Komarovo, Stanitsa Staroniznestablievskaja, Verkhnii Akbash (Ochre burial №11 and an inlet Burial №10 of late Maykop ([47]citing[48]), or, possibly, Zhyvotylivka (see a discussion in[47]), or Usatove type[46]), Volgodonsk (Khutor Popova) in Russia, Gonova Mogila near Tărgovište in Bulgaria, single burials at Csongrád-Kettőshalom in Hungary, Feldioara in Romania, Căinari (4449-4344 calBCE, 5553±32 BP, R-combine of 5580±50 BP and 5536±40 BP, KIA-369) in Moldova, Casimcea (with 15 lance tips), Lungoci in Romania, Reka Devnja (with 32 gold rings) in Bulgaria, Donetsk (with a three-piece composite spear), Luhansk, and Mariupol (Burial (№24) in Ukraine, Kokberek and Nikol'skoye in Russia, double burials at Meşcreac in Romania and at Yama near Zvanivka in Ukraine, two individual burials at Kyulevcha in Bulgaria, near Frolovo and at Veselaya Roscha in Russia, Sunzha in North Ossetia-Alania and Sunzha near Ebarg-Yurt in Ghalghai Mokhk (Ingushetia), and a double burial with a cremation at the Mariupol Neolithic necropolis (Fig. SI1.1.1). Ochre Grave burials at the Neolithic Mariupol necropolis potentially represent a transition from the Neolithic M-t populations to the Eneolithic Ochre Grave/CLV Wave 1 migrants. Eneolithic burials at the Nalchik and Progress-2 cemeteries in the north Caucasus and Berezhnovka in the Middle Volga dated to the second half of the 5th millennium BCE, as well as Oleksandriia and Ogrin-8 in Ukraine containing ochre burials among the early graves, should be considered part the Ochre Grave complex.

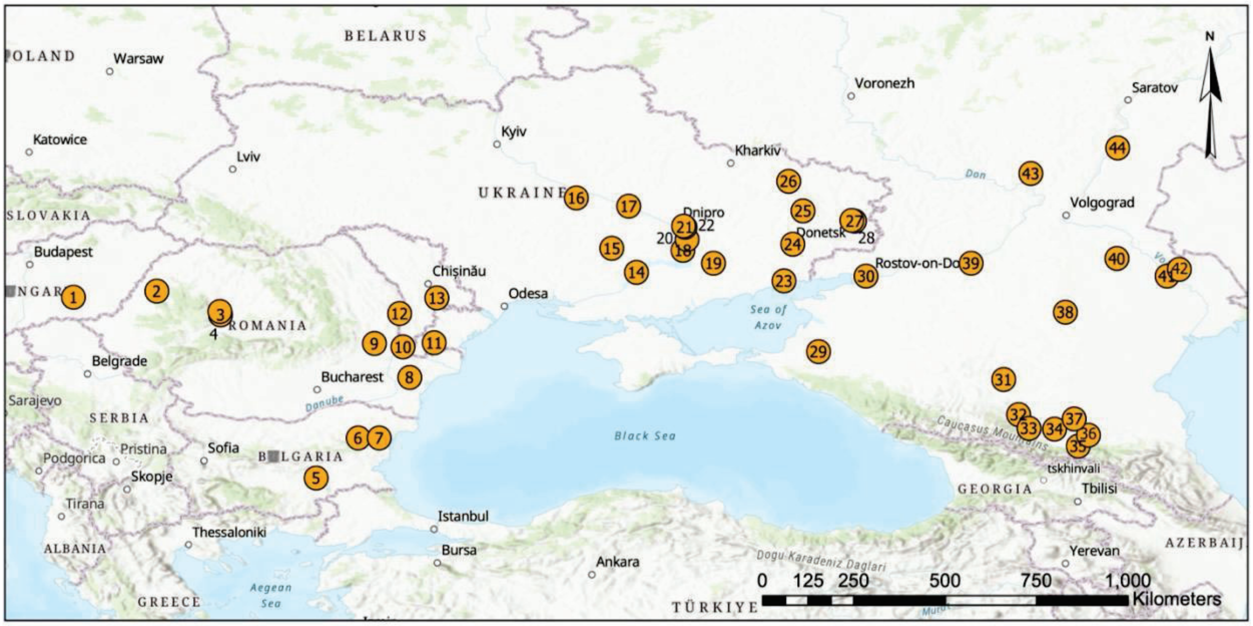

Figure SI1. 1. Ochre Graves in the Ponto-Caspian region.

Map was created using ArcGIS Pro v 3.1 (Environmental Systems Research Institute, Redlands, California, USA). Basemap credits: Esri, TomTom, Garmin, FAO, NOAA, USGS, Esri, USGS.
1, Csongrád-Kettőshalom; 2, Feldioara; 3, Meşcreac; 4, Decea Mureşului; 5, Tărgovište; 6, Kyulevcha; 7, Reka Devnja; 8, Casimcea; 9, Lungoci; 10, Giurgiuleşti; 11, Suvorove; 12, Fălciu; 13, Căinari; 14, Mykhailivka; 15, Kryvyj Rih; 16, Molyukhiv Bugor; 17, Deriivka; 18, Zaporizhzhia; 19, Novodanylivka; 20, Perto-Svystunove; 21, Chapli; 22, Ogrin-8; 23, Mariupol; 24, Donetsk; 25, Zvanivka; 26, Oleksandriia; 27, Luhansk; 28, Oleksandrivs'k; 29, Stanitsa Staroniznestablievskaja; 30, Rostov-On-Don; 31, Aleksandrovskoye (Veselaya Roscha); 32, Progress 2; 33, Nalchik; 34, Verkhnii Akbash; 35, Sunzha (Alania); 36, Sunzha (Ingushetia); 37, Ongusht; 38, Khar Usn; 39, Volgodonsk; 40, Dzhangar; 41, Nikol'skoye; 42, Kokberek; 43, Frolovo; 44, Berezhnovka.

The often lavishly furnished but inconspicuous in the landscape Wave 1 ochre graves date in the 4500-4000 BCE range, while the graves of the Wave 2 steppe people in the 4th millennium are largely inventory-less but appear under prominent above-ground earth mounds. Perhaps the changing burial ritual, which later becomes a hallmark of the Yamna, is associated with some change in ideology, possibly triggered by the environmental upheaval that caused the steppe hiatus. The difference in opulence among the ochre graves (from lavishly furnished to lacking any inventory) suggests that opulence may be a differentiating factor among the interred in the ochre graves where burials with an inventory represent the group's elite. Judging by the location of ochre graves in the North Caucasus-Caspian region (as we have shown in this report and in[49] to be one of the starting points of the CLV expansion), Wave 1 may have started from two relatively distinct geographic points, one in the Lower Volga-Don area and another in the North Caucasus piedmont. We can speculate that the former was composed of BPgroup carriers, while the latter had PVgroup component (or stand-alone Aknashen admixture), which would explain the Aknashen component in Usatove. The possible Usatove-type ochre grave at Verkhnii Akbash[46] provides an archaeological link between the North Caucasus and Usatove. The relative scarcity of ochre graves in the Dnipro-Dniester interfluve may suggest a competing presence of a different population in that territory, the best candidate for which is Trypillia[50].

The presence of steppe people in the Eneolithic NPR can be found in the ceramics style of their faming neighbors. In the early Eneolithic (ca. 4500-4200 BCE), decorative elements associated with the steppe people of the Skelya group, collectively known as Cucuteni C ceramics, are commonly found on Trypillian ceramics beginning with Trypillia A ([51] and references therein). In connection with the Cucuteni C ceramics, assumptions were made about the influx of the steppe populations into Trypillian communities. Some scholars link the spread of the steppe-derived type of ceramics in Trypillia with "steppe wives", who may have made these ceramics according to the traditions of their tribes [16,51]. The presence of CLV ancestry in early Trypillia (this report) corroborates this assertion.

According to the archaeology, the early phase of mobile steppe people groups in the NPR, traditionally designated as the Serednii Stih Archaeological Complex, included archaeological sites such as Oleksandriia (third layer), Serednii Stih II, Stril'cha Skelya, as well as burial complexes of the Novodanylivka type [52,53]. This early phase likely represents Wave 1 of the CLV expansion into the NPR. At the same time, the genetic ancestry of an individual from the Decea Mureșului Ochre Grave cemetery in Transylvania, Romania, was determined to be EEF-derived [5], indicating a diffusion of cultural traditions of the Wave I migrants into the farming communities of the west Pontic.

Novodanylivka burials contain copper items of Balkan provenance[53], indicating contacts between Novodanylivka and the west Pontic, which can be traced to the operation of the circum-Pontic trade network ca. 4700-4200 BCE[16,54]. Other authors suggested the Eneolithic steppe people established a local metallurgy center in the Dnipro Rapids area[52], halfway between Varna and Khvalynsk, which may explain a pronounced presence of Ochre Graves in that area (Fig. SI1.1.1).

The overlapping second wave of CLV migrants in the NPR appears to have been more heterogeneous than Wave 1. People of the CLV Wave 2 interacting with Neolithic hunter-fisher foragers in the Dnipro Valley formed the ancestry of the 4th millennium BCE pastoralist steppe and forest-steppe people in the NPR, which we refer to as Serednii Stih in[49] and in this report, as well as Yamna and Zhyvotylivka-Volchans'k, the latter with an admixture from steppe Maykop and Eneolithic European farmers. Perhaps locally unadmixed Wave 2 migrants produced monuments of the Katarzhyno-type in the steppe. Other Wave 2 migrants formed the remaining diversity of the Eneolithic steppe groups of the 4th millennium, some without clear cultural designation[13,55], but likely sharing common genetic ancestry.

The middle Eneolithic in the steppe (ca. 3900-3500 BCE) follows the so-called hiatus period (ca. 4200-3900 BCE)[52] with less clearly defined chronological sequence of steppe monuments and likely reflecting regional transformations precipitated by atmospheric cooling and fluctuations of the Black Sea level[54,56]. Climatic conditions in the steppe are thought to have returned to more optimal for human habitation after 3900 BCE, which facilitated subsequent cultural transformations[57]. The Middle Eneolithic in the NPR corresponds to the height of Trypillian development (the BII-CI and CI period of Trypillian chronology) and the formation of archaeological groups in the steppe and, partly, forest-steppe zones such as the Lower Mykhailivka, Deriivka II, Kvityana and Konstantinovka, characterized by different forms of complex economy. Such as, bearers of Lower Mykhailivka traditions were among the first full pastoralists in the North Pontic steppe with a developed understanding of domestication and the use of secondary animal products such as milk. Faunal assemblages of Lower Mykhailivka are dominated by domestic ruminants such as sheep and goats, followed by cattle, with a very minor inclusion of non-ruminant herbivores such as horses[58].

The middle Eneolithic was also the time of the expansion of complex burial structures in the steppe. These include cromlechs, stone rings, sanctuaries with pillar structures, ring ditches, and a complex combination of black earth and clay in the structure of the embankment. Ritual steppe architecture of this period acquires considerable monumentality, and its main structural features resemble funeral megalithic architecture of central and northwestern Europe. Some of the earliest such structures in the NPR appear in the steppe-Trypillia contact zone of Pobuzhzhia (the Southern Buh basin) and the Dniester-Prut interfluve[45]. Ritual monumental structures from the middle Eneolithic period are also known in the lower Dnipro and the Molochna River basins. Similar burial structures, containing ditches and cromlechs, accompany burials containing Maykop-Novosvobodnaya burial goods in Siverskyi Donets, Lower Don, and the Transcaucasia.

In the middle Eneolithic, active advance of Trypillian groups towards the steppe zone took place, coinciding with the appearance of Trypillian mega-sites along the forest steppe-steppe boundary in south-central Ukraine. In this advance, Trypillians reached not only the more southern regions of Pobuzhzhia (seen a series of sites with painted Trypillian ceramics such as Novorozanivka, Tashlyk 2, Pugach, etc.) but also the Dnipro (exemplified by finds of clay Trypillian figurines and a flint axe on the east bank of the former Kakhovskiy reservoir near the village of Velyka Lepetykha, in the Kherson region). In contrast to the earlier Trypillia BI and BI-II periods, evidence of steppe elements and imports at Trypillian settlements, including the megasites, is currently scarce. At the same time, a chain of kurgans, with main burials either directly dated or typologically assigned to the Eneolithic, including Katarzhyno Kurgan 1, Dubynove Kurgan 1, and Revova Kurgan 3 with its main burial carrying Usatove genetic ancestry, stretches along a south-north line from Usatove-Velykyj Kuyalnik to the Majdanets'ke-Nebelivka-Tal'yanky megasite concentration at the steppe–forest-steppe boundary, suggesting a potential connection between the Usatove and the megasites' populations. On the other hand, Trypillian imports and influences are evident in the Deriivka-type forest-steppe communities of the Serednii Stih horizon in the Dnipro Valley during this period, as seen in both cemeteries and settlements.

Prior to this report, only a few representatives of the Pontic steppe people from the first half of the 4th millennium BCE have been studied archaeogenetically. These come from an Eneolithic cemetery at Oleksandriia in east Ukraine, the Deriivka II cemetery in the Middle Dnipro Valley, and a Trypillian Kolomiytsiv Yar Tract (KYT) settlement in central Ukraine. Oleksandriia individual (I6561) carried early European farmer (EEF) ancestry, potentially from interactions with farming groups such as Trypillia[8]. Two-thirds of the genetic ancestry of the Deriivka II individual was inferred to be derived from EHG and Caucasus Hunter-Gatherer (CHG)/Iranian Neolithic (a genetic mix broadly defining the "steppe ancestry"), with one-third of the ancestry derived from the Mesolithic and Neolithic populations of the Dnipro Valley[5]. The KYT individual's genetic ancestry was primarily EHG and CHG/Iranian Neolithic-related, with some admixture of WHG ancestry best proxied by Iron Gates Mesolithic[5], potentially mediated through the Neolithic populations of the Dnipro Valley[16].

It is considered that the Pontic steppe groups such as the Katarzhyno type contributed to the formation of the Cernavodă I archaeological group through interactions with Gumelnița and, potentially, remnants of Hamangia, ca. 4100-3900 BCE, and, eventually, forming Usatove after 3800 BCE ([59], Section 1.2.1). Based on the data presented in this report, Cernavodă I was formed by varying contributions from European farmers and the people of the CLV cline including BPgroup and Maykop/Remontnoye, while Usatove is modeled as a roughly even mix of Trypillia

and the PVgroup end of CLV cline ancestries, or a mix of BPgroup and Caucasus Neolithic (Aknashen) ([49]; main text, Table 1; Supplementary Information, section 2).

BPgroup ancestry in Trypillia, estimated to be ca. 5% at the time of the formation of Trypillian ancestry (main text, Table 1), continues to persist in Trypillians into the second half of the 4th millennium BCE, potentially maintained by interactions between late Trypillian groups and Usatove. Individual I20069 from Dănceni, representing the Gordinești group of late Trypillia, has a 25.8±2.4% BPgroup ancestry (Supplementary Information, section 2). The diet of I20069 was similar to that of the Trypillian population from Verteba and distinct from that of the Usatove population from Mayaky (Online Table S4.3).

The late Eneolithic period (3500-3000 BCE) corresponds to Trypillia CII and represents a transition to the Early Bronze Age (EBA). This period is characterized by the fragmentation of the Cucuteni-Trypillia complex into a number of regional groups. This fragmentation also affected the steppe zone, where archaeological groups of the middle Eneolithic took part in the formation of separate local types, which, as a rule, were syncretic in their composition. Trypillia and Maykop-Novosvobodnaya either influenced or took a direct part in the formation of these steppe subgroups[45].

The intertwining of traditions is evident in monuments from the eastern part of the Pontic steppe (to the south of Zaporizhzhia and the north of Tavria). These kurgans, archaeologically associated with Lower Mykhailivka[60] despite postdating the Lower Mykhailivka chronologically[61], consisted of several separate burials, in which the dead were buried in a contracted position on their side. Quite often these burials were accompanied by small round-bottomed and flat-bottomed pots with river shell admixtures in clay. The surface of most of the pots was well smoothed, almost to a shine. Some, mostly flat-bottomed, pots contained thin rope imprints. This flat-bottomed ware imitated the kitchen ware traditions of late Trypillia, while polished ware reflected the influence of late Maykop. Typical late Maykop pots were found in these burials as well[60].

Further to the north, in the territory between the Southern Buh and the Dnipro, the coexistence of different traditions, both in ritual practices and in material culture, was even more pronounced. Such as, late Eneolithic populations of the Dnipro-Buh group, exemplified by kurgans near Pokrov and Shyroke in the Dnipropertrovsk Region[62], combined features of Lower Mykhailivka, Kvityana, Katarzhyno-type, and Trypillia. It is not uncommon for the Dnipro-Buh group to house, under the same kurgan, a group of several burials in which the dead were buried in extended supine, as well as contracted on the side and back burial positions.

The syncretic composition of steppe communities of the late Eneolithic also pertains to the monuments of the Zhyvotylivka-Volchans'k (ZV)/ III-C burial type[47,63]. These burials are characterized by the contracted on the side burial position with palms in front of the face, heads in the southern direction, accompanied by arcuate staff-shaped bone and metal Bolgrad-type pins imitating the staff-shaped pins of Maykop-Novosvobodnaya, as well as late Trypillia painted ceramics and Maykop-type incised ("parquet"-style) and glazed pottery[64–66]. People associated with ZV/III-C monuments utilized ceramic vessel forms, such as amphoras, goblets, and bowls, made using atypical technology for the steppe.

The ZV/III-C burial type is represented in the steppe zone by burials in kurgans. It is worth noting that ZV/III-C burial traditions are similar to those of the earlier Lower Mykhailivka group, and it is conceivable that these two groups are part of the same archaeological continuum. The spread of ZV/III-C traditions over an entire North Pontic area between the Danube and the Prut in the west to the Lower Don in the east reflects the nomadic and highly mobile lifestyle of

ZV/III-C. Most ZV/III-C monuments in the Pontic steppe are dated to ca. 3350-3000 BCE ([64], this report), thus coeval with the Yamna initial expansion phase. Taking into account ^{14}C date from a Bronze-Age burial from Taraclia II.10.2 (I20079, 2566-2347 calBCE) with III-C burial features, the terminal phase of ZV/III-C burials may have extended into the middle 3rd millennium BCE, by which time, judging by the genetic ancestry of I20079, the bearers of ZV/III-C burial type derived all their ancestry from the Core Yamna. In the late 4th millennium, they were among the earliest in the NPR carriers of Yamna genetic ancestry, admixed with steppe Maykop.

ZV/III-C monuments are considered by some archaeologists in the context of the "Gordinești-Late Maykop phenomenon", in which six groups are distinguished: I - Bursuceni (Carpatho-Dniester region), II - Zhyvotylivka (Dnipro region), III – Volchans'k (Azov region), IV - Crimea (Crimean Peninsula), V - Lower Don, VI - Kuban (North Caucasus)[67]. Other ZV/III-C group subdivisions have also been proposed[64]. Thus, the ZV/III-C territory encompasses the entire North Pontic steppe and overlaps with the initial expansion of the Yamna archaeological complex.

The profound impact of Maykop on Late Trypillia, Usatove, and ZV/III-C groups resonates throughout various facets of their existence, evident in an array of archaeological discoveries from the late-4th millennium NPR steppe and forest-steppe. Notably, typical Maykop artifacts, such as metal implements like socketed axes and flat-handled daggers, alongside flint artifacts like diamond (trapezoid)-shaped arrowheads, asymmetrical with one shortened spiked and flag-shaped, or symmetrical/asymmetrical dart tips/knives, showcase this influence. The archaeological findings also include woodworking tools like adzes with asymmetrical blades and grooved chisels, jewelry like pendant rings made of silver, gold, or copper with one, one and a half, or several turns, as well as staff-shaped pins made from various metals or bone. Moreover, unique ceramics, distinguished by their shapes and ornamentation techniques, further attest to the far-reaching influence of Maykop on these archaeological groups[68].

Some scholars suggested the appearance of burial monuments displaying the III-C type features, which also include a specially designated place for the burial ritual involving fire and sacrificial offerings as reflecting the emergence of an institution of social, ideological and trade leadership in the NPR[67].

The highly mobile bearers of ZV/III-C traditions were in contact with communities interspersed throughout the Ponto-Caspian steppe. The system of relations, built on kinship and socio-economic ties, was accompanied by the reception and transfer of information and new technologies, thus facilitating the formation of a unified system, in which late Trypillia and Maykop might have acted as catalysts for the creation of the cultural and ideological basis for the emergence of a new socio-economic system manifested by the Yamna.

In the second half of the 4th millennium BCE, late Trypillian groups such as Gordinești in the middle Dniester (3400-3100 BCE) show an increase of the CHG-rich "steppe ancestry" component ([69], this report), reflecting an end of Trypillian resilience to the eastern admixture. Meanwhile, the emergence of highly mobile steppe groups such as those that left ZV/III-C type burials, connected the Carpatho-Dniester region with the North Caucasus and both became linked to a vast steppe area, where the process of synthesis and integration of diverse cultural formations took place, culminating in the emergence of the Yamna phenomenon.

1.2.1. Northwest Pontic coast in the 4th millennium BCE

In the early Eneolithic, Gumelniţa-Karanovo VI-Kodžadermen (GKK) archaeological complex and the related Varna archaeological group developed from the preceding Neolithic farming communities of the Balkans. Both GKK and Varna show a decline starting around 4200 BCE. The upper layers of GKK tells contain evidence of destruction. Scholars attributed this destruction to internal conflicts as well as conquest by the steppe groups from the north Pontic[54,56]. As the west Pontic part of the Balkan Peninsula became depopulated, settlements connected with GKK begin to appear in the Danube Delta and further northeast[54]. Such as, the Kartal archaeological complex on the Danube contains layers of GKK followed by Cernavodă I. In the Dniester Delta, a population center of Mayaky associated with the Usatove archaeological group and displaying influences from Cernavodă I (the Khadzhider type), Trypillia, and steppe Eneolithic, becomes established in the 4th millennium BCE[70,71]. The Cernavodă I/ Khadzhider population at Kartal displays a genetic continuum from GKK (Kartal B) to steppe (Kartal A). Kartal A genetically overlaps with the Usatove population from Mayaky and Usatove-Velykyj Kuyalnik[72].

The Usatove archaeological group stands out for its unique ceramic style, impressive burial structures, and established metalworking practices. Concentrated primarily within the Dniester-Danube interfluve in Ukraine, approximately 35 sites bearing Usatove characteristics have been identified [59], although the total count may be as high as 100 [73]. Notably, the largest Usatove sites in Ukraine—Usatove-Velykyj Kuyalnik and Mayaky—comprise both kurgan and ground cemeteries. The Mayaky site, in particular, features an elaborate system of ditches, variously interpreted by scholars as a sanctuary, a hillfort ("*horodyshche*"), or a causewayed camp[74].

Usatove can be categorized as "Steppe Trypillia"[51], reflecting material culture influences of Trypillia and the Eneolithic steppe groups [11,12,51,54,59,70,74–76]. Usatove is considered synchronous with the CII period of the Trypillia, which corresponds to *ca.* 3550-2750 BCE [6,77], marking the transition from the Eneolithic to the Bronze Age in the NPR chronology. Steppe influences on Usatove include pottery imports of Eneolithic steppe origin, the steppe-derived elements in Usatove-specific pottery, and signs of social hierarchy in burials attributed to Usatove [78–80]. Individual finds or imitations of Usatove sculpture are known from the Crimea and the North Caucasus. Conversely, ceramic and metal finds from the Usatove graves at Usatove-Velykyj Kuyalnik display Maykop influences[81]. The presence of Yamna burials at Usatove culture sites in northwest Pontic[12] as well as burials corresponding to the Usatove canon and containing Usatove ceramics and figurines in Yamna burial mounds on the east bank of the Dnipro from the rapids to the river delta and in the Azov region[81] indicates potential interactions between late Usatove and Yamna.

Burial mounds of the Usatove elite are distinguished by complex funeral structures such as cromlechs, stone shells, anthropomorphic steles, ditches, cult pits with pots and animal bones, accompanying the burials of people and animals. Among the inventory there are often items of weapons and/or insignia of power. However, such a common type of a weapon as a bow and arrow is practically not represented in the Usatove complexes. Apart from isolated finds in kurgans and mixed layers of the settlements of Usatove-Velykyj Kuyalnik and Mayaky, bifacial triangular arrowheads characteristic of the Eneolithic–Early Bronze Age were found in only two burials. In both cases, their location indicates that they were lodged within the body.

Among the burial goods of Usatove, the uncharacteristic for the Northern Black Sea region of the Early Bronze Age finds such as microlithic trapezoids, have been reported.

Analysis of the context of microlith finds in burials led to the conclusion that they were transversal (cross-blade) arrowheads[82]. These arrowheads, as well as other items of material culture, link Usatove with the eastern Mediterranean and Mesopotamia[54] (further discussed in Section 1.2.3).

1.2.2. Diet isotopes, Reservoir Effect, and the Usatove chronology

Using comparative analysis of ceramic imports from securely dated periods and backed by radiometric radiocarbon dating on charcoal, pottery, and animal bone from Usatove sites, the current chronology of the Usatove archaeological group aligns with the period approximately ranging from 3650 to 2700 BCE.[12,71,76]. However, dates as early as the end of the 6th millennium BCE and as late as the last quarter of the 3rd millennium BCE have been reported for Usatove sites as well [12,76]. Radiocarbon dates on human remains from the Mayaky necropolis assigned to the Usatove period obtained by Accelerator Mass Spectrometry (AMS) do not align with radiometric dates from charcoal and pottery from the same site [5,12,71]. The discrepancy was attributed to radiocarbon reservoir effect (RE), inferred from elevated ratio of stable isotopes of nitrogen [5,12,71]. High $\delta^{15}N$ ratios accompany aquatic-resource-based diet, leading to a reservoir offset (RO) (e.g.,[15,83–89]).

The RO at the Mayaky site has previously been quantified using ROs from Meso-Neolithic sites in the middle and lower Dnipro Valley, based on the assumption that radiocarbon dates obtained on human remains of populations living along the same drainage basin and consuming foods largely derived from freshwater resources will be under the influence of comparable ROs[5]. Archaeological reports indicate a significant presence of remains of aquatic animals at the Mayaky and Usatove-Velykyi Kuialnyk sites. For Mayaky, this evidence is summarized in[71]. At Usatove-Velykyi Kuialnyk, it was determined that fishing in the Khadzhybey Estuary, but also the Black Sea, occupied a prominent place in the daily activities of its inhabitants. The Khadzhybey Estuary was significantly desalinated by rivers, making it habitable for catfish, sturgeon and other fish, the remains of which have been found at the Usatove-Velykyi Kuialnyk site. Stone sinkers were found on the territory of the Usatove-Velykyi Kuialnyk site, while bone fishhooks were found in the kurgans, suggesting that fish were caught using nets and fishing rods. Large quantities of mollusk shells were also found at Usatove-Velykyi Kuialnyk, dominated by *Mytilus* and *Cardium (Cerastoderma) edule* saltwater bivalves[80(p.148)]. Conversely, comparing diet isotopes values of Mayaky humans and local contemporaneous domestic fauna led to a conclusion that dietary input of the Eneolithic residents of Mayaky from terrestrial meat was minimal[71].

Reservoir age offsets in the coastal northern Black Sea region fluctuated in the 340-1110 ^{14}C years range during the Holocene[90]. The reservoir age of the top water layer of the Black Sea after the Mediterranean inflow of ca. 8300 BP/6350 BCE is estimated to be about 400 ^{14}C years[91]. A comparable 440±45 ^{14}C years offset was used in[92] (Method 2) to estimate a RO on ^{14}C dates from human remains and contextual fauna in the Iron Gates area on the Danube, by considering the $\delta^{15}N$ ratios of above 13‰ to reflect 100% aquatic diet, and ratios in the 10-13‰ range to reflect 50% aquatic diet, with a corresponding RO of 220±23 ^{14}C years. Comparing stable isotopes of carbon and nitrogen from the Iron Gates area (N=140 [8,93,94]) with those from the Eneolithic Mayaky samples attributed to Usatove (N=16 [5,12,71], this report) reveals that they are within the same distribution (2-tailed Mann-Whitney test p=0.01928, z-score=2.34494 for

$\delta^{13}C$, p= 0.06724, *z*-score=-1.83128 for $\delta^{15}N$, significance level 0.01). At the same time, diet isotopes from Mayaky are outside of diet isotope distribution of fisher-forager-hunter populations of the Dnipro Valley[16]. Applying the RO Method 2 calculations from[92] to Usatove-designated samples from Mayaky dating to the 6th-5th millennium BP that produced genome-wide aDNA data (I12704, I12710, I12707, I12706, I1729, I1423 (I12705)), we obtained RE-calibrated dates in the 3792-3514 calBCE range (individual RE-adjusted dates are presented in Table SI1 1). The Mayaky RE-calibrated range including Eneolithic Mayaky specimens from[72] (assuming $\delta^{15}N$ ratios compatible to the Mayaky specimens in the current report) and[5] is 3813-3389 calBCE. The RE-adjusted chronological range for human remains thus overlaps with the 3650-2740 BCE radiometric date range from pottery, charcoal and animal bone from Mayaky, obtained by the scintillation ^{14}C technique[71]. The difference between the two ranges is likely due to a combination of factors such as the presence of a large error in radiometric dates and the origin of the bulk of the material for radiometric analysis from sanctuary ditches at Mayaky, potentially containing inclusions of artefacts outside of the Usatove cultural sphere[76].

The RO-adjusted chronological range for Usatove, as determined from samples collected from Mayaky, indicates that the Usatove population emerged within three generations after the steppe hiatus period, persisting until the onset of the Yamna expansion. The upper chronological boundary of the RO-adjusted date range, combined with the reconstruction of the expansion waves of the CLV people presented in this report, raises a possibility that the people of the first CLV wave into the NPR did not disappear by the end of the steppe hiatus, but became the people of the Usatove archaeological group, after interacting with the neighboring Trypillia for over half a millennium and receiving 50% of ancestry from them. The presence of artefacts of the Giurgiuleşti type at the Usatove site of Mayaky[76] corroborates the transformation hypothesis of CLV migrants into Usatove in northwest NPR. Trypillians, on the other hand, were less genetically affected by this interaction. While some Trypillian individuals with elevated CLV ancestry dated to the first half of the 4th millennium BCE like I7923 may have retained a genetic signature of these early interactions with CLV migrants, in those CLV-rich Trypillians dated towards the end of the 4th millennium BCE such as I20069, it is likely a result of interactions between Trypillia and Usatove or CLV-derived steppe people such as the early Yamna.

1.2.3. Contacts between the NPR, the Caucasus, and the Near East

Archaeological evidence suggests contact between the populations of the North Pontic, the Near East, and the Caucasus, beginning in the Neolithic.

The shape of bracelets found at the Neolithic Mariupol Necropolis shows analogies with finds from the early burial phase at the Nalchik Necropolis in the North Caucasus[1]. The style of Mariupol mace heads and bracelets was further suggested to originate from Mesopotamia[1,95]. Shell beads found at the Mariupol Necropolis show analogies with the material from the Neolithic burials at Kaylu in the southeastern Caspian, which, correspondently, are similar to those from the Neolithic sites in the southwestern Caspian Sea area[96]. Early Serednii Stih ceramics display analogies with pre-Maykop ceramics of Svobodnoe, pointing to the existence of trade/exchange relationship between the Serednii Stih of the Dnipro Valley and populations of the North Caucasus Piedmont[97].

Mesopotamian influences on Trypillian plastics and ornaments on pottery are evident on artifacts dated to ca. 4100-3500 BCE[98]. Images on Trypillian ceramics of figures in dresses with

fringe-trim edges[99], also found on Sumerian dress depictions (Fig. SI1.2), appear after 3500 BCE.

Individuals I17973 from Bursuceni and I20079 from Taraclia were interred with staff-shaped pins made of bone. Several late Trypillia, Usatove and ZV/III-C burials contain similar pins[68,100], often accompanied by beads[64,67], like in the case of Individual I20079. Stylistically similar pins made of gold, silver and copper alloy and accompanied by suspended beads and cylinder seals (Fig. SI1.2) have been uncovered at the Early Metal Age sites in Mesopotamia such as the royal Early Dynastic period cemetery at Ur[101,102]. Copper beads resembling Mesopotamian cylinder seals are extensively found in late Trypillian metal hoards[103]. It is conceivable that the hammer-top pins of the Yamna (Fig. SI1.3), sometimes found in association with strung beads[104] were patterned after the Mesopotamian pins and symbolized a particular social status.

Burials of the III-C type are often accompanied by glazed and pierced Maykop-style pottery. The appearance of pottery with pierced ornaments in Maykop ceramics is considered to be an influence of southern Mesopotamian Uruk tradition. In eastern and southeastern Anatolia, northern and western Syria, ceramics with this type of ornament remained in use during the Late Chalcolithic IIb - EBA IA period, or approximately the second half of the 4th millennium BCE. North of Mesopotamia, similar pottery has been found in significant quantities at settlements along the Upper Euphrates.

Another group of finds potentially linking the Eneolithic NPR to the Near East are trapezoid flint microliths. Traceology analysis of these artefacts from the Usatove culture monuments revealed they had a multifunctional purpose, serving as compound sickle and knife components and, possibly, as arrowheads. Many of these, however, did not show any signs of utilization, suggesting that they had a ritual or ceremonial purpose[105]. The association of such microliths with elite Usatove graves such as burial 18/1 at the Usatove site of Mayaky[106] strengthens the latter assertion. In at least three elite Usatove burials trapezoid transversal microliths were found in compact clusters and single trapezoid microliths were found in close association with human remains[82]. Transversal flint microliths, along with a staff-like pin, were found in a III-C type burial 28 of the Slobodzeya Kurgan 1[107]. A microlithic trapezium was also found in the main burial (#18) attributed to the Yamna archaeological complex at Glinoe-DOT Kurgan 1, extending the presence of such microliths in the NPR into the EBA. The calcified trapezium was located close to the femur, suggesting its association with the remains to be a result of a healed injury[82].

The use of arrows with transversal microliths as tips is known in Egypt of the predynastic period, the Old and Middle Kingdoms, traced both from images and finds of arrows in tombs, as well as images of arrows with transversal tips on reliefs and cylinder seals from Uruk (Sumer, late Uruk period, last quarter of the 4th millennium BCE) and Knossos (Crete)[82]. It is noteworthy that the use of microlithic transversal arrowheads in the NPR ceased almost 1500 years before the appearance of the Usatove archaeological group[82]. Late Trypillia as well as steppe pastoralists of the NPR used bifacial triangular points. At the same time, a trapezoid microlith has been found at the Trypillian megasite of Nebelivka[108].

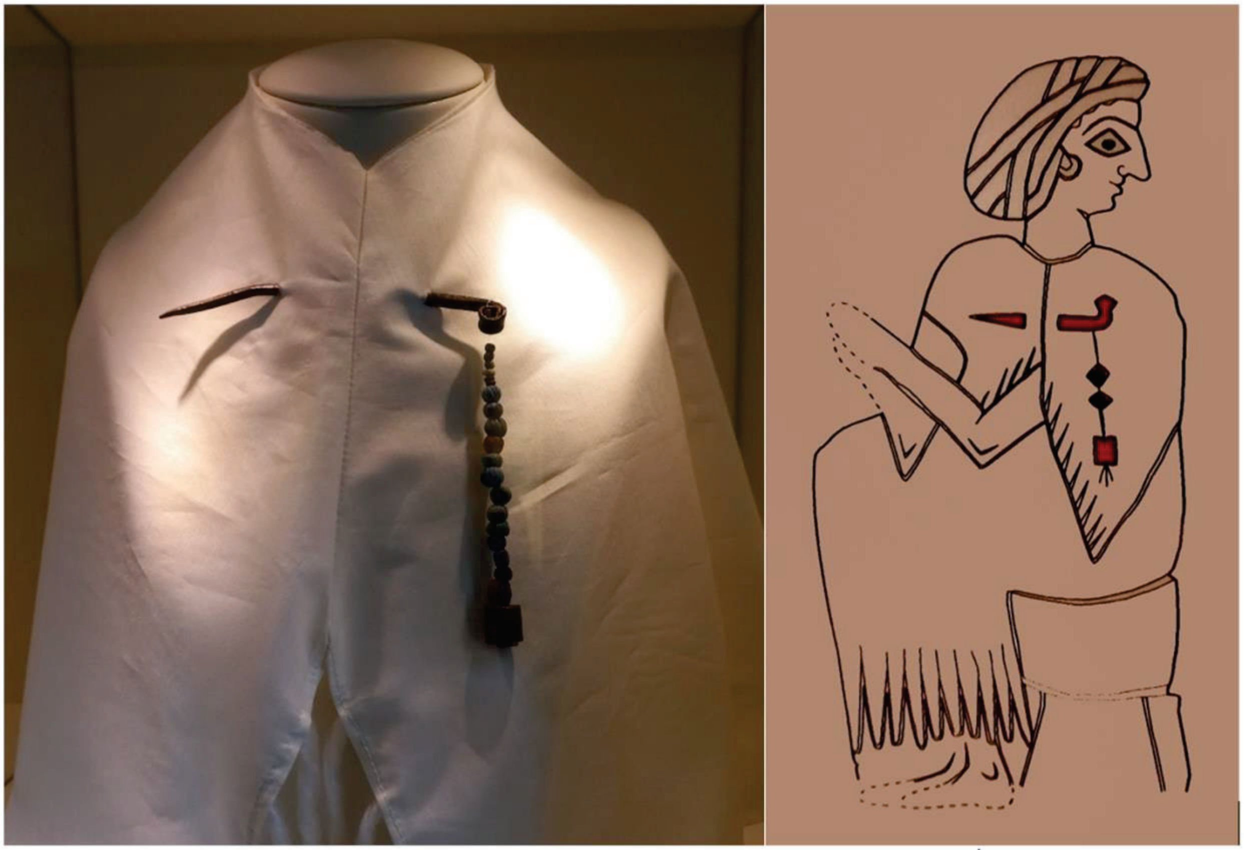

Figure SI1. 2. A cloak pin with beads and cylinder seals suspended from it. Uruk, 4th millennium BCE. Neues Museum, Berlin. Photo: M. Y. Videiko, 2023.

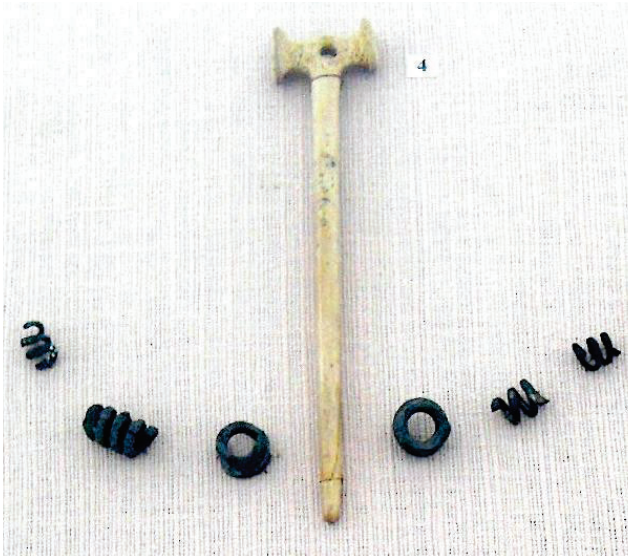

Figure SI1. 3. Hammer-top pin of the Yamna archeological complex. Stari Bilyari Kurgan 1 Burial 4. Photo: S. Ivanova, undated.

1.3. The Early Bronze Age (3300/3000-2400 BCE)

1.3.1. Yamna Archaeological Complex

In the last third of the 4th millennium BCE, the transition to the EBA in the Pontic steppe is marked by the appearance and rapid spread of the traditions of the Yamna archaeological complex. At their maximum extent, the Yamna groups occupied an area from the middle Danube and the northern slopes of the Balkans in the west to Ural Mountains in the east. Several archaeological groups are variably recognized by archaeologists throughout the Yamna domain. In the NPR, these include the Donetsk, Middle Dnipro (Northwestern), Southwestern, Lower Dnipro, Azov-Crimean and Southern Buh groups, with additional divisions proposed within each region. The differences between Yamna regional groups are observed primarily in the material culture, especially, in ceramics and its typology. At the same time, the regional divisions within the Yamna complex that were established by the degree of similarity of ceramics often do not coincide with traditional local variants. Furthermore, comparisons of published ceramic series from individual Yamna regions often show more differences than similarities[109].

Yamna is known predominantly from burial monuments and funeral rites; settlements are rare. In fact, the Yamna archaeological complex is defined primarily by burial traditions, rather than characteristics of the material complex, as it is traditionally done in archaeology, where ceramics provides the most distinguishing features[109].

Yamna burials utilized kurgans constructed in the Eneolithic, but the Yamna also erected kurgans of their own. In the lower Don, there were more than 300 Eneolithic-EBA kurgans and 1,500 Yamna burials excavated by the end of the 20th century[110]. In Romanian Transylvania, the number of the Yamna-age kurgans is estimated to be over 600[111]. As of 2020, 177 kurgans containing 714 Yamna burials have been investigated in Romania. There is information of about 75 investigated Yamna kurgans in Hungary, and about 15 Yamna kurgans in Serbia. According to the latest publications, 80 kurgans containing 460 Yamna burials have been excavated in Bulgaria[109]. The vast majority of Yamna kurgans are located along the river valleys of the Ponto-Caspian steppe, with their highest concentration in the northwest Pontic[112–114].

As of the late 1980s, the number of excavated Yamna burials in Ukraine exceeded 10,000 (an estimate by M. Y. Videiko). Just between the Prut and Dniester rivers 6290 kurgans are known[115] and 10000 are estimated, with over 50% erected during the Eneolithic-EBA[113], of which over 700 kurgans and over 5000 burials have been excavated and published. The highest density of Yamna graves is documented in the Southern Buh area, where more than 5800 kurgans are estimated to have existed in the pre-industrial era[114], while the lowest burial density is found in the Lower Don region. The Yamna burial density as well as the number of stratigraphic phases attributed to the Yamna period pronouncedly decreases towards the Volga-Ural periphery of the Yamna domain[112].

Yamna kurgans typically exhibit a complex stratigraphy. In the mounds constructed above the primary (main) burial, additional (inlet) burials were subsequently added. Larger kurgans can contain several subsequent mounds or local fills associated with a group of burials or a specific complex. Burials within each mound layer, especially if numerous, are arranged in arcs or circles, possibly linked to ancient beliefs about celestial body movements[116]. Elements of mound architecture include ditches, cromlechs, and retaining walls. Burial chambers, mostly rectangular, consist of simple ground graves and some have ledges. Some grave pits have ceilings made of logs or stone slabs. Anthropomorphic steles are occasionally found as part of

the stone ceiling, and wooden carts or their parts being rare elements in the funeral ritual. One Yamna burial in the northwest NPR dated to 3315-2916 calBCE contained a stylized boat made of wooden planks as part of the funerary installation[117].

The position of the deceased in Yamna graves varies considerably. Some researchers identify five main types based on the position of the arms, legs, head, and body inclination, with each type having numerous subvariants[118]. These five types include (1) flexed on the back with bent legs (knees up), arms extended along the body or slightly bent, hands on hips, sometimes legs arranged in a diamond shape (57.2% of the burials); (2) tilted to the right, with left arm bent at the elbow, hand near the pelvis, abdomen or chest; the right arm extending along the body (16.3%); (3) tilted to the left, the right hand is located at the pelvis (13.1%); (4) on the right side, with different positions of the hands (7.3%); (5) on the left side, with different positions of the arms (6.1%). Some researchers propose three (back, the right or left side), or two groups (contracted on the back and contracted on the side)[73,113].

Analyzing main Yamna burials reveals a predominance of the flexed on the back group, possibly indicating social stratification[119]. Some argue that the flexed on the back position characterizes an early chronological stage, while flexed on the side to be the feature of a late stage[118,120,121]. The supporters of the idea of correlating burial position with chronology emphasize that the flexed on the side position mostly present in inlet burials. Yet, when planography data and stratigraphic layers of the Yamna kurgans are considered, the simultaneous occurrence of various positions within one horizon becomes evident[122]. Such as, 95% of multi-layer Yamna kurgans in northwest NPR contain horizons with mixed ritual traditions.

The funeral tradition in the EBA became more unified across the Yamna territory, compared to burial practices of steppe populations from the preceding chronological period. At the same time, differences, primarily in ceramics, as well as in the number and assortment of metal artifacts, persisted among different Yamna groups. This was due, on the one hand, to different Eneolithic substrates in different territories of the Yamna, and on the other, to cultural contacts.

One notable example of regional Yamna specifics is the Bugeac archaeological group in northwest NPR. Bugeac ceramics, unlike in other Yamna regions, have a flat bottom. Some of the vessels demonstrate the influence of archaeological complexes and groups of the Balkan-Carpathian area and Central Europe (Corded Ware, Globular Amphora, Cernavodă II, Kotsofeni, Glina III-Schneckenberg, etc.), and some are imports, while other vessel forms are characteristic only of the Bugeac ceramic complex. Additionally, in contrast to synchronous archeological groups of the North Pontic and the greater Carpatho-Balkan area, a notable concentration of silver jewelry is observed in the Bugeac region in the Usatove and Yamna Bugeac archaeological contexts.

The distinctive funeral rituals of the Bugeac Yamna group are characterized by a kurgan, featuring the main burial at its center in a rectangular burial chamber covered with either wooden or stone slab ceilings (one-third of the burials incorporated ledges), a plant mat beneath the body, and the use of ochre. The deceased is often placed in a contracted position on their back or side, with a prevailing western orientation. Inlet burials are often arranged in a circular formation around the main burial, and intricate mounds with multiple stratigraphic horizons are occasionally present. These elements, observed in diverse forms and combinations, are shared with Late Eneolithic groups across the region, amalgamated into a cohesive complex within the Bugeac group.

Cultural exchanges between the Bugeac Yamna and populations in the Danube region are evident in the ornamentation of pottery, as well as the introduction of askos-type vessels and cups. Furthermore, in the northern part of the Bugeac group territory, the influence of the Globular Amphora archaeological complex is discernible through the import and imitation of ceramics. It has been suggested that the people of Globular Amphora archaeological culture and those of the Yamna (possibly the Bugeac group) played a role in transmitting the ceramic traditions of Usatove to the Złota group within the Corded Ware archaeological complex[123]. Occasional imports from the Balkan-Carpathian area can be traced in Yamna groups further to the east, such as at Sofyivka 10/1, the Kherson region, and the western bank of the Dnipro[124].

Ceramics such as beakers and amphorae, reflecting connections with the Corded Ware archaeological complex, are distributed throughout the northwest Pontic, concentrating along the Dniester and in the south of the steppe zone. Amphoras are often imported, while beakers are imitations, their ornamentation is different from the classical canons of CWC and distorted. It is conceivable that the Bugeac population was responsible for transferring the Corded Ware features to the west and east, evidenced by a beaker with a corded ornament found in the Crimea (Istochnoe 12/5)[125] as well as beakers of the same type from Romania, Bulgaria and Hungary. The discovery in 2023 of a similar beaker near the village of Orlivka, located at a ford on the Danube, is likely part of the same transfer process (the authors are grateful to Dr. Igor Bruyako for access to the information about the Orlivka excavations).

1.3.2. Catacomb Archaeological Complex

Around 2700 BCE, the Catacomb Archaeological Complex emerged in the north-eastern Azov and the lower Don region[126] and later expanded across a significant part of Ukraine. Researchers date the entire existence of the Catacomb to 2700–2000 BCE, with the early stage, marking the formation of Catacomb groups, occurring between 2700–2500 BCE[127]. Excavations in the 1890-1990s uncovered over 1200 Catacomb burials.

West of the Ingulets River, only isolated burials from the early stage of the Catacomb are known[128]. The Catacomb tribes' settlement involved interactions with the local, predominantly Yamna population, evident in the material culture inventory[126,129]. Even in its early stage, the Catacomb lacked unity, exhibiting territorial features that later led to the formation of distinct Catacomb groups during the developed stage (2500–2300 BC), characterized by relatively stable sets of characteristics[127].

The Catacomb is comprised of several regional groups characterized by different burial rites, such as the Donetsk and Ingul groups in the Pontic steppe. The Donetsk group features a flexed and tilted-to-the-right body position in graves, while the Ingul group is characterized by an extended supine position. The catacomb shapes and ceramic complexes also differ between these groups.

The westward expansion of the Catacomb groups reached beyond the Danube, expanding locally over the territories not already settled by the Yamna. Radiocarbon dates from a small sample of Catacomb burials in northwest Pontic align with the ca. 2900-2000 BCE range. The Catacomb' final stage in the NPR coincides with the initial stage of the Babyne cultural circle.

The genetic ancestry of most Catacomb individuals from Ukraine examined to date is consistent with being derived from the Yamna (Table 1). At the same time, the ancestry of a Catacomb individual I11850 from the Odesa kurgan is best modeled as PVgroup+Trypillia (Supplementary Information, section 2).

A low-coverage Catacomb Individual I16668 from Revova stands alone on the PCA in Fig 2a, leaning towards the main Usatove cluster. This position is intriguing, as the main burial №19 in the Revova Kurgan 3 is of the Usatove genetic ancestry, suggesting a genetic ancestry continuum persisting through a change in cultural traditions.

1.4. The Middle Bronze Age (2400-2000 BCE)

The Middle Bronze Age in south-eastern Europe was marked by drastic climatic shifts, likely leading to the disintegration of the Catacomb cultural complex and the formation of a number of new archaeological units in its place.

Among the post-Catacomb formations, the Babyne archaeological circle, which covers most of the NPR area, stands out for its scale and a series of distinguishing features. The formation and development of the Babyne cultural region affected the content and direction of cultural and historical processes in south-eastern Europe during the entire transition period from the Middle to the Late Bronze Age.

The Babyne circle includes the homogenous Dnipro-Don and the more fragmentary Dnipro-Prut archaeological groups. Within the latter, Dnipro-Dniester, Dnipro-Buh (forest-steppe), and Dniester-Prut (steppe) regional subgroups are distinguished. Babyne burial sites are represented by 1140 burials from 592 kurgan and 8 ground (kurgan-less) cemeteries, containing predominantly contracted on the side inhumations. A qualitatively new stage of kurgan construction in the Black Sea region is associated with the Dnipro-Don Babyne, marked by a rapid increase in scale and innovations in architecture, which, sometimes, included three to six Babyne building horizons. Furthermore, 79% of Babyne burials were accompanied by grave embankments and backfill. The Dnipro-Don group started the practice of erecting long graves by lengthening and unifying the mounds. A striking feature of the Babyne kurgan architecture in the Donetsk region is the presence of a variety of megalithic elements. Around 270 settlements are associated with the Dnipro-Don Babyne archaeological group[130].

The Dnipro-Prut Babyne group stretched from the Dnipro Valley and the Crimea in the east to the west bank of the Prut and the lower Danube in the west. The Dnipro-Prut group is represented by about 170 settlements, up to 2500 burials, and several treasure hoards, potentially associated with Babyne[130].

A considerable part of Babyne material culture features, having no local roots, finds striking correspondences in the populations of the Bronze-Age Caucasus and the Carpatho-Danubian region, implying transregional culture synthesis to be part of Babyne's cultural identity. Material culture parallels with the NPR Babyne are recognized in Bronze Age populations of lower Don and Northern Caucasus. The Bronze Age Don-Volga Abashevo, Syntashta, and Potapovo "chariot culture" groups are partially synchronous with the Babyno circle, based on the relative stratigraphy, as well as analogies in the material culture. To the west, in the Transcarpathia and the Middle-Upper Danube, the Middle Rhine, and the Alps, post-CWC/Beaker groups such as Mierzanowice, Polada, and Únětice demonstrate a complex of analogies in funeral rite and material complex with groups of the Babyne circle. In the basin of the Middle-Lower Danube, several archaeological groups of the Middle Bronze Age of Hungary and the Early Bronze Age of Romania appear to be simultaneous with different stages of Babyne. The chronological successor of Babyne in the NPR is the Sabatynivka-Noua archaeological complex[130].

1.5. Flat-top kurgan sanctuaries in the NPR and the origin of Yamna

Kurgans stand out as hallmark features of the Yamna archaeological complex. Most of the Yamna kurgans still standing exhibit a rounded shape, although a substantial number also display a conical form. The origins of conical kurgan mounds date back to the Eneolithic period. Throughout the Yamna period, truncated conical mounds with flat horizontal upper platforms were likely common in the Ukrainian steppe zone, though accurately estimating their prevalence remains difficult[104]. The compacted upper platforms of some of such kurgans in the NPR are usually coated with a thick layer of clay. In the northwest NPR, these include such kurgans as Bakumova Mohyla near Mayaky with a clay horizontal platform 10 m in diameter, and Kurgan Katarzhyno 1 with a trapezoid planform of yellow clay. In the Dnipro Rapids area, Kurgan 22 near the village of Hlukhe had a 50 cm thick clay platform layer. Bakumova Mohyla and Kurgan 22 had a height of 7 m in their finished form.

Flat-top kurgans are also known in the North Caucasus. These include Maykop kurgans such as Zamankul Kurgan 2, Klady Kurgan G and the massive Kurgan 11 that covered it, the Nalchik Kurgan, the Bolshoy Ipatovsky Kurgan (5th embarkment), etc. As in the NPR, the surface of embankment platforms of most flat-top kurgans in the Caucasus was finished with a layer of bright yellow clay coating[104].

One of the most architecturally complex flat-top kurgans in the NPR is the Shakhtar (Shevchenko) Kurgan 29. In addition to the horizontal platform at the top of the fourth embarkment, the Shakhtar Kurgan also had a ledge. The shape and structure of the embarkments of kurgans such as Shakhtar Kurgan 29 likely relate to their use not only as burial places but also as sanctuaries associated with the performance of ceremonial activities. The Lower Dnipro Valley contains a chain of kurgans with ramps and altar platforms with a pavement of turf-mud rolls, with roads radiating from them. One such kurgan is the Vynohradne Kurgan 3 located in the Molochna River Valley (Section 2, Burial Descriptions), from which came one of the closest to the Core Yamna Serednii Stih individual in Burial 15[49]. An estimated 46 sanctuaries like Vynohradne Kurgan 3 with radially extending roadways are concentrated in the Dnipropetrovsk and Kherson regions of southern Ukraine, along the lower stretches of the Dnipro and Ingulets rivers[131].

A particular dense concentration of flat-top kurgan sanctuaries, many with radiating roads still visible in the landscape, can be found within ca. 100-km radius from the Eneolithic-EBA Mykhailivka and Kapulivka sites, the latter featuring Mykhailivka-type early Yamna ceramics[132] (Fig. SI1.1.4). On the Ingulets River, kurgan sanctuaries extend from a ford at Davydiv Brid upstream to Kryvjy Rih, and from a ford at Kakhovka on the Dnipro River upstream to the city of Dnipro. A flat-top kurgan near Pishchanka at the northern edge of the Dnipro River kurgan chain contains an overlapping sequence of Serednii Stih and Yamna burials, in which the earliest Yamna burial dates to 3626-3106 calBCE[133], chronologically overlapping with the earliest Core Yamna individual I32534 from Mykhailivka, presented in this report. The Pishchanka Kurgan is also in the vicinity of Orgin-8, from which one other closest to the Core Yamna Serednii Stih individual originates[49]. A line of sanctuaries with radially extending roads or erected at branching points of dried-up river valleys such as Forois (Zhovtneve) Kurgan 11 in the Tokmak District extends from the main concentration in the Dnipro-Ingulets interfluve northeast towards the Siverskyi Donets River. In the west, flat-top kurgan sanctuaries are found along the Danube and Dniester estuaries. The northernmost kurgan sanctuary is located at the forest – forest-steppe boundary ca. 400 km north of Mykhailivka (Fig. SI1.1.4).

Taken together, the flat-top kurgan sanctuaries found in southern Ukraine encapsulate a continuous archaeological record spanning from the Serednii Stih era to the early Yamna period, as well as establishing a connection with Maykop kurgans in the North Caucasus. Notably, these kurgans harbor the earliest chronologically dated Yamna individuals in the NPR. These complex architectural structures likely served a polyfunctional purpose, acting as both burial grounds and venues for communal ceremonial rituals. This ceremonial function, extending beyond mortuary practices, aligns with the notion that a fundamental aspect of the Yamna culture complex was the shared worldview embraced by its communities[54,109].

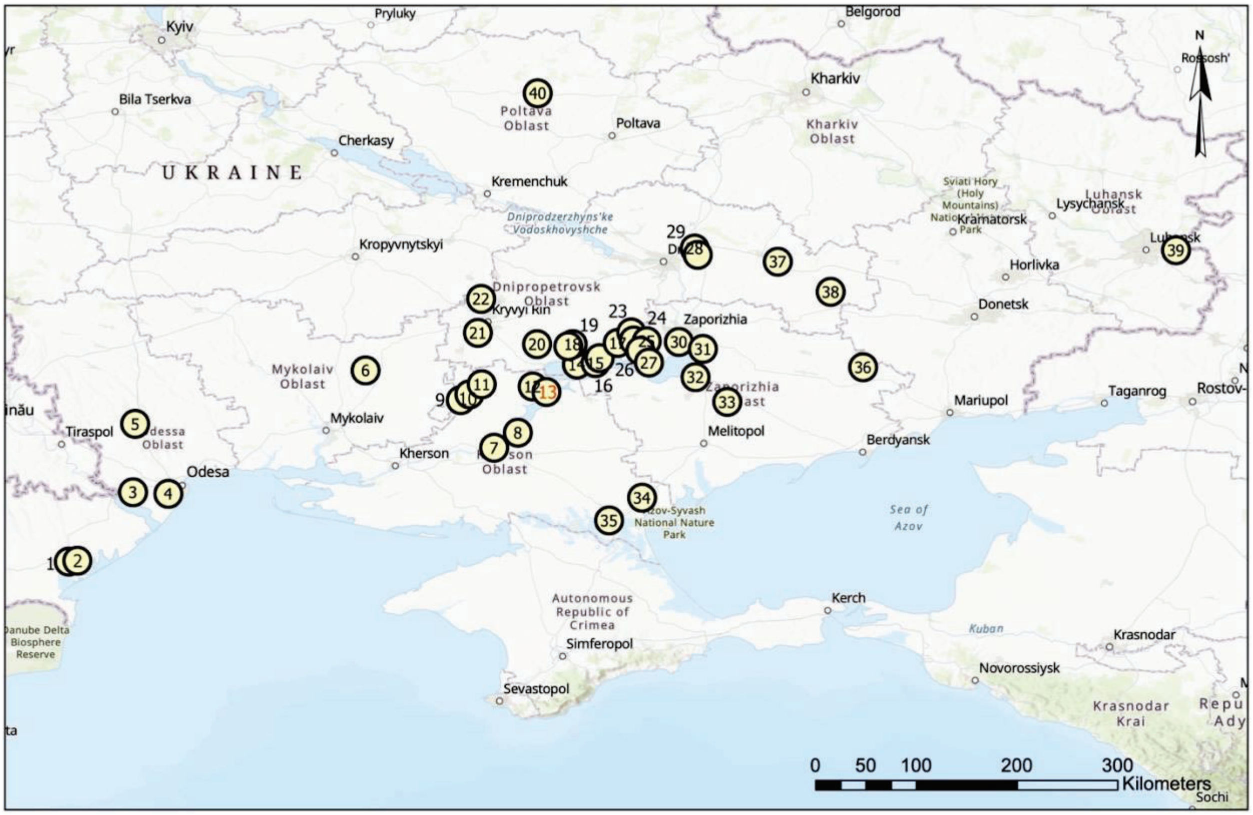

Figure SI1. 4. Flat-top kurgan sanctuaries and ancient river crossings in southern Ukraine.

Map was created using ArcGIS Pro v 3.1 (Environmental Systems Research Institute, Redlands, California, USA). Basemap credits: Esri, TomTom, Garmin, FAO, NOAA, USGS, Esri, USGS.
1, Trapivka; 2, Vyshneve; 3, Mayaky (Bakumova Mohyla); 4, Nova Dolyna; 5, Katarzhyno (Znamianka); 6, Starohorozhene; 7, Kakhovka (Dnipro River crossing); 8, Kairy; 9, Davydiv Brid (Ingulets River crossing and a kurgan); 10, Mala Oleksandrivka; 11, Starosillya (Velyka Mohyla); 12, Shevchenkivka (Kurgan 14); 13, Mykhailivka; 14, Kapulivka; 15, Nikopol; 16, Kam'yanske; 17, Borysivka; 18, Shakhtar (Kurgan 29); 19, Hirnyts'ke; 20, Kam'yanka; 21, Kryvyi Rih (Ryadovi Mohyly); 22, Nedaivoda (kurgan group "Three brothers"); 23, Kyslychuvata (Hola Mohyla); 24, Myrove; 25; Vysoke; 26, Hlukhe; 27, Vyshchetarasivka; 28, Pishchanka; 29, Sokolove; 30, Rozymivka; 31, Hryhorivka; 32, Vasylivka (Kurgan 1); 33, Vynohradne (Kurgan 3); 34, Hordienkivtsi; 35, Vesnyanka; 36 Forois (Zhovtneve Kurgan 11); 37, Bohuslav; 38, Volodymirivka (Kurgan 13); 39, Sykhodil (Pioners'ke); 40, Solontsi (Pidluzhne).

2. Burial descriptions

Written by Alexey G. Nikitin with contributions from Svitlana Ivanova, Sylwia Łukasik, and Henry Shephard

Bulgaria

(Contact: D. Reich)

Durankulak Kurgan F

Durankulak, Tolbukhin district, Southern Dobruja Region (43.693, 28.527)

The cemetery of Durankulak is the largest Late Neolithic-Early Bronze Age cemetery in the Pontic region, containing 1204 burials. In 1980, 39 prehistoric graves (burials 10-49), including complexes with remains of a funeral meal, were uncovered on the border between loess and black earth[26]. One of these complexes was kurgan F, containing burials 12, 15, and 29. Kurgan F was one of seven kurgans at the Durankulak cemetery. The 17 burials from the Durankulak kurgans document the earliest presence in Southern Dobruja of a population carrying the traditions of the Usatove, Proto-Yamna, and Cernavodă I populations. These individuals represent the population that came to Dobruja from the North Pontic steppe after 4200 BCE and filled the prolonged cultural vacuum after the collapse of the Eneolithic cultural system of Varna- Kodjadermen-Gumelnița-Karanovo (KGK) VI[134].

I1456

DUR1, Kurgan F, Burial 15 (main burial), male, adult, 3700-3300 BCE

Contracted on the right side, head to north, facing east.

Golyamata Mogila (Popovo)

Popovo, Bolyarovo Municipality, Yambol Region (42.204, 26.728)

Archaeological rescue excavations of the Big Popovo kurgan 4 km south of the village of Popovo took place in 2006[31]. The kurgan had the shape of a segment of a sphere. Thirty-nine graves were discovered in the kurgan, 12 which (graves 10, 12, 18, 28, 29, 30, 31, 33, 36, 37, 38 and an empty burial pit 39) dated to the second phase of the Early Bronze Age. Four of them belonged to the Yamna complex (graves 28, 36, 37, 39). The dead were laid in the middle of a deep burial pit along its length so that the orientation of the pit was the same as the orientation of the skeletons. The skeletons were oriented in most cases in the east-west direction with the head to the west. The interred were laid on their backs, head slightly bent forward, and legs bent at the knees. The arms were extended along the body. Ochre was found most around the skull. Four of the pits had a wooden cover. On the short walls of the pits, three or four thinner beams (with a circular cross-section) were placed transversely. Six or seven flat beams were placed on top of them, longitudinally of the burial pit.

Pit 39 and grave 37 are the only structures in the kurgan that were dug into the matrix. It is possible that these pits were built together and were in some kind of relationship. After the completion of the funerary rites, the primary mound embankment was raised over them.

The remaining graves from the EBA were dug into the already existing mound embankment. These graves lack a wooden covering. The skeletons were oriented in most cases in the east-west direction with the head to the west. The position of the interred was supine, head slightly bent forward, and legs bent at the knees, which later fell in different directions. The

hands were placed on the pelvis. Red ochre was found on the skeletons, with the skull, feet, and wrists being the most heavily colored. Pieces of red ochre were mostly placed near the head and on the bottom of the burial pit.

The inventory in the graves consisted of hair spirals made of silver and gold. The spirals found in the graves from Popovo find parallels among grave goods from present-day Bulgaria, Serbia, Romania, Moldova and Ukraine, all dated to the second phase of the EBA.

I1448
POP1, Golyamata Mogila, Burial 29, female, 3050-2550 BCE

I1427
POP4, Golyamata Mogila, Burial 37, male, 3050-2550 BCE

Grave 37 was located in the northeast sector of the mound, dug into the mainland, oriented northwest-southeast. A badly crushed skull was found in it, missing the upper jaw and the entire face. A humerus without epiphyses containing traces of ochre was discovered at the southeast under the skull. Eight stake holes were found along the inner periphery of the burial pit. The presence of these holes suggests the possibility that the pit had another wooden structure before being covered with wooden beams and filled in.

Riltsi Kurgan 264
Dobrich, Southern Dobruja Region (43.592, 27.779)

The Riltsi necropolis consisted of more than 30 kurgans arranged in a semi-circle in the west suburb of Dobrich. The kurgans were one to five meters in height. Rescue excavations of Kurgan 264 in 2000 uncovered five graves, archaeologically dated to the EBA. Some of the graves studied in the Dobruja region showed parallels with the Bursuceni-Zhyvotylivka type burials north of the Danube River[32].

I1428
RIL3, Riltsi Kurgan 264, Burial 5, female, 3360-2890 BCE

Excavated by S. Alexandrov in 2000. Semi-supine inhumation to the right, oriented southwest-northeast. Red ochre was placed over the skull and pelvis. Burial inventory included a bovine bone placed near the skull. The burial pit had no covering. The burial is archaeologically attributed to the second stage of the Yamna archaeological complex [135]. Radiocarbon date estimate is based on dates from Riltsi Kurgan 264, Burial 4 (3361-3099 calBCE (4520±34 BP, SUERC- 86512) and Kurgan 260, Burial 1 (3011-2887 calBCE (4314±26 BP, SUERC-84759)[136].

Moldova

(Contact: S. Łukasik, G. Sibru, V. Dergachev)
Samples from Moldova presented in this report were obtained under the authorization of the Institute of Cultural Heritage of Moldova in 2015 and 2019.

Bursuceni Kurgan 1
Bursuceni, Sîngerei district (47.488, 28.016)

In 1977, the Eneolithic Archaeological Expedition of the Institute of History of the Academy of Sciences of Moldova directed by E. V. Yarovoy conducted research on a single kurgan on the west bank of the Middle Prut River, on the route of a dirt road under construction near the village of Bursuceni. The southern half of the mound fell under the construction of the roadway[24].

The kurgan was located on the route of the local road Koshkodeny-Sloboda Megura, 400 m west–southwest of the outskirts of the village of Bursuceni. It was situated in the center of a valley, almost in the middle of a medium-sized upland, the width of which reached 2 km. Within a few kilometers, the mound was clearly visible from anywhere in the valley.

I17973

Br/K1/B21/S1

Bursuceni Kurgan 1 Burial 21, Skeleton 1, male (Individual 5 according to the field numeration), 3354-3103 calBCE (4516±13 BP, weighted mean[10] of 4520±25 BP, PSUAMS-8310, 4530±20 BP, PSUAMS-8723, 4500±20 BP, PSUAMS-8722, this report)

I17974

Br/K1/B21/S2, Bursuceni Kurgan 1 Burial 21, Skeleton 2, female (Individual 6 according to the field numeration), 3334-3030 calBCE (4470±20 BP, PSUAMS-8724, this report)

I17975 (S20195)

Br/K1/B21/S3, Bursuceni Kurgan 1 burial 21, Skeleton 3, female (Individual 8 according to the field numeration), 3334-3030 calBCE (4470±20 BP, PSUAMS-8724 based on the date from the I17974 twin).

Burial 21 was located in the center of the kurgan, at a depth of 3.9 m from the ground and 3.7 m from the surface of the mound. The burial chamber was of irregular rectangular shape with uneven walls and rounded corners, 1.75 m long, 1.4–1.55 m wide, and 0.6 m deep from the ledge, oriented along the line north-north-east - south-south-west. Its southern and eastern walls were sloping to the bottom by 0.05–0.08 m. At the level of the ledge, the pit had a transverse wooden ceiling of nine oak logs with a diameter of 0.18–0.2 to 0.35 m and a length of 1.65 to 1.95 m.

The burial contained skeletons of four individuals, an adult and three children, all in excellent state of preservation.

The skeleton of an adult rested at the western wall of the burial chamber, contracted on the right side, head to the south, face to the east. The arms were sharply bent, with the hands placed at the front of the skull. The legs were sharply bent.

The skeleton of a child (I17974, Individual #6), 2–3 years old, rested in front of the skeleton of the adult, between the adult and another child, contracted on the right side, head to the south-southwest, facing east. The arms were sharply bent, with the hands placed at the front of the skull. The legs were sharply bent.

The skeleton of another child (I17975, Individual #8), 3–4 years old, was laid near the eastern wall of the pit, with the back close to the first child (Individual #6), contracted on the right side with a slight inclination to the chest, head to the south-southwest, facing southeast. The arms were sharply bent, with the hands placed at the front of the skull. The legs were moderately bent.

The skeleton of the third child, 7–8 years old (I17973, Individual #5), was located at the northern wall of the pit, at the feet of the adult and the first two children, contracted on the left side, head to the southeast, facing southwest. The arms were sharply bent, pressed to the chest, with the hands placed at the mandible. The legs were sharply bent, the knees were pressed to the elbows, heels to the pelvis.

All skeletons, especially the frontal parts of the skulls, were intensely colored with crimson ochre. In the southwestern corner of the pit, 0.25 m from the back of the head of an adult, there was a piece of crimson ochre 0.05 m in diameter.

Burial inventory included three clay vessels, a bone pin with a curved end and a pointed opposite end, and a 0.2 cm round hole in the central part (found under the mandible on the chest of the third child (individual #5), a flint insert of a sickle (at the parietal part of the skull of the third child), beads made from shell valves (found on the remnants of the plant mat under the skeleton of the third child), gold temporal ring (under the right temple of the first child (individual #6)), and a flint flake without retouching (laid 0.05 m from the radius bones of the second child (individual #8)).

S17974 and S20195 were considered duplicates during DNA amplification and appeared genetically identical. Thus, individuals 6 and 8 (first and second child) are identical twins.

Cioburciu Kurgan 4

Cioburciu, Ștefan Vodă District (46.599, 29.722)

The Cioburciu kurgan group was located 7 km south of the Cioburciu village, 200 km west of the Cioburciu – Ștefan Vodă highway, on the edge of the plateau of the west bank of the Dniester, limited by a gully-beam system and stream tributaries of the Dniester. The group consisted of five kurgans and was stretched along a north-south line.

Kurgan 4, the largest in the group, 2.7 m high and 60 m in diameter, was located 50 m northwest of Kurgan 3 and 80 m north of Kurgan 2. Kurgan's embankment was subjected to intensive plowing. Thirty-eight burials, eight of them belonging to the Yamna and four belonging to the Catacomb archaeological complexes, were discovered in the kurgan[137].

I20067

CiK4.3, Cioburciu, Kurgan 4, Burial 3, female, 3300-2500 BCE

Burial 3 (Yamna archaeological complex) was discovered in the southeastern sector of the mound, 2.1 m from the central benchmark at a depth of 1.7 m. The contours of the burial chamber were not traced. The skeleton of an adult lay contracted on the back, tilted to the left, with the head to the south-southwest. The skull was facing left. The left arm was extended forward along the body, the humerus and radius bones were partially preserved, the right arm was slightly bent, and the hand rested on the pelvis. The legs were bent to the left, the degree of skeletal contraction was average. The skeleton was painted with bright red ochre, most intensely the skull. Dark brown decay was traced under the interred.

Cotiujeni Kurgan 1

Cotiujeni, Briceni District (48.346, 26.938)

The kurgan chain of five kurgans near the village of Cotiujeni was studied in 1986 [138]. Kurgan 1 was the largest in the group. It was 44 m in diameter and 1.5 m in height. The kurgan contained eight burials.

I20068
CoK1.6, Cotiujeni Kurgan 1 Burial 6, male, 2881-2671 calBCE (4175±20 BP, PSUAMS-10740, this report)

Burial 6 (double burial, Yamna archaeological complex) was discovered 6.3 m west of the central benchmark at the depth of 1.67 m. The rectangular, 1.7 × 1 × 0.6 m burial chamber with rounded corners was oriented along the WNW-ESE line. Both skeletons were laid contracted on their backs, legs to the right, head to WNW. The skeletons were covered with red ochre, more intensely in the head and pelvic area.

Crasnoe Kurgan 9
Crasnoe, Grigoriopol District (47.131, 29.320)

Kurgan 9 was located on the west bank of the Lower Dniester near the village of Crasnoe, northeast of Grigoriopol, 2.5 km northeast of the Grigoriopol-Crasnaia Gorca highway. The kurgan was located on a plateau, on an arable field. It was the largest in the kurgan group. The northern foundation of the kurgan was steeper and 10 m shorter than the southern one. The preserved height of the mound from the buried soil was 4.8 m, from the mainland - 5.4 m, diameter - 60 m. The length of the northern part of the mound was 20 m, the southern part was 30 m in length. The kurgan contained 23 burials. The kurgan was investigated by E. V. Yarovoy in 1979 [23].

I20196
Kr/K9/B9/S2, Crasnoe Kurgan 9, Burial 9, Skeleton 2, female, adult 3352-3101 calBCE (4510±25 BP, PSUAMS-8835, this report)

I17977
Kr/K9/B9/S2(d), Crasnoe Kurgan 9, Burial 9, Skeleton 3, female, subadult, 3352-3101 BCE (based on the date from a co-buried individual I20196)

A triple Burial 9 (female, male and subadult) was found in a pit covered with three massive stone slabs in the southwestern sector of the mound at a depth of 3.4 m from the ground to the stone slab cover. On the western side of the grave cover there was a massive slab of irregular rectangular shape with an oval protrusion on one side, with clearly visible traces of processing. Placed on the side, the slab resembled a generalized image of a bull in profile.

The individuals were buried in extended supine position. All three skeletons were intensively painted with crimson ochre. The male skull was ornamented with bands of ochre. An animal shoulder blade was placed under the right foot of the male skeleton.

The skeleton of an adult female (Individual 2, I20196) lay in an extended position on the back with slight tilt to the left, head to the east-southeast. The skull was inclined towards the left shoulder, facing southwest. The left arm was straight, pressed against the body. The right arm was bent at a right angle, the hand was placed at the pelvis. The spine was bent to the left side - towards the subadult (Individual 3).

Burial Inventory included a 7 × 2.4 × 0.6 cm adze made of local Dniester flint, which was found at the western wall of the pit in the filling 0.1 m west of the feet of the male skeleton.

Two specimens, presumably from Individual 2, were sampled for aDNA analysis. The two samples appeared to belong to two first degree female relatives, thus indicating that two

different individuals from the same burial were sampled and that Individual 3 (I17977) was likely a daughter of Individual 2 (I20196).

Cunicea

Prişanscaia Gora, Soldaneşti District (47.904, 28.674)

The cemetery and settlement at Cunicea-Prişanscaia Gora is a Cucuteni-Trypillia Vykhvatyntsi-type site (Trypillia CII chronological period). The site was excavated in 2012[139]. Samples for molecular archaeology analysis were obtained from S. M. Agulnikov in 2015.

I7920

12M1, Cunicea-Prişanscaia Gora, Section 4, Layer 3, Burial 4, male (adult) 3350-3099 calBCE (4505±25 BP, PSUAMS-4872, this report)

Human remains included maxilla with teeth and long bone fragments.

Dănceni II

Ialoveni District (46.968, 28.717)

The late Trypillian ground cemetery at Dănceni II was located on the edge of a low (5-6 m) terrace above the floodplain on the east bank of the Ishnovets River, about 1.5 km northwest from the village. The cemetery was investigated in 1976 by I.A. Rafalovich and V.A. Dergachev. The burial ground consisted of four or five burials, which were organized in a semicircle with a radius of 6-7 m. To the east of the conditional center of the circle were two adult burials, to the south there was one child's burial, and to the north, destroyed burials of one or two more children were located. The distance between the burials was 1.5-4 m.

The Dănceni site is considered poly-cultural by archaeologists. It reflects interpenetrating traditions of the late Trypillian Gordinești and Usatove cultural groups[140,141]. The site is located approximately halfway between the major Usatove culture sites in the northwest Pontic such as Mayaky and Usatove-Velykyj Kuyalnik, and the Gordinești sites of Cucuteni-Trypillia Archaeological Complex in the middle Dniester area such as Pocrovca and Prydnystryasnske.

I20069

DIIGB301, Dănceni II Ground Burial 2 (301), male, 3323-2935 calBCE (4435±20 BP, PSUAMS-8832, this report)

The rectangular burial chamber of Burial 2 (301) was located at a depth of 0.47 m. It was oriented south-southeast-north-northwest. The interred was laid in a contracted position on the back, knees turned to the left, head to the north, arms extended along the body.

To the left of the interred, at the level of the elbow, there were two amphorae with handles-ears. One of the amphorae (10 cm in height) was equipped with four legs (broken off in ancient times) and covered with painted patterns. The second amphora (12.7 cm in height) was equipped with a lid with a conical ledge with a horizontal hole and two stop handles with vertical holes (6.6 cm in height). The amphora was apparently painted, but the painting has not been preserved. At the feet of the interred, closer to the southwestern corner of the burial pit, there were two bowls made of clay containing shattered burnt shards (chamotte). One of the bowls was ornamented with sickles, cord imprints, and paired knobs. The second bowl (10 cm in height) was decorated with double pinpricks and cord impressions filled with white paste. There was a jar (18.5 cm in height) near the knees of the interred. The upper part of the second similar vessel was found at the feet (14.4 cm in height). Another large vessel, decorated with "sickles" and cord

impressions (31.4 cm in height), was located under the eastern wall of the burial chamber. Dispersed flint flakes and coals were found at the bottom of the chamber.

Giurgiuleşti
Cahul District (45.483, 28.2)

The Giurgiuleşti sanctuary and necropolis was located on the east bank of the Danube. Detailed descriptions of the Giurgiuleşti archaeological complex and associated finds, as well as burial descriptions, can be found in [25,142].

I20072
GK2.6, Giurgiuleşti Kurgan 2 Burial 6, male, 4430-4058 calBCE (5370±26 BP, MAMS-23175[25])

Burial 6 (Gave 3 according to the burial sequence in[25]): a 2–3-year-old child buried in a catacomb with a round vertical shaft, narrow entrance into a chamber, and a domed chamber.

I20073
GK2.11, Giurgiuleşti Kurgan 2 Burial 11, male, 20-25, 4484-4346 calBCE (5571±32 BP, MAMS-28088[25])

Burial 11 (Grave 4 according to the burial sequence in[25]): a 20–25-year-old male buried in a 5 m-deep pit, accompanied by weapons, including a composite sword and spear, jewelry, and sacrificial offerings.

Glinoe (Hlinaia)
Slobodzeya District (46.6797, 29.8099)

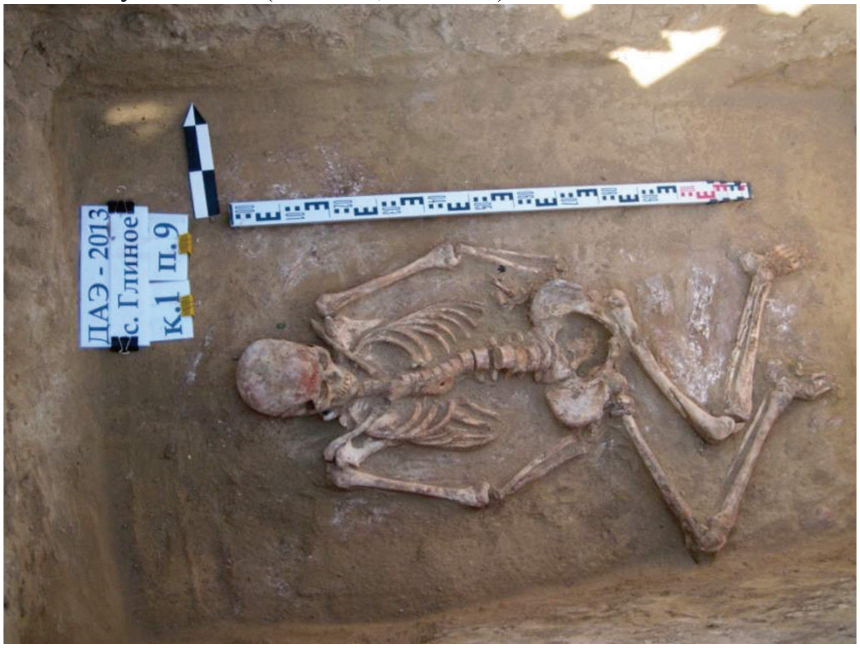

Figure SI1. 5. DOT Kurgan 1, Burial 9. Photo by V. Sinica, 2013.

Two groups of kurgans near the village of Glinoe (Hlinaia) on the east bank of the Lower Dniester were investigated in 2013 by V.S. Sinica. The kurgan group "DOT" is located 1.82 km north-northeast of the northern outskirts of the village, adjacent to the Tiraspol-Dnestrovsk highway. The “SAD” kurgan group is located 2.6 km northeast of the northern outskirts of the village[143].

I12507
DOT kurgan 1, burial 9, DOT K1B9, female (35-50), 2914-2882 calBCE (4275±20 BP, PSUAMS-10777, this report)

Kurgan 1 was located on the slope of a floodplain terrace. On the southern side the height difference from the center to the bottom was about 1.3 m, and on the northern side - about 0.2-0.3 m. The kurgan was erected over Burial 18 of the Yamna archaeological complex. In total, 19 burials of different periods were recorded in the kurgan.

Burial 9 (Yamna archaeological complex) was made in a pit with a ledge. The buried was laid on the back, with the upper extremities along the body, the lower extremities flexed, and the head to the west. The skeletal remains were well-preserved and rather complete. Bones from all parts of the skeleton were present. On the frontal bone there was a strip of red ochre. The individual was estimated to be an adult, 35-50 years (age-at-death was estimated based on cranial suture closure and tooth wear). Morphological traits of the skull (frontal bone, mastoid process, supraorbital ridge, zygomatic bone, and gonial angle) indicated female sex, as confirmed by the aDNA results. Based on long bone measurements, the body height of the individual was 166 cm. The burial inventory consisted of five copper tubular piercings and a mollusk shell piercing as part of a necklace around the neck.

I12512

SAD Kurgan 1, Burial 25, SAD K1B25, male (45-50), 2881-2633 calBCE (4165±25 BP, PSUAMS-10778)

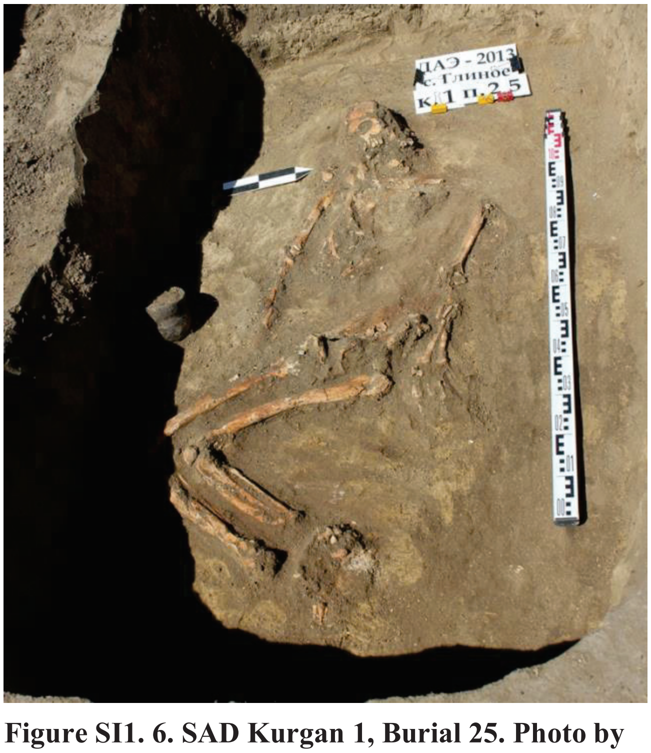

Figure SI1. 6. SAD Kurgan 1, Burial 25. Photo by V. Sinica, 2013.

Kurgan 1 of the “SAD” group was actually two kurgans with fused foundations. The northwestern kurgan (Kurgan 1A) was built in two stages. The first mound with a diameter of up to 19 m, consisting of dark gray chernozem, was built over the main burial 3 of the Yamna archaeological complex. At the time of excavations, the mound was heavily plowed, its maximum height above the ancient horizon was 0.8 m. Later, southeast of the first mound of Kurgan 1A, at a distance of about 20 m from its center, Kurgan 1B, with a diameter of up to 25 m, was built over the main Yamna burial 15, and the center of the mound was shifted to the northwest. In total, 27 burials from different periods were recorded in the mound.

Burial 25 was made in a pit. The interred was laid on the back, with the upper limbs along the body, the lower limbs flexed, and the head to the west. The skeletal remains were poorly preserved and incomplete. Fragments from all parts of the skeleton were present. The individual was estimated to be an adult, 40-50 years (age-at-death was estimated based on tooth wear and pubic symphysis morphology). Morphological traits of the skull and pelvis (mastoid process, supraorbital ridge, orbits, and subpubic angle) indicated male sex, as confirmed by the aDNA results. A reconstruction of the body height of the individual was not

possible owing to the insufficient degree of bone preservation. The burial inventory consisted of a molded goblet to the right side of the skeleton.

I12637

SAD Kurgan 1, Burial 26, K1B26, male, 3300-2500 BCE

Burial 26 was made in a pit. The interred (non-adult) was laid on the back, with the upper extremities along the body, the lower extremities flexed, and the head to the west. The skeletal remains were moderately preserved and partially incomplete. Fragments from all parts of the skeleton were present.

The individual was determined to be an infant, 0.5-1 years (age-at-death was estimated based on the dental development and eruption and long bone measurements). The male sex of the individual was genetically determined. The burial was without an inventory.

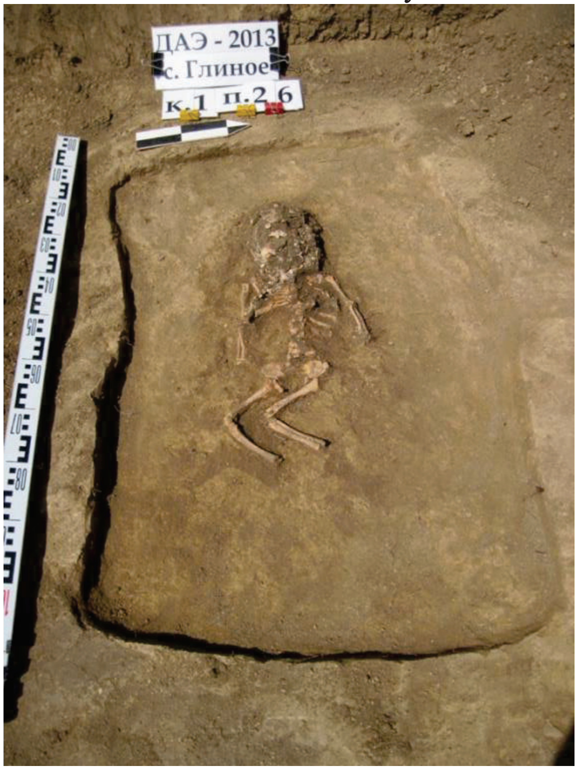

Figure SI1. 7. SAD Kurgan 1, 1Burial 26. Photo by V. Sinica, 2013.

Mărculeşti Kurgan 3

Mărculeşti, Florești District (47.869, 28.248)

Kurgan 3 was excavated in 1986 [144]. It was the largest of the kurgan group, with the diameter of 62 m and 3 m in height.

I17742

MK3.15, Mărculeşti Kurgan 3 burial 15, female, 3300-2500 BCE

Burial 15 (Yamna archaeological complex) was discovered 13.5 m south of the central benchmark, at a depth of 4 m. The rectangular burial chamber 1-1.1 x 1.67 m was oriented along the east-northeast-west-southwest line. The chamber floor was covered with a plant mat. Brown-yellow ochre was observed under the skeleton. The interred was laid contracted on the back, legs to the left head to the east-northeast. The entire skeleton was covered in ochre.

Mereni II Kurgan 1

Mereni, Anenii Noi district (46.95, 29.05)

Kurgan 1 was excavated in 1986-87 by V. A. Dergachev and E. N. Sava. It was located on a high promontory approximately 3.2 km to the east of the Mereni village. The kurgan stood 1.2 m above the ancient surface and was about 35 m in diameter. A total of seven fill layers have been identified in the kurgan. The kurgan contained 16 burials[145].

I17743

MeII1.15, Mereni II Kurgan 1, Burial 15, female (30-35), 3358-3100 calBCE (4515±30 BP, PSUAMS-7822)

Burial 15, main burial in the kurgan, was found in the center of the kurgan at a depth of 2.2 m, beneath a wooden covering of the pit. The burial chamber was dug from the day surface

level in a rectangular pit (1.8 × 1.35 m, depth 0.55 m), oriented from southeast to northwest. Due to the location of the kurgan on the edge of a high promontory with dry sandy soil, the remains of the wooden burial structure were unusually well preserved. The longitudinal walls of the chamber were lined with two thick hewn oak logs. The 1.55 × 0.5 × 0.22 m log under the northeast wall was set on edge. All its surfaces bore traces of adze-like tools with a working blade width of 3.5-4 cm. The surface of the log facing the chamber wall showed signs of burning. Another log, 1.4 × 0.23-0.25 × 0.23-0.24 m, also set on edge, was located under the southwest wall. It was also worked with an adze-like tool, and the surface facing the chamber wall had burn marks. Two short oak logs set on edge were found under the short end sides of the chamber. The log under the southeast wall (0.8 × 0.5 × 0.14-0.16 m), like the block under the northwest wall (1.0 × 0.48 × 0.17-0.2 m), also bore traces of adze-like tools. However, in the first case, the surface of the log facing the chamber wall was heavily burned, while in the second case it was not.

The chamber's covering consisted of five 1.3 m long, 0.14-0.2 m wide, and 0.7-0.9 cm thick hewn oak planks set across the chamber. Their ends rested on the lower logs laid along the long walls of the chamber. The chamber walls were vertical, and the bottom was flat and horizontal.

The buried individual lay in a flexed position on the right side, with the head to the southeast. The arms were bent at the elbows, with the hands at the chin. The legs, with the knees raised to the right, almost touched the pelvic bones with the feet. No traces of ochre were observed. On the bottom of the chamber, brown and whitish decay from the plant bedding was noted.

In front of the face of the deceased, in the eastern corner of the chamber, stood a late Trypillia-style globular amphora (12.5 cm high) with two loop handles with vertical holes. It was made of typical Trypillia clay, without visible inclusions, of high-quality firing, and a pinkish color. It was decorated with metope compositions filled with a brown net pattern.

Ocniţa Kurgan 1

Ocniţa District, west bank of the Lower Dniester (48.385, 27.439)

I20076

OcK1.3, Ocniţa Kurgan 3, Burial 14, female, adult, 2906-2702 calBCE (4230±25 BP, PSUAMS-11947)

A kurgan group located 1.5 km southeast from the Ocniţa village was investigated in 1988[146]. Kurgan 3, based on stratigraphic profiles, consisted of three overlapping mounds. The original mound was constructed over Burial 17 of the Yamna archaeological complex. Subsequently, Burial 14 of the Yamna archaeological complex was incorporated into the northern floor of the second mound. The remaining Yamna burials in the kurgan were situated within the third mound, forming a circle relative to the mound's center, where one group of five burials formed the inner circle, and a group of four burials formed the outer circle.

The burial structure of Burial 14 comprised a 0.9 m deep inlet pit and a rectangular burial chamber measuring 2.1 × 1.32 × 1.06 m, with a depth of 0.95 m. The chamber's northeastern part was oriented along the northeast-southeast direction. The fill consisted of mixed loose soil with remnants of a transverse wooden ceiling, part of which was preserved at the edge of the northwestern wall of the chamber. An adult individual was interred in a flexed position on the back, head to the southwest at 240 degrees, face up, with arms slightly moved to the sides and

left hand resting on the pelvis. Initially raised with knees up, the legs had fallen to the left. The bones were covered in red ochre, most intensely on the right calcaneus and humerus of the right hand. Dark brown decay from a mat interspersed with ochre was traced beneath the interred.

In the western corner of the chamber, a molded amphora of the Globular Amphora style measuring 24 cm in height, with a rim diameter of 12.5 cm, body diameter of 23.4 cm, and bottom diameter of 8.8 cm, was discovered. The vessel, made of a clay mix with ample sand and occasional chamotte grain inclusions, exhibited high-quality firing, with a dense, reddish shard. The outer and inner surfaces were carefully polished, with a gray-black color on the outer surface and a grey color on the inner surface. The vessel had a round-conical body with convex sides and a flat, well-defined bottom. The high and cylindrical neck featured a straight and rounded corolla, with a noticeable low ridge at the neckline and shoulders. Four small vertical handles with round horizontal holes adorned the shoulders, and the edges of these handles resembled low sides. The amphora displayed rich ornamentation, including four parallel horizontal lines of imprints resembling a twisted cord under the rim. Below, short horizontal sections of the same prints formed vertical fields, followed by rows of sickle-shaped depressions, mirroring a similar ornamental system located below the sides at the shoulders.

Sărăteni Kurgan 1

Sărăteni, Leova District (46.613, 28.465)

The Sărăteni kurgan was located approximately 0.9 km east of the southern periphery of the village of Sărăteni. The kurgan occupied the northernmost position within a group made of four kurgans arranged in a row on the edge of a plateau rising above the Prut valley. The mound of the kurgan was plowed annually, and, at the time of the research, it had an irregular circular configuration in plan. The kurgan was about 2 m high and 25 m in diameter. The kurgan contained 14 asynchronous burials.

I17744

SK1.1, Sărăteni Kurgan 1 Burial 1, male, 3300-2500 BCE

Burial 1 (collective, Yamna archaeological complex) was detected in the SW sector of the kurgan, 6.3 m from the center and at a depth of 2.42 m. The grave contained the remains of four individuals, two children and two mature adults. Burial descriptions are presented in[147].

I17745

SK1.9, Sărăteni Kurgan 1 Burial 9, male, 3300-2500 BCE

Burial 9 (Yamna archaeological complex) was discovered in the SE sector of the kurgan, at a distance of 7.6 m from the center and a depth of 3.4 m. The skeleton of a child, moderately twisted on the left side, oriented with the skull to the NE at 70°, facing south. The arms, bent at the elbows at an angle of almost 90°, were placed with forearms forward. The preserved leg was bent at a right angle to the body, and the knee at an angle of 180°, so the tibia was parallel to the femur. The bones of the skeleton were moderately covered with ochre. At the bottom of the pit, in some places, remnants of a plant mat were discovered.

Taraclia II Kurgans 2 and 10

Taraclia, Căușeni District (46.569, 29.116)

A group of 20 kurgans was located on the watershed between the Yalpug and Lungutsa rivers and was a continuation of the kurgan group near the Kazaklia village. The kurgans within

the group were located quite compactly in the form of a strip elongated in the meridional direction. In 1980 and 1982-1984, 19 kurgans were investigated by S. M. Agulnikov, I.V. Manzura and E.N. Sava. Burials of the Late Eneolithic and Late Trypillian chronological period were found in kurgans 2, 10 and 18[148].

Kurgan 2 (H. 1.2 m, D. 36 m) was located at the southern tip of the kurgan group, 0.15 km south from the Taraclia-Novoselovka road. The kurgan contained 15 burials. The kurgan was built over burial 14 (Zhyvotylivka type). Subsequently, burials of the Yamna complex were deposited into it, in the result of which the original center of the mound shifted to the northwest.

Kurgan 10 (H. 9.9 m, D. 80 m) was the largest of the kurgan group. It was located in the southern part of the group. The kurgan contained 21 burials. The first mound (H. 2.7 m, D. 46 m) was built over burials 16 and 17 of the late Trypillian period. The original kurgan was surrounded by a ring ditch (47 m × 1 m × 0.6 m). During the EBA, several burials of the Yamna archaeological complex were deposited into the original mound. Additional mounds built over these burials increased the size of the kurgan.

I20078

TIIK2.14, Taraclia II Kurgan 2 Burial 14, male, 3340-3034 calBCE (4480±25 BP, PSUAMS-8833)

The double burial 14 in a square pit (1.7 m × 1.7 m × 1 m) was located 7.5 m to the southeast from the presumed center of the kurgan. Two skeletons of adult individuals rested in a contracted position on the left side, head to the northeast (80°). Arms were bent, hands in front of the face. The bones of both skeletons were painted with crimson ochre, especially the skulls. At the bottom of the pit, dark brown decay from a plant mat was traced.

Inventory consisted of a Trypillian vessel (H. 9.5 cm), decorated with a painted net ornament, which stood in front of the face of the right (southern) skeleton (presumably the one sampled for this report).

I20079

TIIK10.2, Taraclia II Kurgan 10 Burial 2, male, 2571-2355 calBCE (3965±20 BP, PSUAMS-8834)

Burial 2 was located outside of the kurgan ditch, 31 m to the south from the presumed center of the kurgan, at a depth of 9.9 m. The pit was rectangular in shape, oriented NNW-SSE (1.5 m × 0.65 m × 0.35 m). The poorly preserved skeleton of a child rested in a likely contracted position on the left side, with its head to the north.

Inventory: 1) A Trypillian amphora made of well-elutriated clay (H. 22 cm), painted with brown paint, stood at the feet. 2) A dark-gray goblet made of well-elutriated clay (H. 5.6 cm) laid on its side near the eastern wall, opposite the skeleton. 3) A bone hook-shaped pin with a hole (L. 3.5 cm) laid near the skull. 4) Cylindrical beads made of bone, a brownish mineral, and a shell (D. 0.5 cm), as well as a tubular copper thread, laid near the skull next to the pin. 5) Three beads made of a reddish and white mineral (D. 0.7-0.5 cm), as well as a copper bead, laid at the feet.

Tiraspol Kurgan 3

Tiraspol (46.848, 29.597)

Kurgan 3 near the city of Tiraspol was investigated in 1983[149]. The kurgan was located 500 m to the south of the Tiraspol-Odesa highway. The kurgan was 3.5 m in height from the buried soil and 60 by 60 m in diameter at the base. The kurgan contained 31 burials.

I17747

TiK3.15, Kurgan 3 Burial 15, male, 2865-2576 calBCE (4115±25 BP, PSUAMS-7864)

Burial 15, containing Usatove features, was discovered by a round spot of red-colored fired clay in the western sector of the kurgan, 7.7 from the central benchmark, at a depth of 3.55-3.6 m. The 1 × 06 × 4.03 m rectangular burial chamber had rounded corners and was oriented west-southwest-east-northeast. A child skeleton was laid on the back, slightly tilted to the left, skull to NEE at 75°. Legs were bent, likely up to the chest, with knees to the left. Fragments of a molded jar-shaped vessel were found next to the skull. The vessel was made from clay mixed with sand.

Ukraine

(Contact: A. G. Nikitin, S. Ivanova, I. Potekhina, M. Lillie, N. Kotova, T. Tkachuk)
Samples from Ukraine were obtained in 2005-2014 either directly from the authors of excavations or from the Bioarchaeological Stores of the Institute of Archaeology, National Academy of Sciences of Ukraine, Kyiv, following applicable laws and regulations.

Bil'shivtsi

Ivano-Frankivsk Region, Ivano-Frankivsk District (49.181, 24.75)

The multi-layer archaeological site at Bil'shivtsi (Kuty) is located on a loess hill on the west bank of the Hnyla Lypa River, the east tributary of the Dniester River.

I13071

BLV1, Bil'shivtsi-Kuty Burial 1, female (55-60), 2201-2032 calBCE (3720±25 BP, PSUAMS-7848)

An oval pit, 1.28 × 1.52 m wide and 2.57 m deep, was uncovered at Bil'shivtsi at level V stratigraphic sequence associated with the local Koshylyvtsi group of Trypillia[33]. A dog's skeleton in an extended supine position on the back with hind legs in the shape of a rhomb, skull to the east, facing north, was found at the bottom of the pit, 2.54 m deep. At the depth of 1.5 m three human crania without mandibles were excavated in the south-east part of the pit. Sherds of LBK and Trypillian pottery and a fragment of a clay female figurine were uncovered in the fill of the pit. The dog's skeleton was dated to 3793-3377 calBCE (4840±80 BP, Ki 8273).

At around 4 m to the southeast from the eastern edge of the burial of the dog and the three crania, a two-part burial chamber was excavated. The chamber consisted of a western rectangular 1.65 × 0.8 m wide and 1.9 m deep pit and an oval eastern pit in the shape of a catacomb, 2 × 1.65 m wide, 2 m deep, and 0.7 m in height. A sherd of a Funnel Beaker pot with cord print ornamentation was found in the fill of the catacomb.

The skeleton of a mature woman was found at a depth of 1.3 m in contracted position faced down, legs to the right, arms stretched along the body, left wrist missing, oriented south-east. The bones of a juvenile sheep were found under the torso. A bovine mandible, laid out on ashes, was found in the east part of the pit, at the entrance to the burial chamber near the right foot of the interred. Grave goods included four polished boar tusks, a cylindrical 0.5 × 0.25 mm copper pin, and a triangular cooper knife 5.7 cm long and 3.2 cm wide.

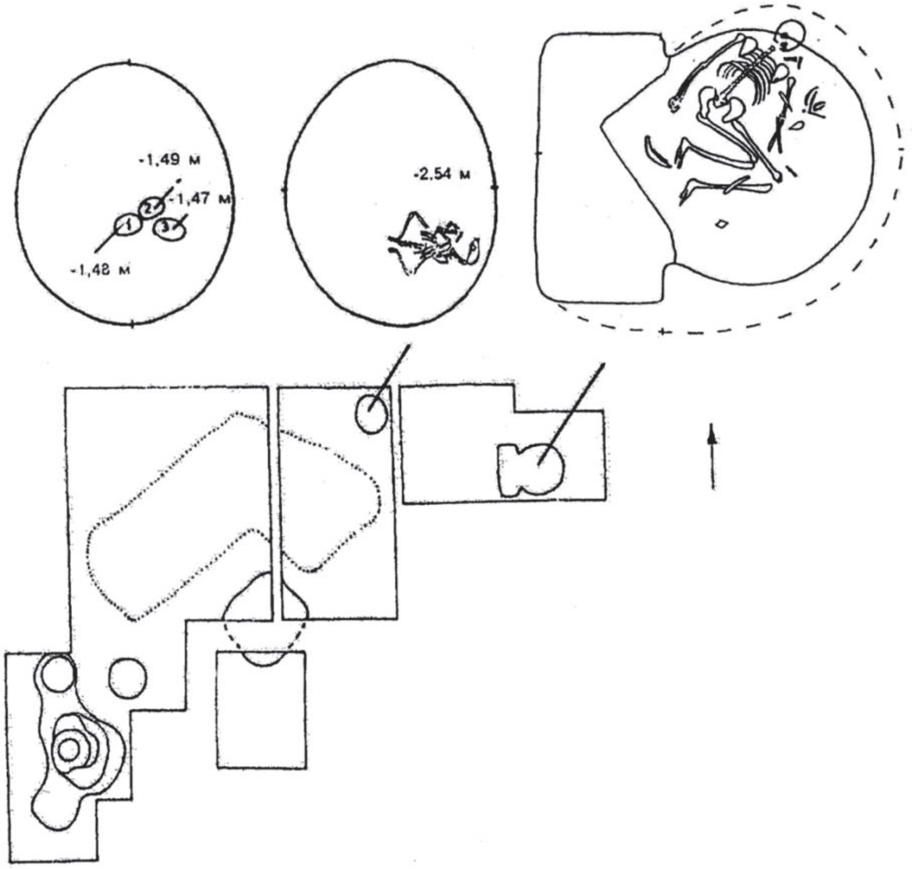

Figure SI1. 8. Bi'lshivtsi (Kuty) burial. Image by T. Tkachuk.

Deriivka II
Onufryiv District, Kirovohrad Region (48.933, 33.766)

The Eneolithic settlement of Deriivka II was discovered by an archaeological survey in 1959. The site was located on the west promontory of the Omelnyk River. The Eneolithic cemetery was located 400-500 m from the western outskirts of the settlement. The site was excavated in 1960-1967 and 1983 by D. Ya. Telegin. The Deriivka II anthropological collection is housed in the Bioarchaeological Stores of the Institute of Archaeology of the National Academy of Sciences of Ukraine in Kyiv. Burial descriptions are from[14].

I27282
Der15, Burial 8, male, 4322-4052 calBCE (5345±30 BP, PSUAMS-10370, this report)

A mature individual in a contracted on the back position, legs flexed upward, hands on the pelvis, eastern orientation.

I27283
Der41, Burial 11, Skeleton 1, male, 4444-4262 calBCE (5500±30 BP, PSUAMS-10371, this report)

Paired burial with a child. Half-sitting position, legs flexed, hands placed near the abdomen. A polished stone axe was placed near the left half of the pelvis.

I28319
Der16, Burial 5, female, 4321-4052 calBCE (5344±29 BP, weighted mean[10] of 5380±90 BP, OxA-5032[15]; 5340±30 BP, Beta-445808)

Burial of a mature female in a contracted on the back position, legs flexed and fallen to the right, oriented to the north. Arms bent at the elbows, hands on the pelvis.

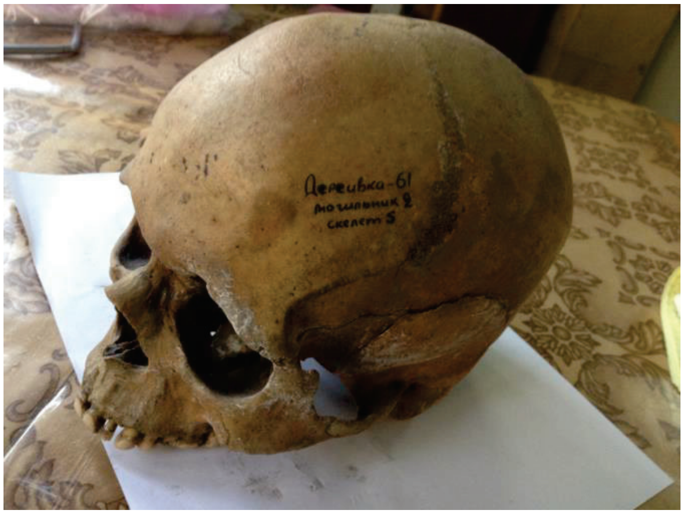

Figure SI1. 9. Deriivka 2 Burial 5. Photo by A. G. Nikitin, 2014.

Dubynove Kurgan 1
Dubynove, Odesa Region, Podilsk District, (48.133, 30.282)

The kurgan was located on a plateau on the west bank of the Southern Buh River, 200 m east of the village Dubynove, at the eastern side of the Kyiv-Odesa highway. An extensive hollow was observed around the kurgan, on the site of an ancient excavation of bulk soil. From the level of this depression, the height of the kurgan was 2.0–2.2 m with a diameter of the embankment of 36 m. A total of 15 burials were identified in the kurgan, including one Eneolithic (Burial 7), two Yamna (Burials 5, 13), five Catacomb (Burials 8–12), one Babyne (Burial 14) and one Late Bronze Age burial (Burial 3).

I12621
DBN1.9, Dubynove Kurgan 1, Burial 9, male (adult), 2575-2348 calBCE (3970±35 BP, PSUAMS-7791, this report)

The oval 2.1 × 1.4 m burial chamber of Burial 9 (Catacomb archaeological complex) was discovered 8 m to the southeast of the central benchmark, at a depth of 2.55 m. It was oriented with its long axis in the south-southwest - north-northeast direction. The entrance well was located at the eastern edge of the chamber. The interred was lying in extended supine position on the back, with his limbs straight, head to the south-southwest. The left hand was placed on the pelvis. The face was turned to the east. There was no inventory.

I12840
DBN1.10, Dubynove Kurgan 1, Burial 10, female (juvenile), 2453-2148 calBCE (3830±30 BP, PSUAMS-7805)

Burial 10 (Catacomb archaeological complex) was discovered 8.3 m southeast of the central benchmark, at a depth of 2.85 m. The burial structure was a 1.4 × 0.7 m elongated pit,

with a lining in the western wall. The interred lay on the back, head to the south-southeast. The skull was tilted towards the left shoulder, the arms extending along the body with the wrists resting on the pelvis. There was no inventory.

D1.11

Dubynove Kurgan 1, Burial 11, 2343-1905 calBCE (3720±70 BP, Ki-11202[13])

Burial 11 (Catacomb archaeological complex) was discovered 14 m southwest of the central benchmark, at a depth of 2.7 m. The burial chamber was either a poorly traced catacomb or a simple pit. It was trapezoidal in plan, measuring 1.5 × 0.75–1 m, with walls preserved to a height of 0.6 m (at a depth of -3.3 m). The pit was oriented lengthwise from east to west. The skeleton lay on the back, almost diagonally across the chamber, with the skull towards the northwest. The legs, initially raised with knees upward, had fallen to the right. A pink ochre deposit was found on the bones, and there was brown decay from bedding on the bottom of the grave. A hand-molded vessel stood in the southeast corner.

Grave Inventory consisted of an "amphora" with a straight neck, spherical body, and flat base distinguished by a rim. Two pairs of arched handles with vertical holes were symmetrically attached to the shoulders. The rim of the neck was marked with notches. Below the rim, it was segmented by seven horizontal grooves, giving it a ribbed appearance. The base of the neck was emphasized by incised lines filled with oblique notches forming a horizontal "herringbone" pattern. Between the handles, a double zigzag line filled with notches was incised across the shoulders. The vessel's body was divided by vertical lines with "herringbone" shading into four panels, which were marked with medians and connected to them by double angles with notches. The bottom of the vessel was decorated with two concentric circles with a cross inside. The exterior of the vessel was coated with light orange slip, and the interior was black-gray. The shard's fracture was dark gray, with chamotte inclusions in the clay. The vessel's height was 17.3 cm, the height of the neck was about 3 cm, the diameter of the slightly asymmetrical rim was 12.3–12.8 cm, the body diameter was 20.4 cm, and the base diameter was 10.3–10.7 cm. The radiocarbon date of the complex was determined by a sample from the bones of the lower limbs.

Low resolution PCR-SNP analysis of the mtDNA coding region of the specimen typed it to haplogroup U[28]. Additional analyses of the mtDNA control region at GVSU produced transitions at nucleotide positions 16192, 16270, 16256, 16293, and 16399, consistent with haplogroup U5a1g1.

Kam’yana Mohyla Kurgan 2

Myrne, Zaporizhzhia Region (46.951, 35.470)

Kam’yana Mohyla is a Ukrainian National Historical and Archaeological Preserve in the Steppe of Azov, 16 km north of from Melitopol, on the west bank of the Molochna River. Kam’yana Mohyla is a natural formation, the height of which reaches 12 m. It occupies an area of 1.6 hectares, on the territory of which there are about 3000 sandstone slabs. Geologically, the monument is a remnant of Sarmatian Sea sandstone (the Tertiary period), with cracks and faults that led to the accumulation of slabs and the formation of a large number of grottoes.

Long-term research of Kam’yana Mohyla led to the discovery of 65 grottoes and caves, on the ceilings of which several thousand petroglyphs from the Late Paleolithic and Mesolithic to the Middle Ages were discovered. The grottoes are mainly small (up to 5-8 m), low (1.2-1.5 m)

rock cracks with flat (or close to them) ceiling slabs. Nowadays, a large part of the grottoes is filled with sand (conserved) to ensure the preservation of ancient petroglyphs.

I20975
KM17P2; KM-2017 Kurgan 2, Burial 2, male, 2872-2627 calBCE (4141±21 BP, BE-8040.1.1[27])

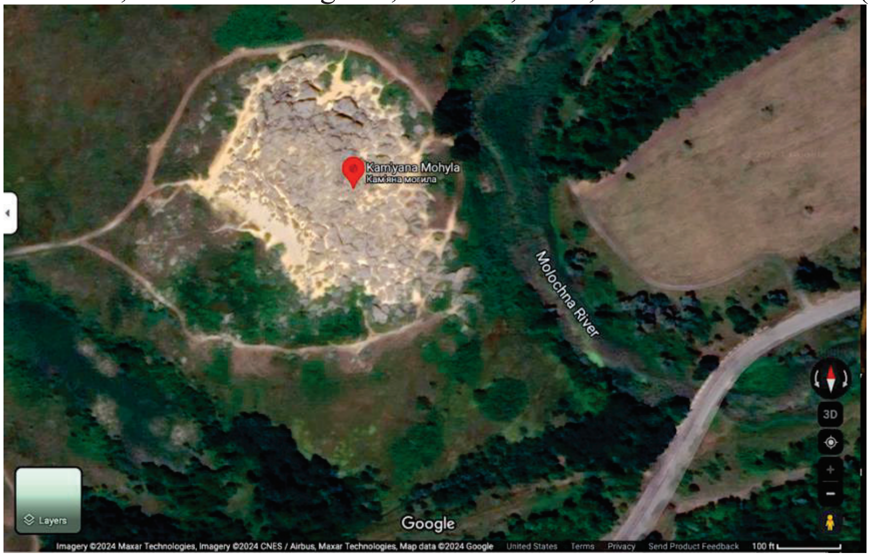

Figure SI1. 10. Kam’yana Mohyla. Map data via Google.

Kurgan 2 was excavated in September 2017 by the Azov Archaeological Expedition of the Institute of Archeology, National Academy of Sciences of Ukraine, led by S. V. Makhortykh[27,150]. The investigated monument was located 200 m north of Kam’yana Mohyla. No visible traces of the mound were recorded at the location of Kurgan 2 before the excavations began. The uneven, bumpy surface was planted with trees and covered with grass. During the excavations, the stratigraphy of the studied area became quite clear. The upper layer (from 0.19 to 0.25 m) consisted of chernozem. A layer of light gray loam, in many places disturbed by burrows, lay under it. The thickness of this layer varied from 0.35 to 0.50 m. Below the light gray loam was a thick layer of brown loam with numerous crushed shells. Its traced thickness was at least 0.4-0.6 m.

During the excavation of the kurgan, three burials and two presumed ritual complexes were discovered. They were located in a layer of light gray loam. Burials 1 and 3 were located in the kurgan’s middle and upper parts, burial 2 in a stone tomb, and two ritual complexes were in its lower part.

Burial 2 was made in a Globular Amphora-style stone sarcophagus, which was located on an ancient horizon consisting of brown loam with numerous admixtures of crushed shell. It had a rectangular shape and was built from rough-hewn sandstone slabs placed on an edge. The burial structure was oriented with its long sides along the northeast-southwest line. No stone covering over the tomb was found. Individual bones of animals, such as the rib of an adult bull, and the hoof of a horse, were associated with the burial. In the central part of the tomb, next to the southern wall, a Unio shell was found.

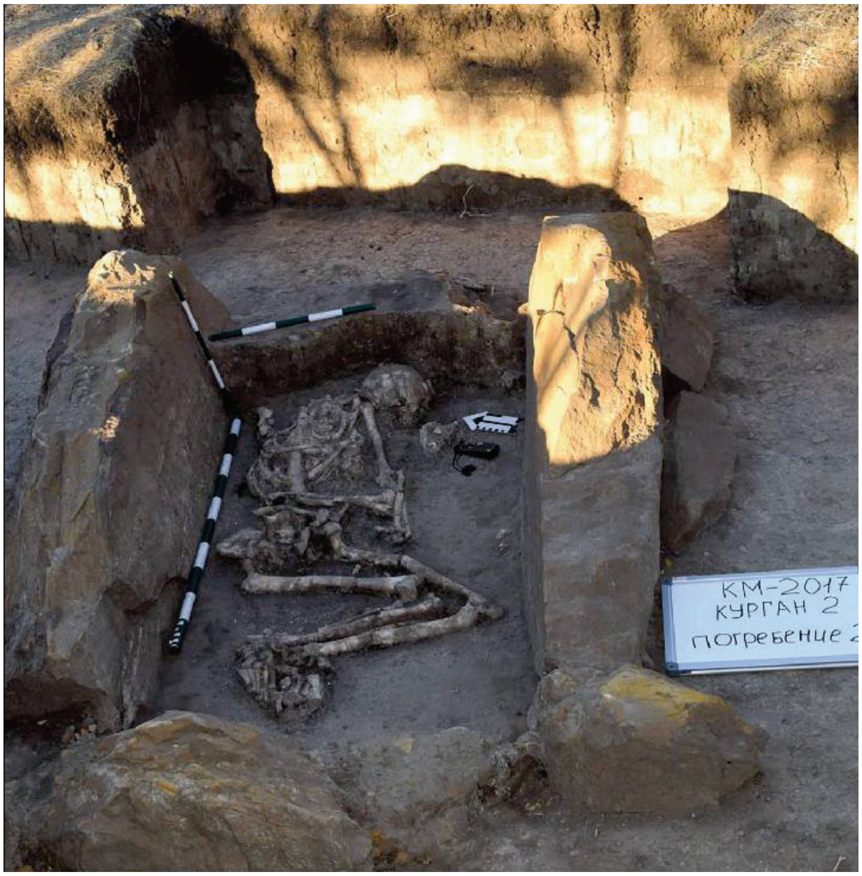

Figure SI1. 11. Kam'yana Mohyla Kurgan 2 Burial 2. Photo: S. Makhortykh, 2017.

The skeleton of a young male (< 25) discovered in the tomb was lying contracted on the left, with the head to the east, tilted to the north. The mandible was displaced and located in front of the face of the interred. The clavicle, revealed between the ribs, was also displaced. The hands of the deceased were also partially displaced. The left hand was placed extended towards the knees. The right arm lay perpendicular to the left and appeared to be separated from the body (the elbow and shoulder bones laid parallel to each other). The legs were bent at the knees and sunk to the left. The phalanx of the finger lying next to the right femur had a red articular surface.

Katarzhyno Kurgans 1 and 2
Znamianka, Ivanovsky district, Odesa Region (47.025, 30.288)

The Katarzhyno kurgan group was excavated in 1990-1991 by the expeditions of the Odesa Protective Archaeological Center under the Ukrainian Society for the Protection of Historical and Cultural Monuments. The kurgan group was located 4 km south of the village Znamianka (until 1932 - the village of Katarzhyno), on the plateau of the east bank of the Malyi Kuyalnyk river. The burial ground consisted of five kurgans. Four of them, including Kurgan 2 with a height of 2.4 m and kurgans 3–5, plowed up to the foundation, stood in a row from north to south. The largest kurgan 1, 6.45 m high, was located somewhat to the side. At the same time, the group closed a rarefied kurgan chain from the north, stretched along the meridional

watershed up to 120 m high above the thalweg of the river. Kurgan 1 of the Katarzhyno group is one of the five largest kurgans excavated in the northwestern Black Sea region.

Kurgan 1 was 80–85 m diameter. Its height at the beginning of the excavations was 6.45 m, while according to the topographic survey of 1950, the height was 7.3 m (the absolute mark is 134.7 m in the Baltic system of heights). The slopes of the kurgan were heavily raked and partially cut off. The summit had also been cut off and disturbed by modern digging. In total, 20 burials were found in the kurgan: one Eneolithic (burial 10), Yamna — 11 burials (1, 2, 6–8, 9 (?), 11–14, 16, 21), Catacomb — two burials (3, 5), Babyne – three burials (9, 17, 20), two burials of the Bronze Age (4, 18) and an unclassified burial 15.

Kurgan 2, 2.4 m high and about 50 m in diameter, was located 20 m northwest of Kurgan 1. The kurgan was heavily plowed, and its top was cut off. In total, six burials were found in the kurgan. Of these, two (Burial 1 and Burial 6) belonged to the Yamna archaeological complex. The other four burials belonged to the Babyne archaeological complex (Burial 5), late nomadic (Burial 2), and two of indeterminate cultural association (Burials 3, 4).

I11999

KTR1.1, Katarzhyno Kurgan 1 burial 1, male, adult, 2626-2475 calBCE (4045±20 BP, PSUAMS-4903, this report)

Burial 1 (Yamna archaeological complex) was found 25.5 m west-southwest of the central benchmark. At a depth of 6.25 m, in a section of 3.2 to 3 m, stones smeared with green clay and large unworked floor slabs of a stone tomb emerged. The southern slab of the stone cyst burst and sank. The tomb was a rectangular stone box of four slabs placed in a ground pit. After the burial, the pit with the tomb was filled in. The burial box, 1.9 × 1.2 × 0.9 m, was oriented in the north-south direction. On the northern, eastern, and southern slabs, traces of ornamentation with pale pink ochre have been preserved. The buried individual was laid with the head to the north, contracted on the back. The legs were bent and turned to the left but may have originally stood with their knees up. The arms extended along the body, the left arm was straightened, the right arm was slightly bent at the elbow, and its wrist almost touched the pelvis (the hand was not preserved). The skull was tilted towards the left shoulder. A limestone triangular slab, without traces of processing, was laid on the chest of the interred. Considering that the roof of the tomb over this part of the burial was not broken, it can be assumed that the slab was placed deliberately. Small sharp-angled stones were placed in the upper left part of the chest, between the femurs, and on the right tibia. At the head, at the left forearm, and the corners of the tomb, scatterings of pink ochre powder were traced. On the chest bones on the right were traces of fire in the form of charcoal and burnt pieces of bark. At the elbow of the right hand and the right wrist, two copper or bronze tubular piercings were found. Other than these, no other funeral offerings were found in the tomb.

I31733

KTR1.10, Katarzhyno Kurgan 1 burial 10, female, 3955-3783 calBCE (5039±27 BP (weighted mean[10] of 4970±110 BP, Ki-11376, 4950±70 BP, Ki-9523[13], and 5060±30 BP, PSUAMS-10375, this report)

The main burial 10 was found 6.5 m southeast of the central benchmark, under an embankment of several layers of earth and a layer of stone. At the level of the ancient day surface, the burial pit was covered with a reed mat containing a preserved direction of the fibers and a single layer of limestone. Among the stone slabs was a fragment of an anthropomorphic

stelae with a chiseled shape resembling a human head. The reed mat near the stellae was covered with a layer of 5–8 mm organic matter in the form of a dense yellowish-white streak (possibly remnants of honey), covered with a large fragment of a ceramic pot. Two other slabs without signs of processing were laid on the sides of the stone syst on the east side. The bottom of the tomb was covered with a dark brown decay of the bedding, on which the interred was laid contracted on the back. The legs were bent, and the knees turned to the right, the arms were straightened and laid along the body with the palms down, the skull was tilted to the left shoulder, and the orientation was southwestern. The reconstructed height of the individual was 178-193 cm[28].

A thin deposit of ochre was detected on the bones A 2.5 × 6 cm lump of ochre laid near the left hand at the level of the elbow. A 2.5 × 10 cm lump of ochre laid on a layer of coal placed on the chest near the right clavicle. At the left hand, closer to the shoulder, there was a vessel placed on a layer of coal. Other finds are represented by flint tools. One of them, with retouched ends, was found under the skull, the other came from the grave fill.

Burial inventory consisted of the following items: 1) A molded asymmetric *askos*-type vessel with an oval-shaped broken-off handle. 2) Half of a molded clay pot with a missing bottom of rough dressing with an ample admixture of crushed shells and a corded ornament in the form of a horizontal four-row belt and bows attached from below. 3) A chisel-type greyish-smoky flint tool with two working sides. 4) The same type of tool (gray flint), but more massive and with one working side.

Ancient DNA from the specimen yielded low coverage at Reich's Lab. On the whole genome PCA, the individual mapped at a distal end of the Eneolithic populations of the Caucasus.

I12168

KTR1.13, Katarzhyno Kurgan 1 Burial 13, male, adult, 3024-2908 calBCE (weighed mean of 4335±25 BP, PSUAMS-9815, and 4375±20 BP, PSUAMS-4762, this report)

Burial 13 (Yamna archaeological complex) was found at a depth of 2.35 m, 5.6 m south of the central benchmark. It was not possible to trace whether there was a ledge near the burial chamber. The 2.0 × 0.9 m chamber was covered with four large limestone slabs without traces of processing. The chamber was rectangular with rounded corners, oriented in length in the northeast-southwest direction. The skeleton was poorly preserved. The humerus of the right hand was missing, the skull was destroyed. The interred was laid on the back, with the head to the northeast. The arms were extended along the torso. Bent legs were placed with knees up. At the bottom of the grave was a bedding of bark sprinkled with chalk. A layer of ochre was deposited at the left shoulder.

I12170

KTR-2.1, Katarzhyno Kurgan 2 burial 1, female, adolescent, 3348-3038 calBCE (4490±30 BP, BETA-647662, this report); 3314-2537 calBCE (4270±90 BP, Ki-17331[13])

The main Burial 1 (Yamna archaeological complex) was found 1.4 m south of the central benchmark, at a depth of 2.3 m. At this level, a deposit of small stones (0.2–0.3 m in size) was recorded, which lay flat in one or two tiers within the grave. Under the stones, the dust of the wooden floor was traced. The burial chamber was rectangular in plan, 1.7 × 1.1 m, with rounded corners, 0.6 m deep from the level of the ancient day surface. The buried individual was laid on the back, with the head to the northeast. The arms were slightly spread apart, bent at the elbows,

and extended along the body. The legs were turned with the knees to the right. Between the right side of the chest and the pelvis was a stone tool. A small piece of limestone rested on the sacrum. Under the pelvic bones and above, at the level of the lumbar spine, a layer of 1 cm of green clay was traced. Burial inventory: 1) Gray sandstone slab, prismatic shape, 11.7 × 6.3 × 3.4 cm, used as a pestle and grater stone. As a result of use, the upper plane of the tool was polished, and the lower and all side faces were uniformly covered with characteristic potholes. 2) Sturdy limestone tile in half-segment shape, 6.6 X 3.6 × 2 cm, with sharp edges. One edge was blunted during the use of the stone, possibly as a scraper or adze.

Kolomiytsiv Yar Tract

Kopachiv, Kyiv Region**,** Obukhiv District (50.130, 30.480)

The Kolomiytsiv Yar Tract archaeological site and burial are detailed in[16].

I7585

KST07, female, 4049-3820 calBCE (5170±30 BP, BETA-523816[16])

Mariupol Necropolis

Mariupol, Mariupol District, Donetsk Region (47.0998, 37.6003)

The necropolis was located on the east bank of the Kalmius River, on a peninsula between the shores of the Sea of Azov and the bend of the Kalmius River, opposite the city of Mariupol. The blast furnace of the Azovstal steel plant was built on the site of the necropolis in the summer of 1931. The necropolis was excavated by Mykola Makarenko in 1930, August 10 through October 15[1].

The necropolis was distinguished in the ground by a strip of red clay 28 meters in length and 2 meters wide. On the peninsula where the necropolis was located and up to the gully that separates it from the general coastal plain, such clays were not present. Deposits of similar clays have been found in ravines and cliffs of the Kalmius River at a significant distance upstream from the burial site. The burials, all in extended supine burial position except for the triple burial 21, were located in this clay strip 0.40 to 0.75 meters from the surface, with a maximum depth of 1.5 meters. A total of 124 burials were excavated.

Extended burials were archaeologically assigned to the Neolithic period. Extended on the back with bent knees burials 21 (a double burial with a cremation) and 24 (covered in red ochre) were considered by Makarenko to be of a later period. The Neolithic double burial 50 and the double burial 21 were cut out of the matrix in the field by Makarenko and brought to the Mariupol Museum of Local History, where they were sampled in the fall of 2021 for the current analysis. The museum and its collections were destroyed by the Russian military during the siege of Mariupol in the spring of 2022[151].

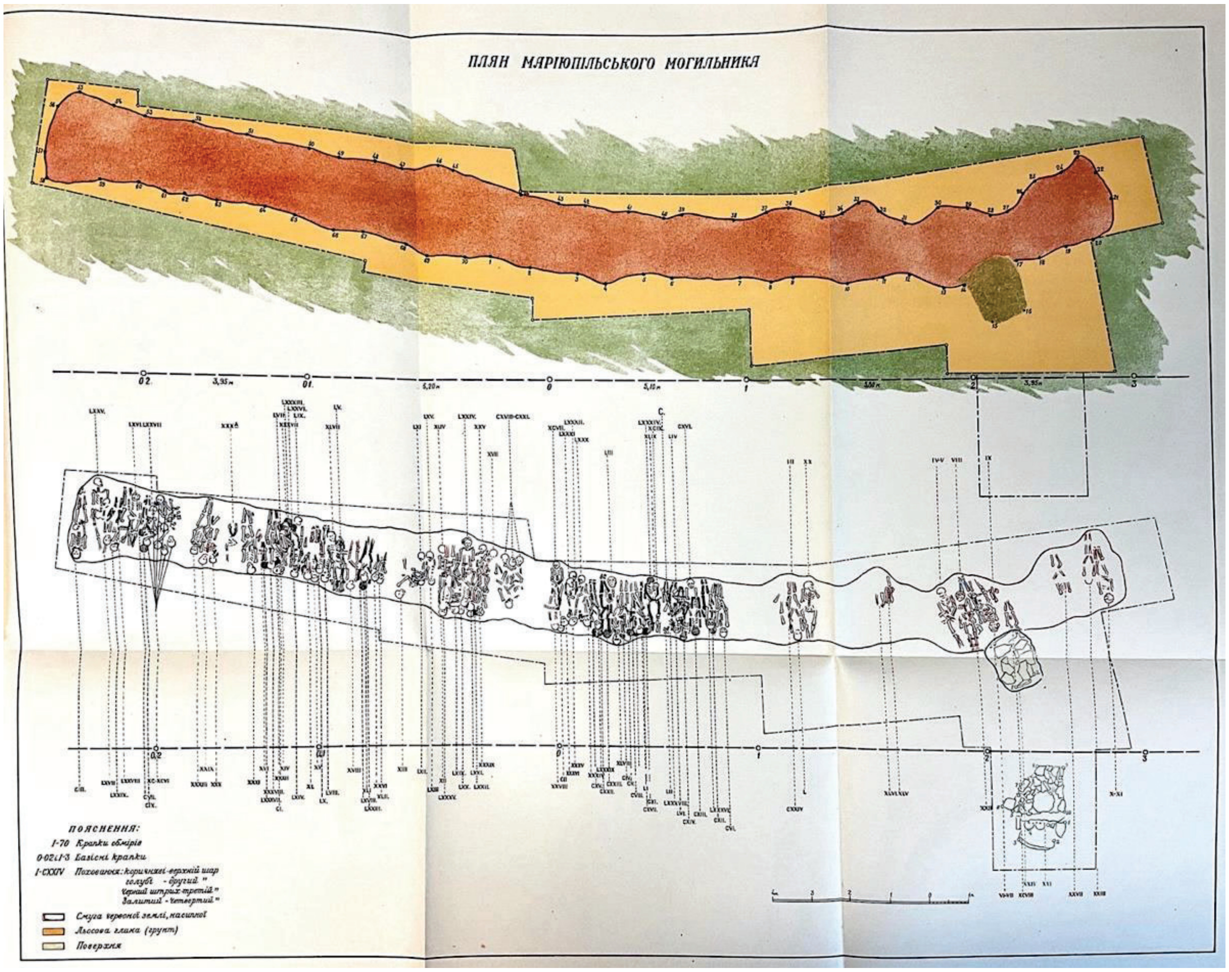

Figure SI1. 12. Neolithic Mariupol Necropolis. Drawing by M. Makarenko, 1933 [1].

I31730

MM50a, Mariupol Necropolis, Burial 50, Skeleton 50a, male (adult), 5474-5236 calBCE (6395±35 BP, PSUAMS-10373, this report)

I31731

MM50b, Mariupol Necropolis, Burial 50, Skeleton 50b (immature), 2204-1986 calBCE (3720±30 BP, BETA-681064, this report, radiocarbon date from tooth enamel carbonate).

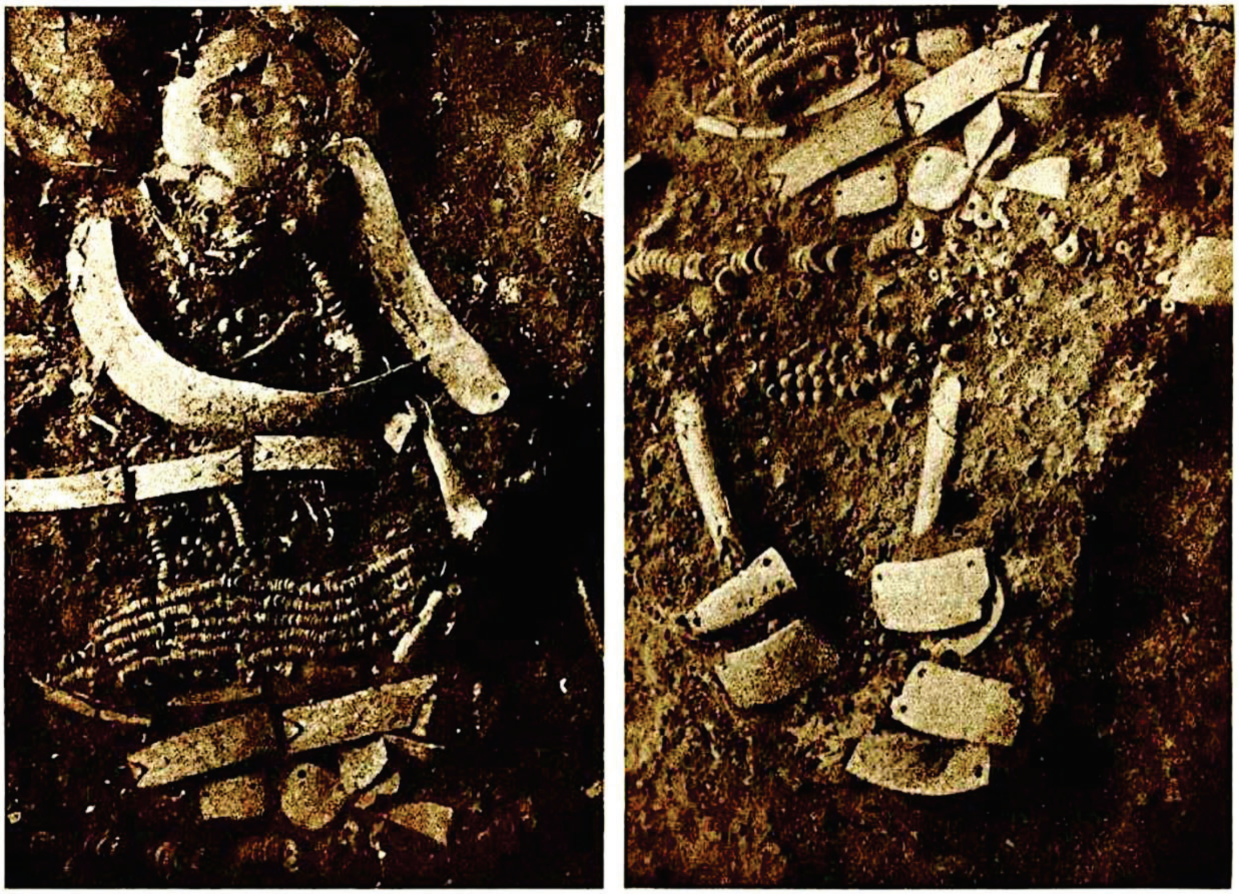

XXVI

ВЕРХНЯ И НИЖНЯ ЧАСТИНА ПОХОВАННЯ L.
UPPER AND LOWER PARTS OF GRAVE L

Figure SI1. 13. Mariupol Necropolis, Burial 50. Photo from[1].

Burial 50 contained a double inhumation of an adult (Burial 50a) and a child (Burial 50b) in an extended supine position, heads to the west. Starting from the sacrum and going down the legs, both skeletons were covered with a mass in the form of clay of a pure purple color. The head of an adult was decorated with a pair of boar tusks so that the sharp ends of the fangs protruded above the forehead. A large boar tusk and a necklace made of mother of pearl cut into disks were found around the neck. The child skeleton, laid on top of the adult, was adorned with boar tusks over the head and neck. A row of narrow plates lay across the chest and around the body. Five necklaces made from mother-of-pearl disks laid on the stomach. The necklaces laid tightly across the skeleton like a belt. Several narrow plates made of boar tusks laid vertically around the right hand, on the line of the strings of the necklace. A belt made of the same narrow boar tusk plates laid below the belt with the necklace. Between them and the rows of small mother-of-pearl necklaces laid a skillfully polished miniature wedge made of green porphyrite, partly covered with rows of necklaces and partly with a row of plates. Below the row of these plates lay wider plates made of the same material, and in between lay a pear-shaped flat pendant with a hole at the narrow end. Further, down to the knees, laid a row of shell beads. Each bead was shaped like a circle with a cut-off segment. On the knees laid two rows of wide boar tusk plates and a scattered mother-of-pearl disks necklace.

I31732

MM.21, Mariupol Necropolis, Burial 21, female, 4715-4548 calBCE (5790±30 BP, PSUAMS-10374, this report)

The burial consisted of a cremation and a double inhumation. The burial was found within a square covered with limestone stones of various sizes, 0.4 m from the sod surface, and between 0.3 m and 0.6 m above the first row of the Neolithic burials. Stones covered the surface with a layer up to 0.4 m in the shape of a crescent. A pile of ashes containing cremated human bones, approximately 0.6 m in diameter, was found in the center of the grave, 0.45 m from the surface. Stones beneath the ashes were burned and covered in soot. A finely knapped arrow or spearhead point with a wide base and traces of fire, an elongated scraper in the form of an arrowhead with finely retouched edges, several boar tusk plates with transverse double incised lines along the shorter edges and without peripheral holes, and a black oblong stone with polished and worn-out edges, were found among the ashes.

The grave was outlined with upright limestone slabs. One large slab was positioned at the eastern edge, surrounded by smaller slabs on the sides. The burial pit was cut into the upper part of the Neolithic burial 98. The two interred individuals lay extended on the back with knees bent and falling to the right side, heads facing east, arms extended over the sacrum bones. The skulls were significantly elevated relative to the postcranial remains and close to each other. A long flint blade was found in the hands of one of the skeletons.

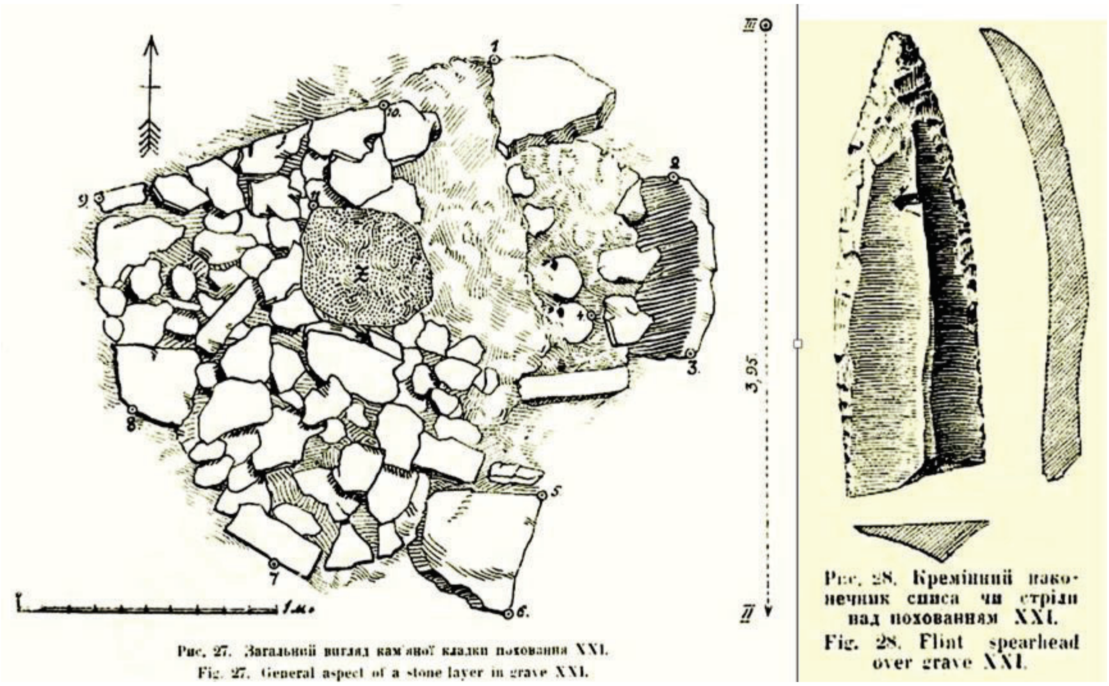

Figure SI1. 14. Left: Mariupol Necropolis Burial 21. Right: a flint arrow or spearhead from the cremation on top of the burial. Images from[1].

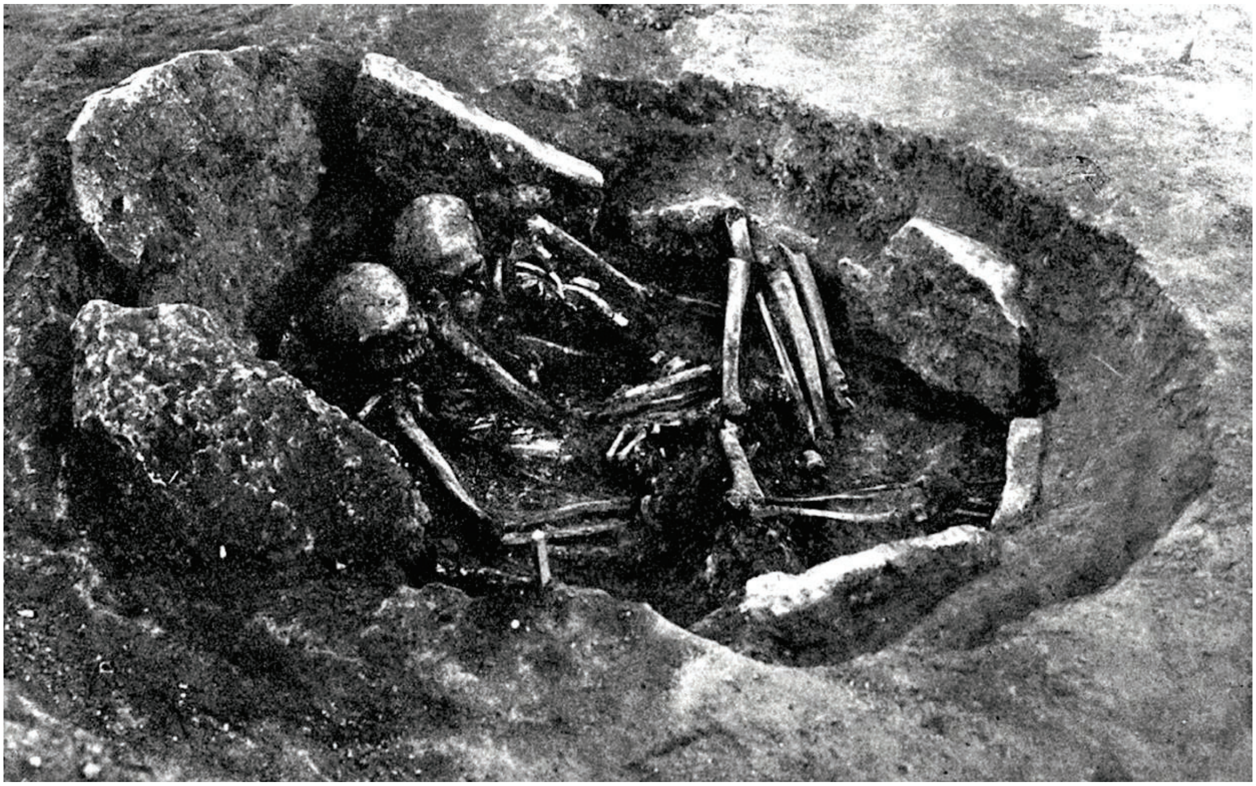
Figure SI1. 15. Mariupol Necropolis Burial 21. Photo by M. Makarenko, 1930.

Mayaky

Belyaiivsky District, Odesa Region (46.397, 30.272)

The Mayaky archaeological complex is located on the southwestern fringe of the Mayaky village on a promontory along the eastern bank of the Dniester River near its convergence with the Dniester Estuary[152]. The 300 × 100 m ancient cemetery situated 200 m north of the network of ditches enclosing a prehistoric sanctuary. Nineteen sites within the cemetery were designated as kurgan or ground-level burial plots [11,76]. Burial positions at the Mayaky necropolis, for the most part, featured left-sided positioning with heads oriented northeastward[11,80]. Artefacts from the Mayaky necropolis reported here were unearthed during archeological in 1965, 1974, and 1986 [11,152,153]. Sanctuary burials 1 and 2 were excavated by V. G. Zbenovich[152]. The Mayaky anthropological collection is housed in the Bioarchaeological Stores of the Institute of Archaeology, National Academy of Sciences of Ukraine, Kyiv, Ukraine, from which it was sampled in 2014. Burial descriptions are from [11,152,154]. Detailed descriptions of burials I12229, I12707, I12705, I12706, and I12710 are also presented in[71].

I12615

Ma6, Mayaki sanctuary, burial 1, female, 5208-4907calBCE (6100±30 BP, BETA-432410[5])

I12228

Ma13, Mayaki sanctuary, burial 2, female, 4486-4350 calBCE (5580±30 BP, BETA-432406[5])

I12229

Mau1, Kurgan 1, Burial 9, female (40-45), 3088-2911 calBCE (4375±25 BP, PSUAMS-7865, this report)

Kurgan 1 was erected over the main burial 9. The burial, containing ceramics of the Usatove type, was centrally located in the kurgan. The burial was made in a rectangular pit with rounded corners, 2.30 × 1.30 m in size, let into the matrix. The depth of the burial pit from the modern surface was 1.65 m^2. The walls of the burial pit were vertical, and the bottom was leveled. The pit was oriented from west to east. The western edge of the pit was destroyed by a depression with a diameter of 0.50 m. The northeastern part of the grave was damaged by a robbers' trench. The remnants of wooden planks found in the filling of the burial pit suggest that the pit had a cover. A stain, formed by organic decay, probably from the bedding on which the deceased was placed, was found at the bottom of the pit.

Only the bones of the legs along with the remnants of the bedding were found *in situ*. Pelvic bones appeared to have been shifted. The cranium was placed on top of the pelvic bones. Disarticulated postcranial skeletal remains (Burial 9.1, female, 92 calBCE - 66 calCE (2020±25 BP, PSUAMS-7792[71]).

Judging by the degree of obliteration of cranial sutures and the wear of the teeth, the individual was 40-45 years old at the time of death. The abrasion of the teeth was continuous. The powerful relief of the cranium and bones of the skeleton led to the male sex assignment by anthropology. On the right side of the braincase of the skull there were a series of stripes of dark violet paint. A similar stripe, crossing the left part of the arch from the back of the head, descended to the eyebrows and under the right eye socket. The left side of the cerebral part of the skull was deformed (plagiocephaly), the right part of the facial skeleton is much smaller than the left. The deformity was created by systematically placing the subject as an infant on his left side. The cracks on the left parietal bone of the skull are traces of blows inflicted on the subject, possibly causing death [155].

I12227

Mayaky, Kurgan 6, Burial 1, female (16-18), 3955-3783 calBCE (5060±30 BP, BETA 441234[5])

Kurgan 6 is the northernmost in the burial ground. The mound has not been preserved. The kurgan had a trench with a diameter of 15-17 m, a width of 0.4-0.5 m, and a depth of 1.55 m. The walls of the trench were vertical, the bottom is flat, and the fill is yellowish-gray loam. In places, black soil with loam inclusions lay on the bottom.

Burial 1: The grave pit is rectangular (1.6 x 1.35 cm) with a depth of 0.6-1.35 m. Up to a depth of 1.2 m, the grave was filled with exceptionally dense grayish-brown loam with carbonate inclusions ("white eyes"), and below that, with dense black soil with loam inclusions. The flexed skeleton lay on the back turned onto her left side, with the skull oriented east-northeast (64°). The hands were raised to the face, with the fingers of the right hand positioned as if pinching, and the wrist turned at a right angle. On all the bones and on the bottom of the grave beneath them, there was decay with spots of dark purple paint. On the metaphyses of the long bones of the limbs, the decay and paint were deposited in transverse stripes 2.5-3 cm wide, spaced 1-1.5 cm apart.

Burial inventory: (1) A pot with cord impressions, with a layer of soot on the outside; (2) An amphora with soot and red ochre spots on the outer walls, containing a 3 cm long wooden stick inside; (3) A pot (the bottom of the grave beneath it was sprinkled with red ochre), with red ochre spots on the outside and a brown deposit inside; (4) An inverted bowl covering a decayed

piece of wood; (5-6) Bowls; (7) A fragment of a light-gray anthropomorphic figurine with engraved ornamentation, with sand grains in the ceramic paste, lying face down with the head to the northeast.

All the pottery was light brown but of different shades, with the color becoming more intense with the increasing size of the items. The ceramic paste contained sand grains and finely crushed shells. Vessels 2-4 were burnished.

Low-resolution PCR-SNP analysis of the diagnostic coding and hypervariable I (HVR-1) regions of mtDNA followed by Sanger sequencing conducted in the Molecular Archaeology Lab at GVSU, following a protocol detailed in[28,35] produced an A13866G coding region transition in one of the amplifications, suggesting the specimen's mtDNA may belong to haplogroup HV20.

I12704

Mayaky, Kurgan 7, Burial 2, male (25-30), 3620-3030 calBCE (RE-adjusted) (5144±20 BP, weighted mean[10] of 5530±32 BP, OxA-22959[12]; 5390±30 BP, BETA-441235[5]; and 5545±40 BP, PSUAMS-7793, this report).

Burial arrangements 7, 8, and 9, identified as kurgans with missing mounds, were found in the northern part of the Mayaky burial complex, situated next to each other. The southeast side of each kurgan was surrounded by a 0.4-0.5 × 0.6-0.65 m ditch. It is likely that the three kurgans were erected in a chronological sequence, with kurgan 7 appearing first, followed by kurgan 9, and, subsequently, kurgan 8[71].

Burial 2 of Kurgan 7 was classified as belonging to the Usatove group through archaeological analysis. The grave was discerned as a discoloration in the transition to sandy loam, situated 0.65 m beneath the current surface. Its depth extended to 1.25 meters, with traceable walls reaching 0.6 meters. The oval-shaped burial pit, measuring 1.4 × 1.15 m, exhibited a slight elongation at the bottom (1.55 × 1.15 m), aligned from southwest to northeast. The backfill comprised three distinct layers: black earth with sandy loam lenses up to a depth of 1.05 m, followed by a thin 0.1-m layer of sandy loam, and black earth with numerous lime inclusions at the base.

Within the grave lay the robust skeleton, positioned in a contracted manner on the left side, with the skull oriented towards the east and facing upwards. The arms were bent and crossed over the chest, with the left hand under the skull and the right hand resting by the facial bones. Traces of raspberry-colored ochre remained on the skull, joints of the thighs, and lower legs, accompanied by remnants of organic material resembling brown mold on the lower legs. Dark red ochre crumbs were discovered between the upper ribs.

Burial inventory consisted of two vessels, found next to the arms of the interred. One, a beige beaker standing 46 mm tall with a rim diameter of 45 mm, featured a short body and a low neck. The vessel was ornamented with four horizontal rows of fine cord coated with red ochre around the neck, while incised lines decorated the body, with a crisscross pattern forming a six-pointed star in the upper region and three parallel grooves at the bottom. The clay composition included finely crushed shells. The second vessel, a heavily fragmented miniature bowl measuring 72 mm in height with a rim diameter of 114 mm and a bottom diameter of 38 mm, typified Usatove pottery. It boasted an S-shaped profile, thin walls, and round cord indentations along the rim, complemented by three rows of thin cord indentations underneath. The interior was coated with red ochre, and the shard exhibited a gray-brown hue, with the temper comprising finely crushed shells and grains of sand.

I12710

Mau12, Kurgan 8, Burial 4, female (*ca.* 60), 3769-3196 calBCE, RE-adjusted (5295±30 BP, PSUAMS-7862, this report)

Burial 4 of Kurgan 8 was discovered within an oval pit, featuring a 0.30-m-high lining and depths ranging from 0.8 to 1.6 m. The dimensions of the grave measured 1.2 by 0.9 m at the top and 1.3 by 0.9 m at the bottom. The grave fill consisted of two layers: compact sierozem containing calcareous nodules overlaying a base layer of continental loess-like loam.

Within the grave, approximately 0.4 to 0.5 m above the bottom, were discovered fragments of horse, cow, and sheep bones covered with dark red ochre, along with two shards and a fragmented light brown Usatove bowl. The bowl displayed coarse molding with notches, impressions of a round-ended stamp, and cord markings, with sand grains mixed into the clay.

At the bottom of the pit lay the contracted skeleton, positioned on the left side with the skull facing north-northeast at an angle of 33 degrees, and hands bent. The left hand was near the face, while the right hand rested under the cervical vertebrae. Stripes of crimson ochre marked the skull vault, with similar markings found on pelvic bones. Evidence of brown decay was noted on the wrist bones and lower epiphyses of the forearm on the right hand, as well as in the region of the left elbow joint.

Burial inventory consisted of five ceramic vessels located alongside the deceased. These included the lower part of a vessel crafted from clay mixed with crushed shells, exhibiting soot stains on the walls; a polished pot featuring imprints of a cord and a pipe, with holes below the rim and sand grains within the clay; a polished bowl containing finely ground shells and sand grains in the clay; a polished amphora adorned with holes beneath the rim and a corded design; and a pot made from a crumbly clay mixture with crushed shells, displaying a pattern of pipe imprints.

I12707

Mau9, Kurgan 8, Burial 6, Skeleton 2, female (25-35), 3511-2917 calBCE, RE-adjusted (5330±40 BP, PSUAMS-7794, this report)

The burial pit for Burial 6 reached depths of 0.75 to 1.50 m, featuring a lining and a ledge. The oval-shaped burial chamber measured 1.3 × 0.9 m at the top and 1.35 × 1.10 m at the bottom. Within this grave, two individuals were interred: a 40 to 55-year-old male (Skeleton 1) and a 25 to 35-year-old female (Skeleton 2).

Skeleton 1, dated to 4229-3656 calBCE, RE-adjusted (5670±30 BP, BETA-4324085[5]), was found at the upper layer of the grave. Beneath a 0.3 m-thick layer of yellow loess-like loam, indistinguishable from the matrix, lay Skeleton 2. This second individual was positioned in a contracted manner on the left side, with the skull facing eastward at an angle of 85 degrees. Notably, the spine exhibited a pronounced curvature, the femurs pressed against the chest, knees brought towards the shoulder joints, and feet positioned near the pelvis. A pot was placed within the hands of the buried individual. Decay stripes with black paint spots were observed on the postcranial skeleton and the ground beneath them, with traces of bright crimson ochre noted on the right parietal bone.

The burial inventory associated with Skeleton 2 included a pot crafted from gray crumbly clay mix, featuring flat stamp impressions and containing a flint flake; a vessel made of a loose clay mix with weak firing featuring two protruding protrusions on the body, adorned with cord and round-ended stamp imprints; and a bowl coated on the outside with a layer of greasy soot.

These latter two vessels displayed gray and light brown hues, with crushed shells incorporated into the clay mix.

I12701
Ma11, Kurgan 9, Burial 1, female (infant-subadult), 3011-2881 calBCE (4300±30 (BETA 432412)[5]

I12706
Mau8, Kurgan 9, Burial 2, female (mature), 3947-3519 calBCE, RE-adjusted (5444±19 BP, weighted mean[10] of 5471±24 BP, 0xA-22960[12], and 5400±30 BP, PSUAMS-7846, this report)

Kurgan 9 had an arc-shaped trench 0.4-0.5 m wide and 0.6 m deep. The mound has not been preserved. During the 1986 excavations of Kurgan 9, two burials were unearthed. Burial 2 was tentatively linked to Usatove due to the absence of burial inventory, while Burial 1 was considered to be from a different time period. Subsequent excavations revealed two additional burials, designated as 3 and 4, with the latter being a double interment.

Burial 2 was identified as a discoloration located at the interface with sandy loam, approximately 0.45 m below the present-day surface. The rectangular pit exhibited rounded corners, almost resembling an oval shape, measuring 1.6 × 1.5 m along a southwest-northeast axis. The walls of the pit were discernible up to a height of 0.6 m, with a bottom depth of 1.05 m. Notably, the southeast wall of the pit featured a 0.5 m-deep and 0.3 m-wide step. The backfill material was compact, gray-brown, sandy-loamy, with sprinklings of lime. A mature adult in a contracted position lay on the left side with the skull oriented northeastward, arms flexed, and hands positioned in front of the face. Traces of raspberry-colored ochre were discovered on various skeletal elements, including the right shoulder bone, lower epiphysis of the right shoulder bone, pelvis, and legs. Additionally, patches of brown-colored organic material were detected on the long bones.

Ma12
Kurgan 10, Burial 2, Skeleton 1, female (>60), 4033-3534 calBCE, RE-adjusted (5530±30 BP, BETA-432413[5])

I1423/ I12705
MAJ1/Mau7, Kurgan 10, Burial 2, Skeleton 2, male (50-55), 3936-3372 calBCE, RE-adjusted (5390±30 BP, PSUAMS-7845, this report)

Kurgan 10 presented as a mound of chernozem soil measuring 0.15 m in height and approximately 20 m in diameter. Among the five burials designated as Usatove, two had incurred damage from pits dating back to the early centuries AD. These graves were filled with dense humus loam.

Burial 2 was housed within an oval-shaped grave pit, measuring 1.3-0.85 × 0.5-0.65 × 0.8 m. Within this burial site lay two individuals. Skeleton 1, identified anthropologically as a female aged over 60 years, was positioned on the left side, with Skeleton 2, a male aged 50–55 years, laid atop Skeleton 1, with the skull resting between the female's shoulder bones and chest. Both skeletons were oriented with their skulls facing east-southeast. The female's arms were

folded, with her left hand positioned in front of her face and her right hand near her knees. The male's arms were bent, and his hands were raised to his face.

The inventory associated with Skeleton 1 included a fragment of a coarse bowl crafted from dark gray clay with sand. Skeleton 2 inventory comprised a brown vessel featuring imprints of a semicircular stamp, along with a loose ceramic mix containing grains of sand.

Low-resolution mtDNA typing was conducted in the Molecular Archaeology Lab at GVSU, following the protocol detailed in[28,35].

Molyukhiv Bugor

Chygyryn district, Cherkasy region (49.134, 32.479)

Molyukhiv Bugor is a multilayer settlement and cemetery. The site is located in the floodplain of the Tyasmin River to the north of the Novoselytsia village. The settlement is located on a sand dune, which has the appearance of an irregular acute triangle, stretched almost exactly from south to north, its length is about 90 m, and the width of the base is about 60 m. It was first excavated in 1950 by O.I. Terenozhkin and subsequently researched in 1955-1956 by V.M. Danylenko. Since 1992, the site has been excavated by T. M. Neradenko [156].

During the excavations, 1,650 m^2 of the area of the site were investigated and the following objects were discovered: a "moat", which encircled the elevated part of the settlement, divided it into two sections, headed to the north of the settlement to the low floodplain and performed certain protective and economic functions; "pillars" in the lowered parts of the sand dune, in the west and north of the settlement, which were probably the basis of light structures above the water; 44 household pits from different historical periods — Neolithic, Eneolithic, Early Iron Age, modern times; four semi-oval-shaped structures barely buried into the ground, stone implements, items of bone and horn, copper products, and other finds. A massive amount of osteological material was found, consisting of more than 172,000 bones of wild and domestic animals, rodents, turtles, birds, fish, mollusks, etc.

Potentially three asynchronous settlements existed on the site during the Neolithic (5400-4800 BCE). In the Eneolithic era, there were possibly three asynchronous settlements on Molyukhiv Bugor as well, dating to ca. 4350-4200 BCE, 4200-4000 BCE, and 3950-3500 BCE. Among the remains of animals in Eneolithic settlements, finds of bones of a domestic and forest cat, a camel, a lion, a leopard, a stone marten, an otter, a ferret, and a hedgehog, which are not known among the osteological materials of other contemporary monuments, are noteworthy.

The first burial on the site was excavated in 1955 by V.M. Danylenko. The interred was crouched on the left side, oriented to the east. A bone buckle with two holes of different diameters of the log type was found in the belt area. In 1999-2005, during the archaeological research of the Neolithic-Eneolithic settlement, six more burials were discovered on its territory[18].

I1454

MOB3, burial 4, Skeleton 2, female, 2286-1886 calBCE (3680+70 BP, Ki-14236)

The double Burial 4 was discovered in 2004 on the western outskirts of the settlement in squares 6A-7A of trench IV at a depth of 70-72 cm from the surface. It was located beyond the "moat" near the place where the "ditch" was interrupted. Excavations proved that the burial, like the other burials on the site, was made in the cultural layer of the settlement. During the cleaning of the area and around it, the following were found: 13 small fragments of Neolithic pottery with

grass and incised ornamentation, 5 small fragments of Neolithic pottery with sand and various ornaments, 1 large fragment of Neolithic pottery with grass and sand with incised lines, 17 fragments of porous Eneolithic pottery, of which 15 were without ornamentation and 2 with cord patterns, 1 small piece of modeling clay, 1 small piece of coarse-grained granite, 4 quartzite flakes, 2 sandstone flakes, 14 flint flakes, 1 flint scraper and its fragment, 1 fragment of a flint knife-like blade with retouch on one edge, and 1 flint core-like fragment.
Skeleton 1 laid on the back, facing north. The right arm was bent at the elbow downward and touched the left hand of Skeleton 2. The left arm was also bent at the elbow upward and placed below the head, which was turned toward Skeleton 2. The legs were slightly bent at the knees. No accompanying artifacts were found. Skeleton 2, slightly smaller in size than Skeleton 1, laid on the back, with the left hand stretched toward Skeleton 1 and touching their right hand. The head was turned toward Skeleton 1. The right arm was bent at the elbow and placed on the abdomen. The legs were strongly bent at the knees and rested on the right leg of the first buried person. No accompanying artifacts were found. The preservation of the skeletons was satisfactory.

Cleaning the bottom of the squares at the burial level revealed the outlines of the burial pit with rounded corners, and the dark-gray sandy loam resulted from the filling of the burial with humus sand from the cultural layer of the settlement, clearly standing out in the light-gray subsoil sand. During the creation of the double Burial 4, an earlier Burial 6 was destroyed. On the map in[18], both burials are marked as № 4.

I1424
MOB1, Burial 6, male, 3777-3648 calBCE (4943±28 (weighted mean[10] of 5020±50 (OxA-17502), and 4909±33 (OxA-17503)[15])

Burial 6 was located on the western outskirts of the settlement, beyond the "moat," near the place where it was interrupted. Judging by the remains of the burial, it was an inhumation, oriented to the southeast, with legs bent at the knees, arms along the body, and hands near the pelvis. The bones of this burial were found in several surrounding squares, and many bones were missing, including the skull. Only the lower part of the skeleton, the pelvis and legs, remained in anatomical order. The leg bones of Burial 6 were located near the bones of the left leg of Skeleton 1 from Burial 4. The level of Burial 6 is 5-6 cm higher than Burial 4. Like all the other burials at the site, Burial 6 was made in the cultural layer of the settlement.

Mykhailivka
Kherson Region, Beryslavskyi District (47.315, 33.959)

The Mykhailivka multi-layer settlement is located 4 km south of the village with an eponymous name in the Beryslavskyi district of the Kherson region. It occupied two hills and partly a third, located across the ravine, as well as the marginal part of the adjacent plateau on the west bank of the Pidpilna River, a western tributary of the Dnipro. The slopes of the plateau were cut by deep ravines. On the high bank, two adjacent hills stood out, rising about 30 m above the level of the Dnipro. The largest (central) hill designated by researchers as Mykhailivka I came close to the bank of the river. One of the ravines separated the central hill from another, smaller one, on which the second settlement, Mykhailivka II, was located, at a distance of 50 m from Mykhailivka I. The area of the central hill was more than 0.5 ha, the height above the level of the

Dnipro in the southeastern part was 23–25 m and almost 30 m in the northwestern part. Its greatest length is 100 m, the width on the north side is 50 m, south - 20 m.

The Mykhailivka archaeological site was studied by 1952-55, 1960, and 1963 expeditions of the Institute of Archaeology, National Academy of Science of Ukraine, led by E.F. Lagodovskaya and O.G. Shaposhnikova [21,157].

The total reconstructed area of the entire Mykhailivka settlement site is about two hectares. Geological cross-sections carried out in different places and directions of the site uncovered three cultural layers, of which the lower (third) was separated from the two upper (second and first) by a sterile horizon. The lower layer of Mykhailivka is dated to ca. 4250-3550 BCE, based on two sets of dates from dwellings 3 and 4. Dwelling 4 dates to 4247-3988 calBCE (5290±43 BP, weighted mean of 5320±60 BP, Ki-9486, and 5260±60 BP, Ki-9487) and dwelling 3 dates to 3912-3540 calBCE (4921±53 BP, weighted mean of 4890±80 BP, Ki-8011, and 4945±70 BP, Ki-8182). The lower layer at the site is associated with the Lower Mykhailivka archaeological culture [61].

The upper two layers, associated with the different phases of the Yamna archaeological complex, formed a continuous chronological sequence. The top layer contained artifacts from the Catacomb archaeological complex. Cultural remains occurred mainly in chernozem and humus loam. However, deepened ground dwellings and dugouts were also traced in the upper layers of light loam. The lower horizon of the middle layer is dated to 3646-3348 calBCE (4710±80 BP, Ki-8012) and the upper horizon is dated to 3371-3026 calBCE (4519±53 BP, weighted mean of 4480±70 BP, Ki-8186, and 4570±80 BP, Ki-8010).

I32534

M1P1.6, Mykhailivka I, square VI, female, 3635-3383 calBCE (4755±25 BP, PSUAMS-10750, this report)

A single molar from excavation square VI of the Mykhailivka I (central) settlement. Excavations 1952. Based on the ^{14}C dating of the settlement layers, the specimen comes from the lower horizon of the second (middle) layer, associated with the early Yamna phase of the site’s occupation.

Liubasha Kurgan (Kurgan 2)

Novohryhorivka, Odesa Region, Mykolaivskyi District (47.557, 30.3143)

Archaeological excavations near the Novohryhorivka village were conducted by the expedition of the Department of Archeology of the Northwestern Black Sea Region of the Institute of Archeology of the National Academy of Sciences of Ukraine. The "Liubasha" kurgan, or Kurgan 2, was located 2 km north of the village Novohryhorivka. The kurgan was located on the plateau of the east bank of the Tiligul River, at the edge of the highest watershed plane in the area, stretching from the southeast to the northwest between Tiligul and the Gluboky Yar ravine. Other kurgans on the same section of the plateau are located 1.13 km southeast and 2.5 km northwest of Liubasha. In total, 17 burials were found in the Liubasha kurgan. Six belonged to the Yamna archaeological complex (Burials 2, 7, 8, 10, 16, 19), and three were of the Catacomb archaeological complex (Burials 3, 9, 11, 12, 15). Five burials (3, 9, 11-12, 15) belonged to the Babyne (Multi-Cordoned Ware) culture complex. Burial 5 belonged to the Sabatynivka group. Burial 14 was from the Sarmatian period and Burials 6 and 13 were of an

indeterminate cultural affiliation. Patinated processed flints likely of a Paleolithic origin were found in the kurgan's embarkment[13].

I12234

Liubasha Kurgan, Burial 3, male, adult, 1499-1127 calBCE (3080±70 BP, Ki-11176[13])

Burial 3 (Babyne archaeological complex) was discovered 10.5 m southwest of the central benchmark, at a depth of 1.5 m. The walls of the burial pit have not been traced. The burial was covered with a platform of planks, laid longitudinally directly on the body or, less likely, settled on the skeleton and bottom without any intermediate layer. There were five or six planks on the platform, the ends of three have been preserved. The semicircular bark (unpeeled) side was facing down. The interred (adult) lay contracted on the left side, with the head to the east. The legs were strongly bent at the knee and hip joints, the left knee was pulled towards the chest. The arms were also bent, the hands were in front of the face. Bone tissue was brittle, grey-blue in color on the limb bones, possibly as a result of thermal exposure. The level at which the skeleton was found was distinguished by a greenish clay coating with one well-preserved smooth edge, interspersed with coals and light firing. Outside the coating, traces of three pits with a diameter of 2–3 cm with wood decay were found, possibly from the racks of some then a light canopy. The radiocarbon age of the burial was established from fragments of bones of the lower extremities.

I7925

Liubasha kurgan, Burial 9, male, adult, 2119-1624 calBCE (3520±80 BP, Ki-11173[13])

Burial 9 (Babyne archaeological complex) was found 16.3 m west of the reference benchmark, at a depth of 4.3 m, under dense silty deposits that accumulated at the edge of the kurgan. The pit was oval in plan (1.56 × 0.93 m), trough-shaped in cross-section, with a depth of 0.15 m (-4.45 m), oriented in the north-south direction. The fill was dense and silty. The skeleton was located under the eastern wall of the pit. The interred individual was lying in a flexed position on the left side, with the head facing south. The arms were stretched along the body, with the hands joined and touching the left thigh. The radiocarbon date of the burial was established based on fragments of leg bones.

I12235

Liubasha kurgan, Burial 11, male, adult, 1686-1311 calBCE (3230±70 BP, Ki-17332[13])

Burial 11 (Babyne archaeological complex) was found 20.3 m southeast of the reference benchmark, at a depth of 4.2 m. The clayey fill of the pit did not differ from the surrounding soil. The oval pit was traced at the bottom: length 1.42 m, width 0.89 m, depth 0.15 m from the level of discovery (-4.35 m). There was a faint white decay on the northern wall of the pit. The wall was not traced at the head end. The interred individual, oriented with the head to the northeast, was lying in a flexed position on the left side. The right arm was bent at the elbow, with the hand positioned in front of the face. The left arm was bent so that the elbow was positioned behind the back, and the hand was placed under the pelvis. The skull was destroyed, with only small fragments of flat bones and a few teeth preserved. Near the skull, a small patinated flint fragment was found.

I7925(d)

Liubasha kurgan, Burial 12, male, adolescent, ca. 2120-1620 BCE (based on I7925 twin)

Burial 12 (Babyne archaeological complex) was found 23.8 m south of the central benchmark, at a depth of 4.05 m. The burial pit was not traced. The skeleton was discovered in clay loam under a dense layer of silt that filled a ground depression formed during the construction of the burial mound. The buried individual (an adolescent) was in a tightly crouched position on the left side, with the head oriented towards the southeast. The arms were bent at the elbows, with the hands brought close to the face of the skull. Genetic analysis revealed that individuals in burials 9 and 12 were twins.

I16674
Liubasha kurgan, Burial 15, male, 2434-1943 calBCE (3740±70 BP, Ki-11201[13])

Burial 15 (Babyne archaeological complex) was situated 24 m southeast of the reference benchmark, at a depth of 4.43 m. The oval pit, measuring 1.2 × 0.8 m, was filled with chernozem and oriented in a northeast-southwest direction. A disarticulated skeleton was located in the middle of the pit, densely folded in a compact "package". The skull was positioned in the southwestern part of this arrangement, resting atop the cervical vertebrae in an upright position before being crushed; the occipital portion of the skull was absent. Amidst the central cluster were the ribs, with one arm bone beneath them. Adjacent to the eastern wall, the bones of the arms were neatly folded, followed by the legs and then the pelvis atop them. Close to the skull at the pit's base, grains of pink ochre and a flint flake were discovered. The burial inventory comprised a light brown flint flake, lacking patina.

I12233
L2.16, Liubasha Kurgan, Burial 16, male, adult, 3074-2888 calBCE (43330±35 BP, PSUAMS-7790, this report)

The burial chamber of Burial 16 (Yamna archaeological complex) was 0.88 m wide, was covered by timber planks. In the decay under the skeleton, longitudinal and transverse fibers (stems of some plants) were clearly visible. The interred lay on the back, head to the west, arms extended along the body. Legs were preserved above the knees. It could be determined that they were bent with the knees to the right. The skeleton was poorly preserved. To the right of the skull, a lump of dark ochre, egg-shaped, about 3 cm high, was found.

Odesa Kurgan
Odesa (46.488, 30.721)

The Odesa Kurgan was studied in 1912-13 by M. F. Shkadyshko and A. V. Dobrovolsky. A detailed analysis of kurgan's stratigraphy was published by V. G. Zbenovich and A. M. Leskov in 1965. Analysis of the stratigraphy of the Odesa kurgan shows the presence of the main and three additional embankments. The main mound was erected over Burial 11. Burial 10 and Burial 7, burials in stone cists, later destroyed, were subsequently added into the main mound. Yamna burials located both in the center and along the kurgan's circumference are probably associated with two subsequent embarkments. Catacomb and Babyne burials, as well as burials of the Late Bronze Age period, were subsequently added into the formed mound of the kurgan[158].

I11850
ODK1, Odesa kurgan, Burial 10, female, 2874-2630 calBCE (4150±25 BP, PSUAMS-11224, this report)

Burial 10, possibly of Catacomb archaeological complex, was lowered into the main mound of the kurgan. The burial pit was covered by a stone slab. The interred was in a contracted position on the back with legs falling to the right side. A stone circle was arranged around the burial.

Ogrin-8 (Igren-8)
Dnipro, Dnipropetrovsk Region (48.444, 35.116)

The archaeological site of Ogrin-8 was settled since the Mesolithic. The earliest occupation phase at the site is connected with the Kukrek archaeological group and dates to the early Holocene[159]. A ground multi-layer cemetery (Neolithic-Late Bronze Age) Ogrin-8 was located on the northern side of the Ogrin' Peninsula at the confluence of the Dnipro and Samara rivers. Excavated in 1932 by M. O. Miller. The next phase of excavations at the cemetery took place in 1945-46 by A. V. Dobrovolsky. In 1974-76, 1978, and 1986, excavations at the cemetery were carried out by D.Ya. Telegin [160].

I2108
Igren-8 9, Burial 20, female (infant), 4340-4178 calBCE (5415±25 BP, PSUAMS-8220, this report)

I1924/ I27935
Igren-8, bag 11, burial 24, female (30-40), 3971-3802 calBCE (5105±25 BP, PSUAMS-8219, this report)
Excavations 1978.

I27930
Igren 8-74 - Individual 3a, male (young adult), 4400-4000 BCE.
Excavations 1974-78. One of the two adult individuals in a double burial at the depth of 0.9-1.0 m, on the back with raised knees, ESE orientation, ochre, and three flint blades between the shoulders.

Revova Kurgan 3
Odesa Region, Shyriaivskyi District (47.268, 30.321)

Kurgans 3 and 4 were investigated in the vicinity of the village of Revova in 2003 by the expedition of the Department of Archeology of the Northwestern Black Sea Region of the Institute of Archeology of the National Academy of Sciences of Ukraine. The kurgans were located on the east side of the Velykyij Kuyalnik river valley, at an altitude of 84 m above the thalweg of the river (125 m in the Baltic system of heights). They occupied the upper part of the slope of the southwestern exposure, gently descending to the river, which is 1.5 km from the kurgans.

Kurgan 3 had a complex organization. It started as an Eneolithic ritual complex (sanctuary). Prior to building the initial sanctuary, the topsoil was cleared, and the ground leveled, leaving the center of construction rising slightly compared to the rest of the surface. Around this elevation, a semicircular ditch about 12 m in diameter was dug. The earth from the ditch was used to build a platform, 12 m in diameter, and 1 m high. It appears that the pit for the main Eneolithic burial 19 was already laid out prior to erecting the platform. In the next construction step, the platform was tiled around with limestone, quarried, apparently, 4 km away from the site. Limestone was sometimes laid in two or three layers; the largest pieces were found surrounding the eastern side of the platform. The final stone mosaic resembled in its outline the shape of a turtle. The whole construction was eventually covered with soil, making it difficult to tell whether it was intentional or was the result of a natural process.

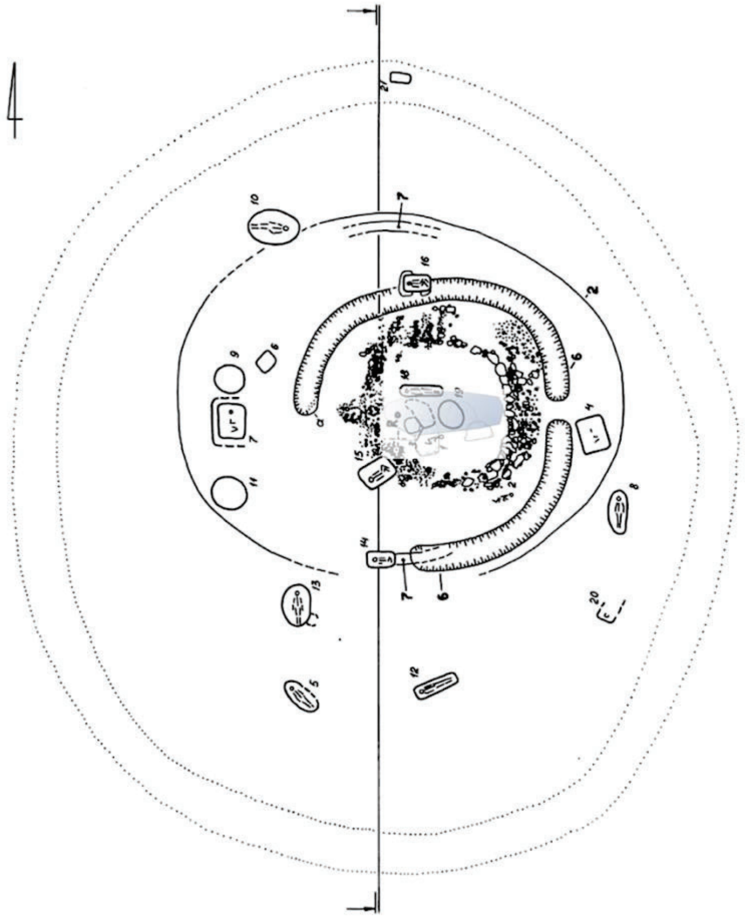

Figure SI1. 16. Revova Kurgan 3 showing a reconstruction of a dolmen over the main Burial 19. Drawing by S. Ivanova and A. G. Nikitin.

Two ritual pits/postholes 9 and 11 to the west of the primary mound likely belonged to the same Eneolithic complex. According to stratigraphy, the complex was completed by an addition of loam, or mound 2, about 18–22 m in diameter.

Burials 3 and 16 (Yamna archaeological complex) were lowered into the central part of the mound from the level of mound 2, later almost completely destroyed by digging. Burials 3 and 16 were covered with large stone slabs (stelae), two in each burial. There was a central cover slab over burial 3, which was likely destroyed by a robber trench. The slabs from burials 3 and 16 were possibly part of a dolmen erected on top of burial 19, and subsequently re-purposed in later burials [161]. The original dolmen's capstone may have been used as a central slab in burial 3.

I16668

REV-3.10, Revova Kurgan 3, Burial 10, male, 2700-2000 BCE

Burial 10 (Catacomb archaeological complex) was found 11 m northwest of the central benchmark, at a depth of 1.5 m. It was identified by a dark oval-shaped spot. The dimensions of the grave pit at the top were 2.6 × 1.8 m, at the bottom – 2.5 × 1.68 m, the traced depth is 0.93–0.95 m (2.43–2.45 m). The southern wall was made in the form of a lining, the northern wall was beveled towards the bottom at an angle. Stone chips were found at the bottom of the burial

chamber. The interred was located closer to the southern wall, in the extended supine position on the back, with the head oriented to the east. The right arm extended along the body, the left arm was bent at the elbow, the hand covering the left hip joint. All that was left of the legs were the femurs and part of the left tibia. There was a faint decay from an organic mat under the skeleton.

I16669

REV-3.13, Revova Kurgan 3, Burial 13, 2581-2210 calBCE (3940±60 BP, Ki-11172)

Burial 13 (Catacomb archaeological complex) was located 8.7 m south-southwest of the central benchmark, at a depth of 1.9 m. A ground catacomb with a collapsed vault was distinguished on the natural ground by its dark fill. The burial 2.1 X 1.5 m chamber had an oval shape and a traced depth of 1.1 m (total depth 3 m). A rounded entrance shaft adjoined the burial chamber from the southeast side. Its diameter was 0.7 m, with a traced depth of 0.5 m. The deceased lay on the back in an extended position, with the head oriented north-northwest. The skull was tilted toward the right shoulder, facing south. The spine was bent. Near the eastern wall, at the level of the forearm, there was a ceramic bowl placed on a layer of burgundy ochre. The bowl's side handles were directed south. Behind the crown of the skull, there were three flints, and another one was located near the left forearm. Dark brown decay of the bedding was traced at the bottom of the burial chamber. The burial inventory consisted of a bowl and a set of flints, including two fragments and two flakes, small to medium in size, with a small area of retouch on the ventral side. The bowl had a rounded bottom and a neatly cut rim, decorated with a composition of grooves and incised lines. The ornament, starting below the rim, included a belt of six grooves and six descending triple festoons. The free spaces between the festoons were filled with rows of worn parallel grooves, applied with a serrated spatula, creating a ribbed texture. The ornament was completed with a pair of horizontal cylindrical protrusions ("spouts"), centered with perforations. The protrusions were placed asymmetrically, with one slightly higher than the other. Below them, between the festoons, was an elongated conical figure formed by three grooves, resembling another festoon but in the opposite direction. The surface of the bowl was light brown, spotted, and polished in places, with the bottom part blackened by soot. The ceramic material of the sherd was dark gray on the break, with inclusions of sand and chamotte. Both the inner and outer surfaces showed traces of smoothing and streaks left by a tool, such as a comb or serrated spatula, on the raw clay. These traces were rougher on the outside, especially near the bottom. The vessel's height was 16.5 cm, and its diameter was 18.5 cm. The radiocarbon date of this complex was established at the Kyiv Radiocarbon Laboratory using samples from fragments of large limb bones.

I16670

REV-3.14, Revova Kurgan 3, Burial 14, 2139-1748 calBCE (3590±70 BP, Ki-11175)

Burial 14 (Babyne archaeological complex) was discovered by fragments of a transverse wooden cover, from which brown decay was preserved in the central part of the pit directly on the bones of the skeleton. The burial was located 6.5 m south of the central benchmark, at a depth of 1 m. The bottom of the burial chamber was partially traced, identified by the decayed brown bedding. The 1.35 × 0.8 m chamber was likely rectangular with strongly rounded corners. The walls were traced to a height of 0.15 m (total depth 1.15 m). The crouched skeleton lay on the left side, oriented west-southwest. The left humerus was parallel to the main axis of the skeleton, and the right humerus was directed toward the left. Other arm bones were not preserved. The burial was sprinkled with scarlet ochre (deposits on the bottom and bones). The

radiocarbon age of the complex was determined at the Kyiv Radiocarbon Laboratory using samples from fragments of limb bones.

I7928

R3.16, Revova Kurgan 3, Burial 16, 2885-2501 calBCE (4135 ± 60 BP, Ki-11059)[13].

Burial 16 (Yamna archaeological complex) was found at a distance of 7 m to the north of the central benchmark, along the edges of limestone anthropomorphic stelae protruding through the ground. The lower faces of both stelae were not processed. The eastern stela, 0.9 × 0.6 m and 20–25 cm thick, had stocky proportions. Half of the slab was outlined by a semicircular protrusion indicating the head (partially knocked down). The western stelae was oblong (1.2 × 0.8 m, thickness 0.12–0.2 m). The head on the anthropomorphic silhouette of this slab was displaced from the middle axial line. The burial chamber was found under the stelae at a depth of 0.8 m from the central benchmark. It was a rectangular pit with rounded corners, 1.4 x 0.75 m, filled with dense loam. The buried (adolescent) was lying on the back, with his head to the west. The arms were slightly bent at the elbows and laid along the body, palms down. The legs were bent and placed with the knees up; long bones are partially preserved *in situ*. The skull was covered with lilac-colored ochre, the hands – with a scarlet-colored ochre. To the right of the skull was a layer of chalk and a lilac ochre pellet in the shape of an egg, about 3 cm long. To the left of the skull was a piece of chalk or lime. The area under the skull was sprinkled with grains of chalk. The bottom of the pit was smeared with light clay.

The specimen failed amplification at Reich's Lab. Low-resolution mtDNA typing was conducted in the Molecular Archaeology Lab at GVSU, following the protocol detailed in[28,35].

I7929

REV-3.19, Revova Kurgan 3, Burial 19, male, 3711-3639 calBCE (4905±20 BP, PSUAMS-4763, this report)

Burial 19 was the main burial in the kurgan. The burial was found at a distance of 2.1 m to the east-northeast of the central benchmark, at a depth of 1.05 m. Scattered and mostly fragmentary human bones lay throughout the fill area in the eastern part of the pit. The layer-by-layer analysis of the remains suggests that the state and placement of the bone remains were consistent with the rite of secondary burial, perhaps with an imitation of the sitting position of the buried, possibly wrapped in cloth. The upper level of the bone "package" was occupied by the remains of the skull (parietal bones) and cervical vertebrae, the middle level consisted of the ribs, and the lumbar vertebrae and the sacrum lay in the lower tier. The bones of the left leg were completely missing, including the foot. The tibia of the right leg, also without a foot, was in a vertical position, while the femur lay horizontally with its head turned towards the main body of bones. The

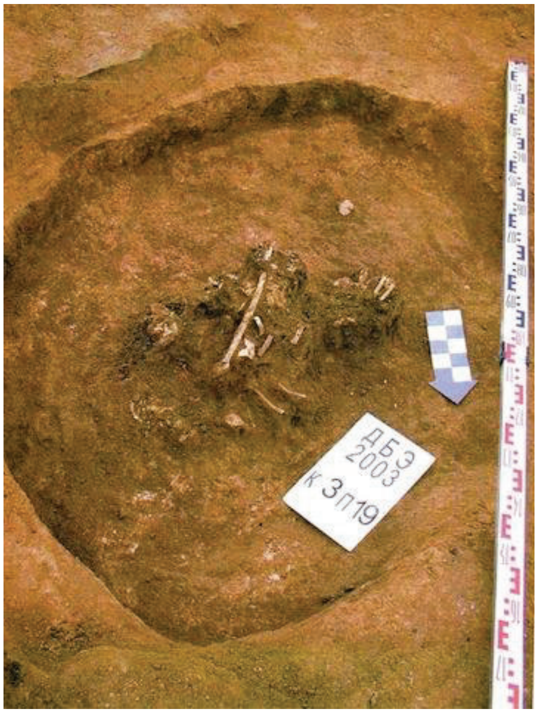

Figure SI1. 17. Revova Kurgan 3, Burial 19. Photo by S. Ivanova, 2003.

vertical position was occupied by the radius of the right hand. Other bones of the arms, including the hands, were absent. The zygomatic bones were located at the bottom of the pit. A patella rested somewhat away from the main assembly. Some fragments of the skull were painted with ochre. At the bottom of the burial chamber, near the bones, grains of scarlet ochre were noted. The dust from burnt timber planks extended throughout the chamber floor.

Shakhtar (Shevchenko) kurgan group

Shakhtar (Shevchenko) Hamlet, Dnipropetrovsk Region Nikopol' District (47.739, 34.179)

Kurgans 28 and 29 of the Shakhtar (Shevchenko) kurgan group, studied by the expedition of the Institute of Archeology of the Academy of Sciences of Ukraine under the leadership of S.V. Polin in 2003, were situated on the first above-floodplain terrace of the east bank of the Solena River, 200 m from the shore edge, 500 m east of the hamlet. The kurgans were 2.8 and 4.5 m high, respectively, located at a distance of 50 m from each other. In terms of size and relative position, these mounds represent a characteristic type of kurgans called "twins".

At the time of excavations, the upper half of Kurgan 28 was covered with turf, and the lower part of the slopes was plowed. The embankment had an asymmetrical shape: the northern slope was noticeably steeper than the southern one, which was greatly stretched and deformed by later diggings. The floors of the embankment along the circumference within a radius of 10–12 m from the top of the kurgan were cut by long-term plowing of the field. The height of the kurgan from the level of the field surface from the north was 2.8 m, and 2.5 m from the south. The apparent diameter of the embankment was 42 m. The kurgan contained 17 burials of the Yamna and Catacomb archaeological complexes as well inlet burials dated to later chronological periods.

I3141

Shakhtar (Shevchenko) Kurgan 28, Burial 12, female (35-45), 3300-2700 BCE[8,104]

Burial 12 (Yamna archaeological complex) was situated 12.8 m south-southwest of the reference point, in a rectangular pit with a wide ledge along the northwest-southeast axis, 1.2 m deep from the mound's surface. The ledge displayed white decay with plant fiber imprints (reeds?) across the pit. The burial chamber's bottom, 1.25 m below the ledge, sloped into the mainland by 0.95 m, narrowing slightly. Upper dimensions: length 1.4 m; center width 0.83 m, SE wall 0.75 m, NW wall 0.9 m. Bottom dimensions: length 13.8 cm; center width 0.78 m, SE wall 0.73 m, NW wall 0.7 m. Filling: loose mixed soil with reed floor decay. Matrix walls exhibited vertical grooves from digging tools, up to 2 cm wide.

The interred was a female, contracted on her back, with her head to the southeast. Her legs, knees bent upward, tilted to the right, her knees rested against the northeastern wall of the chamber. The arms were extended and slightly away from the body. From the cervical vertebrae along the right humerus stretched a low necklace of bone piercings, ending at the elbow with a hammer-head pin lying perpendicular to the arm, with the head on the elbow joint. In the middle of the ulna bones, on the inside of the right arm, opposite the pelvis of the interred, there was the wing of a pelvis of a fetal child. To the left of it, several more very small bones of a skeleton of an infant were located. At the north-eastern wall, under the knees, there was a cracked mass made of a dense but fragile substance of violet color (possibly ochre), about 10 cm in diameter and 4 cm thick. At the left foot there were two hooves (possibly from a sheep-goat), next to them was one small animal bone. At the bottom there were traces of a white plant decay covered by a

layer of red decay 0.5–1.0 cm thick (possibly from felt), covered by brown decay of a similar thickness in the pelvic area.

The necklace comprised eight tubular bone piercings with screw threads and two smooth beads from bird or small animal bones. Thread dimensions: length 2.7–5.7 cm, width 1.1–1.3 cm. Bead dimensions: 1.1 x 1.4 cm, thickness 0.8 cm; 1 x 1 cm, thickness 0.4 cm. The hammer-head pin had a cylindrical shaft, T-shaped head with a hole, shields at ends, length 21.8 cm, base diameter 1.1 cm, head length 3.9 cm, shield diameter 2.1 cm, hole diameter 0.5 cm.

Soldats'ka Slava Kurgan

Baranove, Odesa Region, Ivanivskyi District (46.938, 30.407)

Archaeological excavations near the village of Baranove were conducted in 1990-1991 by the expeditions of the Odesa Protective Archaeological Center under the Ukrainian Society for the Protection of Historical and Cultural Monuments. The "Soldats'ka Slava" kurgan was located on the lands of the Baranove village council of the Ivanovsky district. The kurgan was erected on the watershed between the Maly Kuyalnik River and the Krivaya arroyo, which flows from the left into the valley of the Svinaya River, a tributary of the Khadzhibey Estuary. The top of the watershed is a flat steppe plateau, stretched from north to south and southeast and marked in these directions by tall kurgans. According to measurements before the excavations, the height of the "Soldats'ka Slava" kurgan was 2.55 m. The diameter of the base of the embankment was 53–55 m. In total, 8 burials of the Bronze Age were found in the kurgan, including three of the Yamna archaeological complex (Burials 3, 4, 10), one each of the Kemi-Oba type (Burial 9), and the Babyne archaeological complex (Burial 2), two presumably Catacomb archaeological complex (Burials 1, 11), and Burial 5 without a clearly defined cultural affiliation.

I13072

SL-10, Soldats'ka Slava Kurgan, Burial 10, male, 2800-2000 BCE

Burial 1 (Catacomb archaeological complex) was discovered 9.5 m northwest of the central benchmark, at a depth of 2.4 m. The contours of the pit were not traced. The deceased was laid on the left side, with the head oriented northeast. The right leg was extended and placed over the left leg, which was bent at the knee. The arms were bent at the elbows, with the hands positioned in front of the face.

I12005

SL-10, Soldats'ka Slava Kurgan, Burial 10, male, 3265-2883 calBCE (4350±50 BP, Ki-17903[29])

Burial 10, of the Yamna archaeological complex, was arranged in a 2.25 × 1.40 m rectangular burial chamber. The chamber had a depth of 1.3 m from the ledge (5.65 m) and was covered by bark at its level. In the eastern part of the chamber, in the filling, at a depth of 0.8 m, a 0.8 × 0.5 × 0.15 m limestone cinder was found. The interred was laid contracted on the back, head to the west-south-west, legs bent at the knees and falling to the left, the arms were extended along the body. A thoroughly polished stone axe with no traces of a hilt was placed at the left shoulder. A pair of silver pendants rested at the temples.

Sychavka Kurgan

Sychavka, Odesa Region, Lymanskyi District (46.643, 31.101)

The expedition of the Department of Archeology of the North-Western Black Sea Coast of the Institute of Archeology of the National Academy of Sciences of Ukraine conducted a study of a kurgan near the village Sychavka (former Malaya Aleksandrovka) in 2010.

Three mounds and a cromlech were found in the kurgan. The construction of the kurgan began in the Eneolithic era. Most likely, the anthropomorphic stelae that were used in Burial 15 of the Yamna archaeological complex came from the cromlech. The kurgan was used in the Bronze Age by the tribes of the Yamna archaeological complex (Burials 9, 10, 13, 15, 19), the Catacomb archaeological complex (Burial 12), the Babyne archaeological complex (Burials 17, 18, 21, 23), and the Sabatynivka archaeological group (Burial 6).

I12843

S15, Sychavka Kurgan, Burial 15, male (40-45), 2857-2502 calBCE (4097±24 BP, weighted mean[10] of 3960±80 BP, Ki-16610[30] and 4110±25 BP, PSUAMS-9556, this report)

Burial 15 (Yamna archaeological complex) was discovered 5.2 m to the southeast of the central benchmark at a depth of 0.68 m behind a stone embankment. The part of the embarkment consisted of a large limestone slab, oriented along the west-east axis. The 1.48 × 1.78 × 0.95 × 0.28 m slab was sub-rectangular in shape. The lower side of the slab is slightly wider than the upper side. It was surrounded by masonry on three sides. Human rib fragments as well as a fragment of a stucco vessel were found under the main slab.

The skeleton of a 45-50-year-old male was lying in a very contracted position on his left side with his head tilted to the west. The left arm was extended to the knees, the right arm was bent at the elbow and the wrist was probably placed by the face (only the epiphyses of the ulnar and radial bones have been preserved). A stucco vessel was placed by the head. A stone grinder was located near the elbow. Ochre was not noticed. Under the pelvis, the floor was coated with green clay.

I12231

Sychavka Kurgan, Burial 18, male (18-20), 2118-1565 calBCE (3490±90 BP, Ki-16611[30])

Burial 18 (Babyne archaeological complex) was discovered 4.05 m south of the reference benchmark at a depth of 0.8 m beneath the covering stones. The burial was carried out in an undercut with a narrow slit-like entrance and a ramp. The 1.6 × 0.7m entrance pit was oriented along the west-east line. The entrance to the chamber was closed by two rows of three anthropomorphic limestone stelae of varying preservation. The gaps between the stones were filled with a clay-earth mixture. At a depth of 1.65 m, the ramp dropped vertically by 0.30 m to the bottom of the undercut. At the bottom, it had a bean-like contour measuring 1.6 × 0.6–0.7 m, with a height of 0.7–0.8 m.

The interred lay in a flexed position on the left side, head facing east. The arms were bent at the elbows with the hands raised to the face. The phalanges of the right hand were not preserved, and the left hand was clenched into a fist. The knees were bent at an acute angle to the body. Near the knees was a vessel lying with its mouth towards the southwest, and above it, opposite the arm bones, on light-gray decay, was a stone, possibly a grinding tool. In the northeastern part of the grave, between the left shin and the wall, lay a bone buckle.

Vapnyarka Kurgan 4

Odesa District, Odesa Region (46.576, 30.889)

Kurgans near the village of Vapnyarka comprise a burial ground known since the middle of the 19th century. Kurgan 4 was excavated by a team from the Institute of Archaeology, National Academy of Sciences of Ukraine, in 2008. The kurgan contained 20 interments. Two belonged to the Eneolithic period, 15 were from the Bronze Age, one was Scythian, and two others were from an undetermined period[162].

I12617

V4.3, Vapnyarka Kurgan 4 burial 3, female (12-14), 4089±24 BP (weighted mean[10] of 3960±70 BP, Ki-15230[22], and 4105±25 BP, PSUAMS-10808, this report)

Burial 3 belonged to the Catacomb archaeological complex. The skeleton was laid on the back in a slightly bent position, knees bent to the left, head facing southwest. Arms were slightly bent at the elbows, lying along the torso. A cluster of red ochre 3 × 4 cm was found by the right shoulder. A Catacomb pot was placed behind the head.

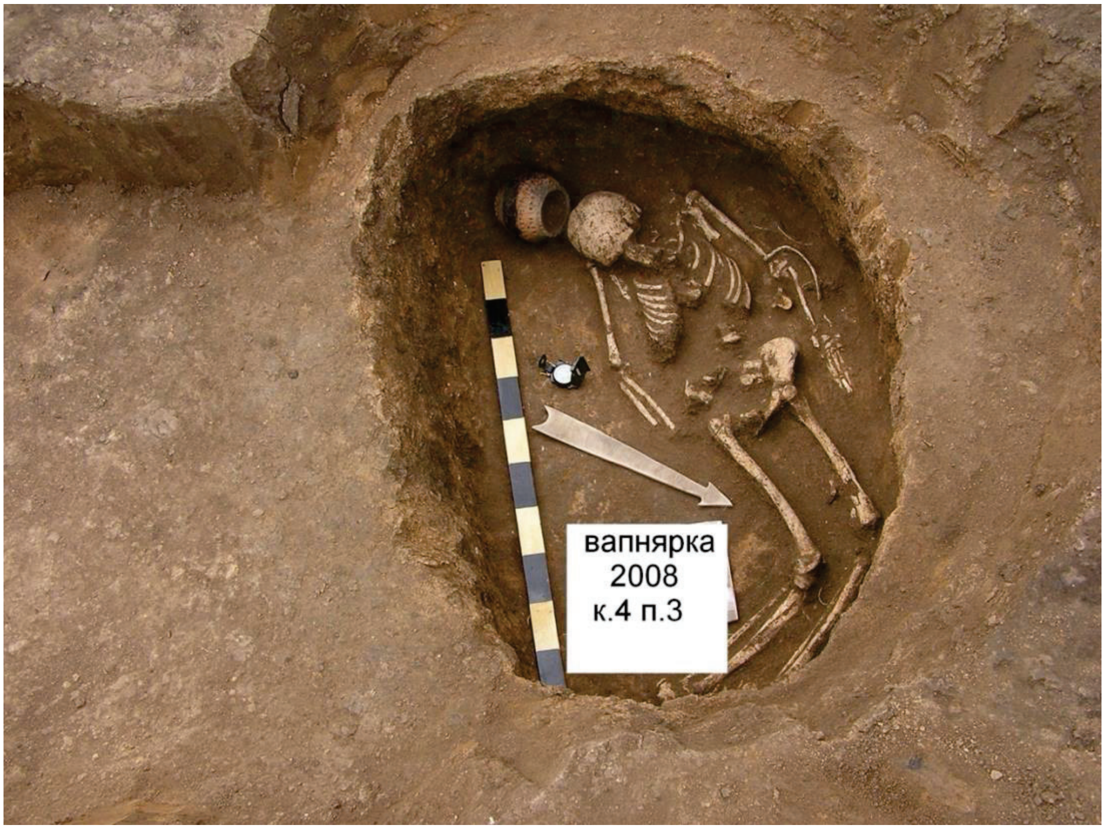

Figure SI1. 18. Vapnyarka Kurgan 4 Burial 3. Photo by S. Ivanova, 2008.

I12618

V4.4, Vapnyarka Kurgan 4 burial 4, female, 3341-3031 calBCE (4480±30 BP, BETA-647661, this report)

Burial 4 (main burial, Kvityana/post-Mariupol archaeological group) was discovered at 2.8 m north of the central benchmark at a depth of 0.45 m. The pit was covered transversely by three large polygonal, torn layer by layer, rough-hewn limestone slabs that split and sank into the grave in ancient times (some of the slabs were almost vertical). The length of the arrangement was 1.6 m, and the width was from 0.5 m (in the west) to 1.0 m (in the east). The dimensions of

the slab fragments: 0.80 × 0.65 × 0.15 m, 0.62 × 0.46 × 0.21 m, 0.38 × 0.38 × 0.18 m. The grave was sub-trapezoidal in plan with rounded corners ("bean-shaped"), tapering to the feet of the buried. It was 1.95 m long (2.0 m along the bottom), 0.95 m wide at the head, 0.70 m at the feet, 0.75 m in the middle part, 0.32 m deep from overlap (1.07 m from the central benchmark). It was filled with mixed soil (chernozem interspersed with fine limestone), identical to the structure of the first embankment. The edges of the pit were slightly beveled. The eastern edge was damaged during the deposition of Burial 9.

The skeleton lay straight on the back with the head to the east. The radius of the left arm rested between the femurs. The right part of the chest was damaged by the stones of the floor. The phalanges of the toes were partially preserved. The skeleton, particularly its frontal part, was weakly colored with bright red ochre. Ochre concertation was near the ulnar epiphyses on the humeral bones and on the upper part of the femoral bones. The heel bones were colored more intensely. At the bottom, near the chest, there was an ochre spot measuring 2 × 2 cm. The burial contained no inventory.

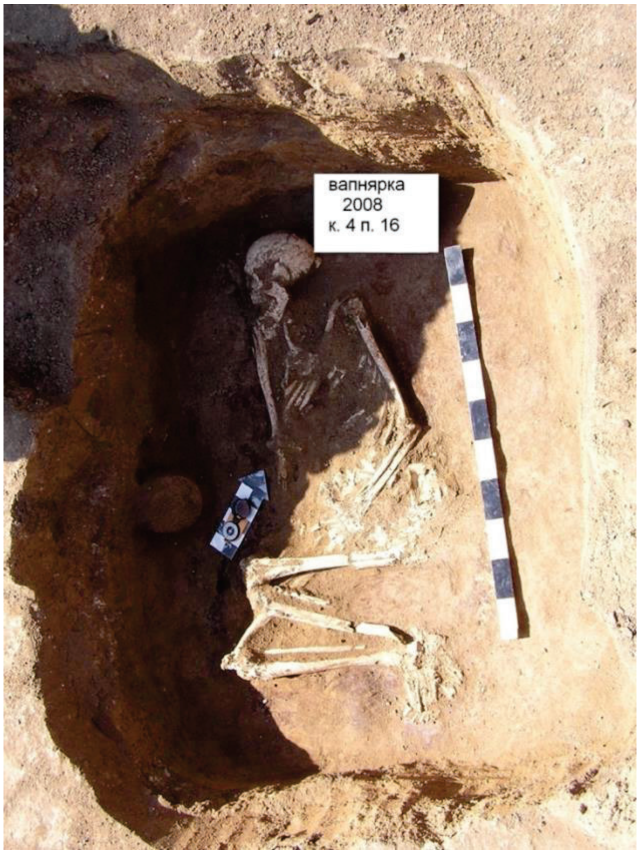

Figure SI1. 19. Vapnyarka Kurgan 4 Burial 16. Photo by S. Ivanova, 2008.

I12619

V4.16, Vapnyarka Kurgan 4, Burial 16, male (50-55), 2868-2462 calBCE (4050±60 BP, Ki-15014[22])

Burial 16 (Yamna archaeological complex) was located at 4.6 m to the west of the central benchmark at a depth of 0.93 m (stone floor level). The grave was covered transversely with three large polygonal limestone slabs torn in layers and slightly hewn. One of them, measuring 1.2 × 0.8 m, covered the pit in the headland and lay almost horizontally, partly on shoulders or a ledge. The second slab, 0.7 × 0.7 m, was located in the center of the grave and laid almost on top of the skeleton, destroying it. The third slab, 0.6 × 0.6 m, laid tilted at the eastern end of the pit. The slabs were partially coated with green clay. The grave was trapezoidal in plan with dimensions 1.45-1.50 × 1.00 (at the head) — 1.30 m (at the feet), 0.55 m deep from the floor. The northern corner was beveled to the bottom. In the north-western corner, a step (or part of the steps) measuring 0.8 × 0.5 m and 0.2-0.3 m deep was found, on which the floor slab was laid. The pit was filled with re-deposited yellow continental loam. At the bottom, brown rot from an organic mat remained.

The massive skeleton was laid contracted on the right side with the head to the northwest. The right arm was straight, the left was bent at the elbow, and the hands were near the knees. The legs were bent at a right angle to the body. A fragment of a goat's horn was found near the skull. A small fragment of a bronze artefact (possibly a string from an ankle bracelet, as there were

traces of green oxide on the large tibia near the ankle), was located. A truncated-conical jar-shaped vessel with two horizontal handles-stops crossed by through vertical holes, containing a small subtriangular stone and a lump of green clay, was located near the left ankle, under the western wall, opposite the right elbow. The vessel, 15.5 × 16.5 × 8.4 cm, was decorated with a cord ornament consisting of three horizontal lines under the crowns and a vertical "herringbone" on the body. The surface was pink, with gray spots, partially peeled off.

Verteba Cave
Bilche Zolote, Chortkiv District, Ternopil Region (48.7889, 25.8715)

The Verteba Cave archaeological site is described in [6,7,9,163]

DNA data on I1926, 1V1a-H1, I2110, 4.V4a-H4, I2111, 5.V5a-H5, I3151, 6 V6a-H6 originally published in[8,9].

I7584
VCS1.CL.05, Excavation Site 1, Individual 1, calcaneus (left side), female (subadult, >6), 3708-3543 calBCE (4875±25 BP, PSUAMS-10769, this report)

I7923
RL2.05, Excavation Site 2, Individual 1, radius (left side), male (adult), 3789-3649 calBCE (4955±30 BP, PSUAMS-8692, this report)

I7586
VCS1.TBL.05, Excavation Site 5, Individual 2, cuboid (left side), female (adult), 3766-3642 calBCE (4920±25 BP, PSUAMS-9527, this report)

I1929
9 V9a-A22.2, Excavation Site 6, Individual 2, female, 4910±400 BP (Ki-14308[6]), 3900-3500 calBCE. Archaeological context was used for calibrated date due to a large margin of error in the existing ^{14}C date.

I7921
A22.P3, Excavation Site 6, Individual 3, female (subadult, >6), third phalanx, 3900-3500 BCE

I13064
VRT-VNWL3, Excavation Site 7, left third metacarpal/ VRT-VSE.22N, right third metacarpal, Individual 2, male (adult), 3648-3528 calBCE (4820±30 BP, PSUAMS-7807, this report)

I2109
3 V3a-H3, Excavation Site 7, Individual 3.18.1, male (18-22), cranial bone, 3709–3537 calBCE (4863±33 BP, OxA-26202[7]

Additional analyses undertaken on the material from the Excavation Site 7 revealed that the following three specimens came from the same individual:

I1926, 1V1a-H1, male (35-45), cranial fragment, 3769-3648 calBCE (4938±22 BP, weighted mean[10] of 4890±30 BP, Beta-432808[8], and 4985±30 BP, OxA-25991[7–9])
I7922, TV2.6.1, individual 1, thoracic vertebrae
I13066, VRT-VTV3, thoracic vertebrae

Vynohradne Kurgan 3
Tokmak District, Zaporizzya Region (47.221, 35.57)

The kurgan group 0.5 km to the west of the village of Vynohradne was located on a high promontory of the right bank of the Molochna River. Kurgan 3 was located at the highest point of the promontory, rising 80 m above the floodplain. The kurgan was excavated by S. Pustovalov and Y. Rassamakin in 1982.

The height of Kurgan 3 was 8.05 m, the diameter was 100 m. The mound had the shape of a truncated cone with a wide and relatively flat square on the top. Under the mound, a unique sanctuary of complex construction was discovered. Under the general embankment, 49 burials of the Eneolithic, Yamna, Catacomb, Babyne and Zrubna cultures were discovered[131].

The embankment of Kurgan 3 had a complex structure. Under the sod surface of the kurgan there were eight separate mounds, which were later united by a multilayered common embankment. A sanctuary was erected on top of the eighth embarkment.

Several stages can be traced in the functioning of the sanctuary. Initially, a 0.7 m × 1.5 m altar made of large clay blocks was erected in the center of the kurgan. The altar was surrounded from the south and east and possibly from the north by a path covered crosswise with reeds. Fragments of animal bones were found on the path. The path was later covered with clay and the altar was enlarged in the northern direction. As a result, a square approximately 20 m in diameter was formed with an altar in the center. Radial embankments were built on the slopes of the eighth embankment during the first stage of sanctuary functioning.

During the second stage, the space between the radial embarkments was filled with sod chernozem blocks, forming a continuous platform of approximately oval shape, 35-45 m in diameter, with an altar in the center. Two ramp entrances were attached to the platform, one from the east, the other from the west. Each of the ramps had two entrances, opposite each other, one from the north and one from the south.

In the last, third stage of the sanctuary's existence, all the ramps were filled in and the kurgan expanded to the north. Only one 10 m-wide entrance from the east remained.

According to aerial photography, 13 straight radial dark lines (roads) ranging in length from 1,200 m to 4,566 m - extend from the kurgan into the steppe to the north, northwest, west, and southwest. These radial lines are crossed by several identical lines running from north to south. The reconstructed number of ramparts coincides with the number of road lines that radially approach the kurgan from the side of the plateau[131].

I1430
VIN1, Kurgan 3 Burial 15, male, adult, 4241-3951 calBCE (5230±60 BP, Ki-14726[19])

The main burial 15 was in the central of the eight mounds of Kurgan 3, 1.1 m from the ancient horizon. The burial chamber was surrounded by a ditch. The adult male at the bottom of the burial chamber was buried contracted on his back ("half-sitting"), with his head to the east. The skull, with a trepanation on the crown in the form of an oval hole, rested against the end wall of the pit, with the mandible touching the upper ribs. Legs were bent with knees up and at the

same time spread apart in different directions (found *in situ* during the excavation). Arms were slightly bent at the elbows, somewhat apart. At the bottom of the chest laid a fragment of a large knife-like blade. The entire skeleton was painted with ochre[20].

The cultural affiliation of the Vynohradne Burial 3.15 is viewed as part of a continuation of Early Eneolithic steppe traditions of the Skeyla or Novodanylivka type[131,164,165].

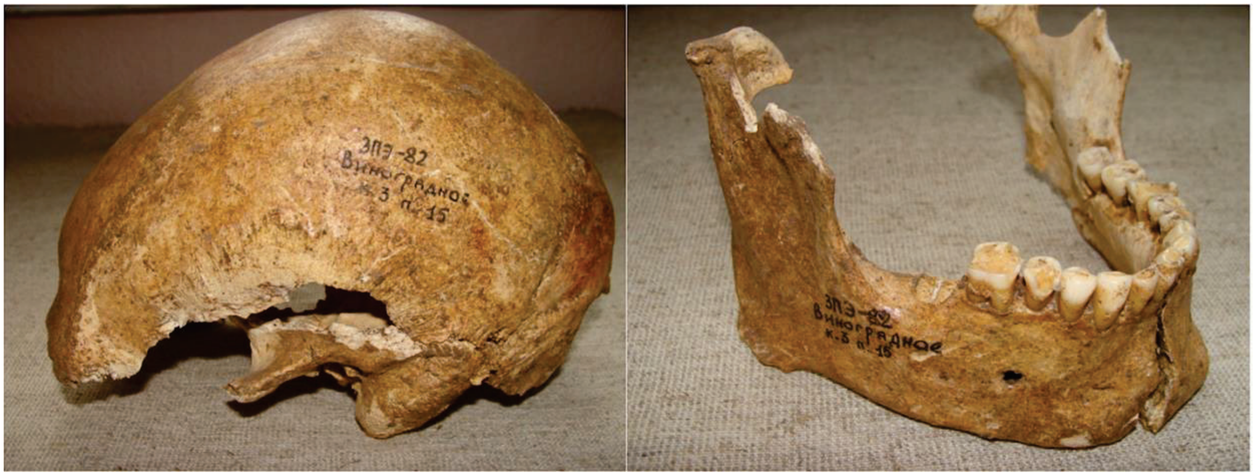

Figure SI1. 20. Vynogradne Kurgan 3 Burial 15. Photo by I. D. Potekhina, 2012.

Yasynuvatka
Vilnianskyi District, Zaporizhzhia Region (48.12, 35.09)

Yasynuvatka is one of the Mariupol-type cemeteries of extended burials in the Dnipro Valley. Yasynuvatka was discovered by A. B. Borodyansky and excavated by D. Ya. Telegin in 1978. The cemetery contained 68 burials. The earliest phase at the cemetery is represented by type A oval pits containing one to six skeletons. Multiple burials in the A-type pits were often on top of one another. Subsequent burials at the cemetery were placed in one large pit Б, 5.2 × 5.6m, the bottom of which was covered in red pigment.

V. N. Danilenko attributed Mariupol-type cemeteries to the Azov-Dnipro archaeological group. D. Y. Telegin considered these cemeteries to belong to several groups of the Dnipro-Donets archaeological complex (DDAC), extending from the Neolithic to the early Eneolithic. The latter included the late DDAC phase with Zasukha-type ceramics, synchronous with the early phase of Serednii Stih, represented by the Oleksandriya settlement and cemetery on the Oskil River [3].

I27982
Burial 23.1, Pit Б-1, single cranium, male (40-45), 5700-5100 BCE
The Bioarchaeological Stores of the Institute of Archaeology of the National Academy of Sciences of Ukraine in Kyiv have individuals labeled Burial 23 Skeleton 1 and Burial 23 Skeleton 2. However, D. Y. Telegin’s sketches of the burial pit only show a single cranium associated with Burial 23.

I27983

Burial 24, male (30-40), 5700-5100 BCE

A male (30-40) burial in the red fill of Pit Б, extended supine, head to the NE (Б-1 type burial). Arms were slightly bent at elbows, wrists on the pelvis.

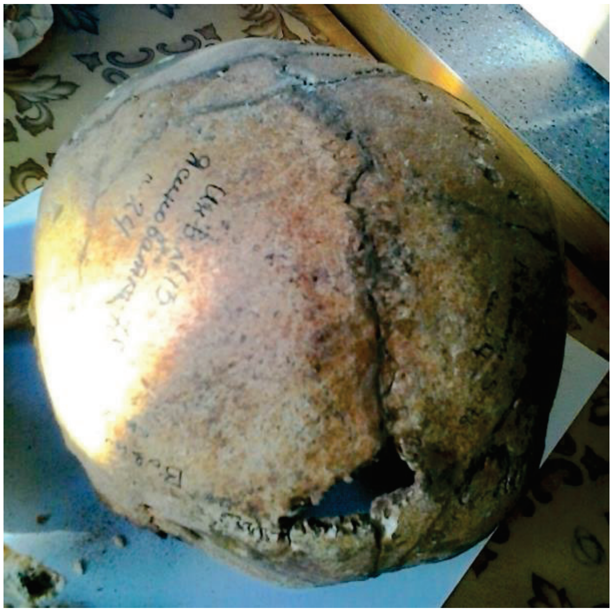

Figure SI1. 21. Yasynuvatka Burial 24, occipital view of the cranium. Photo: A. G. Nikitin, 2014.

I27990

Burial 44, Pit Б-1 type burial (single cranium) in Pit Б, male, 5700-5100 BCE

I27992

Burial 50, female, 5700-5100 BCE

An adult was buried in a boat-shaped grave under the north wall of the collective burial pit Б. The burial position is extended supine, head to NE, and face to the right. The tips of the feet and the cranium were 20-30 cm above the pelvis.

I27994

Burial 55, Pit A2, male (25-30), 5630-5484 calBCE (6650±30 BP, Beta-445802[5])

Burials 55-58, 58a, and 59 were deposited in an oval pit A2, under Burial 31.

3. Archaeogenetic notes and highlights

Written by Alexey G. Nikitin and Iosif Lazaridis

Here we present notes on selected individuals of particular archaeological interest in the light of modeling detailed in Supplementary Information, Section 2, as well as discussing uniparental lineage connections of individuals presented in the current report.

3.1. Genetic ancestry of Zhyovotylivka-Volchans'k/III-C burial type (ZV/III-C) individuals

Six individuals from Moldova are from burials that carry features of ZV/III-C burial type. Five of these, three from a quadruple burial at Bursuceni, one from a double burial at Taraclia II Kurgan 2 and an individual from Mereni kurgan 1 are contemporaneous and date to the chronological interval of 3350-3030 calBCE, which marks the beginning of the initial Yamna expansion.

Individuals I20078 from Taraclia and I17974 from Bursuceni can be modeled as Core Yamna + Steppe Maykop ancestry (Tables SI2 15 and SI2 16). The IBD analysis uncovered that individual I20078 is a second cousin of Steppe Maykop specimen SA6004 from Sharakhalsun Kurgan 2 burial 18[166]. The two individuals have four IBD blocks longer than 20 cM, one of which is 45.9 cM[49]. The I20078- SA6004 kinship provides genetic support for a link between the communities of the ZV/III-C burial type from the North Pontic and those from the Kumo-Manych periphery of the Maykop-Novosvobodnaya domain[64].

Individual I17974 (one of the identical twins from Bursuceni) has much lower amount of Steppe Maykop-related ancestry than the I20078 individual. A parsimonious interpretation of the modeling presented in SI 2 suggests that this individual, like I20078, was formed of the same Yamna+Steppe Maykop admixture process but with about ~1/3 of the Steppe Maykop ancestry found in I20078.

Individual 17973 (the male adolescent from Bursuceni) has ancestry from the south Caucasus (Table SI2 20). Modeling suggests that over 90% of ancestry of this individual comes from an Aknashen-derived source with an admixture of steppe ancestry, similar to that of the Maykop population, but with a higher proportion of the south Caucasus ancestry compared to that of Maykop.

Individual I17743 from Mereni is of Core Yamna ancestry with ca. 7% European farmer admixture and is the earliest directly dated individual in the NPR of the Yamna ancestry containing such admixture. Individual I20079 from Taraclia II Kurgan 10 dated to the middle 3rd millennium BCE is a Yamna descendant (Extended Data Table 4).

3.2. Corded Ware connections

Within our sample selection, Yamna individual I12005 from northwest Pontic (Soldats'ka Slava Burial 10) and an individual from a catacomb burial with an ambiguous cultural placement from the Podillya region in west Ukraine (Bil'shivtsi Burial 1, I13071, 2201-2032 calBCE) demonstrate potential connections to Corded Ware. Their mtDNA lineages belong to distinct sublineages of the T2c1 clade, likely of steppe origin. On the PCA (Figure 2a), they cluster together, with Individual I27983 (Yasynuvatka burial 24) positioned in close proximity. In PCA iterations involving central European Bronze Age specimens, this location is populated by

central European Únětice and Corded Ware. While I27983's contamination raises concerns about its use in comparative analysis, its proximity to the central European Bronze Age is noteworthy. Individual I13071 fits multiple models, most of which suggest a significant North Caucasus genetic ancestry. When modeled with Core Yamna and EEF ancestry, approximately a third of I13071's ancestry appears to originate from EEF (Table SI2 4). Finally, Individual I1456 from Durankulak in Bulgaria contains genetic ancestry components characteristic to Corded Ware (pp. 97-98).

3.3. Uniparental lineage connections in the Ponto-Caspian region

3.3.1 MtDNA lineages

Our study uncovered a considerable amount of mitochondrial lineage diversity in most populations studied, with some lineage sharing among the groups.

Of the nine Neolithic samples in our selection, six carried derivative lineages of U4 and U5 clades. The individual from burial 55 at Yasynuvatka carried haplogroup T2ab1, also found in a Serednii Stih individual from Burial 5 at Deriivka 2. While the origin of the T2 clade is considered to be in the Neolithic Anatolia and the Fertile Crescent where its earliest carriers have been reported[167,168], there is a lack of T2ab1 carrying specimens among prehistoric individuals until the second half of the 3rd millennium BCE, when this lineage is found in the steppe-derived Corded Ware context[169]. Lineage K1a3 of the individual from Yasynyvatka burial 50 is Anatolian derived[168], and clade H identified in the Yasynyvatka individual from burial 24 and derivatives of H have a broad distribution from the European Mesolithic and Neolithic to the Neolithic Levant and Anatolia[170–172]. Two other individuals from the Yasynyvatka cemetery carrying the H clade have been previously reported[35].

Trypillian lineage diversity in our sample group is limited to the Anatolian Neolithic farmer (ANF)/EEF-derived haplogroups and includes members of previously reported mitochondrial lineages in Trypillia. The finding of additional lineages such as K1b2, H2a and H5b not previously identified in Trypillia adds to the substantial lineage diversity in Trypillia reported to date, suggesting a large population size of the founding population, as well as reflecting the overall size of Anatolian farmer-derived mating network and the absence of population bottlenecks [173].

Usatove mitochondrial haplogroup composition consisted of lineages of predominantly ANF/EEF origin (H*, K1b*, T2*, W1, X2d), as well as steppe and European hunter-gatherer-derived U4* and U5a1* lineages. The Mesolithic distribution of U5a1 lineages is primarily confined to the Iron Gates area, as well as Norway and the Baltic region [8,174]. In the Neolithic, U5a1 lineage distribution extends to the North Pontic, likely accompanying the migration of carriers of WHG ancestry to the Dnipro Valley [8]. Carriers of U5a1* in our study were identified in the Neolithic Mariupol-type cemeteries at Mariupol (individual I31730) and Yasynuvatka (individual I27982). Individuals I17974 from Bursuceni and a Catacomb individual from Burial 11 at Dubynove Kurgan 1 carried U5a1-derived haplogroup U5a1g1. Subgroup U5a1g was also reported in a CWC individual from Germany[168], roughly contemporaneous with the Catacomb individual from Dubynove. A Mayaky individual I12707 carried U5a1d1 haplogroup, which was also identified in individual RISE240[175] from Sukhaya Termista in the northwest Manych region of the Ponto-Caspian steppe (the Remontnoye area).

Mitochondrial DNA of individuals I2108 from Ogrin-8 (Serednii Stih) and I12637 from Glinoe (Yamna) belonged to the U2e* lineage. Haplogroup U2 has been present in the Don River Valley in the northeastern NPR since the Upper Paleolithic[171,176]. Carriers of U2e* have been identified in the Mesolithic Baltic, Scandinavia and Neolithic Ukraine[8,169,177].

The U4-derived lineages found in our study are shared with prehistoric groups regionally and throughout Europe. Phylogeographic distribution of the U4 node and its derivatives in pre-Eneolithic Europe includes the Iron Gates area of the Danube, the Baltic region, and the Middle Dnipro Valley [8,174,178]. Chronologically earliest representatives of the U4 haplogroup in Europe date to the Epipaleolithic and come from the Dnipro Valley [5,179].

The chronologically earliest individual I12615 from the Mayaky archaeological site shared mtDNA lineage U4b1b2 with the individual of Serednii Stih ancestry (SSmed) from Kolomiytsiv Yar Tract (KYT)[16]. It has been suggested that the U4b1b1~ lineage represents the pre-Neolithic mitochondrial lineage diversity, expanding from the Mesolithic Balkans and not being directly influenced by the steppe[178], at least in the initial stages of the lineage expansion. Both the Mayaky and the Serednii Stih sites of the Dnipro Valley are located along the route of the Eneolithic circum-Pontic trade network connecting the Balkans and the Eastern Steppe[16,54]. The presence of a Balkan-derived mitochondrial lineage in an individual of steppe ancestry supports the existence of mating interactions between the steppe and their trade allies from the eastern Balkans.

Whole-genome amplification of DNA from individual K1.10 (specimens I12167/I31733) from the Katarzhyno 1 kurgan, a potentially chronologically earliest kurgan in northwestern NPR, did not produce quality nuclear DNA data. The initial mitochondrial DNA analysis of K1.10 was reported in[28]. Subsequent sequencing of additional PCR amplicons of the mtDNA coding region from two additional extractions of K1.10 produced a recurring transition at nucleotide position 12308, suggesting the placement of the specimen in the U clade. Considering the previously reported lack of diagnostic polymorphic sites in the control region of mtDNA, no further refinement of the haplogroup designation for K1.10 can be made.

Two individuals of Yamna ancestry in the Katarzhyno 1 kurgan shared their mtDNA (U4a/U4a2) as well as Y chromosomal lineages. No IBD segments have been identified between the two, probably because of the large time gap between them. The uniparental lineage match between two individuals in the same kurgan raises the possibility of a familial relationship among the interred in EBA kurgans, especially considering that the mtDNA lineage of the main burial in the Katarzhyno 1 kurgan belongs to the U clade as well. However, not enough data is available to test this relationship further.

Mitochondrial DNA lineage U4c1 of the main Yamna burial of the Katarzhyno 2 kurgan has been identified in a Yamna burial from the Prydnystryanske kurgan 4 of the Yampil kurgan sequence from the middle Dniester area, as well as in one of the two early Usatove individuals from burial 6 of Kurgan 8[5,180]. This lineage sharing adds support to the involvement of the Eneolithic communities in the formation of the Yamna groups of the greater northwest Pontic area[181]. On the other hand, mitochondrial DNA lineage sharing between a Trypillian individual from Cunicea and individual I20079 from Taraclia carrying Yamna genetic ancestry provides support for maternal genetic lineage continuum between Trypillia and Yamna in Moldova.

Mitochondrial lineages of burials I13071 (Bil'shivtsi Burial 1, northwestern forest-steppe) I12005 and (Soldats'ka Slava Burial 10, northwest Pontic steppe) belong to the T2c1 haplogroup. Subdivisions of this haplogroup are found throughout Europe in the Neolithic-Bronze Age. The T2c1a2 sub-lineage carried by the Yamna I12005 individual has also been

identified in a Yamna individual I0429 from Samara in Russia[168], suggesting maternal lineage ancestry sharing among the Yamna individuals across the Pontic-Caspian steppe in the EBA.

MtDNA lineage H13 of the Giurgiulești individual I20072 and the VIN 1 Serednii Stih individual I1430 has also been reported in the Mesolithic Iron Gate individuals from Lepenski Vir and Ostrovul Corbului[8], suggesting potential genetic admixture between CLV Wave 1 migrants such as the individuals from the Giurgiulești necropolis and local populations of the Lower Danube. This lineage could also have arrived at the Dnipro Valley as part of the WHG Iron Gates ancestry in the Neolithic[8], thus becoming part of the Dnipro Cline ancestry from which the Serednii Stih ancestry of VIN 1 was 1/5th derived. An H13-derived lineage H13a1 was recorded in a Lower Volga individual I22199 from Berezhnovka II[49], as well as in a Yamna individual from Ishkinovka in pre-Ural steppe[169]. Genetic ancestries of both I22199 and I20072 are derived from Lower Volga populations of the BP group[49]. An H13a1-derived lineage H13a1a2 was observed in a Babyne individual I12235.

Two contemporaneous Yamna individuals from Moldova from Sărăteni and Cotiujeni shared an H13-derived lineage H13a2b2a. Outside of the Pontic steppe, this lineage was found in Bronze Age Germany[182].

Sublineages of the H6a1* haplogroup of the Eneolithic early Yamna individual from Mykhailivka and an EBA Yamna individual from Sărăteni have widespread distribution in Bronze Age Europe. The earliest occurence of H6a1* outside of the NPR is in an EBA Yamna individual from the Kutuluk River near Samara in Russia[169]. The distribution of the H6a1* lineages in Europe and Central Asia suggests their spread is linked to Yamna expansion.

3.3.2. Y lineages

Our study shows a diverse array of Y lineages of our sample selection of the North Pontic area, represented by autochthonous European and Anatolia-derived lineages, as well as lineages that are found in prehistoric populations of North Caucasus.

Y chromosomal lineages identified in Trypillian specimens in the present study include lineages of I2a1b, G2a, E1b1b, and J2a1. Considering the Y chromosome lineage diversity reported in previous studies of Trypillians from Verteba Cave, the Y chromosome lineage diversity in Trypillia appears to be quite substantial and on par with the mtDNA lineage diversity.

Two Y chromosomal lineages, G2a and J2a, found in Trypillian specimens from Verteba Cave, are considered to have originated in Anatolia and are associated with the spread of agriculture out of Anatolia into Europe in the Neolithic [183]. The G2a2b2a lineage identified in two Trypillian specimens in this study has been found in Neolithic northwest Anatolia, Neolithic Austria, Bulgaria, and Hungary, as well as early Eneolithic Bulgaria (Ivanovo) and Romania (Curătești) [5,8,184]. The latter two sites are located in the Lower Danube, either within or bordering the southeastern part of the Romanian historical region of Muntenia, also home to the Eneolithic Bodrogkeresztúr culture, with which Trypillia have been previously shown to share genetic ancestry[8].

Individual I17973 of Caucasus genetic ancestry from Bursuceni carried Y haplogroup J2b2b2~ (J-Z42942). Subineage J2b2* (J-M241) shows up at low diversity in the Balkans during the Neolithic. Its origins remain uncertain[183], but it was present in two Neolithic individuals from the Mentesh Tepe site in Azerbaijan[185], making a Caucasus origin in the I17973 individual probable.

Three of Trypillian specimens in our study carried derivatives of the I2a1b Y haplogroup. Both Trypillian individuals from Moldova carried the I2a1b1a2a2a lineage and one specimen from Verteba Cave in Ukraine carried the I2a1b1a2b lineage. I2a1b is part of the Y lineage diversity of the European Mesolithic and Neolithic, but it is not found in post-Neolithic samples from Europe, except for the Orkney Islands of Scotland [186]. It is possible that the group became part of the European Neolithic ancestry as some of the European Mesolithic hunter-gatherers became integrated into the ANF-derived EEF communities [186,187].

Among the specimens in our selection, a Trypillian individual from Verteba Cave and a proto-Usatove individual from Mayaky carried E1b1b1a1b* Y chromosomal lineages. The proto-Usatove designation of the Mayaky individual is based on the ^{14}C date that predates Usatove even after adjusting for reservoir effect[5] (Section 1.2.1). The E1b1b1a1b lineage is not associated with the Balkan-Danuban route of farming expansion from Anatolia to central Europe in the Neolithic[183]. On the other hand, the E1b1b1a1b~ expansion in the Mediterranean, which is timed to the late Mesolithic in southeast Europe [183], raises a possibility that it coincided with the spread of agriculture out of the Fertile Crescent into southern Europe via the Mediterranean coast beginning ca. 9000 years ago [188]. Such as, Y chromosomal lineage diversity in the Epicardial culture of the Cardial Ware complex of the Neolithic Mediterranean shows Near Eastern influence and Cardial/Epicardial mtDNA lineage frequencies and diversity are comparable to those from Near Eastern Pre-Pottery Neolithic B sites[189]. A bearer of the E1b1b1a1b1 haplogroup belonging to the Cardial Ware archaeological complex was reported from Croatian Zemunica Cave in western Balkans[8]. Cardial Ware complex is considered to have influenced the Late Neolithic-Eneolithic Hamangia culture of the west-northwest Pontic [190]. Hamangia, in turn, is considered to have influenced the formation of Precucuteni-Trypillia A [191]. The presence of the E1b1b1a1b1 lineage in Trypillia and a proto-Usatove individual strengthens the possibility of a link between Hamangia and Trypillia as well as Trypillia and Usatove and potentially connects genetic ancestry of Trypillia and Usatove with Cardial Ware.

Lineages of the Y chromosome in two Neolithic individuals from Yasynyvatka and an Eneolithic individual from Taraclia belong to the Q1b clade. Q1b1 has a general Eurasian distribution. The Taraclia individual shared the Y chromosomal lineage with his second cousin, a steppe Maykop individual from Sharakhalsun (SA6004, Q1b2b1b2b~), suggesting the kinship relationship between the two to be paternally derived.

The Q1a2 lineage identified in an individual from Giurgiuleşti is rare among European Y chromosomal lineages. Its origins in Europe have been connected to Hunnic expansions[192]. In prehistoric individuals, this lineage was detected in the Bronze Age of south Siberia[193].

The J-Y6313-deived J-FT265222 lineage of Y haplogroup J1 identified in a genetically Usatove individual from the Revova Kurgan 3 (Burial 19) is present in modern populations of Europe, as well as Saudi Arabia and Iraq. Another Usatove male from Mayaky carried the R1a lineage, has a widespread Eurasian distribution, but its initial diversification is thought to have started in Iran[194].

4. Strontium isotope analysis of specimens from the Neolithic Mariupol Necropolis and settlement

Written by Alexey G. Nikitin and Virginie Renson

Two individuals from the sample selection reported in this study came from the Neolithic Mariupol Necropolis. One of the specimens, ANB002 (I31731) came from the Neolithic Burial 50 (double burial of an adult and a child (sampled)). Another specimen, ANB003 (I31732) came from Burial 21 (from one of the two skeletons in this tripe burial, dated to the Eneolithic period). These were excavated by M. Makarenko in 1930 prior to the construction of the Azovstal steel plant and subsequently transferred to the Museum of Local History of Mariupol[1], where they remained until the Russian invasion of Ukraine in 2022. The third sample, ANB005, came from the Neolithic layer (257 m^2 of total excavated area) of the Kalmius settlement in the vicinity of the necropolis[195]. The Kalmius settlement archaeological site was discovered in 2010. Excavations were carried out by the Archaeological Expedition of the Mariupol Museum of Local History under the direction of V.M. Gorbov in 2010-2012, 2014, and 2015. The settlement is considered to be associated with the necropolis.

The Museum of Local History of Mariupol and its collections including the excavated artifacts from the Neolithic Mariupol Necropolis, as well as the Kalmius settlement site, were destroyed by the Russian bombardment during the siege of Mariupol in the spring of 2022[151].

Samples of bone and teeth for the current analysis were collected in the fall of 2021. The ANB002 and ANB003 samples, to our knowledge, are the only human biological artefacts from the Mariupol Necropolis that survived the Russian invasion. In addition, these two samples have not been contaminated by heavy metals in the result of modern warfare (WWII, 1941-45, and the Russian invasion of 2022) as well as environmental pollution caused by the operation of the Azovstal steel plant.

The tooth enamel and bone samples were prepared and analyzed at the University of Missouri Research Reactor (MURR). The samples were mechanically cleaned using a microdrill equipped with a bristle brush and the bone samples further cleaned with a diamond bit. A small fraction of the samples was taken for analysis. In the case of the tooth, a small corner was cut using a diamond disk mounted on a Dremel. All materials were rinsed with mQ water. The bone samples were soaked in a solution of 2% NaOCl for eight hours and rinsed three times with mQ water, then soaked in 0.1N acetic acid for 30 minutes, rinsed five times with mQ water, soaked in 0.1N acetic acid for about seven hours, rinsed five times with mQ, and the last two steps were repeated once more. The leaching cycles for the enamel were shorter and as follows: one hour in NaOCl, then two hours and one hour in 0.1N acetic acid. The number of rinsing cycles was similar. All samples were then dried at room temperature. The remaining weights were 4.4 mg, 9.9 mg and 2.3 mg for ANB002, ANB003 and ANB005, respectively. The three samples were dissolved in 2ml of 7N HNO_3 in a PFA vial at 110˚C for 24 hours. The dissolution was followed by an evaporation cycle at 90˚C. The dry residues were re-digested in 7N HNO_3 prior to the Sr separation using a protocol adapted from[196]. The Sr eluates were evaporated at 90˚C, and the residue dissolved in 0.05N HNO_3 before conducting the strontium isotopic analysis following the procedure described in[16]. The average and standard deviation calculated for the SRM987 were 0.71022 ±0.00003 (2sd) (n=16).

The enamel sample (ANB002) has a $^{87}Sr/^{86}Sr$ ratio of 0.70950 and the bone samples have a $^{87}Sr/^{86}Sr$ ratio of 0.70990 and 0.70993, for ANB003 and ANB005 respectively (Table SI1.2).

The strontium isotopic signature of the Mariupol samples was compared with values from[197], which provide estimated ranges of $^{87}Sr/^{86}Sr$ ratios for the main geological ages in Ukraine. The site of Mariupol is located in a geological context that corresponds mainly to Miocene and Pliocene sediments and sedimentary rocks with a $^{87}Sr/^{86}Sr$ ratio estimated between 0.709 and 0.711[197] and a diversity of Precambrian metamorphic rocks with a $^{87}Sr/^{86}Sr$ ratio estimated between 0.712 and 0.780[197]. An overview of the lithology of Ukraine is presented in Fig. SI1. 22.

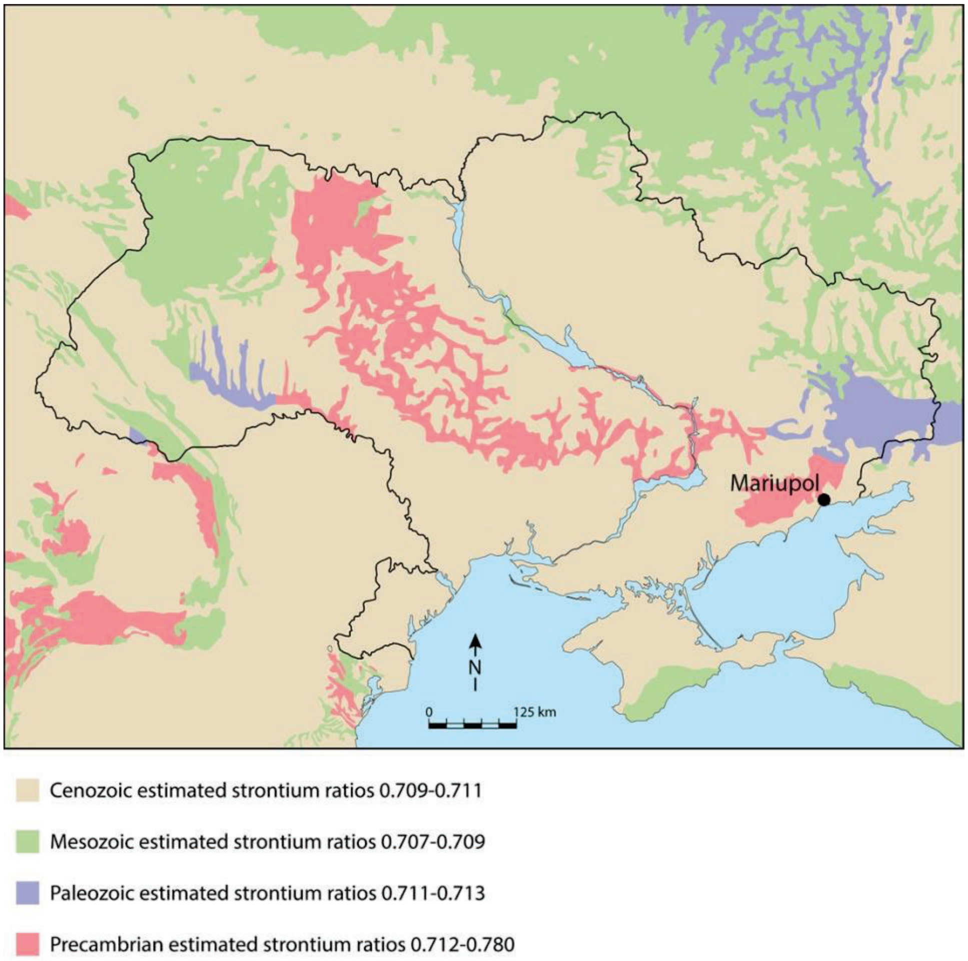

Figure SI1. 22. Simplified geological map of Ukraine.

Modified after[197], CC BY 4.0, to show the location of Mariupol.

All three samples have a signature that is compatible with the range of expected local $^{87}Sr/^{86}Sr$ ratio. These substrates are, however, largely distributed and the same range of $^{87}Sr/^{86}Sr$ ratio can be expected for a large portion of Ukraine. The $^{87}Sr/^{86}Sr$ ratio of seawater (0.7092) falls within the range estimated for Miocene and Pliocene sediments and sedimentary rocks.

The samples were compared in more detail with data available for local baselines and human remains for a number of sites in Ukraine and published in[7,197,198]. We use the local baseline ranges as defined in[197]. The results are presented in Fig. SI1. 23 and Table SI1. 2.

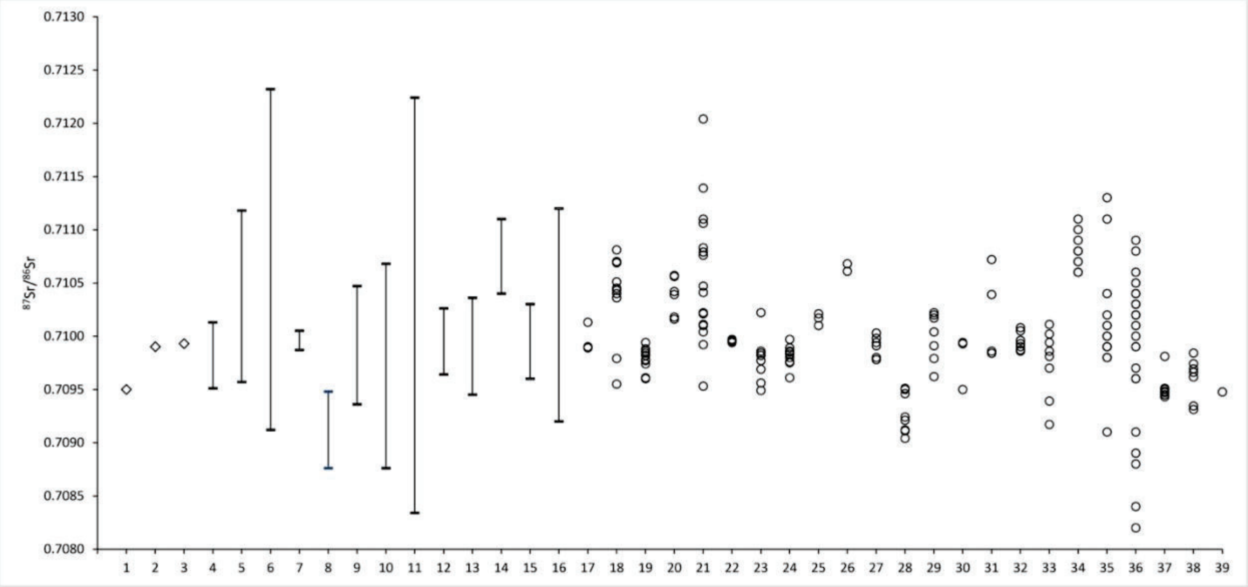

Figure SI1. 23. Comparison of the $^{87}Sr/^{86}Sr$ ratio of the three samples from Mariupol with ratios for baselines and human remains (including enamel, dentin, and bone samples) from other sites in Ukraine and Olenii (Russia).

The baseline ranges are reported as defined in[197]. The values for human remains are from[7,197,198] and are reported by site and by culture. Data used to construct the graph are presented in Table SI1.2. Samples from Mariupol: 1. ANB002, 2. ANB003, 3. ANB005. Baselines: 4. Vynohradne, 5. Pishtchanka, 6. Kirovohrad, 7. Shakhta Stepna, 8. Nevs'ke, 9. Alexandropol, 10. Babyna Mohyla, 11. Drana Kokhta, 12. Ordzhonikidze, 13. Zolota Balka, 14. Medvin, 15. Mamai-Gora, 16. Bel'sk. Human remains (including enamel, dentine and bone): 17. Vynohradne - Eneolithic, 18. Pishtchanka - Eneolithic, 19. Vynohradne - Yamna, 20. Pishtchanka - Yamna, 21. Kirovohrad - Yamna, 22. Shakhta Stepna - Yamna, 23. Vynohradne - Early Catacomb, 24. Vynohradne - Catacomb, 25. Pishtchanka - Catacomb, 26. Kirovohrad - Catacomb, 27. Shakhta Stepna - Catacomb, 28. Nevs'ke - Catacomb, 29. Alexandropol - Iron Age, 30. Babyna Mohyla - Iron Age, 31. Drana Kokhta - Iron Age, 32. Ordzhonikidze - Iron Age, 33. Zolota Balka - Iron Age, 34. Medvin - Iron Age, 35. Mamai-Gora - Iron Age, 36. Bel'sk - Iron Age, 37. Olenii - Eneolithic and/or Bronze Age, 38. Verteba Cave - Eneolithic, 39. Verteba Cave - Iron Age.

The $^{87}Sr/^{86}Sr$ ratio of sample ANB002, 0.70950 ±1 (2se), is within the local baseline ranges of the sites of Kirovograd, Babyna Mogyla, Drana Kokhta, Zolota Balka and Bel'sk, and close to that of Vynohradne and Nevs'ke. It is different from the local baseline ranges for the sites of Pishtchanka, Shakhta Stepna, Alexandropol, Ordzhonikidze, Medvin, and Mamai-Gora.

The $^{87}Sr/^{86}Sr$ ratios of samples ANB003 and ANB005, 0.70990 ±1 (2se) and 0.70993 ±1 (2se), are within the local baseline ranges of the sites of Vynohradne, Pishtchanka, Kirovograd, Shakhta Stepna, Alexandropol, Babyna Mogyla, Drana Kokhta, Ordzhonikidze, Zolota Balka, Mamai-Gora, and Bel'sk. It is different from the local baseline ranges for the sites of Nevs'ke and Medvin.

Table SI1. 2. Strontium isotope ratios for samples from the Mariupol Necropolis and the Kalmius Settlement.

Lab ID material	Sample ID	Sample Description	$^{87}Sr/^{86}Sr$	2*se*
ANB002 (I31731) Tooth enamel	I31731, Mariupol Necropolis V2	Neolithic double Burial 50 (immature), permanent maxillary left incisor	0.70950	0.00001
ANB003 (I31732) bone	I31732, Mariupol Necropolis V3	Eneolithic triple Burial 21 (female)	0.70990	0.00001
ANB005 bone	Kalmius Neolithic Settlement V5	Burial K2 (immature)	0.70993	0.00001

The individuals presenting the $^{87}Sr/^{86}Sr$ ratio the closest to sample ANB002 (I31731) are one from Verteba Cave (Scythian), one from Kirovograd (Yamna), one from Vynohradne (Early Catacomb), two from Nevs'ke (Developed Catacomb culture), one from Babyna Mogyla (Scythian) and six from Olennii (Eneolithic and/or Bronze Age).

The individuals presenting the $^{87}Sr/^{86}Sr$ ratio the closest to both samples ANB003 and ANB005 are one from Vynohradne (Eneolithic), one from Kirovograd (Yamna), one from Shakhta Stepna (Catacomb), seven from Mamai-Gora (Iron Age), two from Bel'sk (Iron Age), one from Olexandropol (Scythian), two from Babyna Mogyla (Scythian), and two from Ordzhonikidze (Scythian).

Sample ANB005 is also similar to three individuals from Shakhta Stepna (two from the Yamna culture and one from the Catacomb culture), one from Vynohradne (Yamna), one from Babyna Mogyla (Scythian), one from Ordzhonikidze (Scythian) and one from Zolota Balka (Scythian).

Sample ANB003 (I31732) is also similar to three individuals from Vynohradne (one from the Eneolithic, one from the Yamna culture, and one from the Catacomb culture) and two from Ordzhonikidze (Scythian).

In addition to Sr isotope ratios, stable isotopes ratios of carbon and oxygen were obtained on tooth enamel of specimen ANB002. The obtained values are as follows: $\delta^{13}C$: -10.4 ‰, IRMS $\delta^{18}O$: -8.7 ‰ (IRMS). Since the radiocarbon date obtained on enamel carbonate of I31731 indicates possible contamination with soil carbonates, it is not clear how diagnostic are the $\delta^{13}C$ and $\delta^{18}O$ measurements from the same enamel sample.

In conclusion, strontium ratios of all three individuals from Mariupol have Sr isotope ratio signature compatible with that of the environment in which they were buried, but this signature is also compatible with a large portion of Ukraine and that of the baselines and human

remain samples from multiple sites in Ukraine as well as proximate to the Sea of Azov sites outside of Ukraine.

References

1. Makarenko, M. O. *The Mariupil Burial-Place*. (The Allukrainian Academy of Sciences, Kyiw, 1933).
2. Telegin, D. Ya. & Potekhina, I. D. *Neolithic Cemeteries and Populations in the Dnieper Basin*. (Archaeopress, Oxford, 1987).
3. Telegin, D. Y. *Neoliticheskiye Mogil'niki Mariupol'skogo Tipa*. (Naukova Dumka, Kiev, 1991).
4. Telegin, D. Ya., Potekhina, I. D., Lillie, M. & Kovaliukh, M. M. The chronology of the Mariupol-type cemeteries of Ukraine re-visited. *Antiquity* **76**, 356–363 (2002).
5. Mattila, T. M. *et al.* Genetic continuity, isolation, and gene flow in Stone Age Central and Eastern Europe. *Commun Biol* **6**, 793 (2023).
6. Nikitin, A. G., Sokhatsky, M. P., Kovaliukh, M. M. & Videiko, M. Y. Comprehensive Site Chronology and Ancient Mitochondrial DNA Analysis from Verteba Cave – a Trypillian Culture Site of Eneolithic Ukraine. *Interdisciplinaria Archaeologica. Natural Sciences in Archaeology.* **1**, 9–18 (2010).
7. Lillie, M. C. *et al.* First isotope analysis and new radiocarbon dating of Trypillia (Tripolye) farmers from Verteba Cave, Bilche Zolote, Ukraine. *Documenta Praehistorica* **44**, 306–324 (2018).
8. Mathieson, I. *et al.* The genomic history of southeastern Europe. *Nature* **555**, 197–203 (2018).
9. Nikitin, A. G. *et al.* Mitochondrial DNA analysis of Eneolithic Trypillians from Ukraine reveals Neolithic farming genetic roots. *PLoS One* **12**, e0172952 (2017).
10. Ward, G. K. & Wilson, S. R. Procedures for comparing and combining radiocarbon age determinations: a critique. *Archaeometry* **20**, 19–32 (1978).
11. Patokova, E. F., Petrenko, V. G., Burdo, N. B. & Polischuk, L. Y. *Pamyatniki Tripol'skoj Kul'tury v Severo-Zapadnom Prichernomorje*. (Naukova Dumka, Kiev, 1989).
12. Petrenko, V., Gerling, C. & Kaiser, E. Majaki – ein komplexes Denkmal der Usatovo-Kultur. Isotopendaten werfen neue Fragen auf. in *Eurasia Antiqua. Zeitschrift für Archäologie Eurasiens* (eds. Hansen, S. & Wagner, M.) vol. 21 45–74 (Rudolf Habelt Verlag, Bonn, 2018).
13. Ivanova, S. V., Petrenko, V. G. & Vetchinnikova, N. E. *Kurgans of Ancient Herdsmen from the South Bug and Dniester Interfluve*. (KP OGT, Odessa, 2005).
14. Telegin, D. Ya. *Dereivka. A Settlement and Cemetery of Copper Age Horse Keepers on the Middle Dnieper*. (British Archaeological Reports, Oxford, 1986).
15. Lillie, M., Budd, C., Potekhina, I. & Hedges, R. The radiocarbon reservoir effect: new evidence from the cemeteries of the middle and lower Dnieper basin, Ukraine. *J Archaeol Sci* **36**, 256–264 (2009).
16. Nikitin, A. G., Videiko, M., Patterson, N., Renson, V. & Reich, D. Interactions between Trypillian farmers and North Pontic forager-pastoralists in Eneolithic central Ukraine. *PLoS One* **18**, e0285449 (2023).

17. Wilde, S. *et al.* Direct evidence for positive selection of skin, hair, and eye pigmentation in Europeans during the last 5,000 y. *Proc Natl Acad Sci U S A* **111**, 4832–7 (2014).
18. Neradenko, T. M. Mohyl′nyk Molyukhiv Buhor. *Arkheolohichni doslidzhennya v Ukrayini 2004-2005 rr.* 91–97 (2006).
19. Rassamakin, Y. Y. New radiocarbon dates to the absolute chronology of Eneolithic of the Black Sea steppe (preliminary analysis). *Arkheologicheskiy al'manakh* **20**, 289–296 (2009).
20. Rassamakin, Y. Y. Eneoliticheskiye pogrebeniya basseyna r. Molochnoy. in *Drevneyshiye skotovody stepey yuga Ukrainy* (ed. Shaposhnikova, O. G.) 31–43 (Naukova Dumka, Kiev, 1987).
21. Kotova, N. S. *Dereivskaya Kul'tura i Pamyatniki Nizhnemikhaylovskogo Tipa*. (Maidan, Kiev, Kharkov, 2013).
22. Ivanova, S. V. New radiocarbon dates for the monuments in the North-Western coast of the Black Sea. *Arheologia* **3**, 69–75 (2010).
23. Serova, N. L. & Yarovoy, E. V. *Grigoriopolskie Kurgany*. (Shtiintsa, Kishinev, 1987).
24. Yarovoy, E. V. Main Eneolithic burials of a mound near the village of Bursuceni in central Moldova (preliminary information). *Bulletin of the Moscow State Regional University (History and Political Science)* 54–68 (2019) doi:10.18384/2310-676X-2019-5-54-68.
25. Govedarica, B. & Manzura, I. The Giurgiuleşti cemetery in chronological and cultural context of southeastern and Eastern Europe. *Eurasia Antiqua* **22**, 1–39 (2016).
26. Todorova, H., Dimov, T. & Vaĭsov, I. Arkheologichesko prouchvane na praistoricheskiya nekropol pri s. Durankulak, Tolbukhinski okrŭg. in *XXVI Natsionalna konferentsiya po akhreologiya v Preslav* 17–18 (Sofia, 1981).
27. Kotova, N., Makhortykh, S. & Dzhos, V. New evidence on the interaction between the Yamnaya and Globular Amphora cultures. *Baltic-Pontic Studies* **25**, 317–341 (2021).
28. Nikitin, A. G., Ivanova, S., Kiosak, D., Badgerow, J. & Pashnick, J. Subdivisions of haplogroups U and C encompass mitochondrial DNA lineages of Eneolithic–Early Bronze Age Kurgan populations of western North Pontic steppe. *J Hum Genet* (2017) doi:10.1038/jhg.2017.12.
29. Ivanova, S. Connections between the Budzhak Culture and Central European groups of the Corded Ware Culture. *Baltic-Pontic Studies* **18**, 86–120 (2013).
30. Ivanova, S. V. & Saveliev, O. K. Barrow near Sychavka Village of Odesa Oblast. *Archeologia* **3**, 70–82 (2011).
31. Agre, D. Arheologicheski razkopki na nadgrobna mogila v zemlishcheto na s. Popovo, obshtina Bolyarovo, Yambolska oblast. in *Arheologicheski otkritia i razkopki prez 2006 g.* 74–75 (Sofia, 2007).
32. Alexandrov, S. Fourth/third millennium BC barrow graves in North-East Bulgaria (120 years of investigations). in *Yamnaya Interactions. Proceedings of the International Workshop held in Helsinki, 25–26 April 2019* (eds. Heyd, V., Kulcsár, G. & Preda-Bălănică, B.) 271–314 (Budapest, 2021).
33. Tkachuk, T. M. The end of the stage CI and the beginning of the stage CII of the Tripolye culture in the Upper Dnestr region (according to material of the Bilshivtsi settlement). *Stratum Plus* **2**, 196–217 (2002).
34. Reimer, P. J. *et al.* The IntCal20 Northern Hemisphere Radiocarbon Age Calibration Curve (0–55 cal kBP). *Radiocarbon* **62**, 725–757 (2020).

35. Nikitin, A. G., Newton, J. R. & Potekhina, I. D. Mitochondrial haplogroup C in ancient mitochondrial DNA from Ukraine extends the presence of East Eurasian genetic lineages in Neolithic Central and Eastern Europe. *J Hum Genet* **57**, 610–2 (2012).
36. Kadurin, S., Yanko-Hombach, V. & Smyntyna, O. The Ukraine: In Search of Submerged Late Palaeolithic Sites on the North-Western Black Sea Shelf. in 413–428 (2020). doi:10.1007/978-3-030-37367-2_21.
37. Larchenkov, E. & Kadurin, S. Paleogeography of the Pontic Lowland and northwestern Black Sea shelf for the past 25 k.y. in *Geology and Geoarchaeology of the Black Sea Region: Beyond the Flood Hypothesis* (Geological Society of America, 2011). doi:10.1130/2011.2473(06).
38. Svitoch, A. A. The Neoeuxinian basin of the Black Sea and the Khvalinian transgression of the Caspian Sea. *Quaternary International* **225**, 230–234 (2010).
39. Marin, I. N. & Palatov, D. M. Insights on the Existence of Ancient Glacial Refugee in the Northern Black/Azov Sea Lowland, with the Description of the First Stygobiotic Microcrustacean Species of the Genus Niphargus Schiödte, 1849 from the Mouth of the Don River. *Diversity (Basel)* **15**, 682 (2023).
40. Marret, F. *et al.* The Holocene history of the NE Black Sea and surrounding areas: An integrated record of marine and terrestrial palaeoenvironmental change. *Holocene* **29**, 648–661 (2019).
41. Dolukhanov, P. M. & Shilik, K. K. Environment, sea-level changes, and human migrations in the northern Pontic area during late Pleistocene and Holocene times. in *The Black Sea Flood Question: Changes in Coastline, Climate, and Human Settlement* 297–318 (Springer Netherlands, 2007). doi:10.1007/978-1-4020-5302-3_12.
42. Kiosak, D. Kamyane-Zavallia, the Easternmost Linear Pottery Culture Settlement Ever Excavated. *Sprawozdania Archeologiczne* **69**, 253–269 (2017).
43. Larina, O. V. Larina O.V. The Linear Pottery Culture of the area between rivers Prut and Dniester. *Stratum Plus* **2**, 10–140 (1999).
44. Tovkailo, M. The Neolithic period in Ukraine. in *Prehistoric Ukraine: From the First Hunters to the First Farmers* (eds. Lillie, M. C. & Potekhina, I. D.) 111–154 (Oxbow Books, 2020).
45. Rassamakin, Y. Y. Steppes of the Black Sea Region within a context of development of first agricultural societies . *Arkheolohiya* 3–26 (2004).
46. Govedarica, B. *Zepterträger, Herrscher Der Steppen: Die Frühen Ockergräber Des Älteren Äneolithikums Im Karpatenbalkanischen Gebiet Und Im Steppenraum Südost-Und Osteuropas.* (Verlag Philipp von Zabern, Mainz am Rhein, 2004).
47. Rassamakin, J. Ja. *Die nordpontische Steppe in der Kupferzeit : Gräber aus der Mitte des 5. Jts. bis Ende des 4. Jts. v. Chr. = Azovo-Pontijskie stepi v ėpochu medi : pogrebal'nye pamjatniki serediny V - konca IV tys. do n. e.* (P. von Zabern, Mainz, 2004).
48. Krupnov, E. I. Polnyy nauchnyy otchet o rezul'tatakh Severo-Kavkazskoy arkheologicheskoy ekspeditsii 1948 g. . *Arkhiv Instituta arkheologii RAN* **280**, (1949).
49. Lazaridis, I., Patterson, N., Anthony, D. & & others. *The Genetic Origin of the Indo-Europeans. in Submission*. (2024).
50. Manzura, I. V. Those Who Possess Scepters. *Stratum Plus* **2**, 237–295 (2000).
51. Petrenko, V. G. Problema «Tripol'ye i step'» i pamyatniki eneolita — ranney bronzy Severo-Zapadnogo Prichernomor'ya. *Materialy po arkheologii Severnogo Prichernomor'ya* **9**, 10–38 (2009).

52. Rassamakin, Y. Y. The Eneolithic of the Black Sea Steppe: Dynamics of Cultural and Economic Development 4500–2300 BC. in *Late Prehistoric Exploitation of the Eurasian Steppe* (eds. Levine, M., Rassamakin, Y., Kislenko, A. & Tatarintseva, N.) 59–182 (McDonald Institute Monographs, Cambridge, 1999).
53. Telegin, D. Y. *Seredn'o-Stohivs'ka Kul'tura Epokhy Midi*. (Naukova Dumka, Kyiv, 1973).
54. Nikitin, A. G. & Ivanova, S. Long-distance exchanges along the Black Sea coast in the Eneolithic and the steppe genetic ancestry problem. in *Steppe Transmissions* (eds. Preda-Bălănică, B. & Ahola, M.) 9–27 (Archaeolingua, Budapest, 2023). doi:10.33774/coe-2022-7m315.
55. Rassamakin, Y. Y. The Kvityanskaya Culture: History and Current State of the Problem. *Stratum Plus* **2**, 117–177 (2000).
56. Anthony, D. W. *The Horse, the Wheel, and Language: How Bronze-Age Riders from the Eurasian Steppes Shaped the Modern World*. (Princeton University Press, Princeton and Oxford, 2007).
57. Manzura, I. V. Eastern Europe at the dawn of the kurgan tradition. in *Ex Ungue Leonem. Sbornik statey k 90-letiyu L'va Samuilovicha Kleyna* (ed. Vishnyatsky, L. B.) 107–129 (Nestor-Istoriya, St. Petersburg, 2017).
58. Mileto, S., Kaiser, E., Rassamakin, Y., Whelton, H. & Evershed, R. P. Differing modes of animal exploitation in North-Pontic Eneolithic and Bronze Age Societies. *STAR: Science & Technology of Archaeological Research* **3**, 112–125 (2018).
59. Manzura, I. History Carved by the Dagger: the Society of the Usatovo Culture in the 4th Millennium BC. in *Repräsentationen Der Macht. Beiträge des Festkolloquiums zu Ehren des 65. Geburtstags von Blagoje Govedarica* (ed. Hansen, S.) 73–96 (Deutsches Archäologisches Insitut, Harrassovitz Verlag, Wiesbaden, 2020).
60. Rassamakin, Y. Y. Stepy Pivnichnoho Prychornomor″ya za doby midi. in *Ukrayina: khronolohiya rozvytku. Z davnikh chasiv do pizn'oyi antychnosti* vol. 1 202–2018 (Kyiv, 2008).
61. Kotova, N. S. & Videiko, M. Y. The Absolute Chronology of the Ukraine during the Eneolithic. in *Zwischen Karpaten und Ägäis. Neolithikum und ältere Bronzezeit.* (eds. Hansel, B. & Studenikova. E.) 121–134 (Verlag Marie Leidorf GmbH · ., Rahden/Westf, 2004).
62. Rassamakin, Y. Y. & Evdokimov, G. L. Novyj pozhdneeneoliticheskij mogil'nik na iyge Khersonscheny v svete regional'nykh issledovanij stepnogo eneolita. *Donets'kyj Archeologichnyj Zbirnyk* **13/14**, 7–29.
63. Trifonov, V. A. Western boundaries of extension of the Maikop culture. *Izvestiya Samarskogo nauchnogo tsentra Rossiyskoy akademii nauk* 276–284 (2014).
64. Rassamakin, Y. Y. Between the Don and the Danube: The Phenomenon of the Zhivotilovka-Volchanskoe Type of Burials at the Turn of the Late Eneolithic and the Early Bronze Age in the Northern Black Sea Steppe. in *The Caucasus. Bridge between the urban centres in Mesopotamia and the Pontic steppes in the 4th and 3rd millennium BC. The transfer of knowledge and technologies between East and West in the Bronze Age* (eds. Giemsch, L. & Hansen, S.) 195–210 (Verlag Schnell & Steiner GmbH, Regensburg, 2021).
65. Rassamakin, Y. Y. Podveski bolgradskogo tipa kak spetsificheskiy indikator migratsionnykh protsessov na rubezhe eneolita-rannego bronzovogo veka v pontiyskikh

stepyakh. in *XV Ural'skoye arkheologicheskoye soveshchaniye. Tezisy dokladov konferentsii* (ed. Morgunova, N. L.) 104–106 (Orenburg, 2001).
66. Kovaleva, I. F. Pogrebeniya zhivotilovskogo tipa v Prisamar'ye. in *Kurgannyye drevnosti stepnogo Podneprov'ya (III–I tys. do n.e.)* (ed. Kovaleva, I. F.) 46–54 (Dnepropetrovsk, 1978).
67. Demchenko, T. On the issue of the Bursuchenskaia cultural group identification within Gordinești - Maykopsk village phe- nomenon. in *Culturi, procese şi contexte în arheologie* (eds. Sîrbu, L., Telnov, N., Ciobanu, L., Sîrbu, G. & Kaşuba, M.) 84–99 (Tipogr. 'Garamont-Studio''', Chişinău, 2016).
68. Dergachev, V. A. Maykop in the late Tripolye culture. in *Antiquities of East Europe, South Asia and South Siberia in the context of connections and interactions within the Eurasian cultural space (new data and concepts). Volume II. Connections, contacts and interactions between ancient cultures of northern Eura* (eds. Polyakov, A. V. & Tkach, E. S.) 128–131 (IIMK RAN, Nevskaya Tipographia, St. Petersburg, 2019).
69. Immel, A. *et al.* Gene-flow from steppe individuals into Cucuteni-Trypillia associated populations indicates long-standing contacts and gradual admixture. *Sci Rep* **10**, 4253 (2020).
70. Nikitin, A. G. & Ivanova, S. The Origins and Chronology of the Usatove Culture. *Archaeologia Lituana* **23**, 148–156 (2022).
71. Nikitin, A. G., Ivanova, S., Culleton, B. J., Potekhina, I. & Reich, D. New radiocarbon and stable isotope data from the Usatove culture site of Mayaky in Ukraine. *SSRN Electronic Journal* (2023) doi:10.2139/ssrn.4236123.
72. Penske, S. *et al.* Early contact between late farming and pastoralist societies in southeastern Europe. *Nature* **620**, 358–365 (2023).
73. Dergachev, V. A. *Late Tripolye and Maykop*. (Wydawnictwo Uniwersytetu Rzeszowskiego , Rzeszów, 2021).
74. Petrenko, V. G. Usatovskaya kulʹtura. in *Drevniye kul'tury Severo-Zapadnogo Prichernomor'ya* (eds. Bruyako, I. V. & Samoylova, T. L.) 163–210 (SMIL, Odessa, 2013).
75. Manzura, I. The Mayaki cemetery as a source for social organization of the Usatovo culture. in *Culturi, Procese și Contexte în Arheologie* (eds. Sîrbu, L., Telnov, N., Ciobanu, L., Sîrbu, G. & Kaşuba, M.) 63–83 (Chişinău, 2016).
76. Petrenko, V. G. & Kaiser, E. Kompleksnyy pamyatnik Mayaki: novyye izotopnyye daty i voprosy khronologii nalichnykh kulʹtur. *Materialy po arkheologii Severnogo Prichernomor'ya* **12**, 31–61 (2011).
77. Diachenko, A. & Harper, T. K. The absolute chronology of Late Tripolye sites: a regional approach. *Sprawozdania Archeologiczne* **68**, 81–105 (2016).
78. Potekhina, I. D. Formuvannya eneolitychnoho naselennya Pivnichno-Zakhidnoho Prychornomorʺya za antropolohichnymy ta arkheohenetychnymy danymy mohylʹnykiv usativsʹkoyi kulʹtury. in *Anotatsiyi dopovidey Mizhnarodnoyi konferentsiyi «Vid paleolitu do kozats'koyi Ukrayiny», prysvyachenoyi 100-litn'omu yuvileyu D.YA. Telehina* (eds. Zaliznyak, L. L. & Potekhina, I. D.) 91 (Oleh Filyuk, Kyiv, 2019).
79. Potekhina, I. D. Antropologicheskie materialy iz mogil'nika Mayaki. in *Pamyatniki Tripol'skoj kul'tury v Severo-Zapadnom Prichernomorje* (eds. Patokova, E. F., Petrenko, V. G., Burdo, N. B. & Polischuk, L. Y.) 125–133 (Naukova Dumka, Kiev, 1989).
80. Patokova, E. F. *Usatovskoye Poseleniye i Mogil'niki*. (Naukova Dumka, Kiev, 1979).

81. Dergachev, V. A. Kulturelle und historische Entwicklungen im Raum zwischen Karpaten und Dnepr. Zu den Beziehungen zwischen frühen Gesellschaften im nördlichen Südost- und Osteuropa. in *Das Karpatenbecken und die osteuropäische Steppe. Nomadenbewegungen und Kulturaustausch in den vorchristlichen Metallzeiten (4000–500 v. Chr.). Prähistorische Archäologie in Südosteuropa 12* (eds. Hänsel, B. & Machnik, J.) 27–64 (Marie Leidorf, Rahden/Westf., 1998).
82. Razumov, S. Transversal arrowheads of the Usatovo culture. in *Connections, contacts and interactions between ancient cultures of Northern Eurasia and civilizations of the East during the Palaeometal period (IV–I mil. BC)* 141–143 (Institute for the History of Material Culture Russian Academy of Sciences, 2019). doi:10.31600/978-5-907053-35-9-141-143.
83. Cook, G. T. *et al.* Problems of dating human bones from the Iron Gates. *Antiquity* **76**, 77–85 (2002).
84. Weber, A. W. *et al.* Chronology of middle Holocene hunter–gatherers in the Cis-Baikal region of Siberia: Corrections based on examination of the freshwater reservoir effect. *Quaternary International* **419**, 74–98 (2016).
85. Cook, G. T. *et al.* A Freshwater Diet-Derived ^{14} C Reservoir Effect at the Stone Age Sites in the Iron Gates Gorge. *Radiocarbon* **43**, 453–460 (2001).
86. Ramsey, C. B., Schulting, R., Goriunova, O. I., Bazaliiskii, V. I. & Weber, A. W. Analyzing Radiocarbon Reservoir Offsets Through Stable Nitrogen Isotopes and Bayesian Modeling: A Case Study Using Paired Human and Faunal Remains from the Cis-Baikal Region, Siberia. *Radiocarbon* **56**, 789–799 (2014).
87. Bonsall, C. *et al.* Radiocarbon and Stable Isotope Evidence of Dietary Change from the Mesolithic to the Middle Ages in the Iron Gates: New Results from Lepenski Vir. *Radiocarbon* **46**, 293–300 (2004).
88. Bonsall, C., Macklin, M., Payton, R. & Boroneanţ, A. Climate, floods and river gods. *Before Farming* **2002**, 1–15 (2002).
89. Bonsall, C. *et al.* Stable isotopes, radiocarbon and the Mesolithic-Neolithic transition in the Iron Gates. *Documenta Praehistorica* **27**, 19–132 (2000).
90. Soulet, G., Giosan, L., Flaux, C. & Galy, V. Using Stable Carbon Isotopes to Quantify Radiocarbon Reservoir Age Offsets in the Coastal Black Sea. *Radiocarbon* **61**, 309–318 (2019).
91. Kwiecien, O. *et al.* Estimated Reservoir Ages of the Black Sea Since the Last Glacial. *Radiocarbon* **50**, 99–118 (2008).
92. Cook, G. T. *et al.* The Mesolithic–Neolithic Transition in the Iron Gates, Southeast Europe: calibration and dietary issues. in *Chronology and Evolution within the Mesolithic of North-West Europe* (eds. Crombé, P., Van Strydonck, M., Sergant, J., Boudin, M. & Bats, M.) 497–515 (Cambridge Scholars Publishing, 2009).
93. Borić, D. & Price, T. D. Strontium isotopes document greater human mobility at the start of the Balkan Neolithic. *Proceedings of the National Academy of Sciences* **110**, 3298–3303 (2013).
94. Borić, D., French, C. & Dimitrijević, V. Vlasac revisited: formation processes, stratigraphy and dating. *Documenta Praehistorica* 261–287 (2008).
95. Munchayev, R. M. *Kavkaz Na Zare Bronzovogo Veka: Neolit, Eneolit, Rannyaya Bronza*. (Nauka, Moscow, 1975).

96. Rigaud, S. *et al.* Exploring Hypotheses on Early Holocene Caspian Seafaring Through Personal Ornaments: A Study of Changing Styles and Symbols in Western Central Asia. *Open Archaeology* **9**, (2023).
97. Telegin, D. Y. Yeshche raz o vydelenii pamyatnikov novodanilovskogo tipa epokhi medi . in *Drevneyshiye obshchnosti zemledel'tsev i skotovodov Severnogo Prichernomor'ya (V tys. do n.e. – V v. n.e.). materialy mezhdunarodnoy konferentsii Kishinev 10-14 dekabrya 1990 g.* (ed. Yarovoj, E. V.) 60–61 (Kiev, 1991).
98. Videiko, M. Y. 'Shumers′kyy slid' v Trypil′s′kiy arkhitekturi? *Trypillian Civilization journal* (2010).
99. Palaguta, I. An Assemblage of Anthropomorphic Figurines of the Neolithic and Copper Age Balkan-Carpathians Cultures: some Observations on the Structure of Images and its Development. in *Cucuteni Culture within the European Neo-Eneolithic Context: Proceedings of the International Colloquium „Cucuteni - 130. 15-17 October 2014, Piatra-Neamţ, Romania: In Memoriam dr. Dan Monah, In Memoriam dr. Gheorghe Dumitroaia* (eds. Preoteasa, C. & Nicola, C.-D.) 327–348 (Editura "Constantin Matasă", Piatra-Neamţ, 2016).
100. Rezepkin, A. Maykop Culture: Issue of Chronology and Cultural Relationships of the Ust-Dzheguta and Psekupsk Variants. *Slovenská archeológia* **68**, 515–526 (2020).
101. The British Museum Online Collection. https://www.britishmuseum.org/collection/search?object=pin&place=Middle%20East&view=grid&sort=object_name__asc&page=2#page-top.
102. *Treasures from the Royal Tombs of Ur*. (University of Pennsylvania, Philadelphia, 1998).
103. Dergachev, V. A. New hoards of metal objects of The Cucuteni-Trypillian Culture found in The Middle Dniester region (Ukraine). in *Culturi, procese şi contexte în arheologie* (eds. Sîrbu, L., Telnov, N., Ciobanu, L., Sîrbu, G. & Kașuba, M.) 36–51 (Tipogr. 'Garamont-Studio', Chişinău, 2016).
104. Chernykh, L. A. & Daragan, M. N. *Kurgany Epokhi Eneolita-Bronzy Mezhdurech'ya Bazavluka, Solenoy, Chertomlyka : Monografiya. Kurgany Ukrainy.* vol. 4 (Oleg Filyuk, Kiev, 2014).
105. Petrenko, V. G., Sapozhnikov, I. V. & Sapozhnikova, G. V. Geometricheskiye mikrolity usatovskoy kul'tury. in *Drevneye Prichernomor'ye. Kratkiye soobshcheniya Odesskogo arkheologicheskogo obshchestva* 42–47 (Odessa, 1994).
106. Manzura, I., Nosova, L. & Petrenko, V. G. Zwei unbekannte Gräber der Usatovo-Kultur auf dem Gräberfeld von Majaki (Ukraine). *Altertum* **68**, 1–14 (2023).
107. Sinica, V. S., Razumov, S. N. & Telnov, N. P. *Archaeological Heritage of Pridnestrovie*. (GUIPP «Bend. tipografiya «Poligrafist», Tiraspol, 2016).
108. *Early Urbanism in Europe. The Trypillia Megasites of the Ukrainian Forest-Steppe*. (De Gruyter Poland Ltd, Warsaw/Berlin, 2020). doi:10.1515/9783110664959.
109. Ivanova, S. & Nikitin, A. G. The Yamna culture: Origin and migrations in the context of the Frontier Theory. *Notes of the History Faculty, Odessa I. I. Mechnikov National University* **31**, (2021).
110. Faifert, A. V. Early stage of the Yamanya culture from the Lower Don Region. (Voronezh State University, 2017).
111. Diaconescu, D. Step by Steppe: Yamnaya culture in Transylvania. *Praehistorische Zeitschrift* **95**, 17–47 (2020).

112. Palalidis, S. A GIS-based approach to the study of the Yamnaya cultural horizon (ca. 3300-2400 BC): preliminary results. in *InFieri. Incontri di Archeologia Sapienza Miscellanea degli atti II (2018-2019) e III (2020)* (eds. Abbondanzieri, E. & et al.) (Edizioni Quasar di Severino Tognon s.r.l., Roma, 2023).
113. Topal, D. Patterns of kurgan landscape between Dniester, Prut and Danube rivers. in *Connections, contacts and interactions between ancient cultures of Northern Eurasia and civilizations of the East during the Palaeometal period (IV–I mil. BC)* 29–32 (Institute for the History of Material Culture Russian Academy of Sciences, 2019). doi:10.31600/978-5-907053-35-9-29-32.
114. Ivanova, S. Intrusions of the steppe population into the Balkan-Carpathian region in the Early Bronze Age: factors and aspects. *Sprawozdania Archeologiczne* **75**, 77–114 (2023).
115. Topal, D. Main characteristics of the Yamnaya barrows in the North-West Pontic area. in *Communication for the Trilateral Research Conference: MOBAB | Mobility in the Balkans during the Bronze Age* (Villa Vigoni, Loveno di Menaggio, Italy, 2022).
116. Dvorianinov, S. A., Petrenko, V. G. & Rychkov, N. A. K izucheniyu orientirovki yamnykh pogrebeniy. in *Drevnosti Severo-Zapadnogo Prichernomoria* (ed. Stanko, V. N.) 22–38 (Naukova Dumka, Kiev, 1981).
117. Subbotin, L. V., Razumov, S. N. & Sinika, V. S. *Semyonovka Barrows*. (Stratum Plus, Tiraspol, 2017).
118. Yarovoy, E. V. *Drevneyshie Skotovodcheskie Plemena Yugo-Zapada SSSR (Klassifikatsiya Pogrebalnogo Obriada)*. (Shtiintsa, Kishinev, 1985).
119. Ivanova, S. V. *Sotsial'naya Struktura Naseleniya Yamnoy Kul'tury Severo-Zapadnogo Prichernomor'ya*. (Druk, Odessa, 2001).
120. Dergachev, V. A. *Moldaviya i Sosednie Territorii v Epokhu Bronzy*. (Shtiintsa, Kishinev, 1986).
121. Nikolova. A. V. Khronologicheskaya klassifikatsiya pamyatnikov yamnoy kul'tury stepnoy zony Ukrainy. (Institute of Archaeology, National Academy of Sciences of Ukraine, Kyiv, 1992).
122. Ivanova, S. *Istoriya Naselennya Pivnichno-Zakhidnoho Prychornomor"ya Naprykintsi IV–III Tys. Do n. e.* . (Buk-Druk, Zhytomyr, 2021).
123. Włodarczak, P. Kultura złocka i problem genezy kultury ceramiki sznurowej w Małopolsce. in *Na pograniczu światów. Studia z pradziejów międzymorza bałtycko-pontyjskiego ofiarowane Profesorowi Aleksandrowi Kośko w 60 rocznicę urodzin* (eds. Bednarczyk, J., Czebreszuk, J., Makarowicz, P. & Szmyt, M.) 511–532 (Wydawnictwo Poznańskie, Poznań, 2008).
124. Rassamakin, Y. Y. & Nikolova, A. V. Carpathian Imports and Imitations in Context of Eneolithic and Bronze Age of the Black See Area. in *Import and Imitation in Archeology* (eds. Biehl, P. & Rassamakin, Y. Y.) 51–88 (Beier & Beran, Langenweibach, 2008).
125. Gening, V. V. & Korpusova, V. N. *Arkheologicheskiye Pamyatniki Krymskogo Prisivash'ya. Kurgany u s. Istochnoye i s. Bolotnoye*. (Institut Zoologii, Kiyev, 1989).
126. Bratchenko, S. N. *Donets'ka Katakombna Kul'tura Rann'oho Etapu*. (Shlyakh, Luhans'k, 2001).
127. Otroshchenko, V. V. Doba bronzy na terenakh Ukrayiny . in *Ukrayina. Khronolohiya rozvytku* (eds. Otroshchenko, V. V., Rassamakin, Y. Y. & Chernykh, L. A.) vol. 1 219–331 (KVSHTS, Kyiv).

128. Kaiser, E. Datarea absolută a culturii înmormântărilor în catacombe din nordul Mării Negre. **3(18)**, 59–70 (2009).
129. Sanzharov, S. N. *Poseleniya Neolita – Ranney Bronzy Severskogo Dontsa*. (SNU, Lugansk, 2000).
130. Lytvynenko, R. O. The Babyne culture circle. (Institute of Archaeology, National Academy of Sciences of Ukraine, Kyiv, 2009).
131. Pustovalov, S. Kurhanni svyatylyshcha doby rann′oyi bronzy stepovoyi chastyny Ukrayiny ta yikh astronomichna skladova. in *Ukrayins'ke nebo. Studiyi nad istoriyeyu astronomiyi v Ukrayini* (ed. Petruk, O.) 39–50 (Instytut prykladnykh problem mekhaniky i matematyky im. YA. S. Pidstryhacha NAN Ukrayiny, Lviv, 2014).
132. Drovosekova, O. V. Materialy epokhi eneolita i bronzovogo veka iz s. kapulovka Dnepropetrovskoy oblasti. in *Starozhytnosti stepovoho Prychornomor"ya i Krymu* (ed. Tolochko, P. P.) vol. 10 131–154 (Zaporizhzhia, 2002).
133. Teslenko, D. L., Kaiser, E. & Shalobudov, V. M. Kurgan sites in the Samara River Basin: Results of interdisciplinary research . *Archaeology and Early History of Ukraine* **46**, 146–177 (2023).
134. Vajsov, I. Das Grab 982 und die Protobronzezeit in Bulgarien. in *Durankulak, Band II. Die prähistorischen Gräberfelder* (ed. Todorova, H.) 159–176 (Publishing House Anubis Ltd., Sofia, 2002).
135. Alexandrov, S. Forth-Second Millennium BC Barrow Graves in North Bulgaria. in *The River and the Time* (ed. Atanasov, V.) 27–39 (Tutrakan, 2010).
136. Suvorov, A. Modelling the Yamnaya Expansion Through Radiocarbon Dates. (University of Helsinki, Helsinki, 2021).
137. Agulnikov, S. & Popovich, S. *Raskopki Kurganov v Rayone Shtefan-Vode, Respublika Moldova: (1989, 1990, 1991)*. (Agenția Națională Arheologică, Chisinau, 2022).
138. Agulnikov, S. M. Issledovanie kurganov v s. Kotiuzhen'. *Archeologicheskie Issledovaniya v Moldove v 1986 g.* 104–120 (1992).
139. Popovici, S. & Ceban, I. Results of the Archaeological Investigations on the Tripolye C2 Site at Cunicea Prișanscaia-Gora. *Arheologia Moldovei* **37**, 205–217 (2014).
140. Dergachev, V. A. Raskopki v Danchenakh i nekotoryye voprosy izucheniya pamyatnikov pozdnego Tripol'ya i Katakombnoy kul'tury. in *Arkheologicheskiye Issledovaniya v Moldavii v 1974-1976 gg.* 35–45 (Кишинев, 1981).
141. Derhachev, V. A. & Manzura, I. V. *Pogrebal'nyye Kompleksy Pozdnego Tripol'ya*. (Štiinca, Chișinău, 1991).
142. Haheu, V. & Kurciatov, S. Cimitirulplan Eneolitic de lingă satul Giurgiuleşti. *Revista Arkheologică* 101–114 (1993).
143. Lysenko, S. D., Razumov, S. N., Lysenko, S. S., Sinika, V. S. & Тельнов, Н. П. Burials from the Late Bronze Age from the barrows of the "DOT", "SAD", "VODOVOD" groups near the Glinoye village on the Left Bank of the Lower Dniester . *Proceedings in Archaeology and History of Ancient and Medieval Black Sea Region* 164–201 (2022) doi:10.53737/2713-2021.2021.54.48.004.
144. Beylekchi, V. S. Raskopki kurgana 3 v s. Mierkulesht'. *Archeologicheskie Issledovaniya v Moldove v 1986 g.* 72–87 (1992).
145. Dergachev, V. A. & Sava, E. N. Excavation of tumuli in the vicinity of the villages of Mereni and Chirca. *Stratum Plus* **2**, 526–562 (2002).

146. Manzura, I. V., Klochko, E. O. & Savva, E. N. *Kamenskie Kurgany*. (Shtiintsa, Kishinev, 1992).
147. Levițki, O., Manzura, I. & Demcenko, T. Necropola tumulară de la Sărăteni. in *Bibliotheca Thracologica XVII* (București, 1996).
148. Sava, E., Agulnikov, S. & Manzura, I. *Issledovaniya Kurganov v Budzhakskoy Stepi (1980-1985 Gg.)*. (Bons Offices, Chişinău, 2019).
149. Savva, E. N. Issledovanie kurgana u g. Tiraspolya. *Archeologicheskie Issledovaniya v Moldavii v 1983 g.* 44–59 (1988).
150. Makhortykh, S. V., Kotova, N. S., Dzhos, V. S. & Radchenko, S. B. New burial and ritual assemblages of Early Bronze Age located near the complex of Kamyana Mohyla. *Archaeology and Early History of Ukraine* **37**, 226–239 (2020).
151. Mykhailova, N. Mykola Makarenko and Mariupol Neolithic Burial Ground The tragic fates of the archaeologist and the site. *Archaeologia Lituana* **24**, 19–33 (2024).
152. Zbenovich, V. G. Issledovanie pozdnetripol'skogo poselenija usatovskogo tipa b s. Mayaki. in *Arkheologicheskie Issledovanija na Ukraine v 1965-1966 gg.* (ed. Tolochko, P. P.) 83–87 (Naukova Dumka, Kiev, 1967).
153. Zbenovich, V. G. *Pozdnetripol'skiye Plemena Severnogo Prichernomor'ya*. (Naukova Dumka, Kiev, 1974).
154. Zinkovsky, K. V. & Patokova, E. F. Issledovaniya Mayakskogo mogil'nika v 1974 g. . in *Arkheologicheskiye issledovaniya Severo-Zapadnogo Prichernomor'ya* 134–143 (Naukova Dumka, Kiev, 1978).
155. Zin'kovskiy, K. V. & Petrenko, V. G. Burials with traces of ochre in Usatovo burial grounds. *Sovetskaya Arkheologia* **4**, 24–39 (1987).
156. Neradenko, T. M. The settlement Molyukhiv Buhor in the works of D. Ya. Telegin and according to modern data. *Archaeology and Early History of Ukraine* **37**, 348–357 (2020).
157. Korobkova, G. F. & Shaposhnikova, O. G. *Poselenie Mikhailovka: Etalonnyj Pamyatnik Drevneyamnoj Kultury*. (Evropejskij Dom, St. Petersburg, 2005).
158. Toshchev, G. N. Yeshche raz o stratigrafii Odesskogo kurgana. in *Kurgany v zonakh novostroyek Moldavii* 175–182 (Shtiintsa , Kishinev, 1984).
159. Biagi, P. & Kiosak, D. The Mesolithic of the northwestern Pontic region: New AMS dates for the origin and spread of the blade and trapeze industries in southeastern Europe. *Eurasia Antiqua* 21–41 (2010).
160. Rassamakin, Y. Y. Mohyl′nyky Ihren′ (Ohrin′) 8 ta Oleksandriya doby eneolitu: problemy datuvannya ta kul′turnoyi prynalezhnosti. *Archeologia* 26–48 (2017).
161. Nikitin, A. G. & Ivanova, S. The megalithic past of the Bronze Age kurgans of the North Pontic Region. in *19th Annual Meeting of the European Association of Archaeologists* 178–179 (Plzen, Czech Republic, 2013).
162. Ivanova, S. V. New radiocarbon dates for the monuments in the north-western coast of the Black Sea. *Archeologia* **3**, 69–75 (2010).
163. Nikitin, A. G. Bioarchaeological Analysis of Bronze Age Human Remains from the Podillya Region of Ukraine. *Interdisciplinaria Archaeologica. Natural Sciences in Archaeology.* **2**, 9–14 (2011).
164. Pustovalov, S. Zh. Molochans′ke svyatylyshche. *Problemy Arkheolohiyi Podniprov″ya* 104–118 (1999).
165. Rassamakin, Y. Y. Das Frühäneolithikum der Don-Dnepr-Region (zweites bis drittes Viertel des 5 Jts. V. Chr.): einige Proleme der Interpretaon von archäologischen

Denkmälern . in *Repräsentationen der Macht. Beiträge des Festkolloquiums zu Ehren des 65. Geburtstages von Blagoje Govedarica* (ed. Hansen, S.) 27–43 (Harrassovitz Verlag, Wiesbaden, 2020).
166. Wang, C.-C. *et al.* Ancient human genome-wide data from a 3000-year interval in the Caucasus corresponds with eco-geographic regions. *Nat Commun* **10**, 590 (2019).
167. Broushaki, F. *et al.* Early Neolithic genomes from the eastern Fertile Crescent. *Science (1979)* **353**, 499–503 (2016).
168. Mathieson, I. *et al.* Genome-wide patterns of selection in 230 ancient Eurasians. *Nature* **528**, 499–503 (2015).
169. Haak, W. *et al.* Massive migration from the steppe was a source for Indo-European languages in Europe. *Nature* **522**, 207–11 (2015).
170. Lazaridis, I. *et al.* Genomic insights into the origin of farming in the ancient Near East. *Nature* **536**, 419–424 (2016).
171. Fu, Q. *et al.* The genetic history of Ice Age Europe. *Nature* **534**, 200–205 (2016).
172. Mallick, S. *et al.* The Simons Genome Diversity Project: 300 genomes from 142 diverse populations. *Nature* (2016) doi:10.1038/nature18964.
173. Ward, R. H., Frazier, B. L., Dew-Jager, K. & Pääbo, S. Extensive mitochondrial diversity within a single Amerindian tribe. *Proceedings of the National Academy of Sciences* **88**, 8720–8724 (1991).
174. Günther, T. *et al.* Population genomics of Mesolithic Scandinavia: Investigating early postglacial migration routes and high-latitude adaptation. *PLoS Biol* **16**, e2003703 (2018).
175. Allentoft, M. E. *et al.* Population genomics of Bronze Age Eurasia. *Nature* **522**, 167–172 (2015).
176. Krause, J. *et al.* A Complete mtDNA Genome of an Early Modern Human from Kostenki, Russia. *Current Biology* **20**, 231–236 (2010).
177. Jones, E. R. *et al.* The Neolithic Transition in the Baltic Was Not Driven by Admixture with Early European Farmers. *Current Biology* **27**, 576–582 (2017).
178. Juras, A. *et al.* Maternal genetic origin of the late and final Neolithic human populations from present-day Poland. *Am J Phys Anthropol* **176**, 223–236 (2021).
179. Allentoft, M. E. *et al.* Population genomics of post-glacial western Eurasia. *Nature* **625**, 301–311 (2024).
180. Krzewińska, M. *et al.* Ancient genomes suggest the eastern Pontic-Caspian steppe as the source of western Iron Age nomads. *Sci Adv* **4**, (2018).
181. Włodarczak, P. Kurgan rites in the Eneolithic and Early Bronze Age Podolia in light of materials from the funerary ceremonial centre at Yampil. *Baltic-Pontic Studies* **22**, 246–283 (2018).
182. Knipper, C. *et al.* Female exogamy and gene pool diversification at the transition from the Final Neolithic to the Early Bronze Age in central Europe. *Proceedings of the National Academy of Sciences* **114**, 10083–10088 (2017).
183. Battaglia, V. *et al.* Y-chromosomal evidence of the cultural diffusion of agriculture in southeast Europe. *European Journal of Human Genetics* **17**, 820–830 (2009).
184. Lipson, M. *et al.* Parallel palaeogenomic transects reveal complex genetic history of early European farmers. *Nature* **551**, 368–372 (2017).
185. Guarino-Vignon, P. *et al.* Genome-wide analysis of a collective grave from Mentesh Tepe provides insight into the population structure of early neolithic population in the South Caucasus. *Commun Biol* **6**, 319 (2023).

186. Dulias, K. *et al.* Ancient DNA at the edge of the world: Continental immigration and the persistence of Neolithic male lineages in Bronze Age Orkney. *Proceedings of the National Academy of Sciences* **119**, (2022).
187. Nikitin, A. G. *et al.* Interactions between earliest Linearbandkeramik farmers and central European hunter gatherers at the dawn of European Neolithization. *Sci Rep* **9**, 19544 (2019).
188. Paschou, P. *et al.* Maritime route of colonization of Europe. *Proceedings of the National Academy of Sciences* **111**, 9211–9216 (2014).
189. Fernández, E. *et al.* Ancient DNA Analysis of 8000 B.C. Near Eastern Farmers Supports an Early Neolithic Pioneer Maritime Colonization of Mainland Europe through Cyprus and the Aegean Islands. *PLoS Genet* **10**, e1004401 (2014).
190. Gaskevych, D. L. North-Pontic Impresso: Origin of the Neolithic Pottery with Comb Decoration in the South of Eastern Europe. *Stratum Plus* **2**, 213–251 (2010).
191. Burdo, N. B. Late Neolithic cultural elements from the Danube and Carpathian regions of Precucuteni – Trypillia A culture. *Documenta Praehistorica* **XXXVIII**, 357–371 (2011).
192. Neparáczki, E. *et al.* Y-chromosome haplogroups from Hun, Avar and conquering Hungarian period nomadic people of the Carpathian Basin. *Sci Rep* **9**, 16569 (2019).
193. de Barros Damgaard, P. *et al.* The first horse herders and the impact of early Bronze Age steppe expansions into Asia. *Science (1979)* eaar7711 (2018) doi:10.1126/science.aar7711.
194. Underhill, P. A. *et al.* The phylogenetic and geographic structure of Y-chromosome haplogroup R1a. *European Journal of Human Genetics* **23**, 124–131 (2015).
195. Gorbov, V. & Kolesnik, A. New Neolithic Settlement in Mariupol and Its Place in the System of Synchronous Monuments. *Science Journal of VolSU. History. Area Studies. International Relations* **21**, 16–31 (2016).
196. De Muynck, D., Huelga-Suarez, G., Van Heghe, L., Degryse, P. & Vanhaecke, F. Systematic evaluation of a strontium-specific extraction chromatographic resin for obtaining a purified Sr fraction with quantitative recovery from complex and Ca-rich matrices. *J Anal At Spectrom* **24**, 1498 (2009).
197. Ventresca Miller, A. R. *et al.* Re-evaluating Scythian lifeways: Isotopic analysis of diet and mobility in Iron Age Ukraine. *PLoS One* **16**, e0245996 (2021).
198. Gerling, C. *Prehistoric Mobility and Diet in the West Eurasian Steppes 3500 to 300 BC*. (De Gruyter, 2015). doi:10.1515/9783110311211.

Supplementary File SI2

Population genetic analysis of populations from the North Pontic and adjacent regions

Written by Iosif Lazaridis with input from David Reich, Nick Patterson, and Alexey G. Nikitin

Contents

Overview *99*

Modeling framework *99*

An exploratory Principal Component Analysis *100*

Populations that form a clade (N=1) with one of the sources *102*
Mykhailivka_I32534 (3635-3383 calBCE) 102

Admixture f_3-statistics *103*

Populations that can be modeled as mixtures of two of the sources (N=2) *110*
Bulgaria_C_ProtoYamna (3500-3000 BCE) 110
Bulgaria_EBA_Yamna (3300-2000 BCE) 111
Bulgaria_Riltsi_EBA_Yamna (3300-2500 BCE) 113
Moldova_EBA_Yamna (3400-2500BCE) 113
Moldova_EBA_Yamna_o (2865-2576 calBCE) 115
Moldova_GlobularAmphora_Yamna (2906-2702 calBCE) 115
Ukraine_EBA_Yamna (3300-2500 BCE) 116
Ukraine_MBA_Catacomb_o1 (2201-2032 calBCE) 117
Ukraine_MBA_MultiCordonedWare_Babine (2400-1900BCE) 117
Ukraine_N_I27992 (5363-5216 calBCE) 118
Usatove (4400-3600 BCE) 118
Usatove_I20078 (3340-3034 calBCE) 119
Zhivotilovka_I17974 (3334-3030 calBCE) 120

Populations that cannot be well-modeled *120*
GlobularAmphora (3400-2600 BCE) 120
Trypillians (4700-2700 BCE) 121
Ukraine_EBA_Catacomb_possible (2874-2630 calBCE) 122
Zhivotilovka_I17973 (3354-3103 calBCE) 123

Re-analysis of Early Bronze Age outliers from Ukraine from Mathieson et al. (2018) (ref.[16]) *124*

Re-analysis of data from Penske et al. (2023) (ref.[22]) *127*
Chalcolithic Southeastern Europe (4900-3800 BCE) 127
Kartal clusters A and B (4150-3400 BCE) 128
Early Bronze Age (BOY_EBA and MAJ_EBA) (3300-2500 BCE) 130

Timing the admixture of Caucasus-Lower Volga and European farmer groups *133*

Summary of key findings *134*
The ancestral landscape: a compendium 135

Appendix I: Co-modeling populations with Dnipro-Don hunter-gatherer and European farmer ancestry *137*
Choosing additional Right populations 137
Modeling Peri-Pontic populations 140

Appendix II: Co-modeling populations with Dnipro-Don hunter-gatherer and Steppe Maykop ancestry *143*

Appendix III: Do the core Yamna have Trypillian ancestry? *144*

Appendix IV: Source of hunter-gatherer ancestry in the core Yamna along the UNHG-GK2-EHG continuum.. *145*
Appendix V: European farmer ancestry in the Ukraine Neolithic hunter-gatherers *147*
References *150*

Table SI2. 1. Populations that can be modeled as simple clades (*N*=1) with one of the sources. 102
Table SI2. 2. Model competition for Mykhailivka_I32534. Each column shows the p-value of a model when alternative models (rows) are placed on the Right set. 103
Table SI2. 3. Significantly negative (Z<-3) admixture f3-statistics. 104
Table SI2. 4. Populations that can be modeled as 2-way mixtures clades (*N*=2) of the sources..............108
Table SI2. 5. Model competition for Bulgaria_EBA_Yamna. Each column shows the p-value of a model when alternative models (rows) are placed on the Right set. 112
Table SI2. 6. Admixture of Bulgarian Yamna individuals. 112
Table SI2. 7. Model competition for Bulgaria_Riltsi_EBA_Yamna. Each column shows the p-value of a model when alternative models (rows) are placed on the Right set. 113
Table SI2. 8. Model competition for Moldova_EBA_Yamna. Each column shows the p-value of a model when alternative models (rows) are placed on the Right set. 114
Table SI2. 9. Admixture proportions for Moldova_EBA_Yamna. Individuals 10208, I10398, I12637, I7847 are part of the Core Yamna group and used as a source here...114
Table SI2. 10. Model competition for Moldova_EBA_Yamna_o. Each column shows the p-value of a model when alternative models (rows) are placed on the Right set. 115
Table SI2. 11. Model competition for Moldova_GlobularAmphora_Yamna. Each column shows the p-value of a model when alternative models (rows) are placed on the Right set..116
Table SI2. 12. Model competition for Ukraine_EBA_Yamna. Each column shows the p-value of a model when alternative models (rows) are placed on the Right set. 116
Table SI2. 13. Admixture of Ukrainian Yamna individuals. Individuals included in the Core Yamna group (I12168, I20975, I2105, I3141_enhanced) used as a source are not shown. 117
Table SI2. 14. 3-way models with varying hunter-gatherer ancestry for Bronze Age Ukraine. 118
Table SI2. 15. Model competition for Usatove_I20078. Each column shows the p-value of a model when alternative models (rows) are placed on the Right set. 119
Table SI2. 16. Model competition for Zhivotilovka_I17974. Each column shows the p-value of a model when alternative models (rows) are placed on the Right set. 120
Table SI2. 17. Modeling Trypillia_I20069. 121
Table SI2. 18. Feasible models at p=0.01 level for Ukraine_EBA_Catacomb_possible. 122
Table SI2. 19. Model competition for Ukraine_EBA_Catacomb_possible. Each column shows the p-value of a model when alternative models (rows) are placed on the Right set. 123
Table SI2. 20. Feasible models at p=0.01 level for Zhivotilovka_I17973. 123
Table SI2. 21. Feasible 2-way models for Ukraine_EBA_Ozera_I1917. 124
Table SI2. 22. Model competition for Ukraine_EBA_Ozera_I1917. Each column shows the p-value of a model when alternative models (rows) are placed on the Right set. 124
Table SI2. 23. 3-way models applicable to all three Ukraine_EBA outlier individuals. The model that is feasible for all three individuals in the tournament approach is highlighted in bold. 125
Table SI2. 24. Model competition for Ukraine_EBA_Deriivka_I4110. Each column shows the p-value of a model when alternative models (rows) are placed on the Right set. 126
Table SI2. 25. Model competition for Ukraine_EBA_Deriivka_I5882. Each column shows the p-value of a model when alternative models (rows) are placed on the Right set. 126
Table SI2. 26. Model competition for Ukraine_EBA_Deriivka_I5884. Each column shows the p-value of a model when alternative models (rows) are placed on the Right set. 127
Table SI2. 27. KTL_A is derived from a BPgroup+Trypillia mixture even when adding sources defining the CLV (Aknashen) or Volga (Lebyazhinka) clines or allowing for Dnipro-Don hunter-gatherer (Ukraine_N or GK2) ancestry. 128

Table SI2. 28. 3-way admixture models for KTL_B. 129
Table SI2. 29. Model competition for KTL_B. Each column shows the p-value of a model when alternative models (rows) are placed on the Right set. 129
Table SI2. 30. Modeling BOY_EBA and MAJ_EBA. 130
Table SI2. 31. Model competition for BOY_EBA. Each column shows the p-value of a model when alternative models (rows) are placed on the Right set. 131
Table SI2. 32. Feasible 3-way models for MAJ_EBA with Core Yamna as one of the sources. 131
Table SI2. 33. Feasible 2-way models for MAJ_EBA when Don Yamna and Serednii Stih are included as additional proximate sources. 132
Table SI2. 34. Model competition for MAJ_EBA. Each column shows the p-value of a model when alternative models (rows) are placed on the Right set. 132
Table SI2. 35. Admixture of MAJ_EBA individuals. 133
Table SI2. 36. 4-source model with Right=Base. High standard errors necessitate the introduction of an outgroup population to Base that can differentiate between the four sources. 137
Table SI2. 37. Adding outgroups to Base set. We model the “PeriPontic” meta-population with diverse added outgroups to Base. Top-20 models of lowest RMSE are shown. Proximity to median is the sum of the squared difference between admixture proportions for each added outgroup to the median proportions across all 20 outgroups. The two chosen outgroups for addition are highlighted in bold and minimize the RMSE (China_Xinjiang_Xiaohe_BA.SG) and proximity to median (Tarim_EMBA1). 140
Table SI2. 38. Statistics showing how added Right outgroup populations differentiate between the populations of the 4-source model. 140
Table SI2. 39. 4-source modeling of Peri-Pontic populations with Trypillian ancestry as the 4th source. 140
Table SI2. 40. 4-source modeling of Peri-Pontic populations with Steppe Maykop ancestry as the 4th source. 143
Table SI2. 41. Adding populations to Base set. We add populations to the Right and examine the standard errors. Top-20 populations with lower standard errors are shown. 146
Table SI2. 42. 3-source models for Ukraine_N. All sources other than GK2 were considered. We manually tested the LBK+Lebyazhinka+Serbia Iron Gates and CHG+Lebyazhinka+Serbia Iron Gates models and include them in this table 147

Figure SI2. 1. An exploratory PCA. 101
Figure SI2. 2. DATES estimates of admixture timing of CLV and European farmer ancestry admixture. 134
Figure SI2. 3. P-value and Root Mean Square Error (RMSE) of 4-way model (Aknashen, BPgroup, GK2, Trypillia) when adding diverse outgroups to the Base set. Models with <10% RMSE are shown. 139
Figure SI2. 4. P-values for models with Serbia Iron Gates Mesolithic+Lebyazhinka HG + either LBK or CHG sources for individuals of Ukraine_N. p=0.05 vertical and horizontal lines are shown at log10(0.05)= -1.30103. 149

Overview

The North Pontic region (NPR) forms the western end of the Eurasian steppe and connects that great expanse of flatlands to its east with the world of central and southeastern Europe to its west. We study Eneolithic and Bronze Age populations that lived north and west of the Black Sea to arrive at key insights about their population history. First, we identify single individuals at Giurgiuleşti and Csongrád that crossed the NPR from the east without any discernible admixture with native populations, suggesting that long-distance migration occurred even before it became common during the Yamna expansion of the Bronze Age. However, in the main Pre-Yamna expansion period populations of the NPR were formed by admixture of varied Caucasus-Lower Volga (CLV) people from the east with native populations of the NPR. The Trypillian farmers received a little ancestry from these CLV migrants, but also contributed about half of the ancestry of Usatove-related populations that were formed on the basis of an even Trypillian-CLV admixture. CLV migrants also admixed with NPR hunter-gatherers (forming the Serednii Stih populations, and eventually the Yamna themselves). These NPR hunter-gatherers were not isolated from the farmers of Europe but also received substantial input from them. The unprecedented mobility of the Yamna expansion included many individuals without any admixture of the pre-Yamna populations, but also many of diverse additional ancestry: from European farmers (west of the NPR), Ukraine hunter-gatherers (from the NPR itself), but also Maykop and Steppe Maykop admixture (from east of the NPR). The Yamna were thus formed from proximal Caucasus-Lower Volga and North Pontic Region sources who themselves included ancestry from nearly every native group of the Eurasian steppe, and more distally from ancestors further afield in Southeastern Europe, the Caucasus, and Central Asia. Formed by the confluence of diverse ancestors in the NPR, the Yamna and their descendants then expanded, and often incorporated others in their society in the NPR, proving that their remarkable demographic success did not fully eclipse the people that preceded them.

Modeling framework

We used the same qpWave/qpAdm setup[1,2] modeling framework as in the linked paper (ref.[3] to study populations of the North Pontic region (NPR). Briefly, the set of Right outgroup populations is:

Right: OldAfrica[4-6], Russia_AfontovaGora3[7], CHG[8], Iran_GanjDareh_N[9], Italy_Villabruna[7], Russia_Sidelkino.SG[10], Turkey_N[11]

This includes an African outgroup, and representatives of "Ancient North Eurasians" (Afontova Gora 3), inland West Asian populations (CHG and Ganj Dareh), Western hunter-gatherers (Villabruna), Eastern hunter-gatherers (Sidelkino), and Anatolian farmers (Turkey_N).

The set of candidate source populations is:

Sources: Armenia_Aknashen_N[12], BPgroup[13] and ref.[3], CoreYamna (high data quality set of 104 individuals identified in ref.[3] and including individuals from ref.[3] and ref.[2,10,11,13-18]), GK2 (ref.[3]), GlobularAmphora[16,18], Lebyazhinka_HG[2,16], Maykop[13], PVgroup[13] (grouped in ref.[3]), Remontnoye (ref.[3]), Russia_Karelia[2,16,19], Serbia_IronGates_Mesolithic[16], TTK[20], Trypillia[21] and ref.[3], Ukraine_N[16] and ref.[3], YUN_CA[22]

The set of sources includes population sources for each of the clines involving Pontic-Caspian steppe populations:

- The Volga cline, formed on the basis of North Caucasus-Lower Volga Eneolithic populations (Berezhnovka-2/Progress-2 group / BPgroup or Progress-2/Vonyuchka-1 / PVgroup) admixing with Eastern European hunter-gatherers (EHG) (Russia_Karelia as a remote source of EHG, or Lebyazhinka_HG from the Middle Volga). We also include the Neolithic individual from Tutkaul (TTK) as the Volga cline includes ancestry from an eastern Siberian/Central Asian source that can be modeled with TTK as a source.

- The Dnipro cline, formed with Ukraine Neolithic hunter-gatherer admixture (either Ukraine_N or GK2 from Golubaya Krinitsa on the Middle Don, itself ~2/3 Ukraine_N in ancestry) with a population of the Caucasus-Lower Volga cline (below), using either Core Yamna (as the sampled end point of the cline) or Remontnoye, a population midway on the Caucasus-Lower Volga Cline.
- The Caucasus-Lower Volga (CLV) cline, formed on the basis of largely "Caucasus Neolithic" and descended populations (Aknashen-related) and "Lower Volga Eneolithic" populations (BPgroup-related) with intermediate groups in the North Caucasus at Maykop (largely Caucasus Neolithic) and PVgroup (largely lower Volga Eneolithic) and north of the Manych Depression (at Remontnoye)

To these populations we add three groups representing European farmers: Trypillians (from Moldova and Ukraine), Globular Amphora (from Poland and Ukraine), and YUN_CA (Chalcolithic individuals from Yunatsite, Bulgaria). We also include Iron Gates hunter-gatherers from Serbia (also known as Balkan hunter-gatherers) to account for the possibility that populations of different hunter-gatherer ancestry than the three Neolithic groups could be contributing ancestry to the region.

The Core Yamna, one of the chosen sources, form a tight cluster in PCA and were determined in ref.[3] as comprising of Yamna or Afanasievo individuals that form a clade (using the same Right set as here) to a high degree of confidence (p=0.2 threshold and at least 400,000 autosomal SNPs) with the Samara Yamna (the Yamna population with the highest number of individuals that is part of the Yamna PCA cluster). This cluster does include some high-quality individuals from north and west of the Black sea; we will analyze below Yamna individuals from the region that are *not* assigned to the Core Yamna, while also noting those that do.

As we did not want to assume that these 12 source populations admixed only in the combinations manifested in the three Pontic-Caspian clines, we allowed combinations (of N=1, 2, 3) populations in our modeling of the following set of Test populations:

Test: Bulgaria_C_ProtoYamna, Bulgaria_EBA_Yamna, Bulgaria_Riltsi_EBA_Yamna, Giurgiuleşti, GlobularAmphora, Igren_o, Moldova_Crasnoe_Eneolithic, Moldova_EBA_Yamna, Moldova_EBA_Yamna_o, Moldova_GlobularAmphora_Yamna, Mykhailivka_I32534, SShi, SSlo, SSmed, Trypillia, Ukraine_EBA_Catacomb, Ukraine_EBA_Catacomb_possible, Ukraine_EBA_Yamna, Ukraine_MBA_Catacomb_o1, Ukraine_MBA_MultiCordonedWare_Babine, Ukraine_N_I27992, Usatove, Usatove_EBA, Usatove_I20078, Usatove_Yamna, Zhivotilovka_I17973, Zhivotilovka_I17974

Some of these populations (in particular the Serednii Stih subsets SShi, SSmed, SSlo) were also considered in the linked manuscript ref.[3] but we wanted to see how they might be related in terms of their origin to the others from the NPR.

An exploratory Principal Component Analysis

We used smartpca[23] to perform principal component analysis (PCA) using the same set of populations to form the axes as in ref.[3] (Figure SI2. 1):

OberkasselCluster (set of trans-Alpine WHG individuals identified in[20]), Russia_Firsovo_N, Iran_HajjiFiruz_C[17], Iran_C_SehGabi[9], Iran_C_TepeHissar[24], Israel_C[25], Germany_EN_LBK[2,11,26,27]

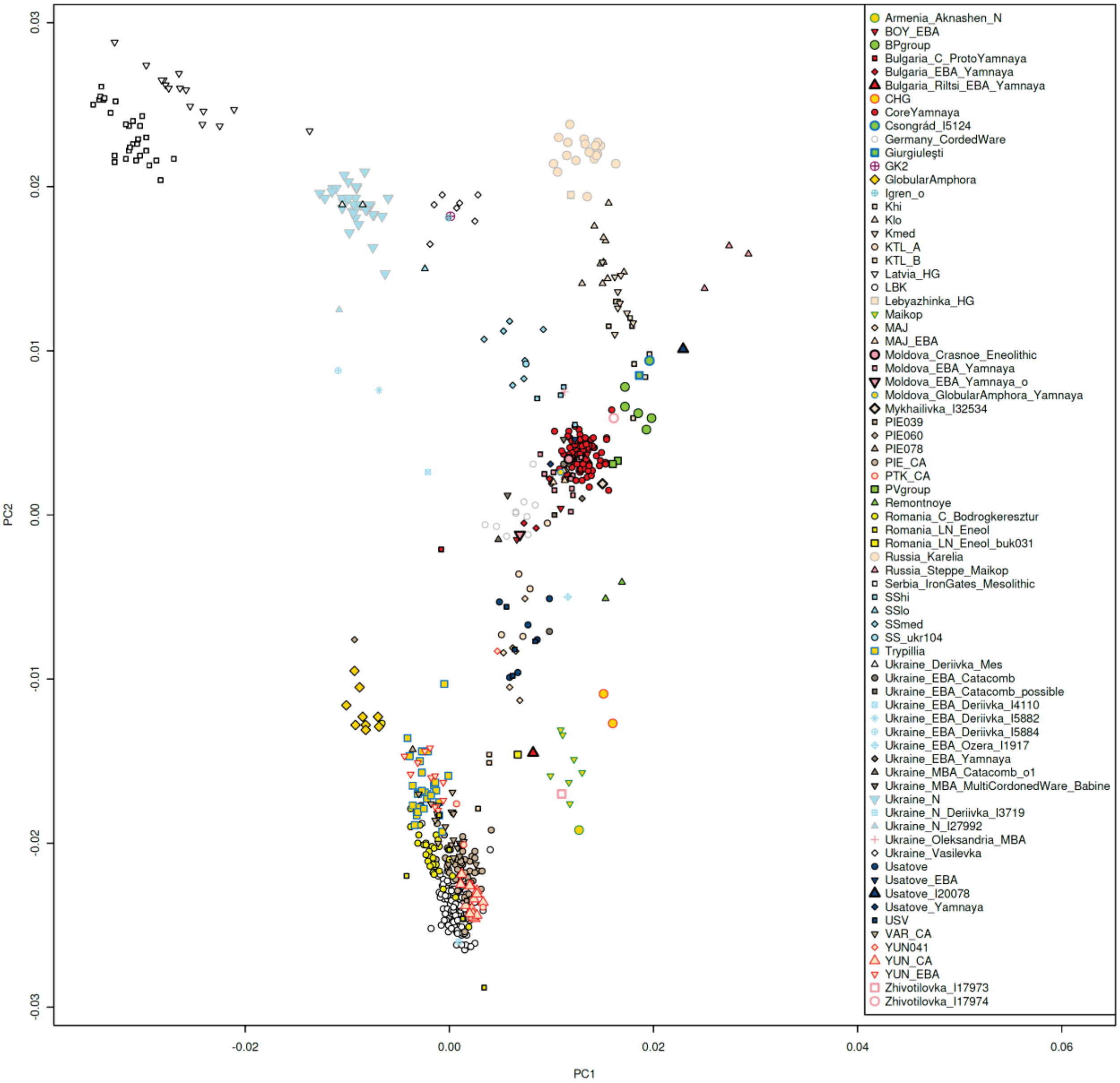

Figure SI2. 1. An exploratory PCA.

We observe that individuals from the North Pontic region and from southeastern Europe are mostly not along the three steppe clines, except, of course, the aforementioned Serednii Stih individuals that define the Dnipro cline. A Serednii Stih individual I28319 published by Mattila et al.[28] as SS_ukr104 clusters with the SSmed subset and forms a clade with it using qpWave (p=0.281).

Most individuals of interest are to the left and bottom of the PCA (with respect to the Dnipro cline), towards the cline formed by European farmers (LBK and YUN_CA) on one side and Balkan hunter-gatherers (Serbia Iron Gates Mesolithic). Clearly ancestry from European farmers of mixed hunter-gatherer ancestry played a role in their formation which we formally explore below. We highlight here a few individuals that are unusual in that they are shifted *away* from the farmer-hunter-gatherer cline of southeastern Europe. Two of these are individual I17974 from Bursuceni and individual I20078 from Taraclia, both from III-C type burials from Moldova (p. 14). Two other individuals (from Giurgiuleşti in Moldova and Csongrád in Hungary, both from the Ochre Grave type steppe burials from the second half

of the 5th millennium BCE (p. 11) are also similarly shifted and near the BPgroup (Lower Volga-North Caucasus Eneolithic group) at one end of the Volga cline.

The Core Yamna clearly played a key role in events of the Bronze Age. One individual of interest is Mykhailivka_I32534 from Ukraine and another Moldova_Crasnoe_Eneolithic (individual I20196) which cluster with the core Yamna itself. Note that the core Yamna contains individuals from Ukraine, Moldova, Romania, and Bulgaria (ref.[3]). At the same time, many other western Yamna-related individuals deviate towards the European farmer cline, and as we will see, we find examples of Yamna ancestry combining with diverse pre-existing populations both west and east of the NPR.

Populations that form a clade (N=1) with one of the sources.

The following Test populations form a clade (using a p-value cutoff of 0.05) with one of the sources (Table SI2. 1):

Table SI2. 1. Populations that can be modeled as simple clades (*N*=1) with one of the sources.

Test	Source	P-value
Usatove_Yamna	CoreYamna	0.178
Ukraine_EBA_Catacomb	CoreYamna	0.075
Giurgiuleşti	BPgroup	0.896
Giurgiuleşti	PVgroup	0.689
Usatove_EBA	CoreYamna	0.864
Usatove_EBA	PVgroup	0.067
Moldova_Crasnoe_Eneolithic	CoreYamna	0.683
Igren_o	GK2	0.991
Mykhailivka_I32534	BPgroup	0.163
Mykhailivka_I32534	CoreYamna	0.684
Mykhailivka_I32534	PVgroup	0.659

Usatove_Yamna is individual I12229_enhanced (3088-2911 calBCE) with a potentially Usatove cultural affiliation but a Yamna genetic profile and its label reflecting these affinities. Also cladal with the Core Yamna are the individuals of population "Ukraine_EBA_Catacomb" (p=0.075). The individual from Giurgiuleşti (I20072) is cladal with the Lower Volga-North Caucasus Eneolithic groups (BPgroup and PVgroup) and represents an example of long-range migration from the east into Moldova (4330-4058 calBCE). Usatove_EBA (individual I20079) postdating the expansion of the Yamna and the aforementioned Usatove_Yamna individual (2571-2355 calBCE) is also cladal with the Yamna.

Moldova_Crasnoe_Eneolithic (individual I20196; 3352-3101 calBCE) is dated to the Yamna expansion and has a Yamna genetic profile. Igren_o (I27930) is analyzed in ref.[3] and appears to be of hunter-gatherer ancestry similar to the GK2 individual from Golubaya Krinitsa and unlike the Serednii Stih individuals of similar age from Igren.

Mykhailivka_I32534 (3635-3383 calBCE)

Finally, the low coverage individual I32534 is a clade with Yamna, although we can also fit it as a clade with the Lower Volga-Caucasus Eneolithic groups BPgroup and PVgroup. This individual has a very old date of 3635-3383 calBCE which potentially establishes the early presence of genetically Yamna individuals in the Dnipro region which is consistent with the idea of Yamna being a late Serednii Stih population from the Dnipro-Don area.

To determine if some of the models of Table SI2. 1 can be rejected, we used a model competition approach in which we tested the resilience of each model (with BPgroup, Core Yamna, and PVgroup as the source) when placing the alternative sources in the Right set. The results (Table SI2. 2) indicate that only the Core Yamna model is resilient to either BPgroup or PVgroup being placed on the Right; i.e., neither BPgroup nor PVgroup share any common genetic history with the I32534 that cannot be well-modeled as Core Yamna. By contrast, both BPgroup and PVgroup are rendered infeasible in at least one of their competition comparisons (and their p-values are low 0.1-0.11 for the other one). This comparison suggests that only the Core Yamna model is unambiguously feasible for this individual.

Table SI2. 2. Model competition for Mykhailivka_I32534. Each column shows the p-value of a model when alternative models (rows) are placed on the Right set.

	BPgroup	CoreYamna	PVgroup
BPgroup		5.5E-01	6.9E-05
CoreYamna	1.0E-01		1.1E-01
PVgroup	4.2E-02	7.7E-01	

We were also concerned that the individual could fit as a clade with the Core Yamna even though it might not be "exactly" Core Yamna but may harbor other ancestry that is not, however, sufficient to cause the basic Core Yamna model to fail.

First, we considered whether the individual could harbor less NPR hunter-gatherer ancestry than the Core Yamna, as it is on that far end of the cline (Figure SI2. 1; beyond the main Core Yamna cluster and away from NPR hunter-gatherers like Ukraine_N and GK2). Adding either Ukraine_N (-6.2±7.9%; p=0.689) or GK2 (-3.5±9.9%; p=0.543) is consistent with this position, but the negative proportions of NPR ancestry are non-significant. Thus, we can say that it is *possible* that this early individual might have slightly less NPR hunter-gatherer ancestry than the Core Yamna cluster, this is *unproven*. To the limits of our resolution, the Mykhailivka individual is within the Core Yamna variation.

Second, we considered whether the individual might harbor some European farmer ancestry. This is a topic that we address below for other populations of Ukraine, Moldova, and Bulgaria, i.e., from the western portion of the Yamna range. However, when we add European farmer sources as a 2nd source, their inferred proportion is again negative and non-significant (-5.4±5.1% for Globular Amphora; -4.9±5.6% for Trypillia; -3.3±4.8% for YUN_CA). Thus, there is no evidence at all for the presence of farmer ancestry in the Mykhailivka individual which appears, in this comparison as well, to be unadmixed Core Yamna.

NOTE: Late in the consideration of this paper for publication we generated new data on the I32534 individual (genetic ID I32534_enhanced.TW) raising the number of SNPs overlapping the 1240k capture to 187,106. We checked that the main conclusions of our analysis held with the enhanced data; the individual continues to be a clade (p=0.25) with Core Yamna and cluster with it in PCA. We can additionally reject of Table SI2.1 one of the models (BPgroup; p=0.022) but not the other (PVgroup; p=0.438). In terms of model competition (Table SI2. 1), the PVgroup model fails marginally (p=0.041), but the CoreYamna one does not (p=0.094) when CoreYamna and PVgroup are placed respectively on the Right. These observations are consistent with the conclusions drawn from the lower coverage version of the individual.

Admixture f_3-statistics

To test for the presence of admixture we computed f_3-statistics[29] of the form f_3(Source$_1$, Source$_2$; Test) for all Test populations with at least two individuals using inbreed: YES in qp3Pop. A significantly negative statistic of this form proves that admixture is present in the Test population, thus we show the lowest value of the Z-score of these statistics in Extended Data Table 1. Note that the pair of sources that

minimizes the statistic are not necessarily those that are involved in the admixture. It is further possible that populations have admixture by more than two sources. The formal modeling in terms of sources using qpAdm will follow in this note. However, the simple f_3-statistics are useful as an initial test of the presence of admixture and complement the visual impressions of Figure SI2. 1 in which some populations appear to be "intermediate" between others, a situation that may arise in the presence of admixture. Our statistics prove -if significantly negative- that admixture took place; however, if they are not significantly negative or even positive it is still possible that the population is admixed, either between sources not available to us or because of post-admixture genetic drift which masks the signal of admixture.[29]

It is clear from Extended Data Table 1 that admixture is present in many of the Test populations. We show all negative f3-statistics (Z<-3) in Table SI2. 3.

Table SI2. 3. Significantly negative (Z<-3) admixture f3-statistics.

Test	Source1	Source2	f_3(Source$_1$,Source$_2$; Test	Z-score
BOY_EBA	Maikop	TTK	-0.008641	-3.1
BOY_EBA	Lebyazhinka_HG	Maikop	-0.007111	-3.2
BOY_EBA	Lebyazhinka_HG	YUN_CA	-0.007646	-3.8
BOY_EBA	Armenia_Aknashen_N	Russia_Karelia	-0.009700	-5.2
BOY_EBA	Russia_Karelia	YUN_CA	-0.006633	-5.4
BOY_EBA	Maikop	Russia_Karelia	-0.007918	-5.7
BOY_EBA	GlobularAmphora	TTK	-0.015147	-5.9
BOY_EBA	TTK	YUN_CA	-0.016241	-6.7
BOY_EBA	Serbia_IronGates_Mesolithic	TTK	-0.015798	-6.7
BOY_EBA	TTK	Trypillia	-0.016097	-7.0
Bulgaria_EBA_Yamna	Armenia_Aknashen_N	Serbia_IronGates_Mesolithic	-0.005933	-3.2
Bulgaria_EBA_Yamna	GK2	Remontnoye	-0.006798	-3.3
Bulgaria_EBA_Yamna	Armenia_Aknashen_N	TTK	-0.013115	-3.4
Bulgaria_EBA_Yamna	BPgroup	Serbia_IronGates_Mesolithic	-0.004690	-3.5
Bulgaria_EBA_Yamna	Maikop	Serbia_IronGates_Mesolithic	-0.005223	-3.5
Bulgaria_EBA_Yamna	PVgroup	Serbia_IronGates_Mesolithic	-0.005649	-3.7
Bulgaria_EBA_Yamna	GK2	Maikop	-0.007256	-3.8
Bulgaria_EBA_Yamna	PVgroup	YUN_CA	-0.005650	-3.8
Bulgaria_EBA_Yamna	GlobularAmphora	PVgroup	-0.005953	-3.8
Bulgaria_EBA_Yamna	Remontnoye	Ukraine_N	-0.005622	-3.8
Bulgaria_EBA_Yamna	GlobularAmphora	Lebyazhinka_HG	-0.008478	-4.0
Bulgaria_EBA_Yamna	PVgroup	Trypillia	-0.005726	-4.0
Bulgaria_EBA_Yamna	Armenia_Aknashen_N	Lebyazhinka_HG	-0.012810	-4.1
Bulgaria_EBA_Yamna	Lebyazhinka_HG	Trypillia	-0.007850	-4.1
Bulgaria_EBA_Yamna	CoreYamna	Trypillia	-0.005127	-4.4
Bulgaria_EBA_Yamna	GK2	YUN_CA	-0.007725	-4.5
Bulgaria_EBA_Yamna	GlobularAmphora	Russia_Karelia	-0.006210	-4.6
Bulgaria_EBA_Yamna	Maikop	Ukraine_N	-0.006579	-4.7
Bulgaria_EBA_Yamna	BPgroup	Trypillia	-0.006051	-4.7
Bulgaria_EBA_Yamna	Remontnoye	Serbia_IronGates_Mesolithic	-0.007206	-4.8
Bulgaria_EBA_Yamna	Lebyazhinka_HG	Maikop	-0.011224	-4.8
Bulgaria_EBA_Yamna	CoreYamna	GlobularAmphora	-0.005899	-4.8
Bulgaria_EBA_Yamna	Armenia_Aknashen_N	GK2	-0.012229	-4.9
Bulgaria_EBA_Yamna	Armenia_Aknashen_N	Ukraine_N	-0.008989	-5.1
Bulgaria_EBA_Yamna	CoreYamna	YUN_CA	-0.006165	-5.1
Bulgaria_EBA_Yamna	Serbia_IronGates_Mesolithic	TTK	-0.014443	-5.7
Bulgaria_EBA_Yamna	BPgroup	YUN_CA	-0.007671	-5.8
Bulgaria_EBA_Yamna	Russia_Karelia	Trypillia	-0.007378	-5.8
Bulgaria_EBA_Yamna	BPgroup	GlobularAmphora	-0.008182	-5.9
Bulgaria_EBA_Yamna	Lebyazhinka_HG	YUN_CA	-0.012305	-6.2
Bulgaria_EBA_Yamna	GlobularAmphora	TTK	-0.016893	-6.3
Bulgaria_EBA_Yamna	TTK	Trypillia	-0.015892	-6.4
Bulgaria_EBA_Yamna	TTK	YUN_CA	-0.016344	-6.5
Bulgaria_EBA_Yamna	Armenia_Aknashen_N	Russia_Karelia	-0.012830	-7.2
Bulgaria_EBA_Yamna	Maikop	Russia_Karelia	-0.010932	-7.6
Bulgaria_EBA_Yamna	Russia_Karelia	YUN_CA	-0.011836	-9.2
CoreYamna	Armenia_Aknashen_N	GK2	-0.004997	-3.4
CoreYamna	Armenia_Aknashen_N	Ukraine_N	-0.002192	-3.4
CoreYamna	Russia_Karelia	Trypillia	-0.001419	-3.8

CoreYamna	Armenia_Aknashen_N	Lebyazhinka_HG	-0.009012	-5.4
CoreYamna	Lebyazhinka_HG	YUN_CA	-0.004897	-5.9
CoreYamna	Lebyazhinka_HG	Maikop	-0.006080	-6.1
CoreYamna	GlobularAmphora	TTK	-0.006999	-7.0
CoreYamna	TTK	YUN_CA	-0.007528	-8.8
CoreYamna	Serbia_IronGates_Mesolithic	TTK	-0.008643	-9.1
CoreYamna	TTK	Trypillia	-0.007869	-10.2
CoreYamna	Russia_Karelia	YUN_CA	-0.004369	-10.4
CoreYamna	Armenia_Aknashen_N	Russia_Karelia	-0.008849	-11.7
CoreYamna	Maikop	Russia_Karelia	-0.006310	-13.6
GlobularAmphora	Serbia_IronGates_Mesolithic	YUN_CA	-0.005914	-8.2
KTL_A	GK2	Trypillia	-0.003633	-3.2
KTL_A	BPgroup	Serbia_IronGates_Mesolithic	-0.003121	-3.4
KTL_A	GlobularAmphora	Lebyazhinka_HG	-0.005451	-3.8
KTL_A	GK2	Maikop	-0.005506	-4.0
KTL_A	Lebyazhinka_HG	Maikop	-0.006503	-4.2
KTL_A	Armenia_Aknashen_N	Lebyazhinka_HG	-0.009745	-4.2
KTL_A	Serbia_IronGates_Mesolithic	YUN_CA	-0.003915	-4.6
KTL_A	Lebyazhinka_HG	Trypillia	-0.006059	-4.7
KTL_A	Armenia_Aknashen_N	GK2	-0.010073	-5.2
KTL_A	Serbia_IronGates_Mesolithic	TTK	-0.009574	-5.8
KTL_A	PVgroup	Serbia_IronGates_Mesolithic	-0.006417	-6.1
KTL_A	Remontnoye	Serbia_IronGates_Mesolithic	-0.007217	-7.2
KTL_A	CoreYamna	Trypillia	-0.005272	-7.6
KTL_A	GK2	YUN_CA	-0.009099	-7.7
KTL_A	Maikop	Ukraine_N	-0.007547	-8.5
KTL_A	Lebyazhinka_HG	YUN_CA	-0.011654	-8.6
KTL_A	CoreYamna	GlobularAmphora	-0.006496	-8.8
KTL_A	GlobularAmphora	Russia_Karelia	-0.007968	-8.9
KTL_A	GlobularAmphora	TTK	-0.014412	-9.0
KTL_A	Armenia_Aknashen_N	Serbia_IronGates_Mesolithic	-0.011441	-9.0
KTL_A	Maikop	Russia_Karelia	-0.009051	-9.4
KTL_A	TTK	Trypillia	-0.014159	-9.5
KTL_A	Maikop	Serbia_IronGates_Mesolithic	-0.009057	-9.9
KTL_A	GlobularAmphora	PVgroup	-0.009919	-9.9
KTL_A	Armenia_Aknashen_N	Ukraine_N	-0.011916	-10.0
KTL_A	Ukraine_N	YUN_CA	-0.008018	-10.1
KTL_A	BPgroup	Trypillia	-0.008121	-10.3
KTL_A	PVgroup	Trypillia	-0.009156	-10.4
KTL_A	Armenia_Aknashen_N	Russia_Karelia	-0.013487	-10.5
KTL_A	TTK	YUN_CA	-0.015581	-10.7
KTL_A	CoreYamna	YUN_CA	-0.007454	-10.7
KTL_A	Russia_Karelia	Trypillia	-0.008838	-10.8
KTL_A	PVgroup	YUN_CA	-0.010446	-11.5
KTL_A	BPgroup	GlobularAmphora	-0.009961	-11.5
KTL_A	BPgroup	YUN_CA	-0.010492	-13.0
KTL_A	Russia_Karelia	YUN_CA	-0.014186	-17.5
KTL_B	Maikop	YUN_CA	-0.003385	-3.1
KTL_B	Armenia_Aknashen_N	Ukraine_N	-0.004875	-3.1
KTL_B	Maikop	Trypillia	-0.003554	-3.3
KTL_B	PVgroup	Trypillia	-0.003903	-3.4
KTL_B	GlobularAmphora	Maikop	-0.003888	-3.4
KTL_B	CoreYamna	GlobularAmphora	-0.003660	-3.6
KTL_B	Maikop	Serbia_IronGates_Mesolithic	-0.004580	-3.6
KTL_B	GlobularAmphora	PVgroup	-0.004941	-4.0
KTL_B	BPgroup	Trypillia	-0.004211	-4.1
KTL_B	GlobularAmphora	TTK	-0.008496	-4.3
KTL_B	TTK	Trypillia	-0.008044	-4.3
KTL_B	Armenia_Aknashen_N	Serbia_IronGates_Mesolithic	-0.007009	-4.4
KTL_B	Remontnoye	YUN_CA	-0.005468	-4.6
KTL_B	Lebyazhinka_HG	YUN_CA	-0.008166	-5.0
KTL_B	BPgroup	GlobularAmphora	-0.006027	-5.4
KTL_B	Serbia_IronGates_Mesolithic	YUN_CA	-0.006101	-5.5
KTL_B	GK2	YUN_CA	-0.008770	-6.4
KTL_B	PVgroup	YUN_CA	-0.007972	-6.8
KTL_B	TTK	YUN_CA	-0.013052	-7.1

KTL_B	Ukraine_N	YUN_CA	-0.007740	-7.4
KTL_B	CoreYamna	YUN_CA	-0.007513	-7.8
KTL_B	BPgroup	YUN_CA	-0.009376	-9.0
KTL_B	Russia_Karelia	YUN_CA	-0.009922	-9.1
MAJ	GlobularAmphora	TTK	-0.005897	-4.0
MAJ	Russia_Karelia	Trypillia	-0.003183	-4.2
MAJ	Maikop	Serbia_IronGates_Mesolithic	-0.003844	-4.4
MAJ	TTK	Trypillia	-0.006015	-4.6
MAJ	GlobularAmphora	PVgroup	-0.004787	-5.2
MAJ	Armenia_Aknashen_N	Russia_Karelia	-0.006176	-5.2
MAJ	Armenia_Aknashen_N	Ukraine_N	-0.006010	-5.6
MAJ	PVgroup	Trypillia	-0.004653	-5.6
MAJ	Armenia_Aknashen_N	Serbia_IronGates_Mesolithic	-0.006427	-5.7
MAJ	GK2	YUN_CA	-0.005917	-6.0
MAJ	TTK	YUN_CA	-0.008553	-6.1
MAJ	Ukraine_N	YUN_CA	-0.004529	-6.4
MAJ	CoreYamna	YUN_CA	-0.004196	-6.8
MAJ	BPgroup	Trypillia	-0.004805	-6.9
MAJ	BPgroup	GlobularAmphora	-0.006099	-7.7
MAJ	Lebyazhinka_HG	YUN_CA	-0.009271	-8.1
MAJ	PVgroup	YUN_CA	-0.007234	-8.8
MAJ	BPgroup	YUN_CA	-0.008380	-12.3
MAJ	Russia_Karelia	YUN_CA	-0.009438	-12.7
Moldova_EBA_Yamna	BPgroup	Serbia_IronGates_Mesolithic	-0.002102	-3.1
Moldova_EBA_Yamna	GK2	Maikop	-0.003499	-3.2
Moldova_EBA_Yamna	BPgroup	Trypillia	-0.002141	-3.5
Moldova_EBA_Yamna	Russia_Karelia	Trypillia	-0.002403	-3.8
Moldova_EBA_Yamna	Armenia_Aknashen_N	GK2	-0.006795	-3.8
Moldova_EBA_Yamna	Maikop	Ukraine_N	-0.002679	-4.0
Moldova_EBA_Yamna	PVgroup	Serbia_IronGates_Mesolithic	-0.003288	-4.0
Moldova_EBA_Yamna	BPgroup	YUN_CA	-0.002755	-4.2
Moldova_EBA_Yamna	BPgroup	GlobularAmphora	-0.003481	-4.9
Moldova_EBA_Yamna	Armenia_Aknashen_N	Lebyazhinka_HG	-0.010154	-5.2
Moldova_EBA_Yamna	Armenia_Aknashen_N	Ukraine_N	-0.005418	-5.4
Moldova_EBA_Yamna	Lebyazhinka_HG	Maikop	-0.007595	-5.9
Moldova_EBA_Yamna	Lebyazhinka_HG	YUN_CA	-0.007280	-6.4
Moldova_EBA_Yamna	GlobularAmphora	TTK	-0.009166	-6.8
Moldova_EBA_Yamna	Serbia_IronGates_Mesolithic	TTK	-0.010293	-7.6
Moldova_EBA_Yamna	TTK	YUN_CA	-0.009653	-7.8
Moldova_EBA_Yamna	TTK	Trypillia	-0.009772	-8.1
Moldova_EBA_Yamna	Russia_Karelia	YUN_CA	-0.005772	-8.6
Moldova_EBA_Yamna	Armenia_Aknashen_N	Russia_Karelia	-0.009660	-8.9
Moldova_EBA_Yamna	Maikop	Russia_Karelia	-0.007198	-10.0
PIE_CA	Lebyazhinka_HG	YUN_CA	-0.001961	-3.3
PIE_CA	CoreYamna	YUN_CA	-0.000845	-3.4
PIE_CA	BPgroup	YUN_CA	-0.001158	-3.5
PIE_CA	GK2	YUN_CA	-0.002016	-4.0
PIE_CA	Russia_Karelia	YUN_CA	-0.002388	-6.3
PIE_CA	Ukraine_N	YUN_CA	-0.002111	-6.4
PIE_CA	Serbia_IronGates_Mesolithic	YUN_CA	-0.002351	-6.9
SShi	TTK	Trypillia	-0.006530	-3.3
SShi	Armenia_Aknashen_N	Ukraine_N	-0.005370	-3.5
SShi	Russia_Karelia	YUN_CA	-0.004976	-3.9
SShi	PVgroup	Serbia_IronGates_Mesolithic	-0.005535	-4.0
SShi	Armenia_Aknashen_N	Lebyazhinka_HG	-0.010008	-4.0
SShi	Maikop	Russia_Karelia	-0.005602	-4.1
SShi	BPgroup	Serbia_IronGates_Mesolithic	-0.006004	-4.9
SShi	Serbia_IronGates_Mesolithic	TTK	-0.011729	-5.8
SShi	Armenia_Aknashen_N	Russia_Karelia	-0.010140	-6.3
SSmed	Armenia_Aknashen_N	GK2	-0.008076	-3.3
SSmed	Ukraine_N	YUN_CA	-0.004136	-3.5
SSmed	TTK	YUN_CA	-0.008692	-3.6
SSmed	GK2	Remontnoye	-0.007015	-3.8
SSmed	GK2	Maikop	-0.007250	-4.0
SSmed	GlobularAmphora	Lebyazhinka_HG	-0.008404	-4.1
SSmed	Remontnoye	Russia_Karelia	-0.005689	-4.1

SSmed	GlobularAmphora	TTK	-0.010775	-4.3
SSmed	TTK	Ukraine_N	-0.009840	-4.4
SSmed	Lebyazhinka_HG	Maikop	-0.010004	-4.5
SSmed	Armenia_Aknashen_N	Lebyazhinka_HG	-0.014347	-4.8
SSmed	Lebyazhinka_HG	Trypillia	-0.009367	-4.8
SSmed	Armenia_Aknashen_N	Serbia_IronGates_Mesolithic	-0.008215	-5.0
SSmed	TTK	Trypillia	-0.011786	-5.3
SSmed	Maikop	Serbia_IronGates_Mesolithic	-0.007260	-5.6
SSmed	CoreYamna	Ukraine_N	-0.006231	-5.9
SSmed	Lebyazhinka_HG	YUN_CA	-0.011894	-6.0
SSmed	GlobularAmphora	Russia_Karelia	-0.008100	-6.5
SSmed	Maikop	Ukraine_N	-0.008213	-6.5
SSmed	Remontnoye	Ukraine_N	-0.008754	-6.7
SSmed	PVgroup	Ukraine_N	-0.009437	-7.0
SSmed	CoreYamna	Serbia_IronGates_Mesolithic	-0.007461	-7.1
SSmed	Armenia_Aknashen_N	Ukraine_N	-0.011203	-7.1
SSmed	BPgroup	Ukraine_N	-0.008400	-7.3
SSmed	Maikop	Russia_Karelia	-0.009773	-7.4
SSmed	Armenia_Aknashen_N	Russia_Karelia	-0.013743	-8.1
SSmed	Remontnoye	Serbia_IronGates_Mesolithic	-0.011216	-8.3
SSmed	Russia_Karelia	Trypillia	-0.009862	-8.6
SSmed	Russia_Karelia	YUN_CA	-0.010519	-8.7
SSmed	PVgroup	Serbia_IronGates_Mesolithic	-0.012754	-9.1
SSmed	Serbia_IronGates_Mesolithic	TTK	-0.021782	-9.8
SSmed	BPgroup	Serbia_IronGates_Mesolithic	-0.012501	-10.6
Trypillia	BPgroup	YUN_CA	-0.002135	-5.9
Trypillia	Armenia_Aknashen_N	Serbia_IronGates_Mesolithic	-0.005434	-6.7
Trypillia	CoreYamna	YUN_CA	-0.001755	-7.1
Trypillia	Lebyazhinka_HG	YUN_CA	-0.005731	-8.7
Trypillia	GK2	YUN_CA	-0.005328	-9.4
Trypillia	Russia_Karelia	YUN_CA	-0.004783	-12.5
Trypillia	Ukraine_N	YUN_CA	-0.006006	-19.1
Trypillia	Serbia_IronGates_Mesolithic	YUN_CA	-0.008350	-23.8
Ukraine_EBA_Yamna	Armenia_Aknashen_N	Ukraine_N	-0.004698	-3.3
Ukraine_EBA_Yamna	BPgroup	Trypillia	-0.003759	-3.4
Ukraine_EBA_Yamna	Lebyazhinka_HG	YUN_CA	-0.005863	-3.5
Ukraine_EBA_Yamna	BPgroup	YUN_CA	-0.004216	-3.7
Ukraine_EBA_Yamna	Lebyazhinka_HG	Maikop	-0.006927	-3.8
Ukraine_EBA_Yamna	Armenia_Aknashen_N	Lebyazhinka_HG	-0.009373	-3.8
Ukraine_EBA_Yamna	Russia_Karelia	Trypillia	-0.004508	-4.0
Ukraine_EBA_Yamna	BPgroup	GlobularAmphora	-0.004784	-4.1
Ukraine_EBA_Yamna	Serbia_IronGates_Mesolithic	TTK	-0.009665	-4.8
Ukraine_EBA_Yamna	GlobularAmphora	TTK	-0.010686	-5.2
Ukraine_EBA_Yamna	TTK	YUN_CA	-0.010973	-5.7
Ukraine_EBA_Yamna	TTK	Trypillia	-0.010950	-5.8
Ukraine_EBA_Yamna	Russia_Karelia	YUN_CA	-0.007809	-6.8
Ukraine_EBA_Yamna	Armenia_Aknashen_N	Russia_Karelia	-0.011213	-7.6
Ukraine_EBA_Yamna	Maikop	Russia_Karelia	-0.009610	-8.1
Ukraine_N	Lebyazhinka_HG	Serbia_IronGates_Mesolithic	-0.005269	-6.4
Ukraine_N	Remontnoye	Serbia_IronGates_Mesolithic	-0.004091	-6.7
Ukraine_N	CoreYamna	Serbia_IronGates_Mesolithic	-0.003057	-7.6
Ukraine_N	PVgroup	Serbia_IronGates_Mesolithic	-0.005488	-9.1
Ukraine_N	BPgroup	Serbia_IronGates_Mesolithic	-0.006053	-11.8
Ukraine_N	Serbia_IronGates_Mesolithic	TTK	-0.014370	-15.1
Ukraine_N	Russia_Karelia	Serbia_IronGates_Mesolithic	-0.007871	-17.2
Ukraine_Vasilevka	Serbia_IronGates_Mesolithic	TTK	-0.005716	-3.0
Usatove	Maikop	Serbia_IronGates_Mesolithic	-0.003147	-3.2
Usatove	CoreYamna	Trypillia	-0.002533	-3.2
Usatove	Armenia_Aknashen_N	Ukraine_N	-0.005042	-3.9
Usatove	Ukraine_N	YUN_CA	-0.003424	-3.9
Usatove	GK2	YUN_CA	-0.004916	-4.0
Usatove	Armenia_Aknashen_N	Serbia_IronGates_Mesolithic	-0.005460	-4.1
Usatove	Armenia_Aknashen_N	Russia_Karelia	-0.005776	-4.1
Usatove	Russia_Karelia	Trypillia	-0.003697	-4.2
Usatove	PVgroup	Trypillia	-0.004189	-4.3
Usatove	GlobularAmphora	TTK	-0.007527	-4.4

Usatove	TTK	Trypillia	-0.007953	-5.1
Usatove	PVgroup	YUN_CA	-0.005918	-5.9
Usatove	TTK	YUN_CA	-0.009666	-6.0
Usatove	CoreYamna	YUN_CA	-0.004786	-6.1
Usatove	BPgroup	GlobularAmphora	-0.006454	-6.8
Usatove	Lebyazhinka_HG	YUN_CA	-0.009264	-7.1
Usatove	BPgroup	Trypillia	-0.006422	-7.4
Usatove	BPgroup	YUN_CA	-0.008935	-10.1
Usatove	Russia_Karelia	YUN_CA	-0.008941	-10.1
USV	Lebyazhinka_HG	Trypillia	-0.004575	-3.1
USV	Serbia_IronGates_Mesolithic	YUN_CA	-0.003328	-3.3
USV	Remontnoye	Serbia_IronGates_Mesolithic	-0.003991	-3.3
USV	Remontnoye	YUN_CA	-0.003944	-3.7
USV	CoreYamna	Trypillia	-0.003226	-3.8
USV	GlobularAmphora	Russia_Karelia	-0.004343	-3.9
USV	Armenia_Aknashen_N	Lebyazhinka_HG	-0.010196	-4.2
USV	CoreYamna	GlobularAmphora	-0.003979	-4.3
USV	GlobularAmphora	TTK	-0.009137	-4.7
USV	Armenia_Aknashen_N	GK2	-0.009620	-4.8
USV	Maikop	Russia_Karelia	-0.005972	-5.2
USV	Maikop	Ukraine_N	-0.005614	-5.2
USV	Russia_Karelia	Trypillia	-0.005670	-5.7
USV	PVgroup	Trypillia	-0.006213	-5.8
USV	TTK	Trypillia	-0.009831	-5.8
USV	GlobularAmphora	PVgroup	-0.007390	-6.1
USV	Maikop	Serbia_IronGates_Mesolithic	-0.007194	-6.5
USV	GK2	YUN_CA	-0.008862	-7.0
USV	Armenia_Aknashen_N	Serbia_IronGates_Mesolithic	-0.010486	-7.2
USV	Ukraine_N	YUN_CA	-0.007081	-7.4
USV	Armenia_Aknashen_N	Russia_Karelia	-0.010918	-7.4
USV	BPgroup	Trypillia	-0.006681	-7.4
USV	CoreYamna	YUN_CA	-0.006285	-7.5
USV	TTK	YUN_CA	-0.013112	-7.6
USV	Lebyazhinka_HG	YUN_CA	-0.011617	-7.7
USV	PVgroup	YUN_CA	-0.008638	-7.9
USV	Armenia_Aknashen_N	Ukraine_N	-0.010691	-7.9
USV	BPgroup	GlobularAmphora	-0.008020	-7.9
USV	BPgroup	YUN_CA	-0.009909	-11.0
USV	Russia_Karelia	YUN_CA	-0.011918	-12.0
VAR_CA	Lebyazhinka_HG	YUN_CA	-0.002115	-3.1
VAR_CA	GK2	YUN_CA	-0.002488	-4.2
VAR_CA	Russia_Karelia	YUN_CA	-0.002851	-6.3
VAR_CA	Ukraine_N	YUN_CA	-0.003039	-7.7
VAR_CA	Serbia_IronGates_Mesolithic	YUN_CA	-0.003861	-9.3

We next proceed to model this admixture.

Table SI2. 4. Populations that can be modeled as 2-way mixtures clades (*N*=2) of the sources.

Test	A	B	P-value	A	B	S.E.
Bulgaria_C_ProtoYamna	CoreYamna	GlobularAmphora	0.099	45.3%	54.7%	2.7%
Bulgaria_EBA_Yamna	Armenia_Aknashen_N	Lebyazhinka_HG	0.082	55.9%	44.1%	1.7%
Bulgaria_EBA_Yamna	BPgroup	GlobularAmphora	0.812	70.8%	29.2%	1.5%
Bulgaria_EBA_Yamna	BPgroup	Trypillia	0.246	70.4%	29.6%	1.5%
Bulgaria_EBA_Yamna	CoreYamna	GlobularAmphora	0.310	83.3%	16.7%	1.6%
Bulgaria_EBA_Yamna	CoreYamna	Trypillia	0.728	82.8%	17.2%	1.6%
Bulgaria_EBA_Yamna	CoreYamna	YUN_CA	0.736	85.7%	14.3%	1.4%
Bulgaria_EBA_Yamna	GlobularAmphora	PVgroup	0.458	26.2%	73.8%	1.9%
Bulgaria_EBA_Yamna	Lebyazhinka_HG	Maykop	0.189	38.5%	61.5%	1.7%
Bulgaria_Riltsi_EBA_Yamna	Armenia_Aknashen_N	GlobularAmphora	0.361	61.4%	38.6%	6.2%
Bulgaria_Riltsi_EBA_Yamna	Armenia_Aknashen_N	Trypillia	0.212	56.4%	43.6%	7.0%
Bulgaria_Riltsi_EBA_Yamna	BPgroup	YUN_CA	0.124	38.2%	61.8%	3.3%
Bulgaria_Riltsi_EBA_Yamna	CoreYamna	YUN_CA	0.058	42.9%	57.1%	3.7%

Bulgaria_Riltsi_EBA_Yamna	GlobularAmphora	Maykop	0.354	38.8%	61.2%	6.1%
Bulgaria_Riltsi_EBA_Yamna	Maykop	Trypillia	0.538	55.8%	44.2%	6.3%
Bulgaria_Riltsi_EBA_Yamna	Maykop	YUN_CA	0.103	63.7%	36.3%	5.4%
Bulgaria_Riltsi_EBA_Yamna	PVgroup	YUN_CA	0.335	40.4%	59.6%	3.4%
Bulgaria_Riltsi_EBA_Yamna	Remontnoye	Trypillia	0.075	42.9%	57.1%	5.3%
Bulgaria_Riltsi_EBA_Yamna	Remontnoye	YUN_CA	0.558	50.3%	49.7%	4.5%
Moldova_EBA_Yamna	BPgroup	GlobularAmphora	0.300	78.9%	21.1%	1.1%
Moldova_EBA_Yamna	CoreYamna	GlobularAmphora	0.511	93.0%	7.0%	1.0%
Moldova_EBA_Yamna	CoreYamna	Trypillia	0.545	92.8%	7.2%	1.0%
Moldova_EBA_Yamna	CoreYamna	YUN_CA	0.361	94.0%	6.0%	0.9%
Moldova_EBA_Yamna	GK2	Remontnoye	0.672	29.6%	70.4%	2.1%
Moldova_EBA_Yamna_o	BPgroup	GlobularAmphora	0.473	53.0%	47.0%	2.4%
Moldova_EBA_Yamna_o	BPgroup	Trypillia	0.321	52.1%	47.9%	2.4%
Moldova_EBA_Yamna_o	CoreYamna	GlobularAmphora	0.265	62.4%	37.6%	2.7%
Moldova_EBA_Yamna_o	CoreYamna	Trypillia	0.523	61.3%	38.7%	2.8%
Moldova_EBA_Yamna_o	CoreYamna	YUN_CA	0.191	67.9%	32.1%	2.4%
Moldova_EBA_Yamna_o	GlobularAmphora	PVgroup	0.831	44.7%	55.3%	2.7%
Moldova_EBA_Yamna_o	PVgroup	Trypillia	0.136	54.5%	45.5%	2.8%
Moldova_GlobularAmphora_Yamna	BPgroup	GlobularAmphora	0.052	74.5%	25.5%	2.2%
Moldova_GlobularAmphora_Yamna	CoreYamna	GlobularAmphora	0.180	88.0%	12.0%	2.5%
Moldova_GlobularAmphora_Yamna	CoreYamna	Trypillia	0.057	88.6%	11.4%	2.6%
Moldova_GlobularAmphora_Yamna	GK2	Remontnoye	0.395	33.3%	66.7%	3.7%
Moldova_GlobularAmphora_Yamna	Lebyazhinka_HG	Maykop	0.091	44.1%	55.9%	2.5%
Moldova_GlobularAmphora_Yamna	Remontnoye	Ukraine_N	0.388	69.1%	30.9%	3.1%
SShi	CoreYamna	GK2	0.134	83.7%	16.3%	3.0%
SShi	CoreYamna	Lebyazhinka_HG	0.075	85.8%	14.2%	2.6%
SShi	CoreYamna	Russia_Karelia	0.058	87.5%	12.5%	2.3%
SShi	GK2	PVgroup	0.088	30.3%	69.7%	3.2%
SShi	PVgroup	Ukraine_N	0.135	74.0%	26.0%	2.5%
SSlo	Armenia_Aknashen_N	GK2	0.204	15.4%	84.6%	3.8%
SSlo	BPgroup	Serbia_IronGates_Mesolithic	0.063	59.5%	40.5%	3.4%
SSlo	BPgroup	Ukraine_N	0.885	33.9%	66.1%	5.2%
SSlo	CoreYamna	GK2	0.142	31.4%	68.6%	7.5%
SSlo	CoreYamna	Ukraine_N	0.750	37.8%	62.2%	5.7%
SSlo	GK2	Maykop	0.283	83.2%	16.8%	4.0%
SSlo	GK2	PVgroup	0.079	76.1%	23.9%	6.7%
SSlo	GK2	Remontnoye	0.314	78.1%	21.9%	5.3%
SSlo	PVgroup	Ukraine_N	0.906	31.4%	68.6%	5.1%
SSlo	Remontnoye	Ukraine_N	0.264	25.6%	74.4%	4.4%
SSmed	CoreYamna	GK2	0.520	60.6%	39.4%	3.2%
SSmed	CoreYamna	Ukraine_N	0.309	65.5%	34.5%	2.3%
SSmed	GK2	Remontnoye	0.740	58.0%	42.0%	2.6%
Ukraine_EBA_Yamna	BPgroup	GlobularAmphora	0.265	77.7%	22.3%	1.5%
Ukraine_EBA_Yamna	CoreYamna	GlobularAmphora	0.853	91.9%	8.1%	1.6%
Ukraine_EBA_Yamna	CoreYamna	Trypillia	0.697	91.9%	8.1%	1.6%
Ukraine_EBA_Yamna	CoreYamna	YUN_CA	0.295	93.6%	6.4%	1.4%
Ukraine_EBA_Yamna	GK2	Remontnoye	0.781	30.9%	69.1%	2.6%
Ukraine_MBA_Catacomb_o1	Armenia_Aknashen_N	CoreYamna	0.083	42.4%	57.6%	9.3%
Ukraine_MBA_Catacomb_o1	Armenia_Aknashen_N	GK2	0.301	63.5%	36.5%	6.0%
Ukraine_MBA_Catacomb_o1	Armenia_Aknashen_N	Lebyazhinka_HG	0.272	66.8%	33.2%	5.2%
Ukraine_MBA_Catacomb_o1	Armenia_Aknashen_N	Russia_Karelia	0.188	69.7%	30.3%	4.9%
Ukraine_MBA_Catacomb_o1	Armenia_Aknashen_N	Ukraine_N	0.145	65.5%	34.5%	5.6%
Ukraine_MBA_Catacomb_o1	BPgroup	GlobularAmphora	0.253	59.3%	40.7%	5.3%
Ukraine_MBA_Catacomb_o1	BPgroup	Trypillia	0.410	56.4%	43.6%	5.3%
Ukraine_MBA_Catacomb_o1	BPgroup	YUN_CA	0.560	62.8%	37.2%	4.5%
Ukraine_MBA_Catacomb_o1	CoreYamna	GlobularAmphora	0.162	69.6%	30.4%	6.2%
Ukraine_MBA_Catacomb_o1	CoreYamna	Trypillia	0.264	66.2%	33.8%	6.4%
Ukraine_MBA_Catacomb_o1	CoreYamna	YUN_CA	0.458	71.7%	28.3%	5.1%
Ukraine_MBA_Catacomb_o1	GK2	Maykop	0.227	30.8%	69.2%	6.1%
Ukraine_MBA_Catacomb_o1	GlobularAmphora	PVgroup	0.551	35.6%	64.4%	5.3%
Ukraine_MBA_Catacomb_o1	GlobularAmphora	Remontnoye	0.507	23.1%	76.9%	6.5%
Ukraine_MBA_Catacomb_o1	Lebyazhinka_HG	Maykop	0.226	27.5%	72.5%	5.4%
Ukraine_MBA_Catacomb_o1	Maykop	Russia_Karelia	0.171	75.1%	24.9%	5.0%
Ukraine_MBA_Catacomb_o1	Maykop	Ukraine_N	0.130	71.2%	28.8%	5.6%
Ukraine_MBA_Catacomb_o1	PVgroup	Trypillia	0.531	61.8%	38.2%	5.9%
Ukraine_MBA_Catacomb_o1	PVgroup	YUN_CA	0.454	68.0%	32.0%	4.9%

Ukraine_MBA_Catacomb_o1	Remontnoye	Trypillia	0.354	75.1%	24.9%	7.1%
Ukraine_MBA_Catacomb_o1	Remontnoye	YUN_CA	0.186	81.6%	18.4%	6.0%
Ukraine_N_I27992	Armenia_Aknashen_N	GK2	0.313	29.9%	70.1%	8.2%
Ukraine_N_I27992	Armenia_Aknashen_N	Ukraine_N	0.238	30.8%	69.2%	7.5%
Ukraine_N_I27992	CoreYamna	Serbia_IronGates_Mesolithic	0.073	66.5%	33.5%	8.5%
Ukraine_N_I27992	GK2	GlobularAmphora	0.409	67.0%	33.0%	8.1%
Ukraine_N_I27992	GK2	Maykop	0.366	68.9%	31.1%	8.9%
Ukraine_N_I27992	GK2	Trypillia	0.535	69.0%	31.0%	7.8%
Ukraine_N_I27992	GK2	YUN_CA	0.640	74.4%	25.6%	6.7%
Ukraine_N_I27992	GlobularAmphora	Lebyazhinka_HG	0.454	40.1%	59.9%	6.9%
Ukraine_N_I27992	GlobularAmphora	Russia_Karelia	0.243	46.3%	53.7%	6.6%
Ukraine_N_I27992	GlobularAmphora	TTK	0.107	53.3%	46.7%	5.8%
Ukraine_N_I27992	Lebyazhinka_HG	Trypillia	0.356	62.3%	37.7%	6.6%
Ukraine_N_I27992	Lebyazhinka_HG	YUN_CA	0.292	68.7%	31.3%	5.9%
Ukraine_N_I27992	Maykop	Ukraine_N	0.279	32.5%	67.5%	8.2%
Ukraine_N_I27992	Remontnoye	Serbia_IronGates_Mesolithic	0.084	57.0%	43.0%	7.4%
Ukraine_N_I27992	Remontnoye	Ukraine_N	0.262	38.5%	61.5%	9.6%
Ukraine_N_I27992	Russia_Karelia	Trypillia	0.171	56.0%	44.0%	6.2%
Ukraine_N_I27992	Russia_Karelia	YUN_CA	0.114	63.0%	37.0%	5.3%
Ukraine_N_I27992	Trypillia	Ukraine_N	0.111	29.3%	70.7%	7.8%
Ukraine_N_I27992	Ukraine_N	YUN_CA	0.155	75.8%	24.2%	6.6%
Usatove	PVgroup	Trypillia	0.128	45.4%	54.6%	1.5%
Usatove_I20078	BPgroup	TTK	0.091	79.1%	20.9%	4.5%
Usatove_I20078	CoreYamna	TTK	0.606	65.2%	34.8%	3.6%
Usatove_I20078	PVgroup	TTK	0.072	72.2%	27.8%	4.4%
Zhivotilovka_I17974	CoreYamna	Lebyazhinka_HG	0.077	92.1%	7.9%	4.0%
Zhivotilovka_I17974	CoreYamna	TTK	0.616	89.0%	11.0%	3.4%
Zhivotilovka_I17974	GK2	PVgroup	0.597	18.7%	81.3%	4.2%
Zhivotilovka_I17974	Lebyazhinka_HG	PVgroup	0.161	17.3%	82.7%	4.5%
Zhivotilovka_I17974	PVgroup	Russia_Karelia	0.135	85.1%	14.9%	3.8%
Zhivotilovka_I17974	PVgroup	Serbia_IronGates_Mesolithic	0.083	91.6%	8.4%	2.4%
Zhivotilovka_I17974	PVgroup	Ukraine_N	0.401	84.5%	15.5%	3.6%

Populations that can be modeled as mixtures of two of the sources (*N*=2)

We next examined populations for which there are feasible models of 2-way admixture (Table SI2. 4). We define feasible models as having p>0.05, standard errors ≤10%, and admixture proportions within two standard errors of the endpoints of the [0, 1] interval. Alternative models exist for many of these populations. We did not discuss further the Serednii Stih subsets (SShi, SSmed, SSlo) here, except to note that they are not modeled with European farmer populations as sources (YUN_CA or Trypillia) but with sources that are included in the analysis of the linked study ref.[3] to which we refer the reader. Here we can summarize that the Serednii Stih can be modeled with Core Yamna (the end point of the Dnipro cline) and Dnipro-Don hunter-gatherers (Ukraine_N or GK2), or with populations of the CLV cline (PVgroup, BPgroup, Remontnoye, or Maykop) and on the whole can be seen as the result of fusion of CLV cline migrants with local Dnipro-Don foragers that is generative of the Dnipro cline.

We use the model tournament approach of all-against-all to see if we can distinguish between some of the alternative models.

Bulgaria_C_ProtoYamna (3500-3000 BCE)

For this individual (I1456 / 3500-3000 BCE) from Durankulak only a single 2-way feasible model exists that involves a fairly even mix of Core Yamna (~45%) and Globular Amphora (~55%) ancestries (Table SI2. 4). Models with Trypillian (p=1e-5) or YUN_CA (p=3e-13) alternative sources of European farmer ancestry both fail.

It has been postulated that the Globular Amphora was the source of farmer ancestry in the Corded Ware population of central-northern Europe on the basis of the fact that this European farmer population shares IBD segments with the Corded Ware.[30] The proportion of farmer ancestry in the Corded Ware and diverse derived populations is fairly even[31] and amounts to about ~1/4 as estimated in ref.[2] Thus, the Corded

Ware represents a mixture of similar components as the Bulgarian "Proto-Yamna" individual, albeit in different proportions.

We applied the Core Yamna + Globular Amphora to Corded Ware individuals from Germany and obtained an estimate of 26.6±1.3% (p=0.034). For both the Proto-Yamna individual and the Corded Ware individual, we noticed that shared genetic drift with the Villabruna hunter-gatherer (from the Base set of outgroups) is underestimated (Z<-2.5), suggesting that there is an excess of hunter-gatherer ancestry (above and beyond what was part of the Globular Amphora population sample we have) in both. Adding Serbia Iron Gates hunter-gatherers as a 3rd source results in a successful fit in both Proto-Yamna (p=0.328) and Corded Ware (p=0.156) with a significant proportion of such ancestry (6.1±2.6% and 2.9±1.2% respectively). We also added Latvia_HG[16] from the Baltic region as the 3rd source inspired by the model of ref.[32] which includes an amount of northeastern European ancestry in the Corded Ware. This model also fits for both the Proto-Yamna (6.2±2.9%; p=0.262) and Corded Ware (3.2±1.3%; p=0.135) and thus the source of the Villabruna-related ancestry in the Corded Ware and Proto-Yamna cannot be well-determined geographically. As the territory of the Globular Amphora is located in-between the Balkans and the Baltic, we think it likely that the Corded Ware was formed by admixture with a local Globular Amphora-related population that had a slight excess of hunter-gatherer ancestry rather than an extra pulse of admixture from either the Balkans or the Baltic.

We were motivated to pursue the Corded Ware connection by the additional observation that the Bulgarian Proto-Yamna individual belonged to Y-haplogroup R-M417.[33] This lineage is typical of the Corded Ware populations[2,14,31,32] We offer the hypothesis that the Proto-Yamna individual is connected to the formation of the Corded Ware population as its autosomal ancestry points to the same sources (Core Yamna and Globular Amphora with a slight excess of hunter-gatherer ancestry) and was part of the same Y-chromosome lineage whose origin prior to the appearance of the Corded Ware is unclear.

Possibly, both the Proto-Yamna individual and the nascent Corded Ware were derived from a zone of interaction between Yamna-related and Globular Amphora-related populations which must have generated a cline of variable ancestry between the two sources: the Corded Ware emerged as an expansive archaeological culture in which ~1/4 of the Globular Amphora ancestry was incorporated while the Bulgarian Proto-Yamna individual had even more Globular Amphora ancestry.

As a final test, we fit the Proto-Yamna individual as Corded Ware + Globular Amphora (p=0.438) with an estimated 37.4±3.7% Globular Amphora. Given that Corded Ware itself has 26.6±1.3% of Globular Amphora ancestry, the total amounts to (1-0.374)*0.266+0.374≈54%, close to the directly estimated 54.7±2.7% Globular Amphora ancestry (Table SI2. 4) from the Core Yamna + Globular Amphora model.

Bulgaria_EBA_Yamna (3300-2000 BCE)

For the Yamna from Bulgaria, we see that the only models that are feasible in model competition are the ones that involve Core Yamna as one source and a European farmer population as the other (Table SI2. 5). Models without Core Yamna as a source are feasible against the Base set of outgroups (Table SI2. 4) but are not resilient to the addition of Core Yamna to the set of outgroups. Model competition does not allow us to decide between the different European farmer populations, but the models agree in deriving a similar proportion of such ancestry (~14-17%) from the farmer source. It is thus not clear whether the farmer ancestry was of local origin in Bulgaria or was acquired north of Bulgaria (where Trypillians and Globular Amphora-related populations lived).

Table SI2. 5. Model competition for Bulgaria_EBA_Yamna. Each column shows the p-value of a model when alternative models (rows) are placed on the Right set.

	BPgroup+GlobularAmphora	BPgroup+Trypillia	CoreYamna+GlobularAmphora	CoreYamna+Trypillia	CoreYamna+YUN_CA	Armenia_Aknashen_N+Lebyazhinka_HG	GlobularAmphora+PVgroup	Lebyazhinka_HG+Maykop
BPgroup+GlobularAmphora		3.18E-01	4.40E-01	8.22E-01	8.49E-01	4.05E-05	3.23E-03	1.93E-07
BPgroup+Trypillia	8.97E-01		1.58E-01	8.22E-01	5.79E-01	2.68E-05	4.06E-03	5.85E-08
CoreYamna+GlobularAmphora	8.60E-16	2.32E-15		7.19E-01	7.94E-01	1.47E-31	1.05E-15	3.87E-38
CoreYamna+Trypillia	3.71E-15	5.91E-16	1.17E-01		5.63E-01	1.41E-34	3.54E-15	8.08E-38
CoreYamna+YUN_CA	2.79E-15	2.51E-15	4.50E-01	8.31E-01		4.55E-31	4.13E-15	4.68E-37
Armenia_Aknashen_N+Lebyazhinka_HG	2.60E-01	1.51E-02	1.26E-01	1.72E-01	1.37E-01		2.88E-02	1.57E-01
GlobularAmphora+PVgroup	7.03E-02	1.91E-02	2.61E-01	6.24E-01	7.94E-01	7.30E-05		2.11E-04
Lebyazhinka_HG+Maykop	4.82E-01	8.42E-02	4.48E-01	7.65E-01	6.16E-01	8.09E-02	6.07E-02	

We fit the Core Yamna+YUN_CA model to individuals within the Bulgarian Yamna group (Table SI2. 6). We see that this group included both individuals that were unadmixed Core Yamna as well as others with a significant amount of farmer ancestry. Thus, while we cannot be certain that the admixture between European farmers and Yamna occurred locally in Bulgaria, there were certainly core Yamna individuals present there, so the opportunity for such local admixture existed. These results are consistent with the penetration of southeastern Europe by Yamna well beyond the geographical transition zone of the steppe by Yamna individuals and their admixture with local farmers.[12]

Table SI2. 6. Admixture of Bulgarian Yamna individuals.

Test	P-value	CoreYamna	YUN_CA	S.E.
I1427	0.436	100.9%	-0.9%	2.8%
I1448	0.639	77.7%	22.3%	3.1%
I18793	0.889	89.0%	11.0%	2.2%
I18794	0.696	78.3%	21.7%	2.6%
I18801_d	0.628	99.0%	1.0%	3.5%

Bulgaria_Riltsi_EBA_Yamna (3300-2500 BCE)

Multiple models apply to the Riltsi Yamna (Table SI2. 4) which is analyzed separately from the other Yamna from Bulgaria. It is clear that in model competition, models with core Yamna as a source do not fit best (Table SI2. 7), but rather two models that include local (YUN_CA) ancestry and ancestry from the CLV Cline (either PVgroup or Remontnoye) fit better. These models only lose a match to another model of Maykop+Trypillian ancestry. Since Maykop is itself part of the CLV Cline, we can conclude that the Riltsi Yamna individual (I1428; 3300-2500 BCE) are descended from CLV cline populations that admixed with European farmers and not from the Core Yamna as the other Yamna individuals from southeastern Europe.

Table SI2. 7. Model competition for Bulgaria_Riltsi_EBA_Yamna. Each column shows the p-value of a model when alternative models (rows) are placed on the Right set.

	Armenia_Aknashen_N+Trypillia	BPgroup+YUN_CA	CoreYamna+YUN_CA	Armenia_Aknashen_N+GlobularAmphora	GlobularAmphora+Maykop	Maykop+Trypillia	Maykop+YUN_CA	PVgroup+YUN_CA	Remontnoye+Trypillia	Remontnoye+YUN_CA
Armenia_Aknashen_N+Trypillia		4.46E-02	3.61E-02	9.91E-02	1.11E-02	7.01E-01	3.81E-04	6.25E-02	1.52E-01	1.24E-01
BPgroup+YUN_CA	6.26E-03		7.10E-02	1.51E-02	3.23E-02	4.89E-02	1.92E-03	4.21E-01	1.66E-01	5.26E-01
CoreYamna+YUN_CA	3.34E-06	1.13E-01		6.63E-06	3.98E-04	6.64E-04	8.09E-06	2.33E-01	9.77E-02	2.90E-01
Armenia_Aknashen_N+GlobularAmphora	3.16E-01	2.96E-01	1.64E-01		4.62E-01	8.10E-01	2.27E-01	6.10E-01	2.19E-01	8.16E-01
GlobularAmphora+Maykop	5.57E-02	2.60E-02	1.60E-02	9.31E-02		6.84E-01	1.55E-01	1.20E-01	2.21E-02	2.65E-01
Maykop+Trypillia	4.03E-02	5.66E-03	5.59E-03	3.79E-02	4.79E-03		1.19E-04	1.53E-02	1.60E-02	4.14E-02
Maykop+YUN_CA	6.50E-02	1.51E-02	9.08E-03	1.57E-01	3.85E-01	6.29E-01		8.37E-02	3.18E-02	2.10E-01
PVgroup+YUN_CA	9.23E-02	2.09E-01	1.05E-01	2.11E-01	3.21E-01	4.29E-01	5.61E-02		2.16E-01	6.93E-01
Remontnoye+Trypillia	3.40E-01	4.53E-02	3.65E-02	2.01E-01	1.20E-02	6.87E-01	4.48E-04	6.06E-02		6.97E-02
Remontnoye+YUN_CA	4.15E-01	1.83E-01	9.09E-02	6.40E-01	5.30E-01	7.66E-01	1.80E-01	4.37E-01	1.32E-01	

Moldova_EBA_Yamna (3400-2500BCE)

The model tournament for the Yamna of Moldova (Table SI2. 8) shows that it is the models with Core Yamna and European farmer (Trypillian or YUN_CA) ancestry that fare best.

Table SI2. 8. Model competition for Moldova_EBA_Yamna. Each column shows the p-value of a model when alternative models (rows) are placed on the Right set.

	BPgroup+GlobularAmphora	CoreYamna+GlobularAmphora	CoreYamna+Trypillia	CoreYamna+YUN_CA	GK2+Remontnoye
BPgroup+GlobularAmphora		6.55E-01	7.40E-01	5.77E-01	4.25E-02
CoreYamna+GlobularAmphora	2.50E-29		6.24E-01	4.61E-01	6.29E-52
CoreYamna+Trypillia	1.99E-29	5.47E-05		1.79E-04	1.98E-53
CoreYamna+YUN_CA	8.04E-29	7.03E-03	4.15E-03		3.02E-46
GK2+Remontnoye	3.80E-01	7.70E-01	8.41E-01	6.90E-01	

When we look at individuals (Table SI2. 9) we see that they have inherited different amounts of European farmer ancestry. Four individuals are included in our Core Yamna definition so by definition we do not provide estimates of European farmer ancestry in them (10208, I10398, I12637, I7847). in addition, some of the other modeled Moldovan Yamna individuals are consistent with having no European farmer ancestry. Thus, Moldovan Yamna (like those from Bulgaria) included both unadmixed members of the core group as well as those that had acquired some European farmer ancestry.

Table SI2. 9. Admixture proportions for Moldova_EBA_Yamna. Individuals 10208, I10398, I12637, I7847 are part of the Core Yamna group and used as a source here.

Test	P-value	CoreYamna	YUN_CA	S.E.
I10174	0.222	96.0%	4.0%	4.1%
I10206	0.600	83.7%	16.3%	2.9%
I10414	0.314	95.2%	4.8%	2.1%
I10415	0.605	97.4%	2.6%	4.6%
I10424	0.184	96.6%	3.4%	2.1%
I12507	0.358	95.5%	4.5%	2.2%
I12512	0.242	85.8%	14.2%	4.2%
I17743	0.593	93.1%	6.9%	2.4%

I17744	0.410	95.5%	4.5%	2.4%
I17745	0.228	88.9%	11.1%	2.5%
I20068	0.185	96.7%	3.3%	2.4%
I7848	0.015	93.0%	7.0%	2.1%

Moldova_EBA_Yamna_o (2865-2576 calBCE)

We analyze this outlier individual (I17747; 2865-2576 calBCE) separately as it has much farmer ancestry (Table SI2. 4). The results of the model tournament (Table SI2. 10) show that models with Core Yamna and European farmer (Trypillian or Globular Amphora) ancestry work best for this individual.

Table SI2. 10. Model competition for Moldova_EBA_Yamna_o. Each column shows the p-value of a model when alternative models (rows) are placed on the Right set.

	BPgroup+GlobularAmphora	BPgroup+Trypillia	CoreYamna+GlobularAmphora	CoreYamna+Trypillia	CoreYamna+YUN_CA	GlobularAmphora+PVgroup	PVgroup+Trypillia
BPgroup+GlobularAmphora		1.62E-02	2.34E-01	1.17E-01	1.12E-02	7.14E-01	7.89E-03
BPgroup+Trypillia	6.13E-01		2.09E-01	5.32E-01	3.26E-01	7.86E-01	8.36E-02
CoreYamna+GlobularAmphora	3.20E-05	9.63E-06		1.14E-01	7.78E-03	1.25E-05	1.06E-06
CoreYamna+Trypillia	4.12E-05	7.68E-05	2.88E-01		2.71E-01	2.47E-05	3.49E-06
CoreYamna+YUN_CA	8.82E-05	1.59E-04	3.33E-01	4.92E-01		3.06E-05	6.53E-06
GlobularAmphora+PVgroup	4.47E-01	1.77E-02	2.24E-01	1.34E-01	1.52E-02		1.61E-02
PVgroup+Trypillia	5.75E-01	2.97E-01	1.95E-01	5.06E-01	3.29E-01	9.01E-01	

Moldova_GlobularAmphora_Yamna (2906-2702 calBCE)

This individual (I20076; 2906-2702 calBCE) is also analyzed separately. In the model competition (Table SI2. 11) it is also inferred to be a mixture of core Yamna and European farmers (Trypillians or Globular Amphora).

Table SI2. 11. Model competition for Moldova_GlobularAmphora_Yamna. Each column shows the p-value of a model when alternative models (rows) are placed on the Right set.

	BPgroup+GlobularAmphora	CoreYamna+GlobularAmphora	CoreYamna+Trypillia	GK2+Remontnoye	Lebyazhinka_HG+Maykop	Remontnoye+Ukraine_N
BPgroup+GlobularAmphora		1.59E-01	5.63E-02	4.67E-01	3.04E-03	4.05E-01
CoreYamna+GlobularAmphora	1.13E-13		9.07E-02	1.31E-17	3.07E-28	1.06E-25
CoreYamna+Trypillia	1.85E-13	1.06E-01		9.22E-19	1.76E-29	4.99E-28
GK2+Remontnoye	1.56E-01	3.88E-01	1.70E-01		8.23E-03	4.49E-01
Lebyazhinka_HG+Maykop	1.05E-01	3.09E-01	1.27E-01	5.90E-01		4.45E-01
Remontnoye+Ukraine_N	9.46E-03	2.28E-01	7.33E-02	3.40E-01	3.63E-03	

Ukraine_EBA_Yamna (3300-2500 BCE)

For the Ukraine Yamna we see that the Remontnoye+GK2 and BPgroup+Globular Amphora models fail in competition that include Core Yamna as a source (Table SI2. 12). The other models remain viable in competition to each other.

Table SI2. 12. Model competition for Ukraine_EBA_Yamna. Each column shows the p-value of a model when alternative models (rows) are placed on the Right set.

	BPgroup+GlobularAmphora	CoreYamna+GlobularAmphora	CoreYamna+Trypillia	CoreYamna+YUN_CA	GK2+Remontnoye
BPgroup+GlobularAmphora		4.32E-01	2.90E-01	1.28E-01	4.30E-02
CoreYamna+GlobularAmphora	4.61E-27		5.24E-01	1.81E-01	5.25E-41

CoreYamna+Trypillia	3.13E-27	7.59E-01		2.34E-01	4.28E-39
CoreYamna+YUN_CA	4.13E-27	8.65E-01	7.53E-01		8.51E-39
GK2+Remontnoye	4.11E-01	8.13E-01	7.45E-01	3.97E-01	

We must note here that four Yamna individuals are included in the "Core Yamna" group definition: I12168, I20975, I2105, I3141_enhanced. Thus, in Ukraine too there were indeed representatives of the Core Yamna group without evidence of any farmer admixture, as well as individuals as those included under the Ukraine_EBA_Yamna genetic grouping (I12619, I12843, I11999, I12617) with some farmer admixture. Thus, the Yamna of Ukraine (in whose territory co-existed herder and settled farmer populations) was indeed home to Yamna of the core group (whose ancestry was from the Serednii Stih and consisted of the mix of eastern CLV migrants and Ukraine_N-related foragers) as well as Yamna who had acquired a fraction of European farmer ancestry. We fit the same model for the Ukraine_EBA_Yamna individuals that we did for Bulgarian ones (Table SI2. 6) and show the results in Table SI2. 13 (We also fit the model with the geographically more proximal Trypillia as a source). As in Bulgaria the Yamna of Ukraine included individuals of both the core group and those admixed with European farmers; the Yamna advance must have been rapid enough to ensure that at least individuals from the core group reached Bulgaria without any admixture from the farmer groups in the intervening space, but clearly some Yamna in both Ukraine (closer to the unknown origin of the core Yamna group) and Bulgaria had acquired some European farmer ancestry.

Table SI2. 13. Admixture of Ukrainian Yamna individuals. Individuals included in the Core Yamna group (I12168, I20975, I2105, I3141_enhanced) used as a source are not shown.

Test	P-value	CoreYamna	YUN_CA	S.E.
I11999	0.026	95.9%	4.1%	2.1%
I12617	0.270	93.4%	6.6%	3.8%
I12619	0.207	92.6%	7.4%	2.3%
I12843	0.090	91.8%	8.2%	2.1%
**Test**	**P-value**	**CoreYamna**	**Trypillia**	**S.E.**
I11999	0.062	93.9%	6.1%	2.6%
I12617	0.188	93.6%	6.4%	4.7%
I12619	0.443	90.3%	9.7%	2.6%
I12843	0.038	91.1%	8.9%	2.6%

Ukraine_MBA_Catacomb_o1 (2201-2032 calBCE)

This individual (I13071; 2201-2032 calBCE) fits multiple models (Table SI2. 4) and cannot be well resolved using the model tournament approach. In terms of the models with Core Yamna and European farmer ancestry that seem to describe well Bronze Age populations from the NPR, it seems to have ~1/3 of farmer ancestry.

Ukraine_MBA_MultiCordonedWare_Babine (2400-1900BCE)

There are no feasible 2-source models for the Multi-Cordoned Ware/Babine population (Table SI2. 4)

We also note that for Multi-Cordoned Ware the model with Trypillian as a source (that worked for the Ukraine_EBA_Yamna) fails (p=4e-5). This model fails as it underestimates (Z=-3.6) shared genetic drift with the Villabruna hunter-gatherer Right population, suggesting that the non-Yamna source is enriched

in hunter-gatherer ancestry. This can be assessed directly by examining 3-way models with both European farmers and Ukraine_N/Serbian Iron Gates as sources (Table SI2. 14) which confirms our intuition.

Table SI2. 14. 3-way models with varying hunter-gatherer ancestry for Bronze Age Ukraine.

					Proportions			Std. errors		
Test	A	B	C	P-value	A	B	C	A	B	C
Ukraine_EBA_Yamna	CoreYamna	GlobularAmphora	Serbia_IronGates_Mesolithic	0.783	91.3%	7.8%	0.9%	1.7%	1.7%	1.5%
Ukraine_EBA_Yamna	CoreYamna	GlobularAmphora	Ukraine_N	0.761	91.3%	8.1%	0.7%	2.3%	1.6%	2.3%
Ukraine_EBA_Yamna	CoreYamna	Serbia_IronGates_Mesolithic	Trypillia	0.840	90.5%	2.0%	7.6%	1.8%	1.4%	1.6%
Ukraine_EBA_Yamna	CoreYamna	Serbia_IronGates_Mesolithic	YUN_CA	0.776	90.9%	3.0%	6.1%	1.9%	1.4%	1.4%
Ukraine_EBA_Yamna	CoreYamna	Trypillia	Ukraine_N	0.740	89.7%	7.9%	2.4%	2.5%	1.6%	2.2%
Ukraine_EBA_Yamna	CoreYamna	Ukraine_N	YUN_CA	0.580	89.5%	4.0%	6.5%	2.6%	2.2%	1.3%
Ukraine_MBA_MultiCordonedWare_Babine	CoreYamna	GlobularAmphora	Serbia_IronGates_Mesolithic	0.026	81.0%	15.1%	4.0%	1.8%	1.8%	1.5%
Ukraine_MBA_MultiCordonedWare_Babine	CoreYamna	GlobularAmphora	Ukraine_N	0.182	76.6%	15.4%	8.1%	2.4%	1.7%	2.2%
Ukraine_MBA_MultiCordonedWare_Babine	CoreYamna	Serbia_IronGates_Mesolithic	Trypillia	0.029	79.3%	6.2%	14.5%	2.0%	1.4%	1.7%
Ukraine_MBA_MultiCordonedWare_Babine	CoreYamna	Serbia_IronGates_Mesolithic	YUN_CA	0.027	80.1%	8.1%	11.8%	1.9%	1.4%	1.4%
Ukraine_MBA_MultiCordonedWare_Babine	CoreYamna	Trypillia	Ukraine_N	0.356	73.3%	15.4%	11.4%	2.6%	1.6%	2.2%
Ukraine_MBA_MultiCordonedWare_Babine	CoreYamna	Ukraine_N	YUN_CA	0.571	72.6%	14.6%	12.9%	2.8%	2.2%	1.4%

Thus, both the Yamna of Ukraine of the EBA and the later Multi-Cordoned Ware of the MBA were largely descended from the Core Yamna but the latter experienced gene flow from a population that seems to have had more hunter-gatherer ancestry.

It was previously observed that Bronze Age populations from neighboring Romania included those that had a high proportion of hunter-gatherer ancestry:[12]

"The first cluster is made up mainly of individuals from Arman (Cârlomănești) who have very low amounts of CHG/EHG ancestry but substantial (~24%) Balkan hunter-gatherer ancestry as does an individual from Târgșoru Vechi (I7152) in the mid-3rd millennium BCE and an individual from Ploieşti Triaj (I10494/ROU_Ploieşti_B) at ~30%."

These individuals, together with those of Ukraine both point to the existence of Chalcolithic populations of high hunter-gatherer ancestry that bequeathed it to those of the Bronze Age. We know of at least one such Chalcolithic individual from Gura Baciului of >50% hunter-gatherer ancestry,[12,34] which provides direct evidence for the existence of such populations during the Chalcolithic.

Ukraine_N_I27992 (5363-5216 calBCE)

We do not draw any conclusions from this Neolithic individual (I27992; 5363-5216 calBCE) as it has a high contamination rate mtcontam=[0.717,0.942]. It is not cladal with Ukrainian Neolithic hunter-gatherers but this would be a consequence of contamination and thus not evidence for early contact with the Ukrainian Neolithic population.

Usatove (4400-3600 BCE)

The Usatove population label includes individuals from Mayaky dated to the late 5th and early 4th millennia BCE. It can only be modeled as a mixture of ~45% PVgroup and ~55% Trypillians (Table SI2. 4). The next best model (however, p=0.002) involves ~56% Remontnoye ancestry and ~44% Trypillian ancestry. The model with BPgroup+Trypillians fails clearly (p<1e-4). We were curious about this as BPgroup and PVgroup are closely related populations within the North Caucasus-Lower Volga Eneolithic. We examined the 3-way model with BPgroup+Armenia_Aknashen_N ancestry substituting PVgroup, as PVgroup belongs on the CLV cline having some southern Caucasus Neolithic ancestry along that cline. Indeed, this model fits (p=0.393) with an estimated 14.4±3.1% Aknashen ancestry.

We also confirmed that the PVgroup+Trypillian model fits the population MAJ of ref.[22] a different Eneolithic sample set from Mayaky (p=0.231) as well as the USV Usatove-related population (p=0.083). The authors of ref.[22] observe that:

“By contrast, MAJ and USV can be modelled as VAR_CA or Ukraine Trypillia (around 50%), Steppe Eneolithic (around 35%) and Caucasus Eneolithic/Maykop (around 15%) as minor third component.”

Our results agree with this general conclusion.

The Usatove population at Mayaky is then modeled as a fairly even mix of CLV Cline migrants (as those of PVgroup, or alternatively a mix of BPgroup and Aknashen) with Trypillian farmers. We note that neither YUN_CA nor Globular Amphora work for the Usatove (p<1e-4).

An interpretation of this modeling is that Usatove is formed by westward migrations of CLV Cline people into Ukraine. This migration is also evidenced in sites of the Serednii Stih culture which were formed on the basis of migrations of CLV Cline people admixing with Ukraine_N-related hunter-gatherers of the Dnipro-Don region. Thus, the CLV cline migrations westward admixed with both the hunter-gatherers of the Dnipro-Don region (creating the Serednii Stih populations along the “Dnipro Cline”) and the Trypillian farmers to the west (creating the Usatove). Their different substratum (hunter-gatherer vs. farmer) is what separates Serednii Stih from Usatove. What unifies them is their common ancestry from the CLV cline.

Usatove_I20078 (3340-3034 calBCE)

This individual from a III-C type burial at Taraclia is chronologically later and an outlier with respect to the Usatove individuals from Mayaky and is analyzed separately. Model competition (Table SI2. 15) shows that only the model that involves Core Yamna and TTK ancestry remains feasible. TTK-related ancestry entered the ancestry of the Core Yamna via its CLV-cline component as such ancestry contributed to Lower Volga-North Caucasus Eneolithic populations (like BPgroup and PVgroup which also appear as sources for this individual). However, the fact that the Core Yamna+TTK model outcompetes the BPgroup/PVgroup+TTK models suggests that the individual shares common history with the Core Yamna and is not from a population derived independently from the CLV Cline with extra TTK-related ancestry.

Table SI2. 15. Model competition for Usatove_I20078. Each column shows the p-value of a model when alternative models (rows) are placed on the Right set.

	BPgroup+TTK	CoreYamna+TTK	PVgroup+TTK
BPgroup+TTK		2.87E-01	1.81E-03
CoreYamna+TTK	2.10E-02		1.15E-01
PVgroup+TTK	1.28E-03	2.83E-01	

We sought to determine if the TTK-related ancestry in this individual could be from a source like the “Steppe Maykop”[13] which is modeled in the linked manuscript ref.[3] as having a substantial ancestry from a TTK-related “Central Asian/Siberian” source. Thus, we fit the Core Yamna+Russia_Steppe_Maykop to this individual (p=0.345) with a predicted 60.6±6.2% Steppe Maykop ancestry. The model still fits (p=0.432) in competition with the CoreYamna+TTK model while conversely the CoreYamna+TTK model fails (p<1e-4) when Steppe Maykop is added to the Right set. As the Steppe Maykop is both

temporally and geographically closer to our individual of interest and wins against it, we can conclude that this individual is of likely mixed Yamna-Steppe Maykop origins.

Zhivotilovka_I17974 (3334-3030 calBCE)

This individual from a III-C type burial at Bursuceni cannot be well-resolved using the model tournament as several models remain feasible (Table SI2. 16). The CoreYamna+TTK model (that can be fit successfully for the I20078 individual previously discussed) has the highest p-value (0.616) and low standard error (3.4%) and it is reasonable that alternative models cannot be rejected for this individual because of the much lower amount of TTK-related ancestry (~11% vs. ~35% for the I20078 individual). We also tested the CoreYamna+Steppe Maykop model (p=0.324) with an estimated 18.2±6.0% Steppe Maykop ancestry. A parsimonious interpretation of the data is that this individual (like I20078) was formed of the same Yamna+Steppe Maykop admixture process but with about ~1/3 of the Steppe Maykop ancestry found in I20078.

Table SI2. 16. Model competition for Zhivotilovka_I17974. Each column shows the p-value of a model when alternative models (rows) are placed on the Right set.

	CoreYamna+Lebyazhinka_HG	CoreYamna+TTK	GK2+PVgroup	Lebyazhinka_HG+PVgroup	PVgroup+Russia_Karelia	PVgroup+Serbia_IronGates_Mesolithic	PVgroup+Ukraine_N
CoreYamna+Lebyazhinka_HG		6.50E-01	6.99E-01	1.52E-01	1.59E-01	1.87E-01	6.40E-01
CoreYamna+TTK	3.09E-02		3.49E-01	1.08E-01	1.24E-01	5.15E-02	2.77E-01
GK2+PVgroup	2.56E-02	4.22E-01		7.01E-02	6.35E-02	7.92E-02	1.80E-01
Lebyazhinka_HG+PVgroup	7.23E-02	5.11E-01	6.18E-01		1.24E-01	1.18E-01	5.02E-01
PVgroup+Russia_Karelia	9.14E-02	6.11E-01	5.77E-01	1.42E-01		8.70E-02	5.28E-01
PVgroup+Serbia_IronGates_Mesolithic	1.17E-02	5.99E-01	3.69E-01	2.35E-01	2.04E-01		1.76E-01
PVgroup+Ukraine_N	7.51E-02	5.89E-01	4.67E-01	2.45E-01	1.95E-01	1.25E-01	

Populations that cannot be well-modeled

Three populations cannot be well-modeled according to our feasibility criteria: GlobularAmphora, Ukraine_EBA_Catacomb_possible, Zhivotilovka_I17973 even if we allow for *N*=3 sources. We examine them here separately.

GlobularAmphora (3400-2600 BCE)

If we relax our p-value threshold to 0.001, then the best 2-way model (p=0.004) for the Globular Amphora population involves 75.7±0.9% YUN_CA and 24.9±0.9% Serbia_IronGates_Mesolithic ancestry which point to this population having a higher proportion of hunter-gatherer ancestry than the Chalcolithic population from Yunatsite in Bulgaria. The obtained proportions are similar to the analysis

of a subset of Globular Amphora individuals studied in ref.[16] Due to the relative homogeneity of both European farmers (largely descended from their Anatolian predecessors[11,35]) and European hunter-gatherers (largely descended from the WHG[36] or “Villabruna[7]/Oberkassel[20] cluster”) alternative models could be feasible and the set of sources used may not be ideal for modeling the Globular Amphora population.

We were curious about the bad fit of the 2-way model and observed that it overestimates (Z=1.9-2.3) shared genetic drift with the two highland West Asian[37] population outgroups (CHG and Ganj Dareh Neolithic). As this ancestry differentiated the Neolithic people of the South Balkans from those of central-northern Europe[12,16,38] we tried an alternative model with LBK (Linearbandkeramik) as the farmer source. This is also a priori more plausible given that the Globular Amphora emerged in areas of central-eastern Europe previously occupied by LBK farmers. Indeed, this model fits well (p=0.319) with an estimated 24.7±0.9% LBK ancestry.

Trypillians (4700-2700 BCE)

The Trypillian population is heterogeneous in PCA and cannot be modeled well with any 1, 2, 3 sources. We sought to investigate whether the more notable PCA outlier within this population (I20069; 3323-2935 calBCE from Dănceni) might have different ancestry from the population as a whole. When we model I20069 separately, we do find that it has a significant amount of CLV ancestry (Table SI2. 17) and is composed of ~1/4 PVgroup and ~3/4 Trypillia ancestry (using the remaining 27 Trypillians as a source).

Table SI2. 17. Modeling Trypillia_I20069.

A	B	P-value	A	B	S.E.
BPgroup	Trypillia	0.055	22.7%	77.3%	2.2%
PVgroup	Trypillia	0.122	24.3%	75.7%	2.4%
Remontnoye	Trypillia	0.064	30.2%	69.8%	3.1%

We can also model (p=0.161) the individual as a mixture of 53.7±5.5% Usatove and Trypillia (as Usatove itself is a mixture of PVgroup and Trypillia).

Nonetheless, even after excluding the individual we cannot model Trypillians (p<1e-5 even for *N*=3 models) successfully. To understand the latent structure within this population we turned to modeling each of the 28 individuals under the “Trypillia” label separately in terms of the same sources as we used in the rest of this note (except, of course, Trypillia itself). By doing so we wanted to identify models that could be used to describe each individual separately and disclose any peculiarities of particular Trypillian individuals.

24 of 28 individuals can be modeled in our framework. The four exceptions (to which we will return below) are: VERT105B_wNonUDG.SG, VERT015_wNonUDG.SG, VERT035_wNonUDG.SG, VERT111_wNonUDG.SG from Verteba Cave.[21]

A single model (with BPgroup, YUN_CA, and Serbia Iron Gates Mesolithic ancestry) is feasible for 23 of the 24 remaining individuals. For the remaining individual (VERT103B_wNonUDG.SG) there are other feasible models. Three other models are qualitatively similar to the identified model and are feasible for 22/24 individuals; they differ from it in replacing BPgroup with PVgroup, Remontnoye, or Core Yamna. The unifying element of all these models is that they include some CLV ancestry although it is unclear which part of the CLV cline it is derived from (BPgroup/PVgroup/Remontnoye) or if it also includes some Dnipro-Don hunter-gatherer ancestry (as Core Yamna does).

We show the fit of the top model for all 28 individuals (Extended Data Table 3). We see that for many individuals there is no significant amount of BPgroup-related ancestry, but this kind of ancestry is not present only in the PCA outlier (I20069) but also in several other individuals from the population.

Of the five individuals that do not fit this model, three fit it at the p=0.01 level. We examined qpAdm output for all five individuals to identify the Right populations that cause the poor fit. Z-scores are between -2.4 and +2.2. For three individuals (VERT105B_wNonUDG.SG, VERT015_wNonUDG.SG, and VERT111_wNonUDG.SG) shared genetic drift with CHG is underestimated (Z<-2). For VERT103B_wNonUDG.SG, shared genetic drift with Ganj Dareh is overestimated (Z>2).

In conclusion, we think that Trypillians were mainly formed on the basis of the European farmer cline (represented by YUN_CA and Iron Gates hunter-gatherers) that received admixture from people that had CLV ancestry. We have seen above that Usatove was formed on the basis of CLV people absorbing Trypillian ancestry. We can synthesize the evidence from Usatove and Trypillians as follows: the movement of people with CLV ancestry into the NPR brought them into contact with European farmer populations; in Usatove groups ancestry from the two groups was fairly balanced and in Trypillian groups farmer ancestry predominated but some gene flow occurred from the CLV migrants as well. Individuals like I20069, a fairly even mix of "Usatove" and "Trypillian" groups, bridge the gap between those two differentiated populations.

Ukraine_EBA_Catacomb_possible (2874-2630 calBCE)

Individual Ukraine_EBA_Catacomb_possible (I11850; 2874-2630 calBCE) from the Odesa region cannot be modeled at the p=0.05 threshold but several models exist for it at the p=0.01 threshold (Table SI2. 18), however no single model emerges as a winner in the model tournament (Table SI2. 19). All successful models include some CLV Cline ancestry.

Table SI2. 18. Feasible models at p=0.01 level for Ukraine_EBA_Catacomb_possible.

A	B	P-value	A	B	S.E.
Armenia_Aknashen_N	CoreYamna	0.013	31.6%	68.4%	7.4%
Armenia_Aknashen_N	Lebyazhinka_HG	0.012	64.5%	35.5%	4.3%
Armenia_Aknashen_N	Russia_Karelia	0.013	65.5%	34.5%	4.0%
BPgroup	Trypillia	0.013	69.6%	30.4%	4.3%
BPgroup	YUN_CA	0.016	73.7%	26.3%	3.8%
CoreYamna	Maykop	0.011	67.0%	33.0%	8.2%
GK2	Remontnoye	0.016	12.8%	87.2%	6.6%
GlobularAmphora	PVgroup	0.010	26.6%	73.4%	4.7%
PVgroup	Trypillia	0.036	72.3%	27.7%	4.8%
PVgroup	YUN_CA	0.027	76.4%	23.6%	4.1%
Remontnoye	Russia_Karelia	0.011	91.1%	8.9%	5.3%
Remontnoye	Serbia_IronGates_Mesolithic	0.013	93.2%	6.8%	4.0%
Remontnoye	Ukraine_N	0.016	89.3%	10.7%	5.8%

Table SI2. 19. Model competition for Ukraine_EBA_Catacomb_possible. Each column shows the p-value of a model when alternative models (rows) are placed on the Right set.

	Armenia_Aknashen_N+CoreYamna	BPgroup+Trypillia	BPgroup+YUN_CA	CoreYamna+Maykop	GK2+Remontnoye	Armenia_Aknashen_N+Lebyazhinka_HG	GlobularAmphora+PVgroup	PVgroup+Trypillia	PVgroup+YUN_CA	Remontnoye+Russia_Karelia	Remontnoye+Serbia_IronGates_Mesolithic	Remontnoye+Ukraine_N	Armenia_Aknashen_N+Russia_Karelia
Armenia_Aknashen_N+CoreYamna		1.86E-05	2.70E-05	1.50E-02	2.86E-07	5.58E-15	3.49E-06	1.11E-05	1.17E-05	1.76E-07	5.56E-10	3.91E-09	4.33E-17
BPgroup+Trypillia	2.56E-03		2.35E-02	2.34E-03	6.72E-03	4.57E-07	1.89E-02	4.76E-02	4.43E-02	4.42E-03	1.67E-03	2.76E-03	4.90E-07
BPgroup+YUN_CA	6.90E-04	8.20E-03		4.48E-04	1.69E-03	3.39E-07	6.79E-03	3.23E-02	3.84E-02	1.19E-03	5.89E-04	8.29E-04	2.36E-07
CoreYamna+Maykop	1.35E-02	9.96E-06	1.08E-05		4.14E-08	1.44E-14	2.51E-07	9.55E-07	7.39E-07	2.12E-08	7.26E-12	2.25E-10	5.67E-17
GK2+Remontnoye	3.09E-03	4.50E-03	6.43E-03	2.12E-03		8.84E-05	2.70E-03	6.27E-03	5.72E-03	5.64E-03	3.21E-03	9.53E-03	1.47E-04
Armenia_Aknashen_N+Lebyazhinka_HG	2.70E-03	6.53E-03	6.54E-03	3.41E-03	5.28E-03		2.39E-03	9.00E-03	5.85E-03	2.37E-03	1.59E-03	2.98E-03	1.54E-03
GlobularAmphora+PVgroup	2.88E-02	3.74E-02	3.44E-02	2.47E-02	4.27E-02	5.96E-03		5.23E-02	3.72E-02	2.79E-02	3.13E-02	3.97E-02	5.63E-03
PVgroup+Trypillia	4.10E-02	2.50E-02	3.65E-02	2.36E-02	4.18E-02	4.68E-03	1.43E-02		3.24E-02	2.33E-02	3.22E-02	3.95E-02	5.36E-03
PVgroup+YUN_CA	1.61E-02	1.81E-02	2.57E-02	7.47E-03	1.23E-02	3.49E-03	5.12E-03	2.49E-02		8.12E-03	1.12E-02	1.32E-02	3.36E-03
Remontnoye+Russia_Karelia	5.47E-04	1.93E-03	3.02E-03	5.48E-04	7.59E-03	2.74E-05	5.16E-04	1.03E-03	9.44E-04		5.15E-05	1.44E-03	1.61E-04
Remontnoye+Serbia_IronGates_Mesolithic	4.09E-03	5.37E-03	3.27E-03	2.53E-03	2.10E-02	1.08E-04	2.85E-03	5.11E-03	1.31E-03	2.02E-03		2.29E-02	1.17E-04
Remontnoye+Ukraine_N	7.30E-04	1.85E-03	1.60E-03	7.39E-04	1.23E-02	1.00E-05	7.84E-04	1.30E-03	6.68E-04	1.80E-03	1.90E-03		3.57E-05
Armenia_Aknashen_N+Russia_Karelia	1.02E-03	7.60E-03	8.34E-03	2.00E-03	1.00E-02	1.17E-03	1.56E-03	3.38E-03	2.35E-03	1.44E-02	9.01E-05	2.11E-03	

Zhivotilovka_I17973 (3354-3103 calBCE)

The second individual from a III-C type burial at Bursuceni cannot be modeled in the same way as the first (I17974) as the Core Yamna+TTK model fails (p=6e-41). However, several models with mostly Aknashen Neolithic as one source and different European or steppe populations fit this population at the p=0.01 threshold (Table SI2. 20).

Table SI2. 20. Feasible models at p=0.01 level for Zhivotilovka_I17973.

A	B	P-value	A	B	S.E.
Armenia_Aknashen_N	BPgroup	0.012	91.2%	8.8%	4.1%
Armenia_Aknashen_N	CoreYamna	0.012	90.5%	9.5%	4.7%
Armenia_Aknashen_N	GK2	0.010	94.5%	5.5%	2.8%
Armenia_Aknashen_N	Lebyazhinka_HG	0.011	95.0%	5.0%	2.4%
Armenia_Aknashen_N	Remontnoye	0.011	85.1%	14.9%	8.1%
Armenia_Aknashen_N	Russia_Karelia	0.013	95.3%	4.7%	2.2%
Armenia_Aknashen_N	Serbia_IronGates_Mesolithic	0.011	96.0%	4.0%	2.1%
Armenia_Aknashen_N	Ukraine_N	0.012	94.6%	5.4%	2.5%

The admixture proportions of this individual point to a population of the Caucasus Neolithic (like Aknashen) with a little steppe ancestry. The Maykop population is a similar blend (it can be modeled as 86.2±2.9% Aknashen and 13.8±2.9% BPgroup as discussed in ref.[3]), however it does not suffice as a source for this individual (p=0.0025). Neither does Aknashen Neolithic by itself (p=0.0047). All other single sources fail convincingly (p<1e-32). Examining the qpAdm output of 2-way models we observed that shared genetic drift with all the outgroups was positive which suggests that the individual might have ancestry from a source that has genetic drift not shared with any of the outgroups. Provisionally, we can say that this individual has ancestry from the Caucasus based on its position in PCA and the fact that Maykop is its closest (albeit imperfect) single source: the precise origin of this individual remains unclear.

Re-analysis of Early Bronze Age outliers from Ukraine from Mathieson et al. (2018) (ref.[16])

We discuss separately four previously published individuals from ref.[16] for two of which we report additional data: Ukraine_EBA_Deriivka_I4110, Ukraine_EBA_Deriivka_I5882, Ukraine_EBA_Deriivka_I5884, Ukraine_EBA_Ozera_I1917.

Individual I1917 (Ozera; 3096-2913 calBCE) was described as having some Anatolian Neolithic-related ancestry in its original publication.[16] It can be modeled as a 2-way mixture in our framework of more proximate sources (Table SI2. 21) all of which do indeed possess such Anatolian Neolithic-related ancestry.

Model competition identifies a single feasible model for this individual that involves half Core Yamna and half Maykop ancestry (Table SI2. 22). This may have come about either by the migration of Yamna into the North Caucasus and its admixture with Maykop and successor populations followed by the individual's migration to Ukraine or, alternatively, the admixture may have taken place in Ukraine itself.

Table SI2. 21. Feasible 2-way models for Ukraine_EBA_Ozera_I1917.

A	B	P-value	A	B	S.E.
Armenia_Aknashen_N	CoreYamna	0.089	41.7%	58.3%	4.1%
CoreYamna	Maykop	0.345	50.1%	49.9%	4.5%
GK2	Maykop	0.118	26.7%	73.3%	2.6%
GlobularAmphora	Remontnoye	0.101	12.3%	87.7%	3.4%
Lebyazhinka_HG	Maykop	0.127	23.9%	76.1%	2.2%
Remontnoye	Serbia_IronGates_Mesolithic	0.083	91.8%	8.2%	2.2%

Table SI2. 22. Model competition for Ukraine_EBA_Ozera_I1917. Each column shows the p-value of a model when alternative models (rows) are placed on the Right set.

	Armenia_Aknashen_N+CoreYamna	CoreYamna+Maykop	GK2+Maykop	GlobularAmphora+Remontnoye	Lebyazhinka_HG+Maykop	Remontnoye+Serbia_IronGates_Mesolithic
Armenia_Aknashen_N+CoreYamna		4.25E-01	7.28E-13	2.13E-03	4.84E-13	2.12E-02
CoreYamna+Maykop	4.18E-03		1.76E-13	1.73E-04	1.33E-13	1.71E-03
GK2+Maykop	1.02E-02	4.14E-01		4.30E-03	1.58E-01	8.49E-03
GlobularAmphora+Remontnoye	1.57E-01	4.18E-01	1.76E-02		1.58E-02	9.29E-02
Lebyazhinka_HG+Maykop	6.36E-04	5.39E-02	6.21E-02	1.29E-03		1.34E-03

Remontnoye+Serbia_IronGates_Mesolithic	1.43E-01	2.55E-01	1.59E-02	2.60E-02	4.08E-03	

The other three individuals of interest (all from Deriivka) cannot be modeled as 2-way mixtures and were described as having steppe, hunter-gatherer, and Anatolian Neolithic ancestry in ref.[16]. We identified six 3-way models that are applicable to all three individuals (Table SI2. 23) which indeed combine Anatolian Neolithic-related (via either YUN_CA, Trypillian, or Globular Amphora European populations) ancestry, hunter-gatherer ancestry (represented by Iron Gates hunter-gatherers) and steppe ancestry (either North Caucasus-Lower Volga Eneolithic BPgroup/PVgroup or Core Yamna).

Model competition for each of the three individuals (Table SI2. 24; Table SI2. 25; Table SI2. 26) identifies a single model (with Core Yamna as the steppe source and Trypillians as the farmer source). The amount of Balkan hunter-gatherer ancestry in these three individuals is substantial (~23-44%) and thus provides more evidence (to that of the Multi-Cordoned Ware / Babine) for admixture with populations of high hunter-gatherer ancestry during the Early Bronze Age. They provide additional evidence for the Bronze Age persistence of high hunter-gatherer ancestry from the Chalcolithic also seen in Romania and remarked on in our discussion of the Babine MBA populations.

Table SI2. 23. 3-way models applicable to all three Ukraine_EBA outlier individuals. The model that is feasible for all three individuals in the tournament approach is highlighted in bold.

					Proportions			Std. errors		
**Test**	**A**	**B**	**C**	**P-value**	**A**	**B**	**C**	**A**	**B**	**C**
Ukraine_EBA_Deriivka_I4110	BPgroup	Serbia_IronGates_Mesolithic	Trypillia	0.202	37.5%	24.5%	38.0%	2.8%	2.6%	2.7%
Ukraine_EBA_Deriivka_I4110	BPgroup	Serbia_IronGates_Mesolithic	YUN_CA	0.113	39.4%	29.5%	31.0%	2.7%	2.4%	2.1%
Ukraine_EBA_Deriivka_I4110	CoreYamna	GlobularAmphora	Serbia_IronGates_Mesolithic	0.118	49.2%	33.2%	17.6%	3.2%	3.2%	2.8%
**Ukraine_EBA_Deriivka_I4110**	**CoreYamna**	**Serbia_IronGates_Mesolithic**	**Trypillia**	**0.216**	**45.6%**	**22.5%**	**32.0%**	**3.2%**	**2.6%**	**3.0%**
Ukraine_EBA_Deriivka_I4110	CoreYamna	Serbia_IronGates_Mesolithic	YUN_CA	0.136	47.6%	26.6%	25.9%	3.0%	2.4%	2.3%
Ukraine_EBA_Deriivka_I4110	PVgroup	Serbia_IronGates_Mesolithic	Trypillia	0.054	38.2%	26.3%	35.5%	2.8%	2.5%	2.7%
Ukraine_EBA_Deriivka_I5882	BPgroup	Serbia_IronGates_Mesolithic	Trypillia	0.776	37.1%	42.5%	20.5%	2.6%	2.5%	2.5%
Ukraine_EBA_Deriivka_I5882	BPgroup	Serbia_IronGates_Mesolithic	YUN_CA	0.780	38.0%	45.2%	16.8%	2.5%	2.4%	2.0%
Ukraine_EBA_Deriivka_I5882	CoreYamna	GlobularAmphora	Serbia_IronGates_Mesolithic	0.918	46.8%	15.2%	38.0%	2.9%	2.7%	2.7%
**Ukraine_EBA_Deriivka_I5882**	**CoreYamna**	**Serbia_IronGates_Mesolithic**	**Trypillia**	**0.899**	**45.4%**	**40.2%**	**14.5%**	**3.0%**	**2.5%**	**2.6%**
Ukraine_EBA_Deriivka_I5882	CoreYamna	Serbia_IronGates_Mesolithic	YUN_CA	0.919	46.1%	42.0%	11.9%	2.9%	2.5%	2.1%
Ukraine_EBA_Deriivka_I5882	PVgroup	Serbia_IronGates_Mesolithic	Trypillia	0.547	37.9%	44.0%	18.1%	2.7%	2.6%	2.5%
Ukraine_EBA_Deriivka_I5884	BPgroup	Serbia_IronGates_Mesolithic	Trypillia	0.108	29.6%	45.2%	25.2%	2.5%	2.5%	2.4%
Ukraine_EBA_Deriivka_I5884	BPgroup	Serbia_IronGates_Mesolithic	YUN_CA	0.075	30.8%	48.6%	20.6%	2.5%	2.3%	2.1%
Ukraine_EBA_Deriivka_I5884	CoreYamna	GlobularAmphora	Serbia_IronGates_Mesolithic	0.095	38.3%	20.9%	40.8%	3.0%	2.9%	2.7%
**Ukraine_EBA_Deriivka_I5884**	**CoreYamna**	**Serbia_IronGates_Mesolithic**	**Trypillia**	**0.179**	**36.0%**	**43.5%**	**20.4%**	**2.9%**	**2.4%**	**2.6%**
Ukraine_EBA_Deriivka_I5884	CoreYamna	Serbia_IronGates_Mesolithic	YUN_CA	0.142	37.3%	46.2%	16.5%	3.0%	2.5%	2.1%
Ukraine_EBA_Deriivka_I5884	PVgroup	Serbia_IronGates_Mesolithic	Trypillia	0.151	30.4%	46.7%	22.8%	2.6%	2.4%	2.6%

Table SI2. 24. Model competition for Ukraine_EBA_Deriivka_I4110. Each column shows the p-value of a model when alternative models (rows) are placed on the Right set.

	BPgroup+Serbia_IronGates_Mesolithic+Trypillia	BPgroup+Serbia_IronGates_Mesolithic+YUN_CA	CoreYamna+GlobularAmphora+Serbia_IronGates_Mesolithic	CoreYamna+Serbia_IronGates_Mesolithic+Trypillia	CoreYamna+Serbia_IronGates_Mesolithic+YUN_CA	PVgroup+Serbia_IronGates_Mesolithic+Trypillia
BPgroup+Serbia_IronGates_Mesolithic+Trypillia		1.14E-01	2.13E-01	3.28E-01	2.82E-01	5.35E-02
BPgroup+Serbia_IronGates_Mesolithic+YUN_CA	1.70E-01		1.81E-01	3.40E-01	2.24E-01	5.48E-02
CoreYamna+GlobularAmphora+Serbia_IronGates_Mesolithic	1.81E-03	7.24E-04		1.81E-01	1.09E-01	1.40E-04
CoreYamna+Serbia_IronGates_Mesolithic+Trypillia	1.28E-03	1.17E-03	1.28E-01		1.76E-01	6.42E-05
CoreYamna+Serbia_IronGates_Mesolithic+YUN_CA	1.48E-03	5.35E-04	1.15E-01	2.31E-01		8.93E-05
PVgroup+Serbia_IronGates_Mesolithic+Trypillia	2.79E-01	1.59E-01	1.22E-01	1.70E-01	1.64E-01	

Table SI2. 25. Model competition for Ukraine_EBA_Deriivka_I5882. Each column shows the p-value of a model when alternative models (rows) are placed on the Right set.

	BPgroup+Serbia_IronGates_Mesolithic+Trypillia	BPgroup+Serbia_IronGates_Mesolithic+YUN_CA	CoreYamna+GlobularAmphora+Serbia_IronGates_Mesolithic	CoreYamna+Serbia_IronGates_Mesolithic+Trypillia	CoreYamna+Serbia_IronGates_Mesolithic+YUN_CA	PVgroup+Serbia_IronGates_Mesolithic+Trypillia
BPgroup+Serbia_IronGates_Mesolithic+Trypillia		1.45E-06	1.32E-05	8.00E-01	3.42E-05	6.73E-02
BPgroup+Serbia_IronGates_Mesolithic+YUN_CA	3.17E-02		1.06E-01	1.14E-01	8.17E-01	3.65E-03
CoreYamna+GlobularAmphora+Serbia_IronGates_Mesolithic	4.83E-01	4.37E-01		8.92E-01	9.00E-01	1.57E-01
CoreYamna+Serbia_IronGates_Mesolithic+Trypillia	4.12E-01	1.76E-06	8.57E-06		2.17E-05	1.38E-01

CoreYamna+Serbia_IronGates_Mesolithic+YUN_CA	1.61E-02	3.76E-01	8.82E-02	1.01E-01		6.05E-03
PVgroup+Serbia_IronGates_Mesolithic+Trypillia	4.02E-01	7.03E-07	2.54E-05	9.56E-01	6.29E-05	

Table SI2. 26. Model competition for Ukraine_EBA_Deriivka_I5884. Each column shows the p-value of a model when alternative models (rows) are placed on the Right set.

	BPgroup+Serbia_IronGates_Mesolithic+Trypillia	BPgroup+Serbia_IronGates_Mesolithic+YUN_CA	CoreYamna+GlobularAmphora+Serbia_IronGates_Mesolithic	CoreYamna+Serbia_IronGates_Mesolithic+Trypillia	CoreYamna+Serbia_IronGates_Mesolithic+YUN_CA	PVgroup+Serbia_IronGates_Mesolithic+Trypillia
BPgroup+Serbia_IronGates_Mesolithic+Trypillia		6.19E-03	2.19E-02	2.77E-01	4.85E-02	7.74E-02
BPgroup+Serbia_IronGates_Mesolithic+YUN_CA	1.75E-01		2.37E-01	3.68E-01	2.27E-01	1.21E-01
CoreYamna+GlobularAmphora+Serbia_IronGates_Mesolithic	1.48E-02	9.75E-03		1.16E-01	9.72E-02	1.49E-02
CoreYamna+Serbia_IronGates_Mesolithic+Trypillia	2.35E-02	2.93E-03	1.26E-02		2.95E-02	1.55E-02
CoreYamna+Serbia_IronGates_Mesolithic+YUN_CA	4.08E-02	1.46E-02	1.56E-01	2.65E-01		2.78E-02
PVgroup+Serbia_IronGates_Mesolithic+Trypillia	1.97E-01	1.46E-02	1.61E-02	1.34E-01	3.53E-02	

Re-analysis of data from Penske et al. (2023) (ref.[22])

We also re-analyzed the data from Penske et al. which partially overlap with the data of our study with regard to the cultural distribution of the newly reported data. We have already mentioned in passing that the MAJ population (from Mayaky) and USV from Usatove-Velykyj Kuyalnik can fit similarly to our Usatove population from the Mayaky site. We now look at other populations from the study.

Chalcolithic Southeastern Europe (4900-3800 BCE)

We used YUN_CA (Yunatsite) in our modeling as a source. According to ref.[22]:
"The genetic homogeneity observed in and across the four CA sites (PIE, YUN, PTK and VAR) of the fifth millennium BC matches the cultural homogeneity of the archaeological records and suggests an extended period of a relative stable sociopolitical network and absence of large-scale cultural and genetic transformations."

We confirm that PTK_CA (Tell Petko Karavelovo) is a clade with YUN_CA (p=0.146).

PIE_CA (Pietrele) does not form a clade with YUN_CA (p<1e-8) although it fits with that population as a source better than with any alternative (p<1e-107). Several subsets of PIE were analyzed separately in ref.[22] PIE039 (the earliest dated individual from the site) is a clade with YUN_CA (p=0.805). The individual PIE060 has 32.6±2.0% Iron Gates hunter-gatherer ancestry (p=0.755) in agreement with the observations of ref.[22] Individual PIE078 is not a clade with YUN_CA (p<1e-15) but is a clade with

Trypillia (p=0.374). Overall, we assess that due to the large number of individuals in the PIE_CA population and the existence of outlier individuals at the site this population is overall similar to YUN_CA but includes other diversity that makes it fail the clade test with YUN_CA.

VAR_CA (Varna) does not form a clade with YUN_CA or any other population (p<1e-32) but is well-modeled as a mixture of YUN_CA and 8.8±0.7% Ukraine_N ancestry (p=0.154).

Kartal clusters A and B (4150-3400 BCE)

Of the two clusters at Kartal (ca. 4150–3400 BC; clusters KTL_A and KTL_B) we can model well KTL_A as a mixture of Trypillians and 53.9±1.2% BPgroup ancestry (p=0.618). The model PVgroup+Trypillia fails marginally (p=0.043).

In ref.[22] KTL_A is modeled as Steppe Eneolithic (corresponding to BPgroup or PVgroup in our setup) and VAR_CA. Both these models fail in our setup (p=6e-6 for PVgroup and p=0.002 for BPgroup). Examining qpAdm output we see that these models underestimate (Z=-4.3 and Z=-3.8 respectively) shared genetic drift with the Villabruna (WHG-related outgroup).

We conclude that the best reconstruction of this population of early steppe migrants is a combination of half Lower Volga-North Caucasus Eneolithic (BPgroup or PVgroup) and a Trypillian-related source. This scenario makes geographical sense as we do find unadmixed eastern migrants at Giurgiuleşti and we also know that these eastern migrants did admix with Trypillians to form Usatove (and USV/MAJ) and KTL_A could be derived from the same type of mixture. Note, however, that Usatove has a clear preference (as we explained above) for a PVgroup source and definitely has Aknashen-related ancestry from the Caucasus; the model without any such ancestry is the one that works for KTL_A. Thus, KTL_A represents a southern extension of CLV Cline migrants into Southeastern Europe, derived in this case from the Lower Volga end of the cline.

To verify this claim, we add as a 3rd population either Aknashen Neolithic or Lebyazhinka_HG: BPgroup stands at the end of two clines: the CLV cline to its south and the upriver Volga cline. By adding either of these two sources we can estimate where along either of these clines the admixing population was derived from. These two models are generalizations of the basic BPgroup+Trypillia model that fits KTL_A allowing for one additional source of ancestry (Table SI2. 27). The proportion of Lebyazhinka ancestry is low and non-significant (1.6±2.2%) and so is that of Aknashen ancestry (-2.6±1.9%). Thus, we see no evidence that a population other than the BPgroup was involved in the westward migration that contributed to the KTL_A ancestry. We also add as a 3rd source either Ukraine_N or GK2 to represent Dnipro-Don hunter-gatherer ancestry; these two are low and non-significant (2.8±2.1% or 2.7±2.3%). Thus, we see no evidence that the westward migrants picked any Dnipro-Don hunter-gatherer ancestry from the intervening territory between Trypillians and the Lower Volga.

Table SI2. 27. KTL_A is derived from a BPgroup+Trypillia mixture even when adding sources defining the CLV (Aknashen) or Volga (Lebyazhinka) clines or allowing for Dnipro-Don hunter-gatherer (Ukraine_N or GK2) ancestry.

				Proportions			Std. errors		
A	B	C	P-value	A	B	C	A	B	C
Armenia_Aknashen_N	BPgroup	Trypillia	0.555	-2.6%	55.0%	47.6%	3.4%	1.9%	2.3%
BPgroup	GK2	Trypillia	0.631	51.6%	2.7%	45.7%	2.3%	2.3%	1.3%
BPgroup	Lebyazhinka_HG	Trypillia	0.476	52.2%	1.6%	46.2%	2.6%	2.2%	1.3%
BPgroup	Trypillia	Ukraine_N	0.749	51.9%	45.3%	2.8%	2.0%	1.4%	2.1%

Kartal B (KTL_B) does not fit any 2-way models; the only feasible one with p>0.001 involves 29.2±2.5% Maykop and 70.8±2.5%% Trypillian ancestry (p=0.011). This model underestimates shared genetic drift with Afontova Gora 3 (Z=-3.5).

We examined 3-way models (Table SI2. 28). These invariably involve a CLV cline population (Aknashen, Maykop, or Remontnoye), a Dnipro-Don population (Ukraine_N or GK2) or Globular Amphora and YUN_CA.

Table SI2. 28. 3-way admixture models for KTL_B.

				Proportions			Std. errors		
A	B	C	P-value	A	B	C	A	B	C
Armenia_Aknashen_N	Ukraine_N	YUN_CA	0.096	26.3%	16.4%	57.2%	2.7%	1.4%	2.5%
GK2	Maykop	YUN_CA	0.064	14.3%	24.7%	61.0%	1.6%	2.7%	2.2%
GlobularAmphora	Remontnoye	YUN_CA	0.294	27.3%	29.6%	43.1%	5.0%	2.0%	4.5%
Maykop	Ukraine_N	YUN_CA	0.207	26.8%	14.2%	59.0%	2.4%	1.4%	2.1%
Remontnoye	Serbia_IronGates_Mesolithic	YUN_CA	0.370	28.2%	7.3%	64.5%	2.0%	1.2%	1.8%

The model tournament competition between these models (Table SI2. 29) excludes the models that involve Aknashen and GK2 ancestry but is otherwise inconclusive.

Table SI2. 29. Model competition for KTL_B. Each column shows the p-value of a model when alternative models (rows) are placed on the Right set.

	GK2+Maykop+YUN_CA	GlobularAmphora+Remontnoye+YUN_CA	Maykop+Ukraine_N+YUN_CA	Remontnoye+Serbia_IronGates_Mesolithic+YUN_CA	Armenia_Aknashen_N+Ukraine_N+YUN_CA
GK2+Maykop+YUN_CA		1.08E-01	2.68E-01	6.78E-02	1.99E-02
GlobularAmphora+Remontnoye+YUN_CA	1.76E-01		4.47E-01	5.16E-01	2.19E-01
Maykop+Ukraine_N+YUN_CA	3.34E-02	5.84E-02		1.18E-02	8.47E-03
Remontnoye+Serbia_IronGates_Mesolithic+YUN_CA	1.80E-01	3.99E-01	2.00E-01		4.18E-02
Armenia_Aknashen_N+Ukraine_N+YUN_CA	3.98E-02	4.25E-01	2.15E-01	2.78E-01	

What is clear from the modeling of KTL_B is that it (i) has European farmer ancestry as its primary component, and (ii) that it has CLV cline ancestry. The CLV Cline ancestry is not (in contrast to KTL_A) from the northern (lower Volga) BPgroup end; rather it is from intermediate populations such as Maykop or Remontnoye.

An interpretation of these findings is that at least two groups participated in the westward movement of CLV Cline people into the ancestors of the Kartal populations: one consisting of unadmixed lower Volga-related (BPgroup) into the ancestors of KTL_A and another, consisting of mixed lower Volga-Caucasus Neolithic (Remontnoye or Maykop-related) into the ancestors of KTL_B.

In ref.[22] KTL_B is modeled in alternative ways, as a distal mix of CHG/EHG/Anatolian Neolithic; as a mixture of VAR_CA or Trypillians, Steppe Eneolithic (BPgroup or PVgroup in our setup), and Maykop; or as a simpler model of 12% Trypillian and 88% Maykop. This simpler 2-way model underestimates shared genetic drift with Afontova Gora 3 as we mentioned above; the models of Table SI2. 28 account for this shared history in alternative ways: either (i) via Ukraine_N ancestry (itself a mixture of EHG and WHG ancestries[16], with EHG being a mixture of Ancient North Eurasians like Afontova Gora3 and WHG[2]) or (ii) via Remontnoye ancestry (itself a mixture of BPgroup and Maykop/Aknashen, thus receiving Afontova Gora 3-related ancestry via BPgroup).

Early Bronze Age (BOY_EBA and MAJ_EBA) (3300-2500 BCE)

We show fitting 2-way models for Boyanovo and Mayaky populations of the Early Bronze Age in Table SI2. 30. The EBA from Boyanovo like previously published data[12] and data included in ref.[3] and analyzed above as Bulgaria_EBA_Yamna can be modeled as a mixture of Core Yamna and European farmers.

Table SI2. 30. Modeling BOY_EBA and MAJ_EBA.

Test	A	B	P-value	A	B	S.E.
MAJ_EBA	GK2	Remontnoye	0.511	32.9%	67.1%	3.0%
MAJ_EBA	Remontnoye	Ukraine_N	0.263	69.6%	30.4%	2.6%
BOY_EBA	BPgroup	GlobularAmphora	0.255	77.8%	22.2%	1.5%
BOY_EBA	CoreYamna	GlobularAmphora	0.658	92.1%	7.9%	1.5%
BOY_EBA	CoreYamna	Trypillia	0.396	92.4%	7.6%	1.5%
BOY_EBA	CoreYamna	YUN_CA	0.211	93.8%	6.2%	1.3%
BOY_EBA	GK2	Remontnoye	0.952	32.3%	67.7%	2.5%

The results of the model tournament (Table SI2. 31) show as above (Table SI2. 5) that models involving Yamna ancestry are the only feasible ones.

Table SI2. 31. Model competition for BOY_EBA. Each column shows the p-value of a model when alternative models (rows) are placed on the Right set.

	BPgroup+GlobularAmphora	CoreYamna+GlobularAmphora	CoreYamna+Trypillia	CoreYamna+YUN_CA	GK2+Remontnoye
BPgroup+GlobularAmphora		5.73E-01	3.78E-01	1.63E-01	8.25E-01
CoreYamna+GlobularAmphora	3.21E-11		4.85E-01	2.64E-01	9.78E-21
CoreYamna+Trypillia	4.35E-11	7.74E-02		4.12E-02	5.38E-22
CoreYamna+YUN_CA	6.70E-11	7.56E-01	4.86E-01		1.88E-18
GK2+Remontnoye	2.00E-01	2.48E-01	1.64E-01	8.51E-02	

Curiously, no models with Yamna ancestry appear as feasible for the EBA from Mayaky (MAJ_EBA) in Table SI2. 30. The model with CoreYamna+GlobularAmphora fails at the p=0.05 threshold (p=0.019). We examined 3-way models with Core Yamna as one of the sources. These models involve both about half to two thirds Yamna ancestry but also Dnipro-Don (Ukraine_N or GK2) ancestry as well as Caucasus Neolithic-related (Aknashen or Maykop) ancestry.

Table SI2. 32. Feasible 3-way models for MAJ_EBA with Core Yamna as one of the sources.

				Proportions			Std. errors		
A	B	C	P-value	A	B	C	A	B	C
Armenia_Aknashen_N	CoreYamna	GK2	0.054	21.6%	55.5%	22.9%	5.0%	9.8%	5.5%
CoreYamna	GK2	Maykop	0.163	50.7%	23.0%	26.3%	9.5%	5.0%	5.2%
CoreYamna	Maykop	Ukraine_N	0.084	64.2%	19.6%	16.2%	6.3%	4.0%	3.2%

We have seen above in our analysis of KTL_B that Maykop-related ancestry flowed westwards during the Eneolithic and so its presence in MAJ_EBA could be related to that earlier migration. The combination of Core Yamna with GK2/Ukraine_N in the models of Table SI2. 32 defines populations of the "Dnipro Cline" to which the Serednii Stih belonged. We show in linked manuscript ref.[3] that the Yamna of the lower Don had absorbed Serednii Stih ancestry and can be modeled as a mixture of Core Yamna with Serednii Stih populations having more Dnipro-Don (Ukraine_N/GK2) ancestry. To test if either Serednii Stih or Don Yamna could be contributing ancestry to MAJ_EBA we included Serednii Stih subsets (SShi, SSmed, SSlo) and Don Yamna as additional sources of ancestry.

We do indeed find simpler 2-way models with these additional sources of ancestry (Table SI2. 33) that involve either Don Yamna or Serednii Stih. However, only models with Don Yamna and CLV Cline

populations are feasible in the model tournament competition (Table SI2. 34); the model with ~81% Don Yamna and ~19% Maykop ancestry fares particularly well. Recall that we inferred Core Yamna + Maykop admixture in the ancestry of Ukraine_EBA_Ozera_I1917. The EBA population at Mayaky represents another such mixture of Don Yamna and Maykop-related ancestry.

Table SI2. 33. Feasible 2-way models for MAJ_EBA when Don Yamna and Serednii Stih are included as additional proximate sources.

A	B	P-value	A	B	S.E.
Armenia_Aknashen_N	Russia_Don_EBA_Yamna	0.265	15.8%	84.2%	2.9%
GK2	Remontnoye	0.511	32.9%	67.1%	3.0%
Maykop	SSmed	0.069	29.3%	70.7%	3.2%
Maykop	Russia_Don_EBA_Yamna	0.424	18.9%	81.1%	3.1%
Remontnoye	Ukraine_N	0.263	69.6%	30.4%	2.6%
Remontnoye	SSmed	0.288	43.8%	56.2%	4.6%
Remontnoye	SSlo	0.421	58.5%	41.5%	4.3%
Remontnoye	Russia_Don_EBA_Yamna	0.174	29.2%	70.8%	5.2%

The BOY_EBA (and Southeastern European EBA populations in general) thus contrast with the MAJ_EBA: the former were formed on the basis of Yamna admixing with European farmer populations they encountered as they went beyond the steppe and into the territory of settled agriculturalists in the west; the latter had ancestry from the Don Yamna with Maykop-related people from the North Caucasus in the east.

Table SI2. 34. Model competition for MAJ_EBA. Each column shows the p-value of a model when alternative models (rows) are placed on the Right set.

	Maykop+SSmed	Maykop+Russia_Don_EBA_Yamna	Remontnoye+Ukraine_N	Remontnoye+SSmed	Remontnoye+SSlo	Remontnoye+Russia_Don_EBA_Yamna	Armenia_Aknashen_N+Russia_Don_EBA_Yamna	GK2+Remontnoye
Maykop+SSmed		5.20E-01	2.00E-01	5.80E-02	1.15E-01	1.17E-01	2.06E-01	5.05E-01
Maykop+Russia_Don_EBA_Yamna	4.99E-11		5.56E-12	6.17E-08	5.43E-07	1.77E-01	2.00E-01	1.08E-06
Remontnoye+Ukraine_N	2.78E-03	3.11E-01		1.85E-01	3.09E-01	1.34E-01	2.43E-01	3.96E-01
Remontnoye+SSmed	1.58E-02	4.84E-01	3.59E-01		5.62E-01	1.37E-01	3.51E-01	6.08E-01
Remontnoye+SSlo	1.83E-02	5.37E-01	3.33E-01	3.89E-01		2.66E-01	4.41E-01	6.83E-01
Remontnoye+Russia_Don_EBA_Yamna	2.19E-10	4.40E-01	2.04E-12	1.51E-07	3.07E-06		3.35E-01	3.41E-07
Armenia_Aknashen_N+Russia_Don_EBA_Yamna	1.08E-10	4.78E-01	8.83E-13	1.04E-07	5.82E-06	1.30E-01		4.81E-07
GK2+Remontnoye	2.70E-02	5.97E-01	3.71E-01	3.03E-01	5.58E-01	2.62E-01	4.91E-01	

We sought to investigate if the Maykop-related admixture in MAJ_EBA was driven by any particular individual, so we fit the model to the three individuals from the site, confirming that it was indeed present in all of them (Table SI2. 35).

Table SI2. 35. Admixture of MAJ_EBA individuals.

Individual	P-value	Maykop	Russia_Don_EBA_Yamna	S.E.
MAJ004.merge.bam	0.417	20.0%	80.0%	4.4%
MAJ017.merge.bam	0.581	17.9%	82.1%	4.0%
MAJ019.merge.bam	0.263	21.7%	78.3%	4.4%

Timing the admixture of Caucasus-Lower Volga and European farmer groups

Both Usatove-related (Usatove, MAJ, and USV) and Kartal (KTL_A and KTL_B) populations are inferred to be admixtures of Caucasus Lower Volga people with European farmers. We use DATES[39] to date this admixture in number of generations and convert this to a calendar date by multiplying with 28 years per generation.[40] Our estimates place this admixture in the second half of the 5th millennium BCE (Figure SI2. 2) when we use (YUN_CA, Trypillia, GlobularAmphora, LBK) as the “European farmer” source and (PVgroup, BPgroup, Khi, Csongrád_I5124, Giurgiuleşti) as the “Caucasus-Volga” source. Removing Trypillia from the “European farmer” set and estimating its own admixture time, we obtain a similar date. This supports the idea that the formation of Trypillians (with little CLV ancestry) and Usatove (with about half) were part of the same mid-5th millennium BCE process.

This timeframe corresponds also with the emergence and efflorescence of the Serednii Stih culture from which the Yamna ancestral population was formed.

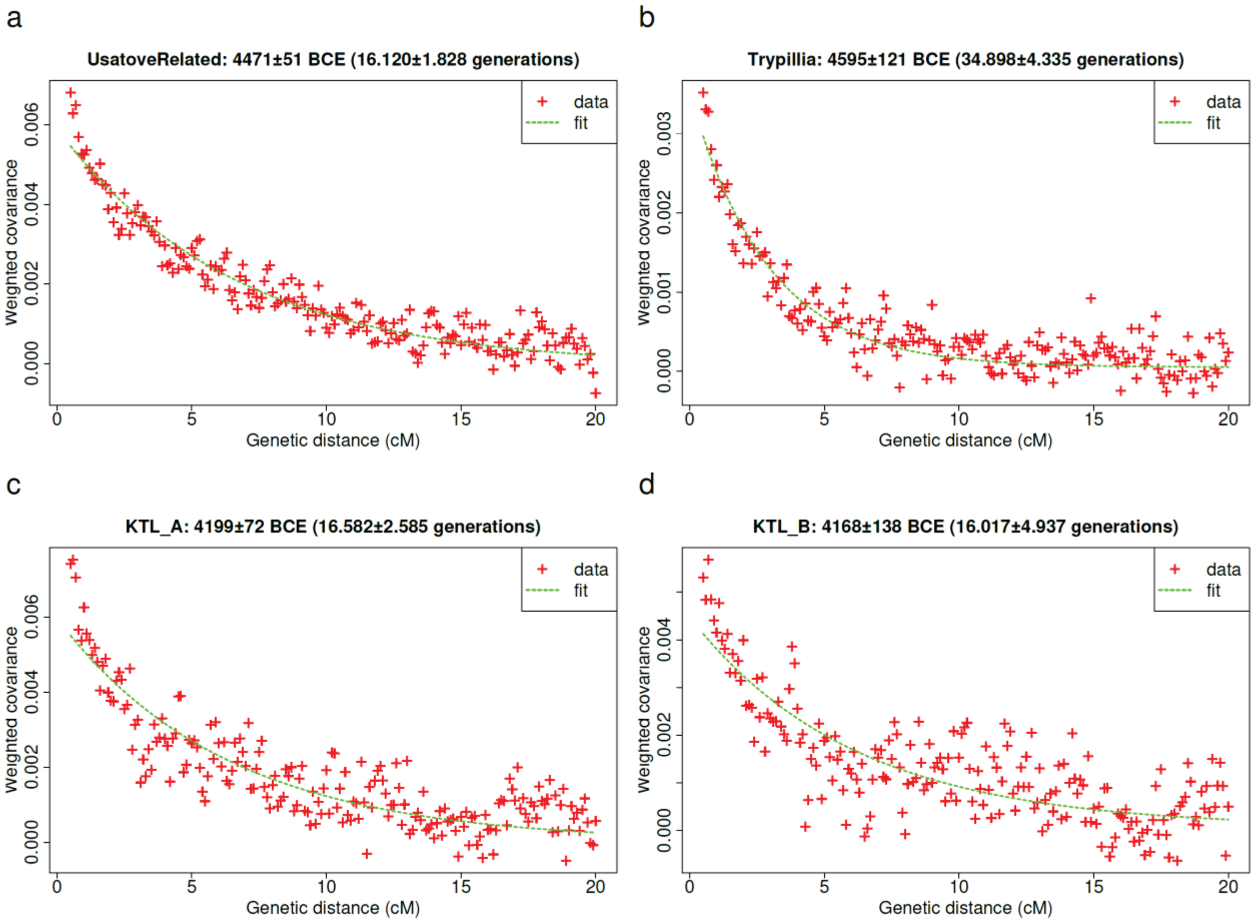

Figure SI2. 2. DATES estimates of admixture timing of CLV and European farmer ancestry admixture.

Summary of key findings

The discussion of this note suggests a complex landscape of interactions between people of the Pontic-Caspian steppe and the diverse descendants of European farmers and hunter-gatherers both before and after the formation and expansion of the Yamna archaeological complex. We extract from our observations a list of key findings; we refer the reader to the preceding discussion for details of the modeling and possible alternative interpretations. The list below represents our synthesizing of the modeling of this note in a way that is not contradicted by the available evidence and should be evaluated critically in the future, both to confirm some of our conjectures and to propose alternative explanations for the available evidence.

1. We find relatively unadmixed representatives of the two expansive populations of the Eneolithic and Early Bronze Age: the Lower Volga-North Caucasus Eneolithic (BPgroup and PVgroup) and the Core Yamna (Table SI2. 1). Of particular importance is the Eneolithic individual from Giurgiuleşti in Moldova which, together with the individual from Csongrád in Hungary discussed in ref.[3] exemplifies the migration of unadmixed people from the Volga westwards. Both these migrants had no detectible ancestry from either the people of the Dnipro-Don region (through which they passed in their migratory route) or the European farmer populations that were settled near their sampling locations. We also find unadmixed Core Yamna either as individuals in

archaeologically uncertain contexts (Table SI2. 1) and also among the Yamna of Bulgaria, Moldova, and Ukraine.
2. In Ukraine, Moldova, and Bulgaria, the Yamna included many individuals who had acquired European farmer ancestry (Table SI2. 6; Table SI2. 9).
3. An individual from Durankulak in Bulgaria is of particular interest as he possesses the "Corded Ware"-related R-M417 Y-haplogroup and a similar mix of Core Yamna and Globular Amphora ancestry as the Corded Ware (but with more ancestry from the Globular Amphora). This individual may stem from the admixture zone between the Core Yamna and the Globular Amphora from which the characteristic 3:1 blend[2,31] of the two components in the ancestry of the Corded Ware is derived.
4. There is some evidence of admixture between the expansive Yamna and populations of high hunter-gatherer ancestry in the Multi-Cordoned Ware / Babine. The existence of groups of both high and low hunter-gatherer ancestry has been observed in the Romanian Bronze Age[12]. The Yamna admixed with diverse farmer groups of both low hunter-gatherer ancestry (as the YUN_CA /Yunatsite of Bulgaria), higher such ancestry (as the Trypillians of Moldova-Ukraine), and higher still (as the Globular Amphora of Ukraine-Poland), and indeed other groups of even higher such ancestry as evidenced by both the Multi-Cordoned Ware / Babine and the Deriivka outliers of the Early Bronze Age.
5. The Yamna also admixed with the Steppe Maykop carrying this mix westwards to Taraclia (Table SI2. 15) and Bursuceni (Table SI2. 16). Yet, at Bursuceni we also find evidence of another individual whose ancestry is unclear but is connected to the Caucasus (Table SI2. 20).
6. The Yamna also admixed with the Maykop or related populations as in Ukraine_EBA_Ozera_I1917 and MAJ_EBA. The latter population is the single example we know of the participation of migrations related to the Lower Don Yamna rather than the Core Yamna.
7. The CLV cline ancestry reached as far south as Bulgaria where a singleton individual from Riltsi possessed it in combination with local farmer ancestry (Table SI2. 7).
8. The Usatove population was formed on the basis of North Caucasus Eneolithic ancestry (as in PVgroup) and Trypillians. It thus contrasts with the Serednii Stih which was formed in combination with Dnipro-Don hunter-gatherer ancestry. Different starting points along the CLV cline (in the east) combined with different local substrata populations (in the west) formed the contrasting genetic profiles of Serednii Stih and Usatove populations.
9. The Eneolithic population from Kartal included individuals well-described as mixtures of the Lower Volga Eneolithic (BPgroup) and Trypillians (Table SI2. 27). Thus, we have evidence for the westward migration of both unadmixed people from the Volga and those who mixed with European farmer-descended populations. Kartal also included individuals with ancestry from more "southern" parts of the CLV Cline such as Maykop or Remontnoye admixing with European farmers (Table SI2. 29). Westward migrations included people of "northern" Volga background (as in Csongrád and Giurgiuleşti) as well as of "southern" background (as in one of the Bursuceni individuals). In combination with European farmers these diverse CLV migrants formed diverse populations as in Kartal. At Mayaky in the Early Bronze Age, the Yamna migrants had absorbed some of the "southern" background ancestry; alternatively, Don Yamna acquired the "southern" (Maykop-related) ancestry in the east before migrating to Mayaky.

The ancestral landscape: a compendium

The key observation about the ancestral landscape of the people north and west of the Black Sea (in the North Pontic region and southeastern Europe) is their extreme diversity. Prior to the east-to-west

migrations associated with Caucasus-Lower Volga (CLV) cline people, the people of the region had ancestry from local Dnipro-Don hunter-gatherers and European farmers with various amounts of Balkan hunter-gatherer (represented by the Iron Gates population from Serbia) ancestry. The CLV migrations were responsible for the emergence of the Yamna (likely in the Dnipro-Don region as part of the "Dnipro cline") and they were followed by the spread of the Yamna itself.

In Table 1 and Extended Data Table 4 we summarize the analysis of this note presenting a "best guess" model for each of the populations we discussed.

Appendix I: Co-modeling populations with Dnipro-Don hunter-gatherer and European farmer ancestry

Populations of the Dnipro Cline can be modeled as mixtures of three sources: Aknashen-BPgroup (defining the CLV cline) and GK2 (as a representative of Dnipro-Don hunter-gatherers). However, we have seen that in the NPR admixture from European farmer descended populations (such as Trypillians) is important in the origins of many populations such as those at Mayaky and Kartal (Extended Data Table 4).

To jointly model both the Core Yamna (and other populations of the Dnipro Cline) and populations with Trypillian/European farmer ancestry, we would like to fit a 4-source model. However, when we fit this model to the Core Yamna or Usatove, i.e., populations with Dnipro-Don hunter-gatherer and European farmer ancestry respectively, we observe that its standard errors are quite high (Table SI2. 36).

Table SI2. 36. 4-source model with Right=Base. High standard errors necessitate the introduction of an outgroup population to Base that can differentiate between the four sources.

		Proportions				Std. errors			
**Test**	**P-value**	**Aknashen**	**BPgroup**	**GK2**	**Trypillia**	**Aknashen**	**BPgroup**	**GK2**	**Trypillia**
CoreYamna	0.892	18.6%	58.7%	21.2%	1.5%	16.3%	17.1%	12.1%	11.2%
Usatove	0.416	24.4%	25.8%	7.9%	41.8%	45.7%	49.3%	34.5%	31.0%
PeriPontic	0.965	22.4%	47.4%	26.9%	3.3%	15.2%	16.1%	11.1%	10.2%

Choosing additional Right populations

To reduce them we can add a new outgroup population to the Right set that can distinguish adequately between the four sources; however, if this outgroup population shares genetic drift with either the test population (e.g., Core Yamna) or the 4 sources in violation of the phylogenetic assumptions of qpAdm[2] it may result in the quintuple of populations (Core Yamna, Aknashen, BPgroup, GK2, Trypillians) being descended from five waves in relation to the Right set, i.e., the test population can no longer be modeled as the proposed 4-way admixture.

Thus, we want to find a phylogenetically informative outgroup to add to the Right set that both reduces the standard errors of the modeling (allowing us to estimate admixture proportions more accurately), but also does not violate the phylogenetic assumptions.

Moreover, this sought-after outgroup must be useful to reduce standard errors for both Core Yamna and Usatove and indeed among a variety of other populations that we wish to model. In order not to show preference for any of the targets, we combine all populations that can be feasibly modeled with the 3-source model, as well as those which also have Trypillian ancestry (Extended Data Table 4) into a "PeriPontic" (circum-Black Sea) meta-population:

"PeriPontic": BOY_EBA, Bulgaria_EBA_Yamna, CoreYamna, GK1, Igren_o, KTL_A, Maykop, MAJ, MAJ_EBA, Moldova_Crasnoe_Eneolithic, Moldova_EBA_Yamna, Moldova_EBA_Yamna_o, Moldova_GlobularAmphora_Yamna, Mykhailivka_I32534, PVgroup, Remontnoye, Russia_Don_EBA_Yamna, SShi, SSlo, SSmed, SS_ukr104, Ukraine_EBA_Catacomb, Ukraine_EBA_Ozera_I1917, Ukraine_EBA_Yamna, Ukraine_Oleksandria_MBA, Ukraine_Vasilevka, Usatove, Usatove_EBA, Usatove_Yamna, USV, Zhivotilovka_I17974

Individuals of the "PeriPontic" population are quite diverse but they should all have (in our reconstruction) ancestry from the four sources or their subset. We confirm this by fitting the 4-source model on the PeriPontic meta-population whose overall admixture proportions reflect an average across its many diverse sub-populations (Table SI2. 36).

In order to find such an outgroup we tried all population labels in our dataset not included in PeriPontic, the four sources or Base, plotting P-values and standard errors in Figure SI2. 3. We see that several outgroups give us leverage to estimate proportions accurately while remaining feasible. We show the top-20 (lowest standard error) models in Table SI2. 37.

Choosing which outgroup to add is a subjective decision; ideally, we would like to avoid outgroups that are "too close" to the modeled and source populations. Such populations may be rejected if they violate the phylogenetic assumptions. For example, the considered outgroup "China_Xinjiang_Ayituohan_Afanasievo_BA.SG"[41] does so (p=2e-5), as it is a Yamna-Afanasievo related population from the eastern edge of the distribution of this genetic cluster and obviously shares post admixture genetic drift with "PeriPontic" which includes many Yamna individuals. Other violations may not be detected by a low p-value (which depends on data quality, and the amount of shared genetic drift) but will bias admixture inferences subtly.

Thus, we should also avoid populations whose estimates are far off from the "consensus" across the different choices. We decided to use the sum of the squared differences of inferred admixture proportions for each outgroup choice from the median (across all 20 outgroups) as a measure of proximity to the consensus (Table SI2. 37). This measure identifies the Tarim_EMBA1 population from Xinjiang, China as the outgroup of choice. Standard errors are minimized for another population from the same region (China_Xinjiang_Xiaohe_BA.SG). Both these populations make good outgroups because of their distance to our region of interest and their description of possessing late "Ancient North Eurasian" (ANE) ancestry unrelated to the steppe migrations (of Afanasievo-related groups).[41]

We were curious about why these two outgroups could differentiate between the sources, so we estimated statistics of the form f_4(Added Outgroup, OldAfrica; Source_1, Source_2). These indicate (Table SI2. 38) that they do so by strongly differentiating steppe sources (GK2 and BPgroup) from farmer ones (Aknashen and Trypillians).

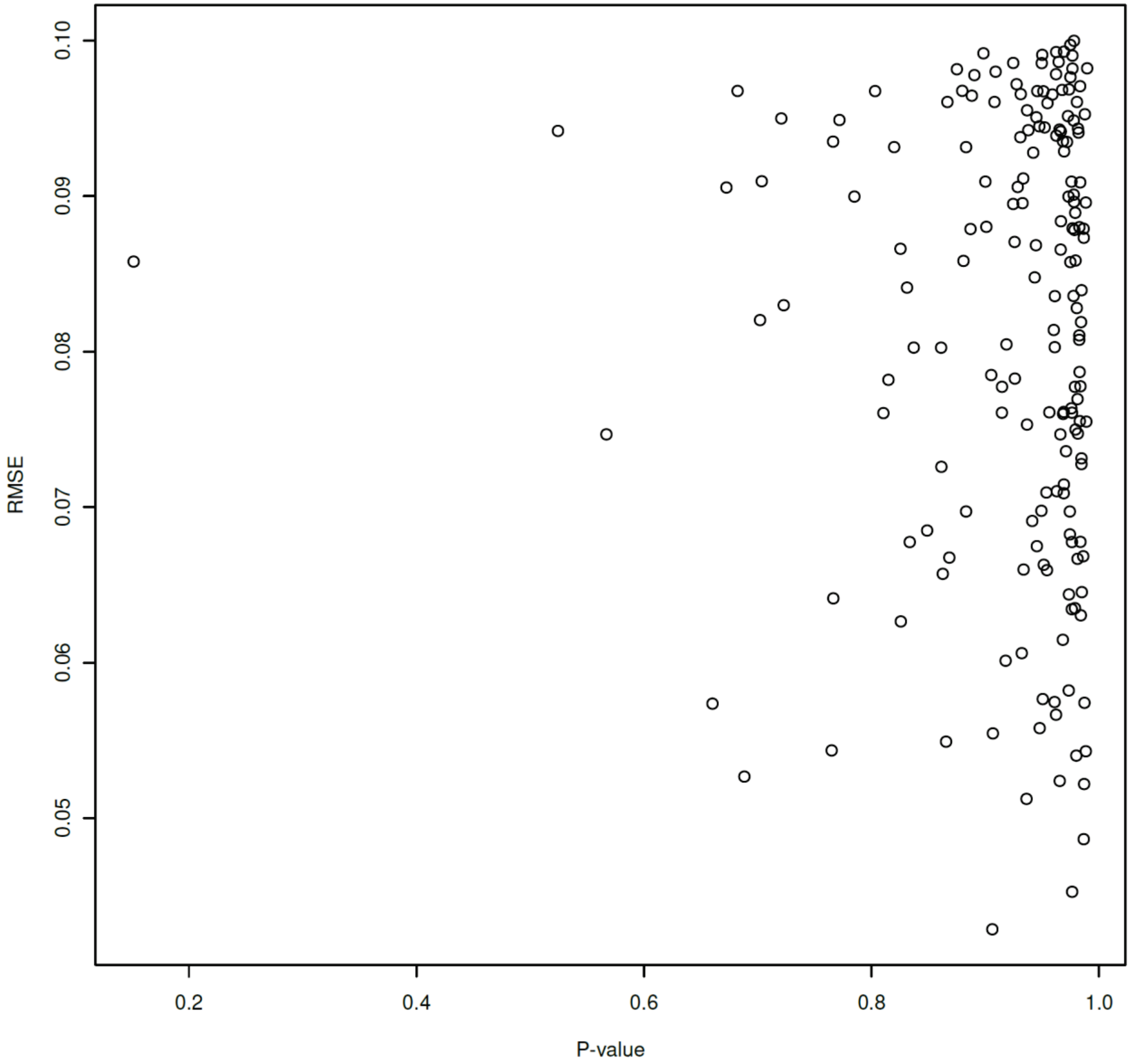

Figure SI2. 3. P-value and Root Mean Square Error (RMSE) of 4-way model (Aknashen, BPgroup, GK2, Trypillia) when adding diverse outgroups to the Base set. Models with <10% RMSE are shown.

Table SI2. 37. Adding outgroups to Base set. We model the "PeriPontic" meta-population with diverse added outgroups to Base. Top-20 models of lowest RMSE are shown. Proximity to median is the sum of the squared difference between admixture proportions for each added outgroup to the median proportions across all 20 outgroups. The two chosen outgroups for addition are highlighted in bold and minimize the RMSE (China_Xinjiang_Xiaohe_BA.SG) and proximity to median (Tarim_EMBA1).

		Proportions				Std. errors				RMSE	Proximity to Median
**Added outgroup**	**P-value**	**Aknashen**	**BPgroup**	**GK2**	**Trypillia**	**Aknashen**	**BPgroup**	**GK2**	**Trypillia**		
**Tarim_EMBA1**	**0.962**	**19.5%**	**50.4%**	**25.0%**	**5.1%**	**6.3%**	**6.6%**	**4.9%**	**4.6%**	**5.7%**	**3.8E-06**
Russia_Khvalynsk_Eneolithic_brother.I6734	0.973	19.8%	50.2%	25.0%	4.9%	6.5%	6.8%	5.0%	4.7%	5.8%	5.8E-06
Ukraine_Deriivka_Mes	0.948	20.2%	50.0%	24.9%	5.0%	6.6%	6.5%	4.4%	4.4%	5.6%	4.0E-05
Usatove_I20078	0.965	19.2%	50.9%	24.6%	5.3%	6.0%	6.0%	4.4%	4.3%	5.2%	7.5E-05
WHG	0.961	19.5%	50.8%	24.3%	5.4%	6.8%	6.6%	4.5%	4.7%	5.7%	8.2E-05
Jordan_EBA	0.936	18.8%	51.0%	24.5%	5.7%	5.7%	6.1%	4.4%	4.0%	5.1%	1.8E-04
China_Xinjiang_Beifang_Xiaohe_BA.SG	0.907	18.6%	51.6%	24.0%	5.7%	6.2%	6.5%	4.7%	4.5%	5.5%	4.1E-04
Serbia_IronGates_Mesolithic	0.951	21.0%	49.1%	25.6%	4.3%	6.9%	6.7%	4.4%	4.6%	5.8%	4.2E-04
Germany_Blatterhohle_MN	0.918	18.3%	52.0%	23.7%	6.0%	7.0%	6.9%	4.9%	4.9%	6.0%	7.2E-04
Csongrád_I5124	0.987	21.4%	48.4%	26.3%	3.9%	6.5%	6.6%	4.9%	4.7%	5.7%	9.8E-04
Latvia_HG	0.866	18.0%	52.7%	22.8%	6.6%	6.6%	6.4%	4.1%	4.4%	5.5%	1.6E-03
**China_Xinjiang_Xiaohe_BA.SG**	**0.906**	**17.4%**	**52.8%**	**23.3%**	**6.6%**	**4.7%**	**4.9%**	**3.8%**	**3.6%**	**4.3%**	**1.6E-03**
TUR_E_Arslantepe_ChL	0.765	22.0%	47.8%	26.7%	3.6%	5.9%	6.6%	4.8%	4.1%	5.4%	1.7E-03
Russia_Khvalynsk_Eneolithic_brother.I6739	0.980	22.1%	47.7%	26.7%	3.5%	6.0%	6.2%	4.7%	4.5%	5.4%	1.8E-03
Kazakhstan_Kumsay_EBA	0.987	22.2%	47.6%	26.7%	3.4%	5.4%	5.5%	4.3%	4.1%	4.9%	2.0E-03
Russia_Steppe_Maykop	0.987	22.7%	47.2%	27.1%	3.1%	5.7%	6.0%	4.6%	4.4%	5.2%	2.7E-03
Russia_CaspianInland_EBA_Yamna_Yamna_o1	0.988	22.8%	47.0%	27.2%	3.0%	6.1%	6.2%	4.7%	4.5%	5.4%	3.0E-03
Khi	0.976	23.6%	46.2%	27.7%	2.5%	5.0%	5.0%	4.0%	4.0%	4.5%	4.6E-03
Serbia_IronGates	0.688	16.1%	54.8%	21.2%	7.9%	6.4%	6.1%	3.9%	4.2%	5.3%	5.5E-03
TUR_E_Arslantepe_ChL_brother.ART014	0.660	14.9%	54.9%	21.8%	8.4%	6.3%	6.9%	5.0%	4.4%	5.7%	6.5E-03
**Maximum difference**		8.7%	8.7%	6.5%	5.9%						

Table SI2. 38. Statistics showing how added Right outgroup populations differentiate between the populations of the 4-source model.

Added outgroup	$Source_1$	$Source_2$	f4(Added outgroup, OldAfrica, $Source_1$, $Source_2$)	Z-score
Tarim_EMBA1	Armenia_Aknashen_N	BPgroup	-0.009261	-20.6
Tarim_EMBA1	Armenia_Aknashen_N	GK2	-0.010226	-17.2
Tarim_EMBA1	Armenia_Aknashen_N	Trypillia	0.000128	0.3
Tarim_EMBA1	BPgroup	GK2	-0.001062	-2.3
Tarim_EMBA1	BPgroup	Trypillia	0.009411	33.4
Tarim_EMBA1	GK2	Trypillia	0.010428	23.1
China_Xinjiang_Xiaohe_BA.SG	Armenia_Aknashen_N	BPgroup	-0.009354	-21.0
China_Xinjiang_Xiaohe_BA.SG	Armenia_Aknashen_N	GK2	-0.009771	-16.5
China_Xinjiang_Xiaohe_BA.SG	Armenia_Aknashen_N	Trypillia	0.000043	0.1
China_Xinjiang_Xiaohe_BA.SG	BPgroup	GK2	-0.000284	-0.6
China_Xinjiang_Xiaohe_BA.SG	BPgroup	Trypillia	0.009456	33.5
China_Xinjiang_Xiaohe_BA.SG	GK2	Trypillia	0.009712	21.3

Modeling Peri-Pontic populations

We modeled all Peri-Pontic sub-populations using the original Base set, augmenting it with either of the two identified outgroup populations, or with both (Table SI2. 39).

Table SI2. 39. 4-source modeling of Peri-Pontic populations with Trypillian ancestry as the 4th source.

			Proportions				Std. errors				
**Test**	**Outgroup set**	**P-value**	**Aknashen**	**BPgroup**	**GK2**	**Trypillia**	**Aknashen**	**BPgroup**	**GK2**	**Trypillia**	**RMSE**
BOY_EBA	Base	0.596	22.0%	46.6%	25.7%	5.7%	39.1%	42.5%	30.3%	26.9%	35.3%
BOY_EBA	+Tarim_EMBA1	0.717	24.7%	43.9%	27.5%	3.9%	13.5%	14.5%	11.0%	10.0%	12.4%
BOY_EBA	+Xiaohe_BA.SG	0.763	23.9%	44.7%	26.9%	4.4%	8.7%	9.0%	6.9%	6.7%	7.9%

BOY_EBA	+both	0.833	24.1%	44.5%	27.0%	4.4%	8.7%	9.0%	7.0%	6.6%	7.9%
Bulgaria_EBA_Yamna	Base	0.828	25.0%	40.0%	23.2%	11.8%	34.8%	36.9%	26.1%	24.1%	31.0%
Bulgaria_EBA_Yamna	+Tarim_EMBA1	0.931	21.5%	44.0%	20.5%	14.0%	12.0%	12.6%	9.4%	8.9%	10.8%
Bulgaria_EBA_Yamna	+Xiaohe_BA.SG	0.810	14.8%	50.9%	15.9%	18.3%	9.2%	9.5%	7.3%	7.0%	8.3%
Bulgaria_EBA_Yamna	+both	0.817	14.8%	51.1%	15.5%	18.6%	13.0%	13.5%	10.5%	9.9%	11.8%
CoreYamna	Base	0.892	19.3%	58.0%	21.7%	1.0%	12.9%	13.7%	9.6%	8.7%	11.4%
CoreYamna	+Tarim_EMBA1	0.892	15.8%	61.7%	19.3%	3.2%	6.5%	6.8%	5.1%	4.8%	5.9%
CoreYamna	+Xiaohe_BA.SG	0.861	13.9%	63.8%	17.8%	4.5%	5.1%	5.2%	4.0%	3.8%	4.6%
CoreYamna	+both	0.884	14.0%	63.7%	17.9%	4.5%	5.2%	5.4%	4.1%	3.9%	4.7%
GK1	Base	0.814	30.9%	8.3%	79.5%	-18.7%	132.5%	138.0%	96.0%	90.6%	116.2%
GK1	+Tarim_EMBA1	0.895	27.2%	12.6%	76.7%	-16.5%	46.3%	50.0%	37.9%	34.2%	42.6%
GK1	+Xiaohe_BA.SG	0.871	18.6%	21.3%	70.9%	-10.8%	15.8%	16.6%	12.6%	11.9%	14.4%
GK1	+both	0.806	19.6%	20.5%	71.1%	-11.2%	16.3%	17.5%	13.3%	12.1%	15.0%
Igren_o	Base	0.644	38.7%	-34.9%	120.0%	-23.9%	1545.3%	1538.0%	1058.0%	1064.1%	1323.4%
Igren_o	+Tarim_EMBA1	0.659	13.1%	-10.5%	102.7%	-5.3%	43.8%	45.5%	34.2%	32.6%	39.4%
Igren_o	+Xiaohe_BA.SG	0.820	-12.4%	16.7%	84.8%	10.9%	26.8%	27.9%	21.7%	20.6%	24.5%
Igren_o	+both	0.539	-9.9%	14.2%	85.4%	10.4%	46.7%	48.4%	37.6%	35.8%	42.5%
KTL_A	Base	0.526	8.1%	43.5%	8.4%	40.0%	126.9%	131.1%	88.9%	84.7%	110.0%
KTL_A	+Tarim_EMBA1	0.446	18.3%	32.8%	15.6%	33.4%	20.3%	21.6%	16.0%	14.7%	18.4%
KTL_A	+Xiaohe_BA.SG	0.344	26.5%	24.3%	21.4%	27.8%	9.3%	9.3%	7.1%	7.1%	8.3%
KTL_A	+both	0.313	27.1%	23.7%	22.2%	27.1%	9.4%	9.6%	7.4%	7.2%	8.5%
Maykop	Base	0.533	114.2%	-18.3%	23.3%	-19.2%	33.0%	34.8%	24.4%	22.6%	29.2%
Maykop	+Tarim_EMBA1	0.620	103.2%	-6.4%	15.7%	-12.5%	16.8%	16.9%	12.6%	12.7%	14.9%
Maykop	+Xiaohe_BA.SG	0.679	106.4%	-9.9%	17.7%	-14.1%	12.9%	13.0%	10.1%	10.2%	11.6%
Maykop	+both	0.688	107.8%	-11.0%	19.1%	-15.8%	14.5%	14.4%	11.1%	11.4%	12.9%
MAJ	Base	0.106	10.1%	37.5%	0.1%	52.3%	94.8%	99.0%	69.9%	65.8%	83.7%
MAJ	+Tarim_EMBA1	0.146	19.2%	27.8%	6.5%	46.5%	28.9%	30.5%	22.7%	21.1%	26.1%
MAJ	+Xiaohe_BA.SG	0.125	23.9%	22.9%	9.9%	43.3%	8.7%	8.7%	6.7%	6.7%	7.8%
MAJ	+both	0.216	22.6%	24.3%	9.0%	44.2%	18.1%	17.7%	13.1%	13.6%	15.8%
MAJ_EBA	Base	0.151	62.3%	2.2%	56.1%	-20.6%	383.5%	397.8%	279.1%	264.6%	336.6%
MAJ_EBA	+Tarim_EMBA1	0.004	24.0%	42.1%	30.5%	3.4%	124.1%	125.2%	91.7%	90.6%	109.2%
MAJ_EBA	+Xiaohe_BA.SG	0.015	21.1%	45.3%	27.4%	6.2%	22.9%	23.4%	17.7%	17.3%	20.5%
MAJ_EBA	+both	0.007	46.2%	17.4%	49.4%	-13.0%	892.4%	993.0%	764.2%	663.6%	837.7%
Moldova_Crasnoe_Eneolithic	Base	0.530	49.5%	26.3%	41.7%	-17.6%	330.5%	336.8%	237.8%	231.7%	288.5%
Moldova_Crasnoe_Eneolithic	+Tarim_EMBA1	0.266	17.4%	60.4%	18.8%	3.3%	40.7%	43.9%	31.8%	28.7%	36.8%
Moldova_Crasnoe_Eneolithic	+Xiaohe_BA.SG	0.582	25.8%	51.8%	24.1%	-1.7%	14.1%	14.2%	10.8%	10.7%	12.6%
Moldova_Crasnoe_Eneolithic	+both	0.213	32.2%	44.9%	30.0%	-7.1%	17.4%	17.6%	13.5%	13.3%	15.6%
Moldova_EBA_Yamna	Base	0.737	22.5%	49.4%	23.1%	5.0%	21.9%	23.3%	16.6%	15.2%	19.6%
Moldova_EBA_Yamna	+Tarim_EMBA1	0.838	17.4%	54.6%	19.7%	8.3%	8.7%	9.0%	6.7%	6.4%	7.8%
Moldova_EBA_Yamna	+Xiaohe_BA.SG	0.770	15.9%	56.4%	18.4%	9.4%	6.9%	7.0%	5.4%	5.3%	6.2%
Moldova_EBA_Yamna	+both	0.868	16.0%	56.1%	18.6%	9.3%	6.7%	6.8%	5.2%	5.1%	6.0%
Moldova_EBA_Yamna_o	Base	0.374	-3.0%	50.9%	3.2%	48.9%	1017.0%	1050.4%	730.9%	697.6%	888.6%
Moldova_EBA_Yamna_o	+Tarim_EMBA1	0.265	-2.2%	50.6%	4.2%	47.4%	42.9%	43.7%	32.7%	31.9%	38.2%
Moldova_EBA_Yamna_o	+Xiaohe_BA.SG	0.458	3.3%	44.4%	8.2%	44.1%	15.5%	15.5%	12.0%	12.0%	13.9%
Moldova_EBA_Yamna_o	+both	0.275	6.0%	41.7%	10.5%	41.8%	16.0%	16.2%	12.6%	12.4%	14.4%
Moldova_GlobularAmphora_Yamna	Base	0.647	64.0%	-2.5%	58.4%	-20.0%	50.8%	52.5%	36.8%	35.2%	44.5%
Moldova_GlobularAmphora_Yamna	+Tarim_EMBA1	0.035	37.0%	25.9%	41.5%	-4.3%	117.5%	130.9%	97.2%	83.9%	108.9%
Moldova_GlobularAmphora_Yamna	+Xiaohe_BA.SG	0.195	31.6%	31.8%	35.8%	0.7%	17.3%	17.9%	13.6%	13.1%	15.6%
Moldova_GlobularAmphora_Yamna	+both	0.056	39.9%	22.7%	43.6%	-6.2%	21.2%	21.7%	16.4%	15.8%	19.0%
Mykhailivka_I32534	Base	0.261	41.5%	42.6%	33.5%	-17.7%	268.0%	280.0%	195.6%	183.8%	235.7%
Mykhailivka_I32534	+Tarim_EMBA1	0.426	25.3%	59.3%	22.1%	-6.6%	73.2%	74.8%	54.3%	52.8%	64.6%
Mykhailivka_I32534	+Xiaohe_BA.SG	0.436	19.1%	65.4%	17.3%	-1.9%	30.0%	30.5%	23.7%	23.3%	27.1%
Mykhailivka_I32534	+both	0.576	21.1%	63.5%	18.3%	-2.9%	33.0%	33.4%	25.5%	25.0%	29.5%
PVgroup	Base	0.767	26.8%	75.9%	8.6%	-11.3%	33.8%	35.3%	24.4%	23.0%	29.6%
PVgroup	+Tarim_EMBA1	0.882	26.8%	76.0%	8.6%	-11.4%	13.1%	13.2%	9.7%	9.6%	11.5%
PVgroup	+Xiaohe_BA.SG	0.844	30.6%	72.0%	11.1%	-13.8%	10.1%	9.8%	7.4%	7.6%	8.8%
PVgroup	+both	0.849	31.9%	70.7%	12.2%	-14.8%	13.1%	12.8%	9.3%	9.6%	11.3%
Remontnoye	Base	0.480	29.8%	70.1%	-10.7%	10.7%	151.8%	169.8%	120.5%	102.5%	138.7%
Remontnoye	+Tarim_EMBA1	0.656	28.0%	72.3%	-12.1%	11.9%	14.8%	15.1%	11.1%	10.9%	13.1%
Remontnoye	+Xiaohe_BA.SG	0.621	34.4%	65.5%	-7.7%	7.8%	10.4%	10.7%	8.2%	7.9%	9.4%
Remontnoye	+both	0.573	36.5%	63.4%	-5.9%	6.0%	11.1%	11.4%	8.7%	8.4%	10.0%
Russia_Don_EBA_Yamna	Base	0.733	32.8%	23.3%	52.4%	-8.5%	20.9%	22.2%	15.5%	14.1%	18.5%
Russia_Don_EBA_Yamna	+Tarim_EMBA1	0.755	25.5%	31.1%	47.4%	-3.9%	9.8%	10.4%	7.8%	7.2%	8.9%
Russia_Don_EBA_Yamna	+Xiaohe_BA.SG	0.466	19.9%	37.1%	43.3%	-0.3%	7.8%	8.0%	6.1%	6.0%	7.0%
Russia_Don_EBA_Yamna	+both	0.484	19.9%	37.2%	43.1%	-0.2%	7.8%	8.0%	6.1%	5.8%	7.0%
SShi	Base	0.402	34.2%	33.6%	46.7%	-14.4%	90.0%	94.4%	64.0%	59.6%	78.5%
SShi	+Tarim_EMBA1	0.464	36.8%	30.7%	48.6%	-16.1%	15.8%	16.4%	12.1%	11.5%	14.1%
SShi	+Xiaohe_BA.SG	0.571	26.5%	41.8%	41.0%	-9.3%	10.9%	10.9%	8.3%	8.2%	9.7%
SShi	+both	0.279	27.3%	41.2%	41.0%	-9.5%	12.7%	12.8%	9.7%	9.6%	11.3%
SSlo	Base	0.283	108.2%	-88.7%	145.1%	-64.5%	324.6%	347.6%	241.4%	218.5%	288.2%
SSlo	+Tarim_EMBA1	0.409	83.1%	-62.4%	127.4%	-48.1%	37.9%	39.9%	29.9%	27.9%	34.3%
SSlo	+Xiaohe_BA.SG	0.312	57.1%	-34.3%	108.6%	-31.3%	24.0%	25.0%	19.5%	18.4%	21.9%
SSlo	+both	0.257	60.8%	-38.4%	110.9%	-33.2%	31.4%	33.1%	25.3%	23.7%	28.7%
SSmed	Base	0.864	36.5%	7.2%	70.2%	-13.9%	52.0%	55.6%	38.0%	34.5%	45.9%
SSmed	+Tarim_EMBA1	0.909	26.9%	17.6%	63.3%	-7.8%	15.3%	15.9%	11.8%	11.3%	13.7%
SSmed	+Xiaohe_BA.SG	0.764	18.1%	27.1%	56.9%	-2.0%	11.6%	12.1%	9.3%	8.8%	10.5%
SSmed	+both	0.675	17.2%	27.8%	55.9%	-1.0%	12.3%	12.8%	9.6%	9.2%	11.1%
SS_ukr104	Base	0.360	67.9%	-18.7%	85.5%	-34.7%	1199.9%	1296.9%	923.3%	826.7%	1079.1%
SS_ukr104	+Tarim_EMBA1	0.529	27.7%	25.5%	54.1%	-7.3%	25.7%	26.9%	20.1%	18.8%	23.1%
SS_ukr104	+Xiaohe_BA.SG	0.422	4.3%	50.5%	37.1%	8.1%	14.9%	15.6%	12.1%	11.4%	13.6%
SS_ukr104	+both	0.159	7.1%	47.6%	38.8%	6.5%	92.7%	99.4%	74.5%	67.7%	84.6%
Ukraine_EBA_Catacomb	Base	0.613	36.3%	32.4%	32.9%	-1.6%	329.0%	325.5%	229.3%	233.0%	283.3%
Ukraine_EBA_Catacomb	+Tarim_EMBA1	0.736	21.9%	47.2%	23.3%	7.6%	20.5%	21.4%	16.2%	15.3%	18.5%
Ukraine_EBA_Catacomb	+Xiaohe_BA.SG	0.770	26.0%	42.9%	25.9%	5.2%	14.5%	15.0%	11.7%	11.3%	13.2%

Ukraine_EBA_Catacomb	+both	0.740	30.4%	38.2%	29.9%	1.5%	15.6%	16.2%	12.4%	11.9%	14.2%
Ukraine_EBA_Ozera_I1917	Base	0.477	104.6%	-25.3%	54.7%	-33.9%	48.8%	51.2%	36.0%	33.6%	43.1%
Ukraine_EBA_Ozera_I1917	+Tarim_EMBA1	0.096	60.2%	21.8%	23.7%	-5.7%	545.7%	547.6%	390.5%	388.6%	474.6%
Ukraine_EBA_Ozera_I1917	+Xiaohe_BA.SG	0.043	54.4%	28.3%	18.6%	-1.2%	80.8%	80.6%	64.4%	64.6%	73.1%
Ukraine_EBA_Ozera_I1917	+both	0.076	57.3%	25.3%	21.1%	-3.7%	28.5%	30.3%	22.5%	20.9%	25.9%
Ukraine_EBA_Yamna	Base	0.494	22.7%	47.0%	24.9%	5.5%	60.8%	64.1%	44.3%	41.1%	53.5%
Ukraine_EBA_Yamna	+Tarim_EMBA1	0.539	17.5%	52.5%	21.4%	8.6%	29.3%	29.9%	21.9%	21.3%	25.9%
Ukraine_EBA_Yamna	+Xiaohe_BA.SG	0.337	8.5%	62.0%	14.8%	14.7%	8.7%	8.8%	6.7%	6.5%	7.8%
Ukraine_EBA_Yamna	+both	0.310	8.2%	62.5%	14.2%	15.1%	12.1%	12.3%	9.0%	8.8%	10.7%
Ukraine_Oleksandria_MBA	Base	0.666	9.8%	61.0%	25.1%	4.1%	95.2%	101.0%	69.4%	63.6%	83.9%
Ukraine_Oleksandria_MBA	+Tarim_EMBA1	0.666	-19.1%	91.7%	5.0%	22.4%	28.4%	28.6%	21.2%	21.0%	25.1%
Ukraine_Oleksandria_MBA	+Xiaohe_BA.SG	0.806	2.8%	68.6%	19.9%	8.7%	19.5%	19.3%	14.9%	15.1%	17.3%
Ukraine_Oleksandria_MBA	+both	0.429	2.4%	69.1%	20.4%	8.0%	23.6%	23.8%	18.5%	18.3%	21.2%
Ukraine_Vasilevka	Base	0.086	21.5%	-31.7%	129.2%	-19.0%	529.3%	543.6%	393.5%	379.2%	467.5%
Ukraine_Vasilevka	+Tarim_EMBA1	0.140	32.7%	-43.5%	137.2%	-26.4%	32.0%	33.6%	25.1%	23.4%	28.9%
Ukraine_Vasilevka	+Xiaohe_BA.SG	0.168	13.9%	-23.5%	123.7%	-14.0%	20.9%	21.9%	16.7%	15.7%	19.0%
Ukraine_Vasilevka	+both	0.090	14.0%	-23.2%	122.6%	-13.3%	21.5%	22.5%	17.0%	16.1%	19.5%
Usatove	Base	0.416	23.0%	27.4%	6.8%	42.9%	45.6%	44.6%	30.4%	31.3%	38.6%
Usatove	+Tarim_EMBA1	0.520	19.3%	31.2%	4.3%	45.3%	10.7%	11.0%	8.4%	8.1%	9.6%
Usatove	+Xiaohe_BA.SG	0.537	20.7%	29.7%	5.2%	44.3%	7.4%	7.4%	5.7%	5.8%	6.6%
Usatove	+both	0.613	20.7%	29.7%	5.4%	44.2%	7.3%	7.4%	5.8%	5.7%	6.6%
Usatove_EBA	Base	0.881	70.8%	0.5%	61.8%	-33.2%	93.5%	99.7%	68.8%	62.7%	82.7%
Usatove_EBA	+Tarim_EMBA1	0.944	63.8%	7.5%	57.0%	-28.4%	34.3%	35.9%	27.0%	25.6%	31.0%
Usatove_EBA	+Xiaohe_BA.SG	0.931	65.7%	6.0%	57.9%	-29.5%	24.1%	25.1%	19.9%	19.0%	22.2%
Usatove_EBA	+both	0.951	64.9%	6.4%	57.6%	-29.0%	25.9%	26.5%	20.8%	20.3%	23.5%
Usatove_Yamna	Base	0.842	53.3%	14.7%	49.9%	-17.9%	29.2%	30.8%	21.6%	20.0%	25.8%
Usatove_Yamna	+Tarim_EMBA1	0.815	62.2%	5.0%	56.5%	-23.8%	20.9%	21.6%	16.0%	15.3%	18.7%
Usatove_Yamna	+Xiaohe_BA.SG	0.874	38.7%	30.4%	39.5%	-8.6%	14.4%	14.6%	11.1%	10.9%	12.9%
Usatove_Yamna	+both	0.078	42.1%	26.3%	41.4%	-9.8%	25.3%	25.3%	18.9%	18.8%	22.3%
USV	Base	0.021	-5.1%	60.8%	-16.6%	60.9%	1757.8%	2004.7%	1390.5%	1144.5%	1608.7%
USV	+Tarim_EMBA1	0.044	22.3%	29.3%	7.5%	40.9%	25.3%	26.1%	19.5%	18.7%	22.6%
USV	+Xiaohe_BA.SG	0.041	31.3%	20.0%	13.9%	34.8%	13.0%	13.1%	10.1%	10.0%	11.6%
USV	+both	0.047	31.3%	20.0%	14.3%	34.4%	11.5%	11.5%	8.8%	8.8%	10.2%
Zhivotilovka_I17974	Base	0.133	38.1%	41.5%	38.6%	-18.1%	345.6%	374.6%	259.5%	230.5%	308.3%
Zhivotilovka_I17974	+Tarim_EMBA1	0.055	-12.3%	94.6%	3.0%	14.7%	90.2%	94.0%	71.2%	67.3%	81.5%
Zhivotilovka_I17974	+Xiaohe_BA.SG	0.099	-5.9%	88.2%	7.1%	10.7%	18.3%	18.5%	13.5%	13.3%	16.1%
Zhivotilovka_I17974	+both	0.064	-2.7%	84.6%	10.0%	8.1%	19.1%	19.8%	14.7%	14.0%	17.1%

We plot the admixture proportions estimated using the Xiaohe BA outgroup (which have the lowest standard errors) in Extended Data Fig. 1 for the populations in which the RMSE is ≤10%. By jointly modeling them we can compare them using the same 4-source model which agrees quantitatively with the results of the simpler 2- and 3-source models derived in this note and in ref.[3]. The three major groups apparent in our modeling are the CLV populations (Remontnoye and PVgroup) characterized by high BPgroup/Aknashen proportions; the Dnipro Cline populations (including several Yamna subsets) characterized by high but dampened amounts of the CLV cline ancestral sources complemented by substantial GK2-related ancestry; and the populations related to Usatove (including also MAJ and KTL_A) in which the Trypillian ancestry is highest.

We were concerned by the fact that KTL_A is shown in Extended Data Figure 1 have both GK2 and Aknashen-related ancestry while it fits the much simpler 2-source BPgroup+Trypillia model as we discussed above. Given the high standard errors of this population we do not think there is strong evidence for this ancestry (Table SI2. 39). Additionally, as we previously examined the BPgroup+Trypillia+(GK2 or Aknashen) 3-source models using the Base outgroup set and find that in both the amount of the 3rd source (GK2 or Aknashen) is low and non-significant (Table SI2. 27). Thus, we think that the reconstruction of the ancestry of this population as a 2-source mixture of BPgroup and Trypillians reflects its most likely history.

Appendix II: Co-modeling populations with Dnipro-Don hunter-gatherer and Steppe Maykop ancestry

We also wanted to co-model the two individuals with Steppe Maykop ancestry (Extended Data Table 4) and thus add Steppe Maykop as a 4th source to the Aknashen-BPgroup-GK2 model. We repeat the analysis of Table SI2. 39 for the set of populations that fit the 3-source model and do not have farmer ancestry and the two individuals with Steppe Maykop ancestry:

"PeriPontic1": CoreYamna, GK1, Igren_o, Maykop, MAJ_EBA, Moldova_Crasnoe_Eneolithic, PVgroup, Remontnoye, Russia_Don_EBA_Yamna, SShi, SSlo, SSmed, SS_ukr104, Ukraine_EBA_Catacomb, Ukraine_EBA_Ozera_I1917, Usatove_I20078, Usatove_Yamna, Zhivotilovka_I17974

Admixture with Steppe Maykop as the 4th source are shown in Table SI2. 40 and visualized in Extended Data Fig. 2 for populations with RMSE ≤10%. The Steppe Maykop ancestry is significantly higher than zero for Usatove_I20078 even with the Base set of outgroups (55.6±5.3%), similar to the 60.6±6.2% estimated for the simpler Core Yamna + Steppe Maykop model for this individual. For Zhivotilovka_I17974 the Steppe Maykop ancestry is 18.2±6.0% for the Core Yamna + Steppe Maykop model; its nominal value is ~17-18% in Table SI2. 40 but with a higher standard error. As we argued above, this individual too is likely to have Steppe Maykop-related ancestry, but the statistical evidence for it is weaker than is the case for Usatove_I20078.

Table SI2. 40. 4-source modeling of Peri-Pontic populations with Steppe Maykop ancestry as the 4th source.

			Proportions				Std. errors				
Test	Outgroup set	P-value	Aknashen	BPgroup	GK2	Steppe Maykop	Aknashen	BPgroup	GK2	Steppe Maykop	RMSE
CoreYamna	Base	0.851	20.4%	57.6%	22.8%	-0.8%	2.0%	6.4%	1.8%	5.2%	4.3%
CoreYamna	+Tarim_EMBA1	0.826	20.7%	55.1%	22.4%	1.8%	1.9%	5.4%	1.7%	3.7%	3.5%
CoreYamna	+Xiaohe_BA.SG	0.710	20.9%	53.9%	21.9%	3.2%	1.9%	5.7%	1.6%	3.9%	3.7%
CoreYamna	+both	0.729	20.7%	55.0%	22.0%	2.3%	1.9%	5.6%	1.7%	3.7%	3.6%
GK1	Base	0.774	1.0%	47.7%	61.4%	-10.0%	5.0%	16.5%	4.8%	13.6%	11.2%
GK1	+Tarim_EMBA1	0.869	1.0%	46.8%	61.0%	-8.8%	4.7%	13.7%	4.5%	9.6%	9.0%
GK1	+Xiaohe_BA.SG	0.847	1.4%	44.4%	60.9%	-6.6%	4.8%	13.7%	4.3%	9.5%	8.9%
GK1	+both	0.851	0.8%	46.6%	60.7%	-8.0%	4.7%	13.5%	4.5%	9.1%	8.8%
Igren_o	Base	0.765	1.4%	1.6%	98.0%	-1.0%	10.0%	34.0%	9.3%	28.1%	23.1%
Igren_o	+Tarim_EMBA1	0.839	-0.2%	10.3%	99.4%	-9.6%	9.4%	26.9%	8.9%	18.3%	17.5%
Igren_o	+Xiaohe_BA.SG	0.854	1.9%	-4.5%	96.5%	6.0%	10.1%	29.4%	8.5%	20.0%	19.0%
Igren_o	+both	0.581	-0.9%	7.7%	97.0%	-3.8%	9.5%	27.0%	8.4%	18.2%	17.5%
Maykop	Base	0.316	87.0%	9.4%	4.1%	-0.6%	5.2%	16.7%	4.3%	12.7%	11.0%
Maykop	+Tarim_EMBA1	0.434	86.6%	10.4%	4.1%	-1.1%	5.0%	14.0%	4.0%	8.4%	8.8%
Maykop	+Xiaohe_BA.SG	0.374	86.2%	14.6%	5.2%	-6.1%	5.1%	14.8%	3.9%	8.9%	9.2%
Maykop	+both	0.267	86.8%	10.9%	5.3%	-3.0%	5.0%	14.4%	3.9%	8.7%	9.0%
MAJ_EBA	Base	0.130	35.2%	20.7%	33.5%	10.6%	4.7%	15.5%	3.9%	12.3%	10.4%
MAJ_EBA	+Tarim_EMBA1	0.107	36.9%	9.9%	32.1%	21.1%	4.4%	12.2%	3.5%	8.0%	7.8%
MAJ_EBA	+Xiaohe_BA.SG	0.147	36.5%	12.7%	32.2%	18.6%	4.4%	12.4%	3.4%	8.1%	7.9%
MAJ_EBA	+both	0.149	36.9%	10.2%	32.4%	20.5%	4.4%	12.0%	3.6%	7.8%	7.7%
Moldova_Crasnoe_Eneolithic	Base	0.627	21.1%	65.1%	24.6%	-10.7%	5.1%	16.8%	4.8%	13.4%	11.3%
Moldova_Crasnoe_Eneolithic	+Tarim_EMBA1	0.250	23.0%	50.7%	21.8%	4.5%	5.2%	15.4%	4.5%	10.3%	9.9%
Moldova_Crasnoe_Eneolithic	+Xiaohe_BA.SG	0.575	21.8%	58.6%	22.5%	-2.9%	4.9%	14.5%	4.2%	9.5%	9.2%
Moldova_Crasnoe_Eneolithic	+both	0.180	22.9%	52.5%	23.2%	1.4%	5.3%	16.0%	4.2%	10.4%	10.1%
PVgroup	Base	0.644	9.9%	94.2%	-2.6%	-1.5%	3.9%	13.2%	3.2%	10.0%	8.7%
PVgroup	+Tarim_EMBA1	0.734	9.2%	97.8%	-2.1%	-4.8%	3.7%	11.2%	3.2%	7.3%	7.1%
PVgroup	+Xiaohe_BA.SG	0.568	8.7%	101.7%	-1.3%	-9.1%	3.8%	11.7%	3.0%	7.6%	7.4%
PVgroup	+both	0.511	9.0%	99.3%	-1.0%	-7.3%	3.5%	10.4%	2.9%	6.8%	6.6%
Remontnoye	Base	0.402	46.6%	47.0%	-0.9%	7.3%	4.3%	14.0%	3.6%	10.4%	9.2%
Remontnoye	+Tarim_EMBA1	0.566	46.6%	46.5%	-1.1%	8.0%	4.1%	11.6%	3.5%	7.2%	7.3%
Remontnoye	+Xiaohe_BA.SG	0.568	46.6%	47.8%	-0.7%	6.3%	4.1%	11.6%	3.3%	7.4%	7.4%
Remontnoye	+both	0.610	46.9%	46.2%	-0.6%	7.6%	3.9%	10.8%	3.3%	6.7%	6.8%
Russia_Don_EBA_Yamna	Base	0.684	20.0%	38.1%	43.7%	-1.7%	2.7%	9.0%	2.6%	7.2%	6.1%

Russia_Don_EBA_Yamna	+Tarim_EMBA1	0.772	20.2%	36.4%	43.3%	0.1%	2.5%	7.2%	2.4%	5.0%	4.7%
Russia_Don_EBA_Yamna	+Xiaohe_BA.SG	0.581	20.6%	33.7%	42.7%	2.9%	2.7%	7.8%	2.2%	5.3%	5.0%
Russia_Don_EBA_Yamna	+both	0.517	20.0%	36.4%	42.6%	1.0%	2.5%	7.4%	2.3%	5.0%	4.8%
SShi	Base	0.426	10.4%	66.3%	32.5%	-9.3%	3.5%	11.7%	3.2%	9.4%	7.9%
SShi	+Tarim_EMBA1	0.520	9.7%	69.8%	33.1%	-12.7%	3.5%	10.3%	3.1%	6.9%	6.6%
SShi	+Xiaohe_BA.SG	0.582	10.5%	65.8%	32.5%	-8.7%	3.4%	10.0%	2.8%	6.8%	6.4%
SShi	+both	0.444	9.5%	69.9%	32.3%	-11.8%	3.6%	10.5%	3.0%	6.9%	6.7%
SSlo	Base	0.385	1.8%	58.9%	83.2%	-43.9%	8.5%	29.8%	8.0%	24.6%	20.2%
SSlo	+Tarim_EMBA1	0.477	3.8%	48.1%	81.9%	-33.8%	7.6%	22.6%	7.3%	15.2%	14.6%
SSlo	+Xiaohe_BA.SG	0.347	4.4%	42.5%	80.3%	-27.2%	8.3%	24.5%	6.9%	15.9%	15.6%
SSlo	+both	0.382	4.0%	45.0%	80.1%	-29.1%	7.5%	21.9%	7.0%	14.3%	14.0%
SSmed	Base	0.814	14.4%	35.3%	56.6%	-6.3%	4.0%	13.4%	3.7%	11.0%	9.1%
SSmed	+Tarim_EMBA1	0.872	14.8%	32.7%	55.9%	-3.4%	3.9%	10.8%	3.3%	7.2%	7.0%
SSmed	+Xiaohe_BA.SG	0.736	15.4%	28.7%	55.0%	0.9%	3.7%	10.7%	3.2%	7.3%	6.9%
SSmed	+both	0.628	15.1%	30.6%	55.2%	-0.8%	3.7%	10.7%	3.2%	7.4%	6.9%
SS_ukr104	Base	0.394	16.4%	36.9%	45.9%	0.8%	5.8%	19.9%	5.2%	16.4%	13.5%
SS_ukr104	+Tarim_EMBA1	0.553	15.7%	40.6%	46.4%	-2.8%	5.1%	15.0%	4.7%	10.4%	9.8%
SS_ukr104	+Xiaohe_BA.SG	0.481	17.6%	28.4%	44.1%	9.9%	5.2%	15.0%	4.7%	10.0%	9.7%
SS_ukr104	+both	0.119	15.6%	36.6%	44.0%	3.8%	6.0%	17.5%	4.9%	11.5%	11.2%
Ukraine_EBA_Catacomb	Base	0.747	37.1%	19.2%	30.7%	13.0%	6.4%	22.0%	5.6%	17.1%	14.6%
Ukraine_EBA_Catacomb	+Tarim_EMBA1	0.848	36.0%	21.0%	31.0%	11.9%	6.0%	17.6%	5.3%	11.4%	11.2%
Ukraine_EBA_Catacomb	+Xiaohe_BA.SG	0.849	36.1%	23.1%	31.3%	9.5%	5.9%	17.5%	5.2%	10.8%	11.0%
Ukraine_EBA_Catacomb	+both	0.818	35.5%	23.3%	31.8%	9.3%	6.0%	17.3%	5.2%	10.8%	10.9%
Ukraine_EBA_Ozera_I1917	Base	0.128	54.2%	31.0%	20.8%	-6.0%	6.0%	19.9%	5.2%	15.2%	13.1%
Ukraine_EBA_Ozera_I1917	+Tarim_EMBA1	0.100	56.5%	16.2%	18.4%	8.9%	5.9%	17.3%	4.9%	10.7%	10.9%
Ukraine_EBA_Ozera_I1917	+Xiaohe_BA.SG	0.074	56.8%	14.6%	17.5%	11.0%	6.0%	17.3%	4.9%	10.6%	10.9%
Ukraine_EBA_Ozera_I1917	+both	0.118	56.5%	15.1%	17.5%	10.9%	5.8%	16.9%	4.7%	10.3%	10.6%
Usatove_I20078	Base	0.740	5.8%	36.6%	1.9%	55.6%	5.0%	16.8%	4.3%	13.5%	11.3%
Usatove_I20078	+Tarim_EMBA1	0.840	5.4%	40.0%	2.6%	52.1%	4.7%	13.8%	3.8%	9.3%	8.9%
Usatove_I20078	+Xiaohe_BA.SG	0.822	5.6%	38.9%	2.1%	53.4%	4.7%	13.6%	3.7%	9.2%	8.7%
Usatove_I20078	+both	0.861	5.3%	40.5%	2.3%	51.9%	4.6%	13.1%	3.6%	8.9%	8.4%
Usatove_Yamna	Base	0.705	28.2%	37.2%	31.3%	3.3%	5.2%	17.1%	4.4%	13.4%	11.4%
Usatove_Yamna	+Tarim_EMBA1	0.462	26.3%	48.9%	33.8%	-9.1%	5.4%	15.0%	4.2%	9.8%	9.6%
Usatove_Yamna	+Xiaohe_BA.SG	0.819	27.8%	39.4%	31.5%	1.4%	5.2%	14.4%	4.3%	9.4%	9.2%
Usatove_Yamna	+both	0.081	27.0%	44.3%	32.1%	-3.4%	5.8%	16.2%	4.6%	10.1%	10.2%
Zhivotilovka_I17974	Base	0.173	13.8%	57.0%	18.2%	11.1%	5.1%	16.6%	4.4%	12.7%	11.0%
Zhivotilovka_I17974	+Tarim_EMBA1	0.188	14.8%	48.7%	16.9%	19.5%	4.8%	13.7%	4.2%	8.6%	8.7%
Zhivotilovka_I17974	+Xiaohe_BA.SG	0.234	14.5%	51.3%	16.9%	17.3%	4.8%	14.1%	4.1%	9.2%	9.0%
Zhivotilovka_I17974	+both	0.255	15.1%	48.2%	17.3%	19.4%	5.0%	13.9%	4.2%	8.6%	8.8%

The models of Extended Data Figure 1 and Extended Data Figure 2 also increase our confidence in the fact that the Core Yamna was indeed formed by the type of admixture of CLV and Dnipro-Don people inferred in ref.[3]. The fourth source, whether Trypillian (from the west) or Steppe Maykop (from the east) contributes a non-significant amount of ancestry to this population (4.5±3.8% and 3.2±3.9%). We cannot really exclude that some such ancestry was present in the Core Yamna population, but the bulk of its ancestry was indeed made up of the postulated CLV-Dnipro/Don mixture.

The Dnipro-Don area did indeed see diverse admixtures in which the CLV-Dnipro/Don mixture was primary and represented the coming together of CLV people (themselves of mixed Aknashen/BPgroup ancestry) with local Dnipro-Don (GK2 or Ukraine_N-related) hunter-gatherers and in which (or through which) both European farmer ancestry (in and from the west) and Steppe Maykop ancestry (from the east) flowed, creating the diverse tapestry of ancestry characterizing its Eneolithic and Bronze Age populations.

Appendix III: Do the core Yamna have Trypillian ancestry?

The fact that Trypillian ancestry exists in many populations of the NPR raises the question if it might also exist in low levels in the Core Yamna. While all populations of the Dnipro cline (such as the Serednii Stih and Yamna) were modeled without such ancestry in ref.[3], as the result of admixture between Caucasus-Lower Volga people with Dnipro-Don hunter-gatherer descendants, it is possible that some such ancestry may exist in them. As a further test of the possible presence of Trypillian ancestry in the Core Yamna we

sought to increase power in the Base set by modifying it as follows, highlighting the newly introduced populations:

Base1: OldAfrica, CHG, Iran_GanjDareh_N, **Serbia_IronGates_Mesolithic, Tarim_EMBA1**, Russia_Sidelkino.SG, **Jordan_EBA**, Turkey_N, **Armenia_Aknashen_N, Trypillia**

This set includes more recent (and higher data quality) populations for both the WHG-related (Iron Gates instead of Villabruna) and Siberian-related (Tarim EMBA1 instead of Afontova Gora 3) ancestry, an additional EBA population from the Levant (Jordan_EBA) and both the Aknashen Neolithic and Trypillian populations. The Aknashen and Trypillia populations will, of course, not be used on the Right when they are included as sources on the left, but their inclusion there allows us to test 3-source models (with only one of the two populations including) for resilience when the other population is on the Right.

The inclusion of such more recent populations on the Base1 risks that they may have had opportunity to share common genetic history with either the sources or target population in violation of the qpAdm phylogenetic assumptions.[2] However, the hope is that they may be able to differentiate the sources more powerfully. This increased power can be assessed by examining whether the fitted model can be estimated with lower standard errors using Base1 vs. Base or the augmented Base sets with the addition of outgroup populations (Table SI2. 39); we will empirically see this below. But, at the outset, we can test whether we can reject 3 waves for the four sources (Aknashen, BPgroup, GK2, Trypillia) using qpWave. We can do so using both the conservative Base set (p=0.0012) but much more distinctly using the Base1 set (p=4e-15). Thus, a priori, before examining the fit of the model to the core Yamna we may think that the Base1 set does indeed provide more power.

The 4-source model remains feasible (p=0.915) with a significant amount of Aknashen (15.6±4.3%) but not Trypillian (3.2±3.1%) ancestry. When Trypillia is excluded as a source, the model still fits (p=0.230) with 21.8±1.4% Aknashen-related ancestry, showing that it is resilient to the presence of Trypillian on the Base1 set; conversely, when Aknashen is excluded as a source and Trypillia is included, then the model does not fit albeit weakly (p=0.010), suggesting that Trypillia is not resilient to the presence of a Caucasus Neolithic population. Examining the qpAdm output reveals that the Trypillia model underestimates (Z=-2.0) shared genetic drift with Jordan_EBA, a Levantine population without known steppe ancestry,[9,12] but with Caucasus-related ancestry suggesting that it may share common history with Aknashen that is not well-modeled when Aknashen is placed on the right set.

The standard error of Trypillian as a 4th source is somewhat improved (3.1% vs. 4.6% for the best choice in Table SI2. 39 and 8.7% for the Base set) but remains consistent with both some European farmer (Trypillian-related) ancestry and with a scenario in which no such ancestry is present. Trypillian-related ancestry in the Yamna is thus possible but unproven. However, using any of the augmented Base sets of Table SI2. 39 or the alternative Base1 set, the proportion of Aknashen-related ancestry remains significant (15.6±4.3% corresponds to Z=3.6 for the 4-source model, and the simpler 3-source model remains feasible with Trypillian on the right set), therefore the presence of some such ancestry must be considered certain.

Appendix IV: Source of hunter-gatherer ancestry in the core Yamna along the UNHG-GK2-EHG continuum

In ref.[3] we noted that populations of the Dnipro-Don cline (Serednii Stih and Yamna) had Ukraine Neolithic (UNHG)-related hunter-gatherer ancestry which could be modeled with either UNHG (from the Dnipro) or a subset of Golubaya Krinitsa (GK2) from the Middle Don. As GK2 itself could be modeled as having 2/3 UNHG and 1/3 EHG ancestry, regardless of the source of the hunter-gatherer ancestry (UNHG or GK2) it would include a substantial amount of UNHG ancestry as would be expected, indeed, from the distribution of the Serednii Stih culture in the Dnipro-Don area where the Core Yamna itself must have emerged.

In ref.[3], we modeled Dnipro-Don populations as mixtures of Core Yamna (one end of the Dnipro-Don cline) and both UNHG and GK2 ancestries. What of the origin of the Core Yamna itself, which forms one end of the Dnipro-Don cline? In ref.[3], we presented proximal models for the origin of that population (as a mixture of Remontnoye and SShi populations), as well as more distal models, including the Aknashen-Bpgroup-GK2 model just discussed which derives their ancestry from a mixture of Caucasus-Lower Volga (CLV) cline people (Aknashen+Bpgroup) with Dnipro-Don hunter-gatherers. We would, however, want to investigate whether GK2 itself suffices as a hunter-gatherer source for the Core Yamna or the source of that ancestry may be slightly different from it along the UNHG-GK2-EHG cline.

We sought to test the model Aknashen-Bpgroup-GK2-UNHG; while this model does fit the core Yamna ($p=0.862$), it can be estimated only with very high standard errors of 27.9±23.5% GK2 and -4.5±18.8% UNHG ancestry using the Base set. Using the Base1 set the model still fits ($p=0.181$) but standard errors are not improved (-12.7±78.9% UNHG ancestry!).

We thus attempt to find for the UNHG as a 4th source, in the same manner as we did for Trypillians as a 4th source, another outgroup which, when added to the Right might bring down standard errors. The top 20 populations with the smaller standard error are shown in Table SI2. 41. Not surprisingly, Ukrainian Neolithic populations and individuals from this study and ref.[16,20,28] provide most traction towards the goal of lowering standard errors. In addition, Iron Gates Mesolithic from Romania, as well as diverse members of the "Oberkassel Cluster" (WHG-related populations of central-northern Europe identified in ref.[20]), are informative. The standard errors remain fairly high although we do observe that in general the proportion of GK2 ancestry is higher than UNHG ancestry and the latter is often negative and occasionally positive by small amounts.

Table SI2. 41. Adding populations to Base set. We add populations to the Right and examine the standard errors. Top-20 populations with lower standard errors are shown.

		Proportions				Std. errors				RMSE
**Added outgroup**	**P-value**	**Aknashen**	**BPgroup**	**GK2**	**UNHG**	**Aknashen**	**BPgroup**	**GK2**	**UNHG**	
France_Mesolithic_1d.rel.PER503.SG	0.891	21.2%	55.6%	26.0%	-2.8%	2.1%	4.8%	12.1%	9.5%	8.1%
Spain_Canes.SG	0.889	21.5%	54.9%	28.4%	-4.8%	1.9%	4.5%	11.5%	9.1%	7.7%
Germany_Niedertiefenbach_Wartberg_LN.SG_brother.KH150622.SG.KH150620.SG	0.914	21.6%	54.8%	28.9%	-5.3%	2.1%	4.4%	11.3%	9.2%	7.7%
Russia_AfontovaGora2.SG	0.910	21.5%	55.0%	28.4%	-5.0%	2.0%	4.3%	11.3%	9.2%	7.7%
Russia_Ekaterinovka_Eneolithic_mother.I6068	0.877	21.4%	55.4%	26.1%	-3.0%	2.2%	4.3%	10.7%	8.9%	7.4%
MesoFr	0.887	21.4%	55.3%	27.2%	-3.9%	2.0%	4.5%	10.9%	8.5%	7.3%
Latvia_MN_o3	0.886	21.5%	55.4%	26.8%	-3.6%	2.1%	4.2%	10.6%	8.9%	7.3%
OberkasselCluster	0.788	20.9%	55.8%	24.7%	-1.4%	1.7%	4.3%	10.5%	7.9%	7.0%
ukr161	0.858	21.1%	55.6%	26.1%	-2.8%	1.8%	4.4%	10.5%	7.8%	7.0%
Romania_IronGates_Mesolithic.SG	0.889	21.5%	54.9%	28.3%	-4.8%	1.8%	4.3%	10.3%	7.9%	6.9%
VO	0.836	20.9%	56.0%	24.6%	-1.5%	1.6%	3.9%	9.9%	7.6%	6.6%
Romania_IronGates_Mesolithic	0.856	21.2%	55.6%	26.1%	-2.9%	1.7%	4.1%	9.8%	7.3%	6.5%
Ukraine_N_1d.rel.I4114	0.834	21.2%	55.6%	26.3%	-3.0%	1.8%	3.9%	9.6%	7.6%	6.5%
Ukraine_N_1d.rel.I5881	0.872	21.5%	55.0%	28.8%	-5.3%	1.7%	3.8%	8.5%	6.5%	5.7%
Ukraine_N_father.or.son.I3718	0.838	21.4%	55.2%	27.4%	-4.0%	1.7%	3.8%	8.2%	6.3%	5.6%
ukr162	0.580	20.2%	57.6%	19.1%	3.1%	1.5%	3.4%	8.1%	6.2%	5.4%
Ukraine_N_son.I1732	0.832	21.0%	56.0%	24.8%	-1.8%	1.6%	3.6%	7.6%	5.7%	5.1%
ukr113	0.452	19.9%	58.0%	17.2%	4.9%	1.4%	3.5%	7.3%	5.3%	4.9%
Ukraine_N_dup.I4112	0.803	20.7%	56.5%	22.7%	0.1%	1.4%	3.3%	6.9%	5.1%	4.6%
ukr117	0.875	21.7%	54.5%	30.5%	-6.8%	1.6%	3.4%	6.5%	5.0%	4.5%

Conversely, we can test the model Aknashen-BPgroup-GK2-EHG, i.e., placing the hunter-gatherer source on the other side of GK2 along the UNHG-GK2-EHG cline. This model *can* be estimated fairly accurately with just the Base set ($p=0.869$) with 21.5±3.8% GK2 and 1.3±4.3% Russia_Karelia ancestry. The nominally positive proportion of EHG ancestry is not significantly different from zero as was the nominally negative proportion of UNHG ancestry in the previously considered model. Thus, significant deviations from GK2 towards either the UNHG or EHG side are supported by the data.

Finally, an alternative approach to placing the hunter-gatherer ancestry is to fit the Aknashen-BPgroup-UNHG-EHG model (using Russia_Karelia as the EHG source), i.e., do not use GK2 source but infer the

position of the hunter-gatherer ancestry along the UNHG-EHG continuum. This model can be well fit and accurately estimated with just the Base set (p=0.759) with an estimated 13.0±1.9% UNHG and 8.4±2.7% EHG ancestry (thus the ratio of UNHG/(UNHG+EHG) is ~61%). GK2 itself can be fit as 66.6±4.6% UNHG and 33.4±4.6% EHG (p=0.556), or the aforementioned ~2/3 ratio. Thus, on the basis of this analysis it appears that GK2 is a reasonable approximation for the hunter-gatherer ancestry in the Core Yamna, although slight deviations to either the UNHG or EHG direction cannot be excluded from the data.

Appendix V: European farmer ancestry in the Ukraine Neolithic hunter-gatherers

We sought to investigate the origins of Ukraine_N (UNHG) for two reasons.

The first, is the observation that in PCA (Figure SI2. 1), the direction of the EHG-UNHG-GK2 cline does not, when further projected tend towards the hunter-gatherers of the Balkans (BHG of Serbia Iron Gates) but is rather shifted "downwards", or, towards populations with farmer ancestry in the context of that PCA.

The second observation is that when we modeled Ukraine_N in the same setup as other populations of this note we can find only a single 2-source model that works (p=0.576) with 72.5±2.9% GK2 and 27.5±2.9% BHG ancestry. If Ukraine_N was part of a broader cline between EHG and BHG then it is surprising that it cannot also be modeled as a mixture of these two sources. However, using either Lebyazhinka or Karelia as the EHG source, this is not possible (p<1e-9) and qpAdm output suggests that these models underestimate shared genetic drift with Turkey_N (Z<-3.5). This is consistent with the shift in PCA towards farmers.

We thus explored 3-source models using all the same Sources as for other populations of this note, excluding only GK2: as the GK2+BHG model is feasible, there are many other generalized models of the form GK2+BHG+X with ~0% of X ancestry that are also feasible and our aim is to understand the position of Ukraine_N in the broader west-east/BHG-EHG continuum.

The feasible models (Table SI2. 42) all include EHG-BHG sources (Lebyazhinka and Serbia Iron Gates), but they all include ~7-9% of farmer ancestry, with the source of this ancestry being unclear. Competition between the models of Table SI2. 42 was inconclusive. We note that p-values for the European sources are higher than for the two Caucasus ones (Aknashen and Maykop) and this cannot be attributed to lower power to reject models as European sources all have larger sample sizes than those from the Caucasus.

Table SI2. 42. 3-source models for Ukraine_N. All sources other than GK2 were considered. We manually tested the LBK+Lebyazhinka+Serbia Iron Gates and CHG+Lebyazhinka+Serbia Iron Gates models and include them in this table.

				Proportions			Std. errors		
A	B	C	P-value	A	B	C	A	B	C
Armenia_Aknashen_N	Lebyazhinka_HG	Serbia_IronGates_Mesolithic	0.128	7.9%	44.7%	47.4%	1.1%	1.7%	1.5%
GlobularAmphora	Lebyazhinka_HG	Serbia_IronGates_Mesolithic	0.133	8.8%	48.6%	42.5%	1.2%	1.6%	1.8%
Lebyazhinka_HG	Maykop	Serbia_IronGates_Mesolithic	0.069	44.4%	8.4%	47.2%	1.7%	1.1%	1.4%
Lebyazhinka_HG	Serbia_IronGates_Mesolithic	Trypillia	0.257	47.8%	44.0%	8.2%	1.7%	1.7%	1.2%
Lebyazhinka_HG	Serbia_IronGates_Mesolithic	YUN_CA	0.198	48.2%	45.0%	6.8%	1.6%	1.6%	1.0%
LBK	*Lebyazhinka_HG*	*Serbia_IronGates_Mesolithic*	*0.154*	*6.6%*	*48.7%*	*44.7%*	*1.0%*	*1.6%*	*1.7%*
CHG	*Lebyazhinka_HG*	*Serbia_IronGates_Mesolithic*	*2e-05*	*6.5%*	*45.6%*	*47.9%*	*1.2%*	*1.9%*	*1.5%*

Given the sampling of Ukraine_N in the Dnipro area in some proximity to European farmer communities to its west, we think it likely that this 6th millennium BCE population was most probably interacted with European rather than Caucasus Neolithic populations.

We wanted to investigate whether the inferred farmer ancestry in Ukraine_N could be driven by individuals within that population as opposed to being a general feature of the population. Thus, we fit a model that included farmer ancestry to all 35 individuals under the Ukraine_N label, as well as relatives not included in the Ukraine_N label and outliers. We show this model with LBK (which is temporally more plausible, albeit genetically quite similar, to the later farmer groups, and also has the largest sample size) as the farmer source in Extended Data Table 2.

It is clear from Extended Data Table 2 individuals from Ukraine_N population have European farmer ancestry in general. An affinity to "Anatolian Neolithic farmer (ANF)" (and hence to the similar European farmers) was mentioned in ref.[20] but was said to include also Balkan hunter-gatherers, Baltic hunter-gatherers and Scandinavian hunter-gatherers.

We noticed that individual I31730 (Mariupol; 5474-5236 calBCE) does not fit the 3-source model (p=0.0037) which underestimates its shared genetic drift with CHG (Z=-2.9). When we use CHG instead of a farmer group as a source for the Ukraine_N population as a whole (Table SI2. 42) the model fails (p=2e-5). Using CHG instead of a farmer group as a source for the particular individual I31730, results in a successful fit (p=0.093) with an estimated 7.4±2.8% CHG-related ancestry. We also applied this alternative model to all individuals of Extended Data Table 2 that do not fit the model with LBK as a source (bottom of Extended Data Table 2) and discovered that one other individual (I1738; Vovnyhy, 5475-5320 calBCE) could be modeled in such a way.

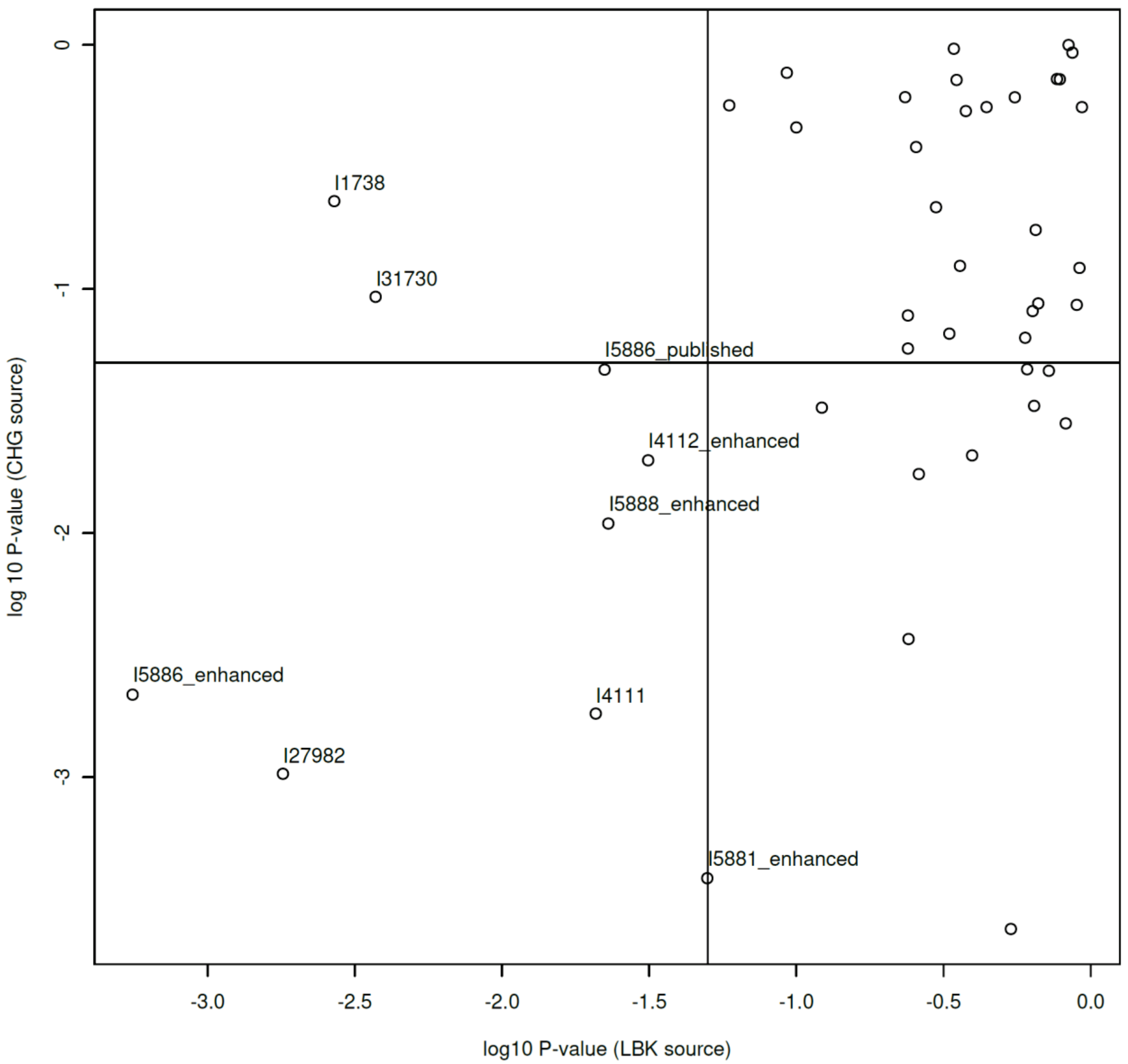

Figure SI2. 4. P-values for models with Serbia Iron Gates Mesolithic+Lebyazhinka HG + either LBK or CHG sources for individuals of Ukraine_N. p=0.05 vertical and horizontal lines are shown at log10(0.05)= -1.30103.

In Figure SI2. 4 we show the p-values for all individuals of Ukraine_N comparing the models with LBK or CHG as a source. The two individuals that fit the CHG model are shown in the upper left quadrant. Individuals of the lower left quadrant fit neither model. Many individuals (upper right quadrant) fit both models, but note that only the LBK model fits Ukraine_N as a whole, while the CHG one does not (Table SI2. 42), a behavior that is driven by individuals (lower right quadrant) that fit the LBK model but not the CHG one.

Thus, while we can conclude that low amounts of European farmer ancestry entered the UNHG population (from the western neighbors of the NPR hunter-gatherers), it is possible that for at least some of them there was CHG-related ancestry as well (from the east). Such ancestry was also detected in the GK1 subset at Golubaya Krinitsa Neolithic in the Middle Don and at the Krivyansky Eneolithic (ref.[3]) in the Lower Don and may have thus extended further west into the Dnipro region.

Modeling the ancestry of European hunter-gatherers as a whole is beyond the scope of this note, but we observe that the farmer ancestry in the Ukraine_N is inferred with Balkan hunter-gatherers as one of the sources and therefore exceeds any that may have existed in that population. As for the northern groups, neither has any discernible LBK ancestry (the likely population transmitting such ancestry to northern Europe) with neither Motala[2,11,36] (0.3±1.2%), Stora Förvar (1.0±1.3%), or Latvia_HG (-2.0±0.8%) having much evidence for it when the same model we applied to Ukraine_N is used (the true history for these northern populations probably involved native sources of WHG-related ancestry rather than the Balkan hunter-gatherers of the Iron Gates). As a control, we used the population from Ajvide in Sweden[42,43], a population of the Pitted Ware culture, which has a minority farmer ancestry component inferred to be 18.9±1.4% LBK ancestry, similar to the 22.1±1.1% inferred for another Pitted Ware population from Västerbjers.[44]

Thus, it appears that Ukraine Neolithic hunter-gatherers, similar to those of the Pitted Ware, incorporated some ancestry from their farmer neighbors while maintaining their overall hunter-gatherer genetic profile. Thus, while our investigation of the presence of European farmer ancestry in some populations like the core Yamna has failed to uncover some ancestry *on top of* the Ukraine Neolithic hunter-gatherer ancestry, some such ancestry may also have entered their population indirectly via populations with UNHG ancestry who had themselves European farmer ancestry.

References

1 Reich, D. *et al.* Reconstructing Native American population history. *Nature* **488**, 370-374, doi:10.1038/nature11258 (2012).
2 Haak, W. *et al.* Massive migration from the steppe was a source for Indo-European languages in Europe. *Nature* **522**, 207-211, doi:10.1038/nature14317 (2015).
3 Lazaridis, I., Patterson, N., Anthony, D. & others. The Genetic Origin of the Indo-Europeans. *in submission* (2024).
4 Skoglund, P. *et al.* Reconstructing Prehistoric African Population Structure. *Cell* **171**, 59-71.e21, doi:10.1016/j.cell.2017.08.049 (2017).
5 Wang, K. *et al.* Ancient genomes reveal complex patterns of population movement, interaction, and replacement in sub-Saharan Africa. *Science Advances* **6**, eaaz0183, doi:10.1126/sciadv.aaz0183.
6 Lipson, M. *et al.* Ancient DNA and deep population structure in sub-Saharan African foragers. *Nature* **603**, 290-296, doi:10.1038/s41586-022-04430-9 (2022).
7 Fu, Q. *et al.* The genetic history of Ice Age Europe. *Nature* **534**, 200-205, doi:10.1038/nature17993 (2016).
8 Jones, E. R. *et al.* Upper Palaeolithic genomes reveal deep roots of modern Eurasians. *Nat Commun* **6**, 8912, doi:10.1038/ncomms9912 (2015).
9 Lazaridis, I. *et al.* Genomic insights into the origin of farming in the ancient Near East. *Nature* **536**, 419-424, doi:10.1038/nature19310 (2016).
10 de Barros Damgaard, P. *et al.* The first horse herders and the impact of early Bronze Age steppe expansions into Asia. *Science* **360**, doi:10.1126/science.aar7711 (2018).
11 Mathieson, I. *et al.* Genome-wide patterns of selection in 230 ancient Eurasians. *Nature* **528**, 499-503, doi:10.1038/nature16152 (2015).
12 Lazaridis, I. *et al.* The genetic history of the Southern Arc: A bridge between West Asia and Europe. *Science* **377**, eabm4247, doi:10.1126/science.abm4247 (2022).
13 Wang, C.-C. *et al.* Ancient human genome-wide data from a 3000-year interval in the Caucasus corresponds with eco-geographic regions. *Nature Communications* **10**, 590, doi:10.1038/s41467-018-08220-8 (2019).
14 Allentoft, M. E. *et al.* Population genomics of Bronze Age Eurasia. *Nature* **522**, 167-172, doi:10.1038/nature14507 (2015).

15 Kumar, V. *et al.* Bronze and Iron Age population movements underlie Xinjiang population history. *Science* **376**, 62-69, doi:10.1126/science.abk1534 (2022).
16 Mathieson, I. *et al.* The genomic history of southeastern Europe. *Nature* **555**, 197-203, doi:10.1038/nature25778 (2018).
17 Narasimhan Vagheesh, M. *et al.* The formation of human populations in South and Central Asia. *Science* **365**, eaat7487, doi:10.1126/science.aat7487 (2019).
18 Patterson, N. *et al.* Large-scale migration into Britain during the Middle to Late Bronze Age. *Nature* **601**, 588-594, doi:10.1038/s41586-021-04287-4 (2022).
19 Mittnik, A. *et al.* Kinship-based social inequality in Bronze Age Europe. *Science* **366**, 731-734, doi:10.1126/science.aax6219 (2019).
20 Posth, C. *et al.* Palaeogenomics of Upper Palaeolithic to Neolithic European hunter-gatherers. *Nature* **615**, 117-126, doi:10.1038/s41586-023-05726-0 (2023).
21 Gelabert, P. *et al.* Genomes from Verteba cave suggest diversity within the Trypillians in Ukraine. *Scientific Reports* **12**, 7242, doi:10.1038/s41598-022-11117-8 (2022).
22 Penske, S. *et al.* Early contact between late farming and pastoralist societies in southeastern Europe. *Nature* **620**, 358-365, doi:10.1038/s41586-023-06334-8 (2023).
23 Patterson, N., Price, A. L. & Reich, D. Population Structure and Eigenanalysis. *PLOS Genetics* **2**, e190, doi:10.1371/journal.pgen.0020190 (2006).
24 Shinde, V. *et al.* An Ancient Harappan Genome Lacks Ancestry from Steppe Pastoralists or Iranian Farmers. *Cell* **179**, doi:10.1016/j.cell.2019.08.048 (2019).
25 Harney, É. *et al.* Ancient DNA from Chalcolithic Israel reveals the role of population mixture in cultural transformation. *Nature Communications* **9**, 3336, doi:10.1038/s41467-018-05649-9 (2018).
26 Lipson, M. *et al.* Parallel palaeogenomic transects reveal complex genetic history of early European farmers. *Nature* **551**, 368-372, doi:10.1038/nature24476 (2017).
27 Rivollat, M. *et al.* Ancient genome-wide DNA from France highlights the complexity of interactions between Mesolithic hunter-gatherers and Neolithic farmers. *Science Advances* **6**, eaaz5344, doi:10.1126/sciadv.aaz5344 (2020).
28 Mattila, T. M. *et al.* Genetic continuity, isolation, and gene flow in Stone Age Central and Eastern Europe. *Communications Biology* **6**, 793, doi:10.1038/s42003-023-05131-3 (2023).
29 Patterson, N. *et al.* Ancient Admixture in Human History. *Genetics* **192**, 1065-1093, doi:10.1534/genetics.112.145037 (2012).
30 Harald, R. *et al.* ancIBD - Screening for identity by descent segments in human ancient DNA. *bioRxiv*, 2023.2003.2008.531671, doi:10.1101/2023.03.08.531671 (2023).
31 Saag, L. *et al.* Genetic ancestry changes in Stone to Bronze Age transition in the East European plain. *Science Advances* **7**, eabd6535, doi:10.1126/sciadv.abd6535 (2021).
32 Papac, L. *et al.* Dynamic changes in genomic and social structures in third millennium BCE central Europe. *Science Advances* **7**, eabi6941, doi:doi:10.1126/sciadv.abi6941 (2021).
33 Underhill, P. A. *et al.* The phylogenetic and geographic structure of Y-chromosome haplogroup R1a. *European Journal of Human Genetics* **23**, 124-131, doi:10.1038/ejhg.2014.50 (2015).
34 González-Fortes, G. *et al.* Paleogenomic Evidence for Multi-generational Mixing between Neolithic Farmers and Mesolithic Hunter-Gatherers in the Lower Danube Basin. *Current Biology* **27**, 1801-1810.e1810, doi:10.1016/j.cub.2017.05.023 (2017).
35 Hofmanová, Z. *et al.* Early farmers from across Europe directly descended from Neolithic Aegeans. *Proceedings of the National Academy of Sciences* **113**, 6886-6891, doi:10.1073/pnas.1523951113 (2016).
36 Lazaridis, I. *et al.* Ancient human genomes suggest three ancestral populations for present-day Europeans. *Nature* **513**, 409-413, doi:10.1038/nature13673 (2014).
37 Lazaridis, I. *et al.* Ancient DNA from Mesopotamia suggests distinct Pre-Pottery and Pottery Neolithic migrations into Anatolia. *Science* **377**, 982-987, doi:10.1126/science.abq0762 (2022).

38 Lazaridis, I. *et al.* Genetic origins of the Minoans and Mycenaeans. *Nature* **548**, 214-218, doi:10.1038/nature23310 (2017).

39 Chintalapati, M., Patterson, N. & Moorjani, P. The spatiotemporal patterns of major human admixture events during the European Holocene. *eLife* **11**, e77625, doi:10.7554/eLife.77625 (2022).

40 Fenner, J. N. Cross-cultural estimation of the human generation interval for use in genetics-based population divergence studies. *Am J Phys Anthropol* **128**, 415-423, doi:10.1002/ajpa.20188 (2005).

41 Zhang, F. *et al.* The genomic origins of the Bronze Age Tarim Basin mummies. *Nature* **599**, 256-261, doi:10.1038/s41586-021-04052-7 (2021).

42 Skoglund, P. *et al.* Genomic diversity and admixture differs for Stone-Age Scandinavian foragers and farmers. *Science* **344**, 747-750, doi:10.1126/science.1253448 (2014).

43 Malmström, H. *et al.* The genomic ancestry of the Scandinavian Battle Axe Culture people and their relation to the broader Corded Ware horizon. *Proc Biol Sci* **286**, 20191528, doi:10.1098/rspb.2019.1528 (2019).

44 Coutinho, A. *et al.* The Neolithic Pitted Ware culture foragers were culturally but not genetically influenced by the Battle Axe culture herders. *American Journal of Physical Anthropology* **172**, 638-649, doi:https://doi.org/10.1002/ajpa.24079 (2020).

Supplementary File SI3

Unsupervised admixture modeling with summary individuals

Written by Iosif Lazaridis

Table SI3. 1. Average proportions of populations of Figure SI3. 4. 158
Table SI3. 2. Comparing ADMIXTURE and qpAdm admixture proportions. We show the *K*=4 admixture proportions of Core Yamna from Table SI3. 1 and the inferred *K*=4 proportions for the two alternative qpAdm models of Core Yamna...159

Figure SI3. 1. Unsupervised ADMIXTURE analysis..155
Figure SI3. 2. Supervised ADMIXTURE analysis. 156
Figure SI3. 3. Projection ADMIXTURE analysis. Core Yamna are projected; other populations are used in an unsupervised ADMIXTURE analysis to compute allele frequencies. 156
Figure SI3. 4. Unsupervised ADMIXTURE with a single pseudo-haploid Core Yamna summary individual. The summary individual is shown at the left side of the plot with a single wide bar.............158
Figure SI3. 5. Unsupervised ADMIXTURE of summary individuals from diverse populations. This figure summarizes the results of Table SI3. 1 as well as additional unsupervised ADMIXTURE runs in which the Core Yamna summary individual was replaced by a single summary individual for each of 97 additional populations...159
Figure SI3. 6. Projection of summary individuals and ADMIXTURE components onto World (left), West Eurasian (middle), and Steppe (right) PCA. 160
Figure SI3. 7. Upper Volga in the "Steppe" PCA. The summary and convex individuals of this population are close to each other, but the summary (blue) individual is slightly outside the convex hull of the four components. 161
Figure SI3. 8. Minimum distance of summary individuals to convex hull of four admixture components for the three PCAs. The minimum distance of points that fall outside the convex hull, even by a small amount are shown. 162
Figure SI3. 9. Unsupervised ADMIXTURE of summary individuals from diverse populations (clean version). 165
Figure SI3. 10. Modeling TUR_C_BA. TUR_C_BA is on the line between Çayönü and BPgroup; this corresponds to the qpAdm model we have developed for this population. When modeled in terms of the 4-source model it is modeled as a mixture of Anatolian (cross) and CHG (circle) ancestral populations. 166

Practitioners of ADMIXTURE analysis[1] know that as the number of ancestral populations (*K*) increases the algorithm often arrives at solutions in which a population of interest forms its own ancestral population. For example, the Kalash, a highly drifted genetic isolate from Pakistan form such a component[2] and are inferred to derive the majority of their ancestry from it with other populations deriving ancestry from this component.

This behavior is problematic for two reasons. First, the directionality of ancestry may be reversed; a highly drifted population may have the same types of ancestry as its neighbors but is shown to be "unadmixed" and contributing ancestry *to* them, rather than being admixed themselves. Second, while at lower *K* (before the emergence of the population-specific component) it may be possible to see what the ancestry of the highly drifted population is in terms of components shared with others, this ability is lost as soon as the population-specific cluster is inferred.

Thus, ADMIXTURE analysis relies on an element of "luck". The practitioner hopes that, for each population of interest, a population-specific component will *not* appear at a *K* value lower than the one for which all informative components of its ancestry have had the opportunity themselves to appear.

As a practical example, we show in Figure SI3. 1 an ADMIXTURE analysis of a set of populations that include diverse hunter-gatherers and Eneolithic/Bronze Age populations representing ancestry of the Pontic-Caspian steppe and of the Near East and Caucasus (from this study and ref.[3]). At *K*=2 populations of European hunter-gatherers (orange) are differentiated from those of the Near East (blue; both Anatolia and Iran/Caucasus). At *K*=3 European hunter-gatherers are split between Balkan hunter-gatherers (orange) and Eastern hunter-gatherers (yellow). The Core Yamna have divided affinities at these first *K*=2, 3 consistent with admixed origins of "north" (European hunter-gatherer) and "south" (Near Eastern) components (*K*=2), and their European component is predominantly EHG and not BHG (*K*=3).

At *K*=4, however, a population component (pink) specific to the Core Yamna appears, and this appears to contribute to much older and remote populations, such as Neolithic Iran, Caucasus hunter-gatherers and the Eneolithic people of both Khvalynsk and Serednii Stih. Clearly, we have a case here in which the pink "Yamna"-centered component is spurious; the algorithm set it to maximum global likelihood because there is a large number of homogeneous Yamna individuals in the dataset. Notice also the conflicting information regarding the hunter-gatherer admixture of the Core Yamna suggested by *K*=3 and *K*=4. The hunter-gatherer component at *K*=3 is almost entirely EHG (yellow); this ancestry is "absorbed" by the Yamna-specific component at *K*=4 which is then shown to require some extra BHG (orange) ancestry.

At *K*=5, an informative component does appear: Near Eastern populations, previously represented by the "blue" component are now split to Anatolian-related (blue) and Caucasus/Iran-related (green). It would be great if we could assess the ancestry of the Core Yamna in terms of these components, but we are unable to do so as the Yamna at *K*=5 continue to belong virtually completely from their population-specific "pink" component, which becomes even more specific to them as it swallows up the all the BHG-related ancestry of the preceding *K*=4 run and many Yamna individuals belong ~100% to it.

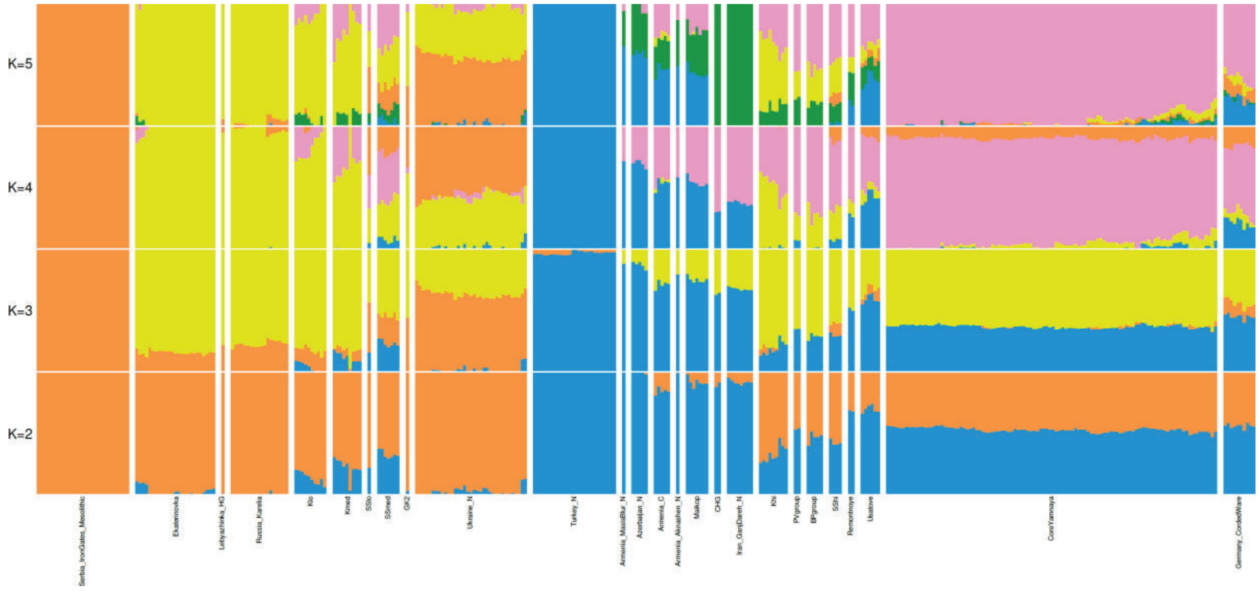

Figure SI3. 1. Unsupervised ADMIXTURE analysis.

A way to overcome this problem is to use a *single* individual from the entire population to represent it. We may create such an individual in different ways; a simple method (which we adopt here) is to simply take all individuals belonging to it and pick an allele at random for each genomic position creating a single pseudo-haploid individual. Another possible approach is to take a single high-quality individual from the population; the drawbacks of this method is that (a) it has slightly less data than using all the individuals, and (b) we are at some risk of picking an individual that systematically differs in some way from the population as a whole. A third approach addresses concern (b) by iterating over *all* individuals of the population.

Supervised ADMIXTURE

A possible objection to the procedure suggested in the previous paragraph is to use *supervised* ADMIXTURE analysis instead. There are a few drawbacks to that approach. First, it requires one to posit a priori what the source populations are. Second, any inhomogeneities in the source populations cannot be addressed: if a population has ancestral variation then by using it as a source in supervised ADMIXTURE then all its individuals are assigned 100% ancestry from its own source; there is no latitude to allow this proportion to vary to cover this variation. Third, it uses information only from the limited number postulated ancestral populations and cannot use data from other populations composed of the same kinds of ancestry.

Fourth, in our practice we notice that the numerical convergence of supervised ADMIXTURE is often to solutions as shown in Figure SI3. 2 in which we use the same dataset as in Figure SI3. 1 but use CHG, Turkey_N, Russia_Karelia, and Serbia_IronGates_Mesolithic as the four sources in a supervised analysis. This solution shows the Core Yamna to have 84.9% (on average) of the CHG component which is much higher amount of "southern" ancestry inferred with either qpAdm or with the unsupervised ADMIXTURE of Figure SI3. 1 (37-53% in K=2, 3, prior to the appearance of the Yamna-centered component). Bias of supervised ADMIXTURE estimates in a different setting was also observed in ref.[4]

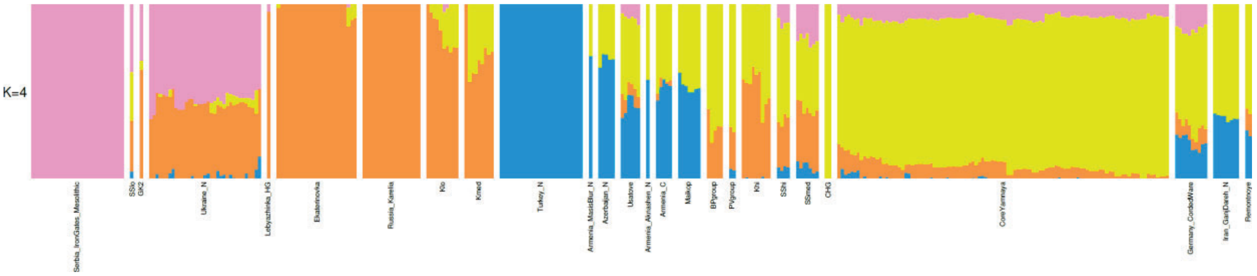

Figure SI3. 2. Supervised ADMIXTURE analysis.

<u>Projection ADMIXTURE</u>

ADMIXTURE can also be run in *projection mode*. This uses the allele frequency (*.P) files of an unsupervised ADMIXTURE analysis on one dataset to estimate the ancestry proportions of a second (test) dataset. An advantage of this method over supervised ADMIXTURE is that it uses more individuals than a pre-set number of source populations and makes no assumptions about their homogeneity.

Its main disadvantage is that the individuals used in the initial unsupervised analysis contribute to the allele frequencies, while the test individuals are treated differently (and do not contribute to the allele frequencies). This leaves room for potential bias, as the test individuals are not treated in the same manner as the ones used to compute the allele frequencies. Another potential problem is that the test individuals might have ancestry not represented in the unsupervised ADMIXTURE set; by projecting them using the allele frequencies computed from that set we are constraining them to be modeled using only the kinds of ancestry present there.

We used projection ADMIXTURE as follows: first, we computed allele frequencies using unsupervised ADMIXTURE on the populations of Figure SI3. 1 (excluding the Core Yamna), and then used these to project all the populations (including the Core Yamna). The results are shown in Figure SI3. 3.

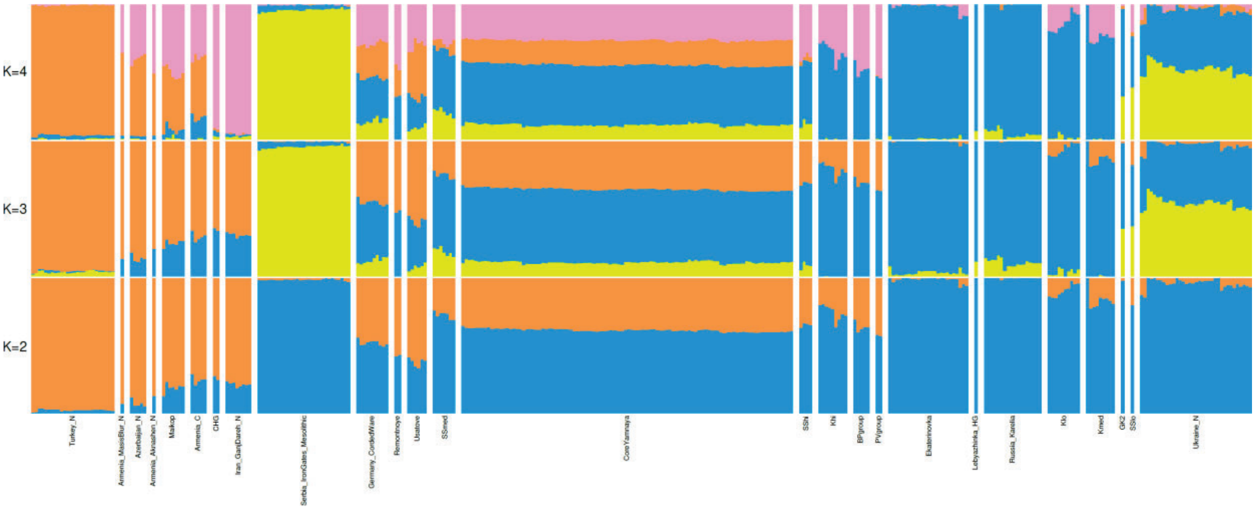

Figure SI3. 3. Projection ADMIXTURE analysis. Core Yamna are projected; other populations are used in an unsupervised ADMIXTURE analysis to compute allele frequencies.

Unsupervised ADMIXTURE with a Core Yamna summary individual

Thus, we adopt the single pseudo-haploid individual method as it does not have the concerns (a) or (b) of unsupervised ADMIXTURE or the potential bias problems of unsupervised/projection ADMIXTURE. The main drawback of the single pseudo-haploid individual method is that it collapses the population into a single individual, thus potentially masking any heterogeneity in it. However, we have validated the homogeneity of the Core Yamna in various ways (by the manner in which this population was assembled by showing that its high quality (at least 400K 1240k SNPs) individuals cluster with Samara Yamna in PCA and also are cladal with it to a high degree of certainty ($p \geq 0.2$) using qpWave[5]). Thus, we are confident that excessive heterogeneity or substantial outlier individuals do not exist among the 104 Core Yamna individuals.

The results of our method are shown in Figure SI3. 4. As is clear, there is no Yamna-specific cluster that appears in either K=3 or K=4 and thus we can assess the ancestry of the Core Yamna in terms of the other components. The averages of the populations of Figure SI3. 4 are shown in Figure SI3. 1.

Comparison of unsupervised ADMIXTURE with qpAdm and with projection ADMIXTURE

It is interesting to compare these inferred proportions with those inferred using qpAdm.[6] Recall that Core Yamna is modeled as either SShi+Remontnoye (SShi representing the Eneolithic Serednii Stih and Remontnoye the extra CLV ancestry) or (Aknashen+BPgroup)+GK2 (Aknashen+BPgroup representing the total CLV ancestry and GK2 the NPR hunter-gatherer ancestry). In Figure SI3. 2 we show this comparison which indicates that the two methods agree with each other within 0.3-3.1% for either of the qpAdm models and all K=4 components. This fairly close agreement provides independent confirmation using a non-*f*-statistics based method of the qpAdm reconstruction of the ancestry of the Core Yamna.

(Note that some slight differences may be inherent between the two methods as qpAdm is a supervised method in which the source populations are proposed a priori while in unsupervised ADMIXTURE they are discovered from the data and have imperfect correspondences to the sampled populations. In particular the EHG-centered component inferred by admixture is clear but may not be entirely corresponding to the sampled EHG as, for example, it is also dominated by the large sample size of individuals from Ekaterinovka which are inferred using qpAdm to have most (but not all) of their ancestry from EHG. Similarly, the CHG and Ganj Dareh Neolithic dominate the CHG-centered component, while it is known that these two populations are not identical to each other and CHG has more affinity to European hunter-gatherers.[7])

We include in Figure SI3. 2 the ADMIXTURE proportions obtained from projection ADMIXTURE (Figure SI3. 3). These differ from the unsupervised ADMIXTURE/qpAdm-inferred range substantially and especially for the Anatolian/CHG-related components, overestimating the former and underestimating the latter. So, while projection ADMIXTURE usefully shows the presence of all four components in the Core Yamna, there is reason to think that its different treatment of projected individuals introduces some bias.

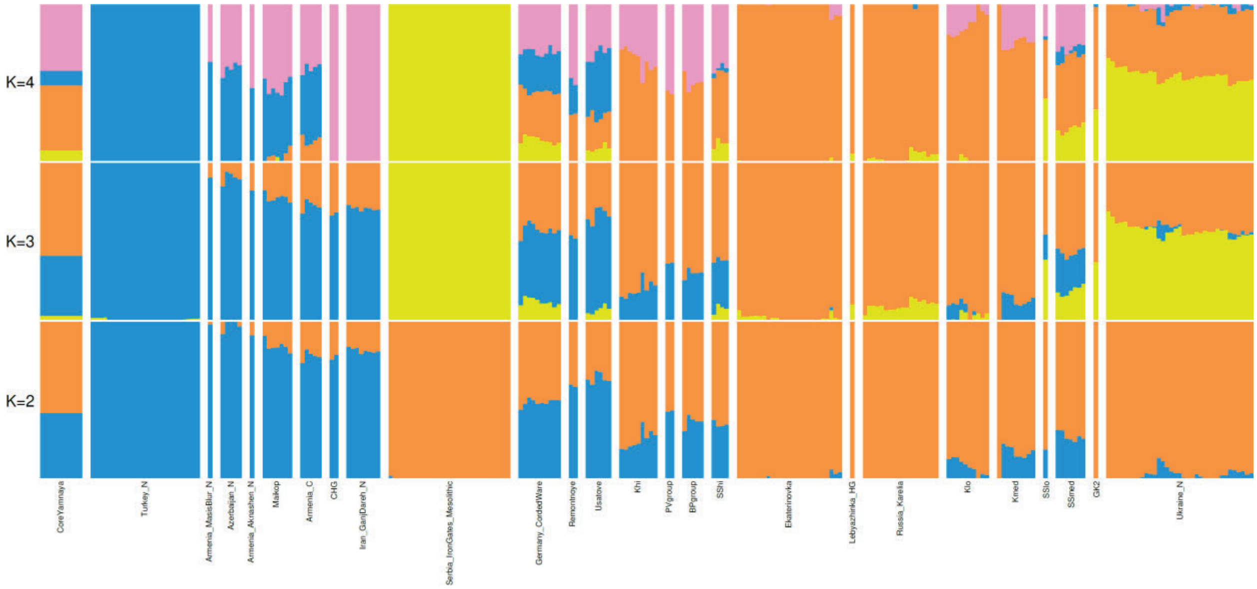

Figure SI3. 4. Unsupervised ADMIXTURE with a single pseudo-haploid Core Yamna summary individual. The summary individual is shown at the left side of the plot with a single wide bar.

Table SI3. 1. Average proportions of populations of Figure SI3. 4.

Population	*N*	Yellow BHG	Orange EHG	Blue Anatolian Neolithic	Pink CHG-Iran
Armenia_Aknashen_N	1	0.0%	0.0%	46.8%	53.2%
Armenia_C	5	0.0%	13.2%	46.2%	40.6%
Armenia_MasisBlur_N	1	0.0%	0.0%	63.4%	36.6%
Azerbaijan_N	5	0.0%	0.0%	59.2%	40.8%
BPgroup	5	0.0%	50.3%	0.0%	49.7%
CHG	2	0.0%	0.0%	0.0%	100.0%
CoreYamna	1	6.8%	41.7%	9.2%	42.3%
Ekaterinovka	25	0.1%	98.9%	0.0%	1.0%
Germany_CordedWare	10	13.4%	31.2%	25.2%	30.2%
GK2	1	33.1%	65.6%	1.4%	0.0%
Iran_GanjDareh_N	8	0.0%	0.0%	0.0%	100.0%
Khi	9	0.0%	64.7%	0.0%	35.3%
Klo	10	0.7%	87.0%	0.0%	12.4%
Kmed	9	0.0%	78.0%	0.0%	22.0%
Lebyazhinka_HG	1	4.6%	95.4%	0.0%	0.0%
Maykop	7	0.4%	3.0%	44.0%	52.6%
PVgroup	2	0.0%	44.1%	0.0%	55.9%
Remontnoye	2	0.0%	30.1%	20.9%	49.0%
Russia_Karelia	18	2.5%	97.4%	0.2%	0.0%
Serbia_IronGates_Mesolithic	29	100.0%	0.0%	0.0%	0.0%
SShi	4	11.1%	45.4%	2.9%	40.6%
SSlo	1	40.0%	37.8%	2.0%	20.2%
SSmed	7	20.6%	46.0%	5.5%	27.9%
Turkey_N	26	0.0%	0.0%	100.0%	0.0%
Ukraine_N	35	54.9%	42.4%	1.6%	1.1%
Usatove	6	8.1%	21.2%	39.5%	31.2%
Armenia_Aknashen_N	1	0.0%	0.0%	46.8%	53.2%

Table SI3. 2. Comparing ADMIXTURE and qpAdm admixture proportions. We show the *K*=4 admixture proportions of Core Yamna from Table SI3. 1 and the inferred *K*=4 proportions for the two alternative qpAdm models of Core Yamna.

Population	BHG	EHG	Anatolian	CHG
CoreYamna	6.8%	41.7%	9.2%	42.3%
0.263*Remontnoye+0.737*SShi	8.2%	41.4%	7.6%	42.8%
0.207*Aknashen+0.568*BPgroup+0.225*GK2	7.4%	43.3%	10.0%	39.2%
CoreYamna (projection ADMIXTURE)	10.9%	44.4%	18.4%	26.3%

Unsupervised ADMIXTURE analysis with summary individuals from diverse populations

We were encouraged by the preceding results to use the same method with summary individuals from populations other than those included in the unsupervised ADMIXTURE analysis. This would allow us to assess their ancestry in terms of the same model as we did for the Core Yamna summary individual. Thus, we repeated the ADMIXTURE analysis, creating summary individuals for each population of interest. The results are shown in Figure SI3. 5.

Naturally, the solution for some of the summary individuals may not be a valid representation of its ancestry. For example, when using OldAfrica or Han.DG as the test populations, this is coerced into the ancestral components generated by the West Eurasian individuals used in the ADMIXTURE analysis.

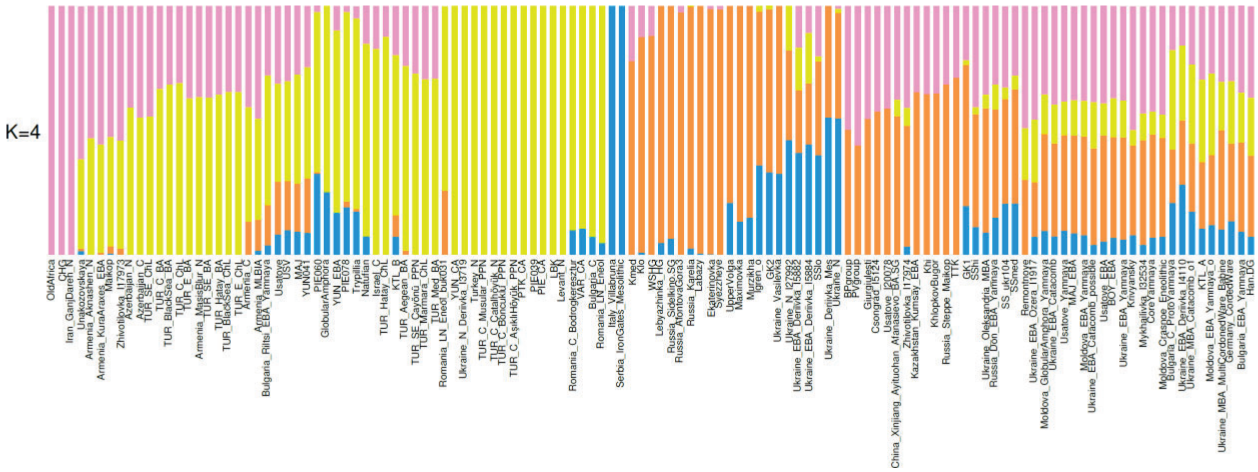

Figure SI3. 5. Unsupervised ADMIXTURE of summary individuals from diverse populations. This figure summarizes the results of Table SI3. 1 as well as additional unsupervised ADMIXTURE runs in which the Core Yamna summary individual was replaced by a single summary individual for each of 97 additional populations.

Removing populations for which the 4-source model is inapplicable

To address the problem of whether individuals are correctly modeled in terms of the four ancestral components, we took the following approach. We performed three principal component analyses, including a "World PCA", a "West Eurasian" PCA[8] and a "Steppe" PCA (as in the main PCA of the paper). In each of these PCA we project the summary individuals for each population as well as summary individuals for the four admixture components; the latter can be generated by examining the *.P (allele frequency) output of ADMIXTURE and picking an allele at random for each position.

These results are shown for OldAfrica and Core Yamna in Figure SI3. 6. Note that in all these plots the ADMIXTURE components are represented by the run of Figure SI3. 4; for each ADMIXTURE run, the *.P files differ from each other, even if they converge to a similar solution. Each plot shows the "convex" (red) individual as a summary of the population given the estimated admixture proportions. It also shows the "summary" (blue) individual itself. Notice that in both "World" and "West Eurasian" PCA plots, the Old African "summary" individual is well beyond the "convex" one: this proves that the modeling of Old Africa in terms of the 4-source model (which is ~100% of the CHG-related component; Figure SI3. 5) is inadequate: Old Africa is squeezed into the components of this model even though in fact it has quite different ancestry. By contrast, the "convex" and "summary" individuals for the Core Yamna are in proximity to each other, and within the convex hull of the four components: the Core Yamna can indeed be modeled in terms of the four components and their modeling ("convex") is in proximity to their actual population ("summary").

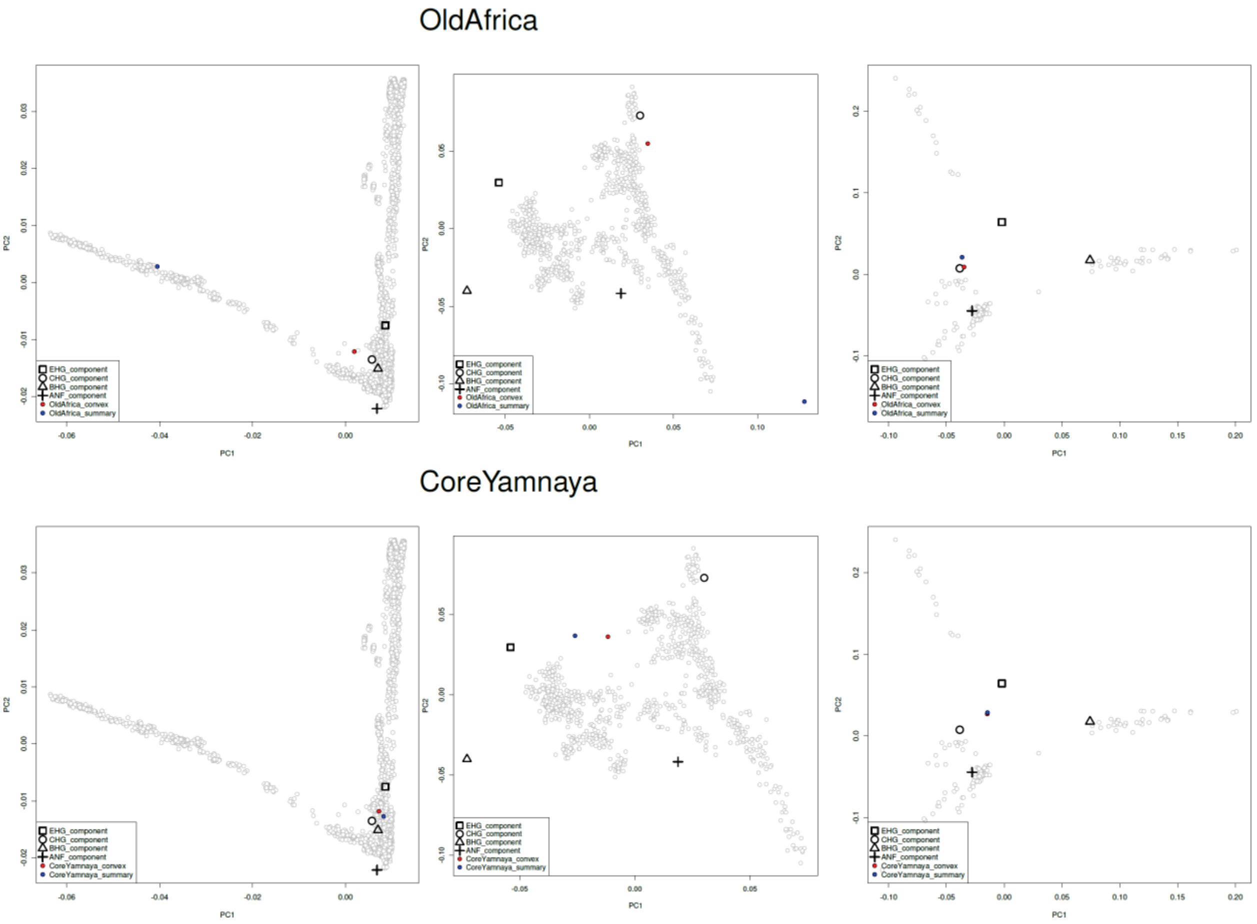

Figure SI3. 6. Projection of summary individuals and ADMIXTURE components onto World (left), West Eurasian (middle), and Steppe (right) PCA.

The approach we took here could be generalized to include other statistics; the basic idea is simple: we use the summary individual and the convex combination of the ADMIXTURE components to see if they agree with each other.

The three PCAs capture different aspects of genetic variation of the populations of interest and are a sufficient filter for our purposes of removing from Figure SI3. 5 those populations which cannot be well-modeled in terms of the four sources. This can be done either by examining the PCAs visually and making a subjective assessment or in an automated way. For “Old Africa” common sense and the visual assessment of Figure SI3. 6 are in agreement that this is a population outside the 4-source model; this may be less clear for other populations. We thus adopted an automated approach as follows.

First, we accept populations whose summary individual is *within* the convex hull of the four ancestral components. From the point of view of the three PCAs, the summary individual *can* be modeled as a convex combination of the four components.

This criterion may be too stringent, however, for populations that occupy the edges of the convex hull. For example, if a population is a BHG/EHG mixture in reality then its summary representation should be exactly on the BHG-EHG edge of the convex hull. However, because of stochastic variation it may, in fact be either within, or slightly outside the convex hull. An example of precisely such a population is Upper Volga (Figure SI3. 7).

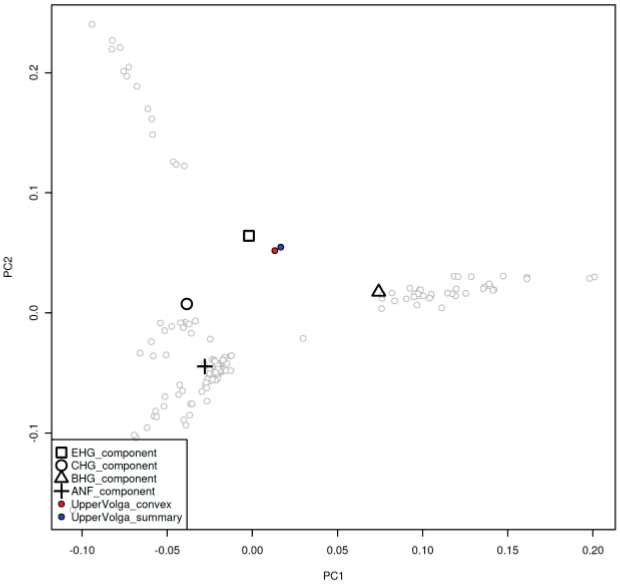

Figure SI3. 7. Upper Volga in the “Steppe” PCA. The summary and convex individuals of this population are close to each other, but the summary (blue) individual is slightly outside the convex hull of the four components.

We can address this issue empirically by examining the minimum distance of a summary individual to the convex hull. This is the minimum distance to the (four) edges of the convex hull. We adapted pseudo-code for this problem (https://stackoverflow.com/a/6853926) to R and can thus compute the minimum distance for all populations that fall outside the convex hull. We show these in Figure SI3. 8.

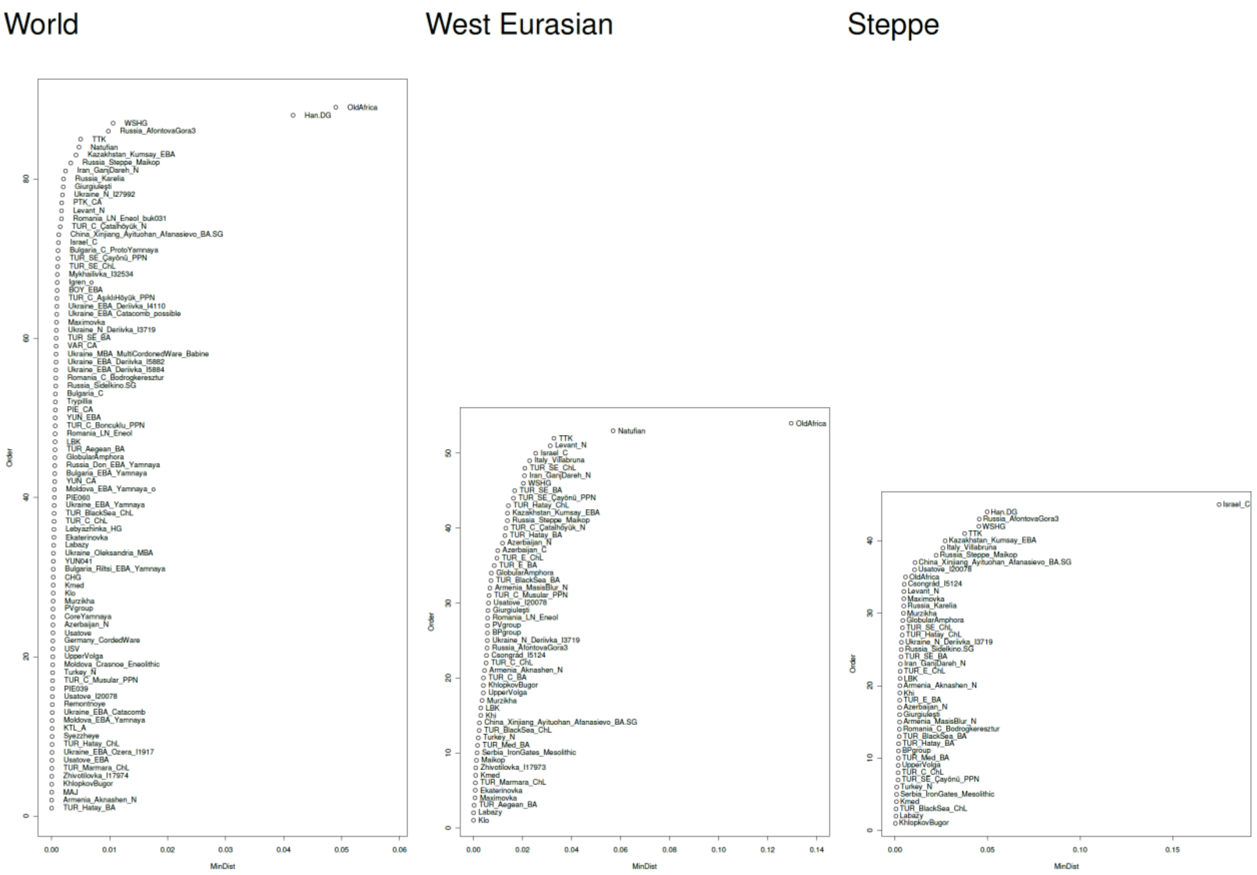

Figure SI3. 8. Minimum distance of summary individuals to convex hull of four admixture components for the three PCAs. The minimum distance of points that fall outside the convex hull, even by a small amount are shown.

The most detached outliers are, as we might expect, Old Africa and Han Chinese in the World PCA. Natufians are a clear outlier in the West Eurasian PCA; this makes sense as the 4-source model does not have a Levantine source and is thus not suitable for ancient Levantine populations, of which the Epipaleolithic Natufians are most representative.[7,9] Israel_C is an outlier for the Steppe PCA; this too could be because of the Levantine ancestry of this population[10] but may also be a consequence of the fact that Israel_C is one of the populations used to form the axes of the Steppe PCA, in contrast to the "World" and "West Eurasian" PCAs that are formed on the basis of modern populations and are thus "neutral" with respected to all the ancient ones.

Other populations that are visibly at the top of the minimum distance plots include those of Siberia (such as Afontova Gora 3) or having ancestry related to it (such as the Neolithic TTK from Tajikistan[11] or the Steppe Maykop[12]).

Our final decision was to omit several populations aggressively that are in the top of Figure SI3. 8 and to keep those that form the undifferentiated set of low-MinDist populations at the bottom. The omitted populations are:

Azerbaijan_C, Azerbaijan_N, China_Xinjiang_Ayituohan_Afanasievo_BA.SG, Han.DG, Iran_GanjDareh_N, Israel_C, Italy_Villabruna, Kazakhstan_Kumsay_EBA, Levant_N, Natufian,

OldAfrica, Russia_AfontovaGora3, Russia_Steppe_Maykop, TTK, TUR_C_Çatalhöyük_N, TUR_E_BA, TUR_E_ChL, TUR_Hatay_BA, TUR_Hatay_ChL, TUR_SE_BA, TUR_SE_Çayönü_PPN, TUR_SE_ChL, Usatove_I20078, WSHG

Omitting these populations from Figure SI3. 5 we plot the remaining ones in Figure SI3. 9.

We highlight some contrasts in these results that correspond to the findings of our formal qpWave/qpAdm analysis.

1. The CLV Cline: its northern end (BPgroup) is a mix of CHG/EHG related components; its southern end (Aknashen) is a mix of CHG/Anatolian ancestry. Intermediate populations (Maykop and Remontnoye) have all three components.
2. The Volga Cline: Khvalynsk subsets (Khi, Klo, Kmed) and Ekaterinovka have more EHG ancestry than the southern end of the cline (BPgroup). Migrants from the Volga cline across the NPR at Giurgiuleşti and Csongrád represent a similar balance of components to the BPgroup and low-EHG end of the Volga Cline.
3. The Dnipro Cline: Serednii Stih subsets (SShi, SSlo, SSmed) and Yamna have some BHG-related ancestry not found in the CLV Cline, but present in NPR hunter-gatherers and their descendants such as Ukraine_N and GK2. Thus, the Dnipro Cline could have formed on the basis of CLV migrants admixing with NPR hunter-gatherers as our other analyses indicate.
4. The second major admixture process in the NPR was what formed the Usatove/USV/MAJ-related populations; these visibly differ from the Serednii Stih/Yamna in having much Anatolian-related ancestry.
5. The proximal source of the Anatolian-related ancestry in the Usatove could be the Trypillians; these also have a little CHG/EHG-related ancestry, consistent with our reconstruction that they absorbed some CLV migrants as well, but to a lesser degree than the Usatove.
6. Populations of Armenia provide an interesting control for our reconstruction, paralleling what is known of the history of the country[13]. First, Aknashen/Masis Blur differ from each other in being more/less CHG-admixed and both lack any steppe influence. The Areni-1 population (Armenia_C) receives EHG-related ancestry. This disappears in the Kura Araxes period and re-appears after the Middle Bronze Age (Armenia_MLBIA) in which it is accompanied by a smidgeon of BHG-related ancestry. That the Armenia_MLBIA population had input from the Yamna is certain because of Y-chromosome evidence[13]. The ADMIXTURE results nicely parallel this, by showing that the early steppe influence (at Areni-1) was CLV-like, while the later, post-2500BCE one included also the BHG-related ancestry present in the Yamna.

Finally, we highlight the limitations of this approach with the concrete example of the Central Anatolian Bronze Age population; we modeled this as a mixture of Çayönü Pre-Pottery Neolithic and steppe populations like BPgroup. However, in Figure SI3. 9 it is modeled as Anatolian and CHG components without any steppe ancestry. Notice that the Çayönü population was excluded Figure SI3. 8 because its predicted position is outside the convex hull of the four admixture components in the West Eurasian PCA. We can see this in Figure SI3. 10. Çayönü is indeed outside the convex hull of the four ancestral components; however, TUR_C_BA is indeed on the line connecting Çayönü with BPgroup; thus, the PCA is consistent with its modeling as such a mixture.

In conclusion, our development of unsupervised ADMIXTURE analysis of summary individuals in this note provides a powerful new way of assessing ancestry that complements the results of qpAdm. The four sources of the unsupervised ADMIXTURE model lead to similar conclusions as our qpAdm analysis

about the formation of populations of the steppe and North Pontic region. However, they should always be interpreted with caution as even populations of much different ancestry (like “Han”) will be forced to have their ancestry from the sources of the model (Figure SI3. 5). This violation can be detected by comparing summary individuals with their representation in a different statistic, such as PCA (Figure SI3. 8). However, even that procedure may miss cases (such as TUR_C_BA) which are seemingly well-modeled by unsupervised ADMIXTURE but in fact may possess ancestry other than what can be expressed in the unsupervised model (such as Çayönü-related ancestry; Figure SI3. 10).

Thus, we end this note by cautioning the reader that the unsupervised ADMIXTURE procedure developed here provides a powerful new tool of ancestry analysis that overcomes the problem of spurious population-centered components, but its results should be interpreted in the context of other lines of evidence (such as PCA and qpAdm) to arrive at a consistent reconstruction.

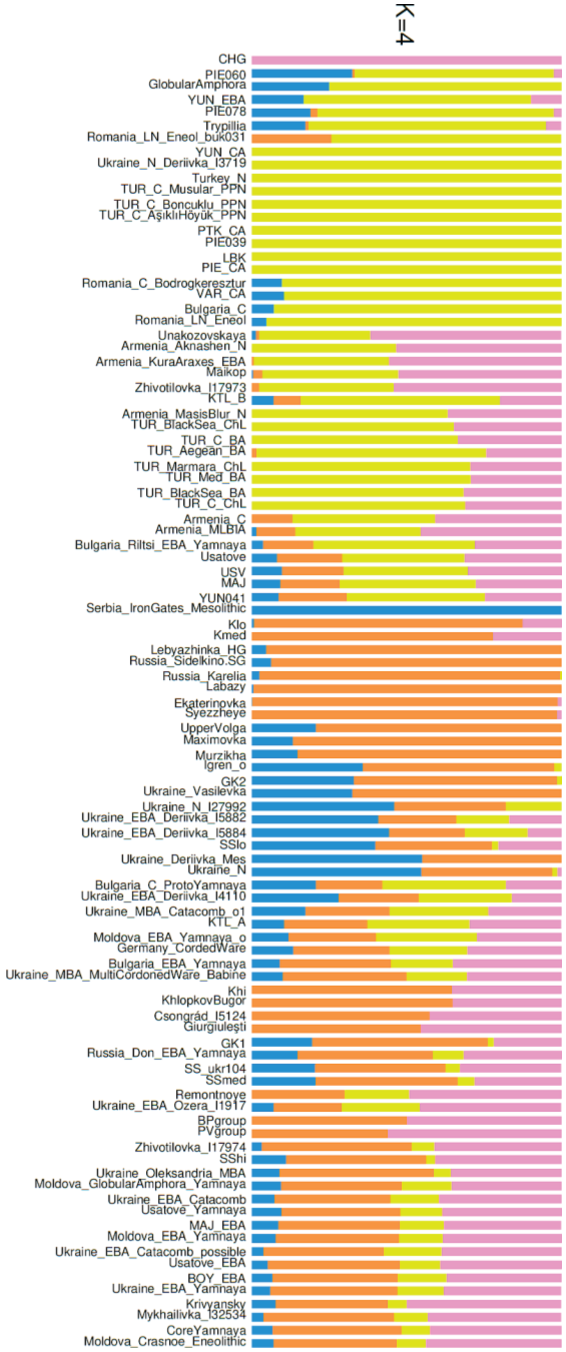

Figure SI3. 9. Unsupervised ADMIXTURE of summary individuals from diverse populations (clean version).

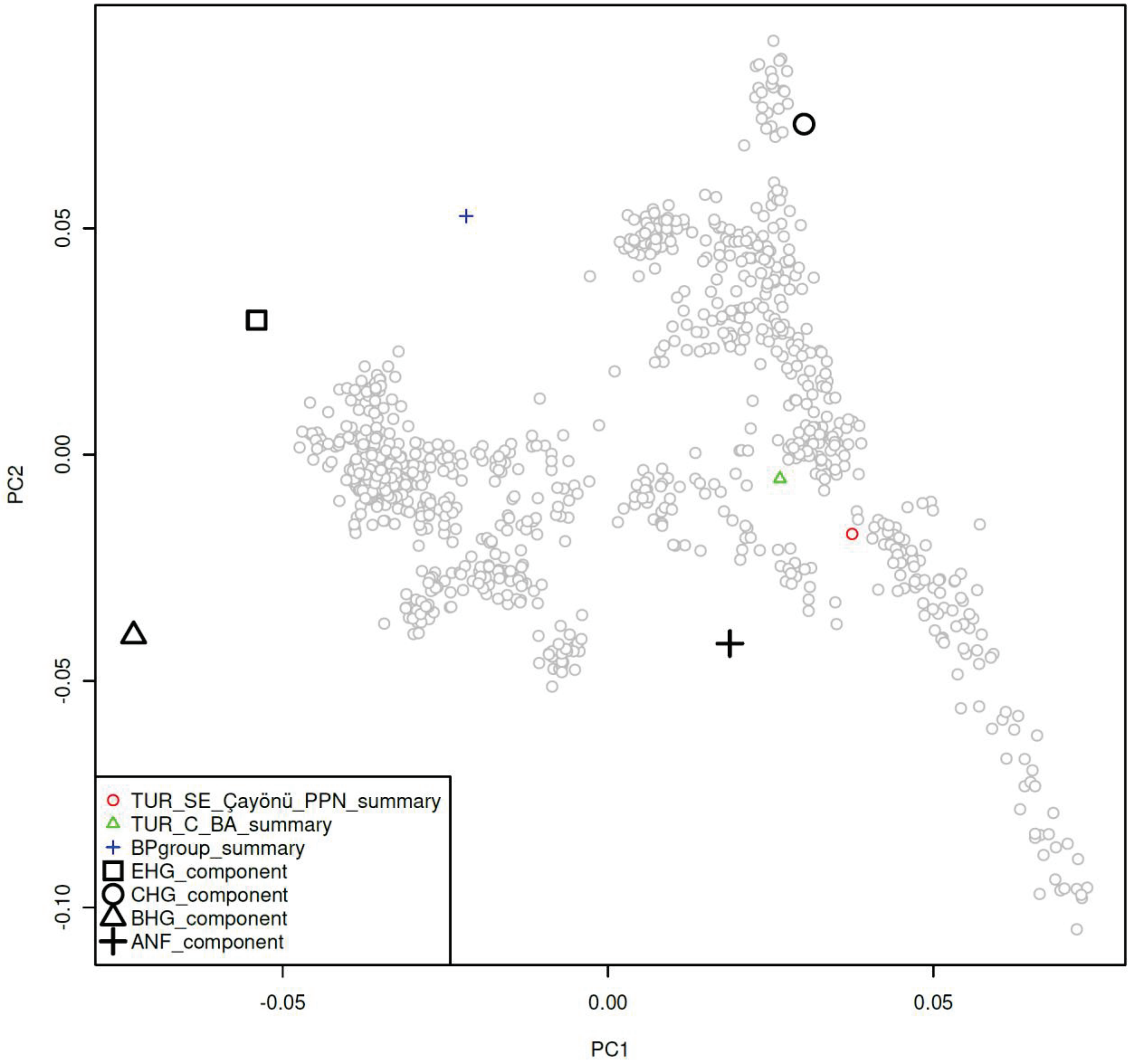

Figure SI3. 10. Modeling TUR_C_BA. TUR_C_BA is on the line between Çayönü and BPgroup; this corresponds to the qpAdm model we have developed for this population. When modeled in terms of the 4-source model it is modeled as a mixture of Anatolian (cross) and CHG (circle) ancestral populations.

References

1 Alexander, D. H., Novembre, J. & Lange, K. Fast model-based estimation of ancestry in unrelated individuals. *Genome Research* **19**, 1655-1664, doi:10.1101/gr.094052.109 (2009).

2 Ayub, Q. *et al.* The Kalash Genetic Isolate: Ancient Divergence, Drift, and Selection. *The American Journal of Human Genetics* **96**, 775-783, doi:https://doi.org/10.1016/j.ajhg.2015.03.012 (2015).

3 Lazaridis, I., Patterson, N., Anthony, D. & others. The Genetic Origin of the Indo-Europeans. *in submission* (2024).

4 Lazaridis, I. & Reich, D. Failure to replicate a genetic signal for sex bias in the steppe migration into central Europe. *Proceedings of the National Academy of Sciences* **114**, E3873-E3874, doi:10.1073/pnas.1704308114 (2017).
5 Reich, D. *et al.* Reconstructing Native American population history. *Nature* **488**, 370-374, doi:10.1038/nature11258 (2012).
6 Haak, W. *et al.* Massive migration from the steppe was a source for Indo-European languages in Europe. *Nature* **522**, 207-211, doi:10.1038/nature14317 (2015).
7 Lazaridis, I. *et al.* Genomic insights into the origin of farming in the ancient Near East. *Nature* **536**, 419-424, doi:10.1038/nature19310 (2016).
8 Lazaridis, I. *et al.* Ancient human genomes suggest three ancestral populations for present-day Europeans. *Nature* **513**, 409-413, doi:10.1038/nature13673 (2014).
9 Lazaridis, I. *et al.* Ancient DNA from Mesopotamia suggests distinct Pre-Pottery and Pottery Neolithic migrations into Anatolia. *Science* **377**, 982-987, doi:10.1126/science.abq0762 (2022).
10 Harney, É. *et al.* Ancient DNA from Chalcolithic Israel reveals the role of population mixture in cultural transformation. *Nature Communications* **9**, 3336, doi:10.1038/s41467-018-05649-9 (2018).
11 Posth, C. *et al.* Palaeogenomics of Upper Palaeolithic to Neolithic European hunter-gatherers. *Nature* **615**, 117-126, doi:10.1038/s41586-023-05726-0 (2023).
12 Wang, C.-C. *et al.* Ancient human genome-wide data from a 3000-year interval in the Caucasus corresponds with eco-geographic regions. *Nature Communications* **10**, 590, doi:10.1038/s41467-018-08220-8 (2019).
13 Lazaridis, I. *et al.* The genetic history of the Southern Arc: A bridge between West Asia and Europe. *Science* **377**, eabm4247, doi:10.1126/science.abm4247 (2022).
